# Triple targeting of mutant EGFR^L858R/T790M^, COX-2, and 15-LOX: design and synthesis of novel quinazolinone tethered phenyl urea derivatives for anti-inflammatory and anticancer evaluation

**DOI:** 10.1080/14756366.2023.2199166

**Published:** 2023-04-11

**Authors:** Hend Kothayer, Samar Rezq, Ahmed S. Abdelkhalek, Damian G. Romero, Samar S. Elbaramawi

**Affiliations:** a Department of Medicinal Chemistry, Faculty of Pharmacy, Zagazig University, Zagazig, Egypt; b Department of Pharmacology and Toxicology, Faculty of Pharmacy, Zagazig University, Zagazig, Egypt; c Department of Cell and Molecular Biology, University of Mississippi Medical Center, Jackson, MS, USA; d Mississippi Center of Excellence in Perinatal Research, University of Mississippi Medical Center, Jackson, MS, USA; e Women’s Health Research Center, University of Mississippi Medical Center, Jackson, MS, USA; f Cardiovascular-Renal Research Center, University of Mississippi Medical Center, Jackson, MS, USA

**Keywords:** Triple EGFR/COX-2/15-LOX inhibitors, quinazoline, phenyl urea, *in vitro* anti-inflammatory, anticancer

## Abstract

We designed and synthesised novel quinazolinone tethered phenyl urea derivatives (**6a–p**) that triple target the double mutant EGFR^L858R/T790M^, COX-2, and 15-LOX. Compounds (**6e**, **6d**, **6j**, **6m**, and **6n**) not only had low micromolar IC50 inhibitory activities against the three targets, but they also showed good selectivity for COX-2 over COX-1 and for EGFR^L858R/T790M^ over wild-type EGFR. Except for **6e** and **6n**, all of the tested compounds inhibited the NO production significantly more potently than celecoxib, diclofenac, and indomethacin. Compounds **6i** and **6k** reduced ROS levels more effectively than celecoxib and diclofenac. In terms of inhibiting TNF-α production, **6o-**treated cells showed TNF-α level, which is ∼10 times lower than celecoxib. Furthermore, **6e** and **6j** had the highest anticancer activity against the breast cancer cell line BT-459 with growth inhibition percentages of 67.14 and 70.07%, respectively. Docking studies confirm their favoured binding affinity. The proposed compounds could be promising multi-targeted leads.

## Graphical Abstract

**Figure:**
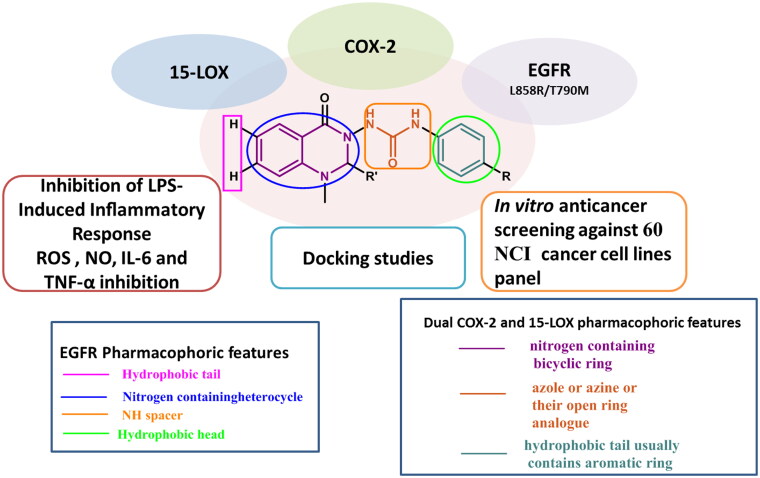

## Introduction

Despite the intense efforts in the development of anticancer drugs, cancer still ranks as one of the most lethal diseases worldwide^1^^,^^2^. Moreover, the development of resistance to some highly effective anticancer agents remains a major challenge in cancer therapy^3^. Consequently, the multifactorial nature of cancer has moved the field towards the design of multi-targeted anticancer agents to overcome the resistance problem^4^.

Anticancer agents with multi-targeted mechanisms not only provide an effective strategy for the treatment of cancers with a low incidence of resistance, but they also avoid the issues of drug-drug interaction and dose-limiting toxicity that are common in combination therapy^4^. Many multi-targeted anticancer agents have succeeded to reach preclinical and clinical stages^3^. As a result, the multi-target-single-agent strategy has become a mainstream approach for cancer treatment, and researchers have become increasingly interested in developing novel multi-target-single-agent drugs in recent years^2^.

**Figure 1.**
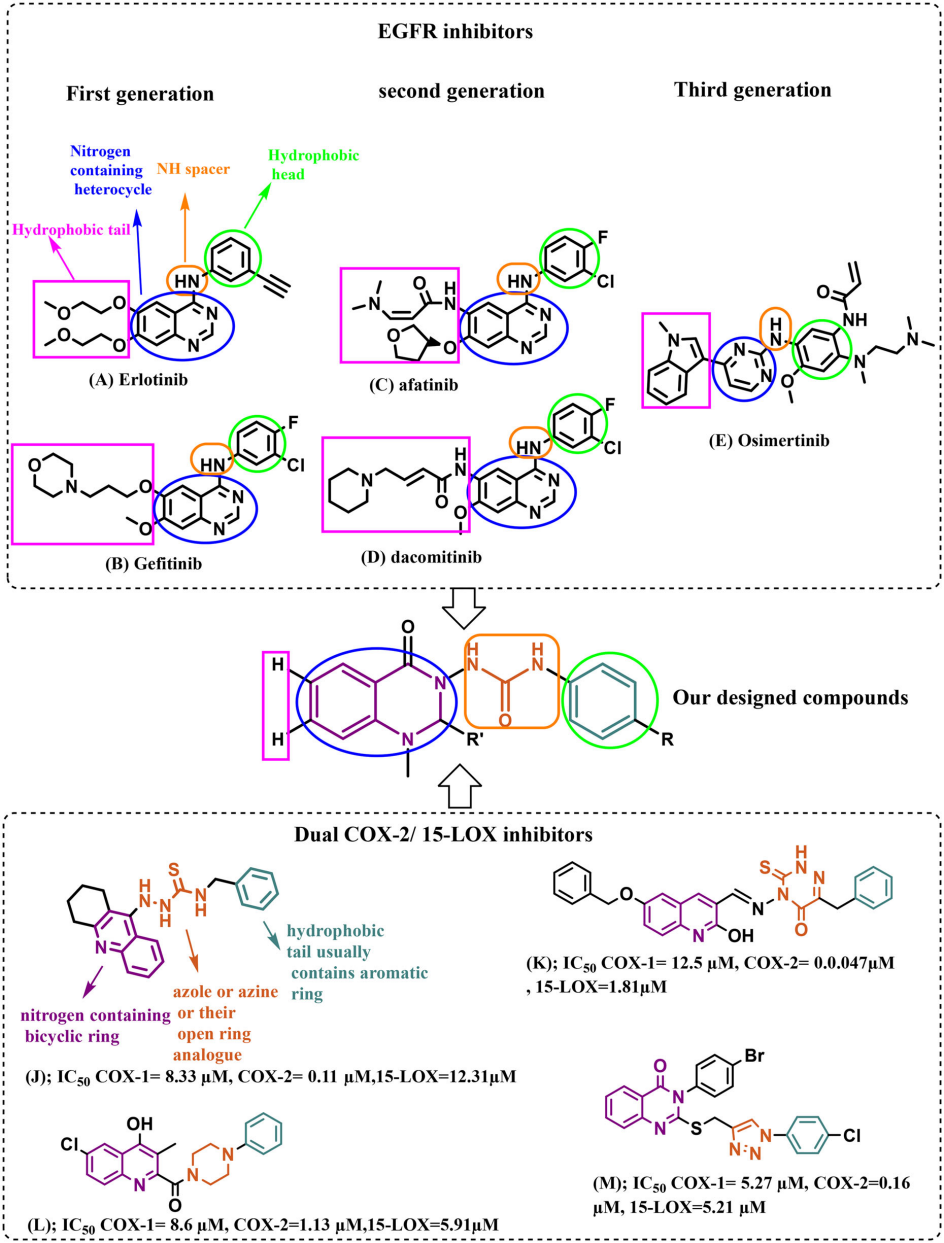
EGFR inhibitors (first, second, and third generations), some reported compounds as dual COX-2/15-LOX inhibitors, and the rationale for the design of our multi-target directed ligands (MTDLs).

Epidermal growth factor receptor (EGFR) is a protein kinase that is overexpressed in a variety of human solid tumours, including pancreatic, colorectal, non-small cell lung (NSCLC), renal cell carcinoma (RCC), ovarian, breast, and head and neck cancers^2^^,^^5^. Thus EGFR inhibitors have become widely used for the treatment of those cancers. Although many tumours show an initial response to the treatment with EGFR inhibitors, resistance to treatment often occurs due to point mutations in the ATP binding pocket of EGFR, such as T790M, L858R, and C797S. These mutations have resulted in the development of three generations of EGFR tyrosine kinase inhibitors (TKIs)^2^^,^^6^. EGFR classic mutations are in-frame deletions in exon 19 and an L858R point mutation in exon 21. First-generation EGFR inhibitors (4- amino quinazolines) erlotinib (**A**) and gefitinib (**B**) were efficacious for these classic mutations (Figure 1). However, resistance develops after the administration of first-generation EGFR TKIs due to a secondary mutation where a bulkier methionine replaces threonine at position 790 (gate keeper) (T790M). The bulkier methionine group sterically hinders the binding of these reversible inhibitors. Also, the T790M mutation confers an increased affinity for ATP to bind with the receptor^6–10^.

As a result, second-generation irreversible inhibitors have been developed, including afatinib (**C**) and dacomitinib (**D**) (Figure 1). These inhibitors possess the aniline-quinazoline along with a reactive acrylamide moiety, which irreversibly (covalently) binds with C797S residue by undergoing a Michael addition reaction. Unfortunately, their poor selectivity between EGFR^T790M^ mutants and the wild-type (WT) EGFR causes dose-limiting major adverse effects, such as grade 3 diarrhoea, skin rashes, and many others^7–10^.

The search for compounds showing selective inhibition against drug-resistant mutant EGFR^T790M^ has led to the design and development of third generation EGFR inhibitors, such as AZD9291 (Osimertinib) (**E**), with the 2-arylamine pyrimidine scaffold exhibited great selective inhibitory effects on EGFR^L858R/T790M^ over wild-type EGFR (Figure 1). They bind irreversibly (covalently) with C797. However, resistance to third-generation inhibitors has also been observed as a result of new mutations, specifically the point mutation C797, which is one of the key residues for drug binding^7–10^.

The relation between inflammation and cancer progression has been recently supported by many studies. Multiple inflammatory mediators are expressed in the majority of cancer cells and contribute to different stages of tumour growth, starting from tumour initiation till metastasis^11^^,^^12^. Cyclooxygenases (COX-1/2) and lipoxygenases (5/12/15-LOX) are among the major enzymes involved in the formation of eicosanoids inflammatory mediators via the arachidonic acid (AA) cascade^11^.

The overexpression of COX-2 in various types of tumours including human colorectal cancer, pancreatic cancers, oesophageal, breast, prostate, lung carcinomas, and melanoma provided evidence for COX-2 role in cancer progression and resistance to radiotherapy and chemotherapy. Moreover, COX-2 inhibitors displayed both chemo-preventive and anticancer effects by synergistically or additively acting with anticancer agents^2^^,^^11^^,^^12^. Interestingly, non-steroidal anti-inflammatory drugs (NSAIDs) with greater COX-2 inhibition selectivity could inhibit angiogenesis and restore normal apoptosis in many types of cancer cells^2^^,^^5^^,^^13^.

Many studies have revealed the role of 15-Lipoxygenase (15-LOX), the other arm of the AA cascade, and its metabolites, in cancer progression and metastasis. The selective 15-LOX inhibitor, PD146176, showed antiproliferative activity against prostatic cancer cells^11^^,^^14^^,^^15^.

Dual COX-2/LOX inhibitors, targeting eicosanoids for anticancer effects, showed superior anticancer activities to their single pathway counterpart inhibitors. This could be because inhibiting only the COX-2 arm increases AA availability and shifts the AA metabolic machinery towards excessive production of downstream LOX inflammatory mediators^11^^,^^16^. Furthermore, the combination therapy of licofelone (a dual COX/LOX inhibitor) with gefitinib (EGFR TKI) showed significant tumour growth inhibition in pancreatic cancer (PC) at a dose lower than doses of the individual agents^4^^,^^17^^,^^18^. Accordingly, we herein describe our efforts to design and synthesise novel quinazolinones as multi-target directed ligands (MTDLs) that triple target EGFR^L858R/T790M^, COX-2, and 15-LOX simultaneously. *In vitro* EGFR, COX-1/COX-2, and 15-LOX inhibitory activities of the newly synthesised derivatives were evaluated, as well as their anticancer and anti-inflammatory activities. Moreover, *in silico* studies were performed for those compounds using docking analysis to elucidate a postulated model for their binding with EGFR^L858R/T790M^, COX-2, and 15-LOX at the molecular level.

### The rationale for designing multi-target directed ligands (MTDLs)

Designing multi-target directed ligands (MTDLs) that triple target EGFR^L858R/T790M^, COX-2, and 15-LOX is very challenging. One of the most critical challenges in designing EGFR TKIs has been the development of secondary mutations. Therefore, there is a critical need to develop reversible non-covalent EGFR inhibitors that are more selective against the double mutant EGFR^L858R/T790M^^6^.

The ATP binding site of EGFR consists of five distinct regions, adenine binding site/hinge region, hydrophilic ribose binding pocket, phosphate binding pocket, back hydrophobic region, and front hydrophobic region^19^^,^^20^. Therefore, the majority of the ATP-mimic EGFR-TKIs share four common pharmacophoric features, including a hydrophobic head (that occupies the back hydrophobic region), a scaffold (primarily a nitrogen-containing heterocycle) capable of interacting *via* an H-bond with the adenine/hinge region, an NH spacer (hinge region), and a hydrophobic tail (that occupies the front hydrophobic region)^19–21^ (Figure 1).

**Figure 2.**
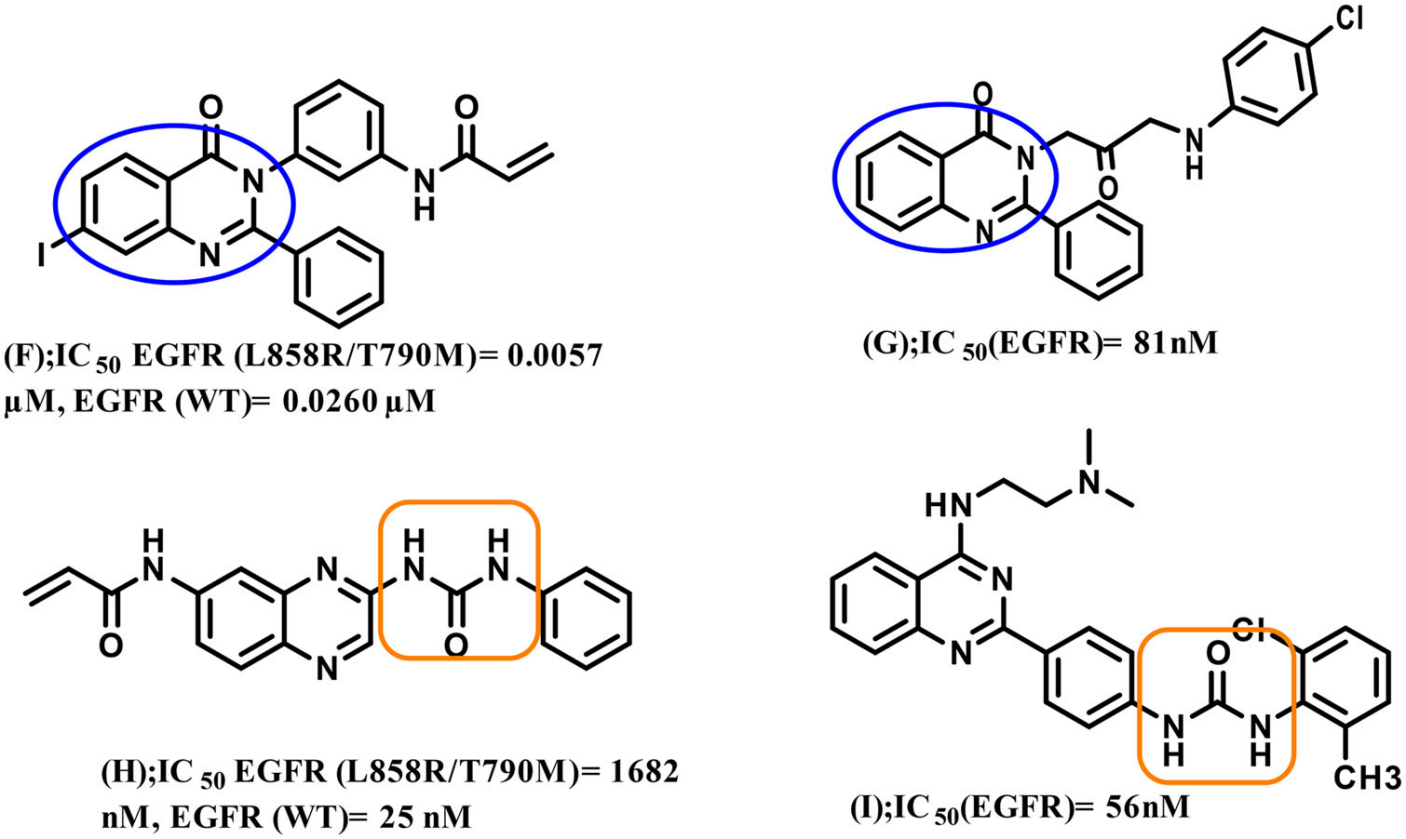
Reported 2,3‐disubstituted quinazolinones or urea-containing compounds with significant inhibitory activity against EGFR TK.

4-Amino-quinazoline represents an important class of first and second generations EGFR-TKIs as it fits into the ATP binding pocket, forming H-bond with the hinge region due to *N*-1 and *N*-3 atoms. The substitution of its *N*-1 and *N*-3 with carbon atoms decreases the inhibitory activity by 3700 and 200-fold, respectively^6^^,^^22^^,^^23^. Recently, some studies have validated 2,3‐disubstituted quinazolinones as promising scaffolds for EGFR inhibition and NSCLC management (compounds **F** and **G**)^7^^,^^24^, the addition of a lipophilic group at the 2‐position improving overall activity^23^. Other studies recommended urea functionality as a hydrogen bond domain (compounds **H** and **I**)^25^^,^^26^ (Figure 2).

The common pharmacophoric structural features of the previously reported dual COX-2/15-LOX inhibitors include three regions: nitrogen-containing bicyclic ring, a central five or six-membered nitrogen-containing heterocycle (azole or azine), and a hydrophobic terminal usually containing an aromatic ring. Replacement of the central azole or azine with an open ring analogue has also been reported as observed in compounds (**J–M**)^16^.

Herein, we attempted to design compounds that match the pharmacophoric features of both EGFR-TKIs and COX-2/15-LOX dual inhibitors by (I) using a quinazoline ring (a well-known scaffold of EGFR-TKIs that occupies the adenine/hinge region, and also presents a nitrogen-containing bicyclic ring in dual COX-2/15-LOX inhibitors pharmacophore), (II) incorporating urea functionality spacer (instead of NH spacer in EGFR-TKIs to trigger extra H-bond interactions, also represents open ring analogue of azole or azine in the dual COX-2/15-LOX inhibitor pharmacophore), and (III) attaching different substituted phenyl rings (as the hydrophobic head and the hydrophobic terminal in the pharmacophores of EGFR-TKIs and dual COX-2/15-LOX inhibitors, respectively).

Moreover, we removed the large hydrophobic tail of EGFR-TKIs, which had little effect on their efficacy^22^^,^^23^, making the molecules smaller and easier to shift to tolerate the bulkier methionine inside the T790M binding pocket. We avoided the acrylamide moiety (Michael acceptor) responsible for irreversible covalent bond formation with C797 at the ATP‐binding site of EGFR‐TK, which caused toxicity in second generation therapies, as well as the C797 mutation responsible for resistance in third generation therapies^7–10^^,^^23^. We also added an extra hydrophobic moiety at position 2 of the quinazoline ring in some compounds to investigate extra hydrophobic interactions with our three targets and the effect of achieving the famous V-shape characteristic of selective COX-2 inhibitors^27^.

## Materials and methods

### Chemistry

All reagents were commercially obtained with the highest percent of purity available, especially for synthesis, unless otherwise mentioned. ^1^H and ^13^C-NMR spectra were recorded on a Bruker Avance III spectrometer operating at 400 and 100 MHz, respectively. The NMR solvent used was DMSO-*d_6_* ((CD_3_)_2_SO). Chemical shifts are reported in parts per million (ppm) relative to the internal standard tetramethylsilane (Me_4_Si). Coupling constants (*J*-value) were calculated in hertz (*Hz*). All NMR characterisations were made by comparison with previous NMR spectra of the appropriate structure class and/or predictions from ChemDraw Ultra™. NMR analyses were performed at the Applied Nucleic Acids Research Centre & Chemistry, Faculty of Science, Zagazig University, Zagazig, Egypt or the Microanalytical Unit–FOPCU–NMR laboratory, Faculty of Pharmacy, Cairo University, Egypt.

Analytical thin layer chromatography (TLC) was carried out on precoated silica plates (ALUGRAM^®^ SIL G/UV_254_) and visualised with UV light (254 nm) and/or ninhydrin stain.

Melting points were determined using a Gallenkamp (London, UK) melting point apparatus and are uncorrected. Mass spectra were collected using a GC/MS Shimadzu Qp-2010 plus instrument (Shimadzu Corporation, Tokyo, Japan) at Micro Analytical Centre, Cairo University, Egypt.

Elemental analyses were performed using a Vario EL-III (Elementar) CHN analyser (Hanau, Germany) at Micro Analytical Centre, Cairo University, Egypt, or the Regional Centre for Mycology and Biotechnology, Al-Azhar University, Cairo, Egypt.

#### General method for the synthesis of N-phenylhydrazinecarboxamides (3a–c)

*N-(4-un/substitiutedphenyl)hydrazine carboxamides* (**3a**, **3b**, **3c**) were prepared according to previously reported procedures^28^^,^^29^. Trimethylamine (27.64 mmol) was added to a solution of the appropriate aniline **1a–c** (13.82 mmol) in dichloromethane (50 ml). The reaction mixture was cooled to 0 °C, followed by the addition of ethyl chloroformate (20.73 mmol) dropwise. Then the reaction mixture was slowly warmed to room temperature and stirred for 2 h. After reaction completion, water (25 ml) was added to quench excess ethyl chloroformate. The layers were separated, and the aqueous layer was extracted twice with dicholoromethane (15 ml). The combined organic layers were dried over anhydrous MgSO_4_, filtered, and evaporated under reduced pressure to obtain the respective crude carbamates **2a–c**.

A mixture of the appropriate carbamate **2a–c** (24.66 mmol) and hydrazine hydrate (73.98 mmol) in ethanol (20 ml) was stirred under reflux for 5 h. After completion of the reaction, the reaction mixture was cooled, and the formed crystals were filtered to obtain the corresponding carboxamides **3a–c**. The crystals were washed with water followed by petroleum ether. The carboxamides **3a–c** were pure enough to be used for the subsequent reactions.

##### *N*-phenylhydrazinecarboxamide (3a)

White microcrystals, 85% yield, M. p. 149–151 °C [Lit.^29^ M. p. 146–148 °C].

##### *N*-(4-methoxyphenyl)hydrazinecarboxamide (3b)

White microcrystals, 80% yield, M. p. 152–154 °C.

##### *N*-(4-fluorophenyl)hydrazinecarboxamide (3c)

White microcrystals, 79% yield, M. p. 178–180 °C [Lit.^29^ (M. p. 182–184 °C].

#### General method for the synthesis of 2-(2-(methylamino)benzoyl)-N-phenylhydrazine-1-carboxamide (5a) and N-(4-substitutedphenyl)-2-(2-(methylamino)benzoyl)hydrazine-1-carboxamide (5b, 5c)

A mixture of *N-methyl isatoic anhydride* (**4**, 0.82 g, 4.63 mmol) and hydrazine carboxamide (**3a**, **3b**, or **3c**, 4.63 mmol) in ethanol (20 ml) containing a catalytic amount of glacial acetic acid (5 drops) was refluxed for 4 h. After cooling, the formed crystals were isolated by filtration under vacuum to provide the required intermediates **5a–c** with a 78–85% yield and sufficient purity to be used in the subsequent step without further purification.

##### 2-(2-(Methylamino)benzoyl)-*N*-phenylhydrazine-1-carboxamide (5a)

Pale-yellow crystals, 85% yield, M. p. 205–207 °C. ^1^H NMR (400 MHz, DMSO-*d_6_*) *δ* 10.01 (s, 1H, NHCONHNH, exch.), 8.84 (s, 1H, NHCONHNH, exch.), 8.01 (s, 1H, NHCONHNH, exch.), 7.66 (d, *J* = 7.6 Hz, 1H, benzohydrazide-C_3_-H), 7.54 (s, 1H, NHCH_3_, exch.), 7.46 (d, *J* = 7.6 Hz, 2H, phenyl-C_2,6_-H), 7.30 (m, 3H, phenyl-C_3,4,5_-H), 6.95 (t, *J* = 7.9 Hz, 1H, benzohydrazide-C_5_-H), 6.67 (d, *J* = 8.0 Hz, 1H, benzohydrazide-C_6_-H), 6.58 (t, *J* = 7.5 Hz, 1H, benzohydrazide-C_4_-H), 2.79 (s, 3H, NHCH_3_).

##### *N*-(4-methoxyphenyl)-2-(2-(methylamino)benzoyl)hydrazine-1-carboxamide (5b)

Yellow crystals, 80% yield, M. P. 152–154 °C. ^1^H NMR (400 MHz, DMSO-*d_6_*) *δ* 9.98 (s, 1H, NHCONHNH, exch.), 8.65 (s,1H,NHCONHNH, exch.), 7.92 (s, 1H, NHCONHNH, exch.), 7.66 (d, *J* = 7.5 Hz, 1H, benzohydrazide-C_3_-H), 7.55 (s, 1H, NHCH_3_, exch.), 7.39–7.28 (m, 3H, benzohydrazide-C_5_-H and phenyl-C_2,6_-H), 6.84 (d, *J* = 9.1 Hz, 2H, phenyl-C_3,5_-H), 6.66 (d, *J* = 8.1 Hz, 1H, benzohydrazide-C_6_-H), 6.57 (t, *J* = 7.9 Hz, 1H, benzohydrazide-C_4_-H), 3.70 (s, 3H, OCH_3_), 2.78 (s, 3H, NHCH_3_).

##### *N*-(4-fluorophenyl)-2-(2-(methylamino)benzoyl)hydrazine-1-carboxamide (5c)

White fluffy crystals, 78% yield, M. p. 178–180 °C. ^1^H NMR (400 MHz, DMSO-*d_6_*) *δ* 10.01 (s, 1H, NHCONHNH, exch.), 8.88 (s, 1H, NHCONHNH, exch.), 8.04 (s, 1H, NHCONHNH, exch.), 7.66 (d, *J* = 7.7 Hz, 1H, benzohydrazide-C_3_-H), 7.57–7.43 (m, 3H, NHCH_3_, exch. + phenyl-C_2,6_-H), 7.41–7.29 (m, 1H, benzohydrazide-C_5_-H), 7.09 (t, *J* = 8.9 Hz, 2H, phenyl-C_3,5_-H), 6.66 (d, *J* = 8.4 Hz, 1H, benzohydrazide-C_6_-H), 6.58 (t, *J* = 8.0 Hz, 1H, benzohydrazide-C_4_-H), 2.78 (s, 3H, NHCH_3_).

#### General methods for the synthesis of compounds (6a-p)

##### a- Synthesis of compounds (6a, b, g, k, and l)

To a suspension of benzoylhydrazine-1-carboxamide (**5a**, **5b**, or **5c**, 2.81 mmol) in ethanol (25 ml), 37% formalin or acetaldehyde (4.215 mmol) and 5 drops glacial acetic acid were added. The reaction mixture was heated under reflux for 2 h and then concentrated to its half volume under vacuum evaporator. The mixture was cooled, and a precipitate was allowed to be formed. The obtained product was collected under vacuum filtration and crystallised from ethanol to produce the title compounds **6a**, **b**, **g**, **k**, and **l**.

##### b- Synthesis of compounds (6c–f, 6h–j, 6m–p)

A mixture of benzoylhydrazine-1-carboxamide (**5a**, **5b**, or **5c**, 2.81 mmol) and the appropriate aromatic aldehyde (2.81 mmol) in glacial acetic acid (20 ml) was heated under reflux for 4 h. The reaction mixture was concentrated to its half volume and then poured into cold water (50 ml). The separated solid was isolated by filtration, dried, and crystallised from ethanol to give the title compounds **6c–f, 6h–j, 6m–p**.

##### 1-(1-Methyl-4-oxo-1,4-dihydroquinazolin-3(2*H*)-yl)-3-phenylurea (6a)

White fluffy crystals, 91% yield, M. p. 240–241 °C. ^1^H NMR (400 MHz, DMSO-*d_6_*) *δ* 8.99 (s, 1H, NNHCONH, exch), 8.58 (s, 1H, NNHCONH, exch), 7.76 (d, *J* = 7.6 Hz, 1H, quinazolinone-C_5_-H), 7.47–7.43 (m, 3H, quinazolinone-C_7_-H, phenyl-C_2,6_-H), 7.28–7.24 (m, 2H, phenyl-C_3,5_-H), 6.97 (t, *J* = 7.5 Hz, 1H, quinazolinone-C_6_-H), 6.87 (m, 2H, quinazolinone-C_8_-H and phenyl-C_4_-H), 4.70 (s, 2H, quinazolinone-C_2_-H), 2.87 (s, 3H, NCH_3_).^13^C NMR (101 MHz, DMSO-*d_6_*) *δ* 163.29 (C), 154.73 (C), 149.58 (C), 139.40 (C), 134.01 (C), 128.70 (2 × Ar-CH), 128.25 (Ar-CH), 122.09 (Ar-CH), 118.43 (2 × Ar-CH), 118.22 (Ar-CH), 115.91 (Ar-CH), 112.64 (Ar-CH), 69.35 (CH_2_), 35.28 (NCH_3_). **MS**, m/z: 296.20 (M^+^). Analysis calcd. for C_16_H_16_N_4_O_2_: C, 64.85; H, 5.44; N, 18.91. Found: C, 64.60; H, 5.08; N, 18.58.

##### 1-(1,2-Dimethyl-4-oxo-1,4-dihydroquinazolin-3(2*H*)-yl)-3-phenylurea (6b)

Brown crystals, 87% yield, M. p. 161–163 °C. ^1^H NMR (400 MHz, DMSO-*d_6_*) *δ* 8.91 (s, 1H, NNHCONH, exch.), 8.61 (s, 1H, NNHCONH, exch.), 7.73 (dd, *J* = 7.7, 1.6 Hz, 1H, quinazolinone-C_5_-H), 7.53–7.39 (m, 3H, quinazolinone-C_7_-H and phenyl-C_2,6_-H), 7.34–7.22 (m, 2H, phenyl-C_3,5_-H), 6.97 (t, *J* = 7.4 Hz, 1H, quinazolinone-C_6_-H), 6.84–6.74 (m, 2H, quinazolinone-C_8_-H and phenyl-C_4_-H), 4.92 (q, *J* = 5.9 Hz, 1H, CHCH_3_), 2.89 (s, 3H, NCH_3_), 1.24 (d, *J* = 5.9 Hz, 3H, CHCH_3_). ^13^C NMR (101 MHz, DMSO-*d_6_*) *δ* 161.48 (C), 154.65 (C), 146.83 (C), 139.44 (C), 134.22 (C), 128.70 (2 × Ar-CH), 127.89 (Ar-CH), 122.12 (Ar-CH), 118.56 (2 × Ar-CH), 117.47 (Ar-CH), 115.06 (Ar-CH), 112.74 (Ar-CH), 75.71 (CHCH_3_), 34.74 (NCH_3_), 14.08 (CHCH_3_). **MS**, m/z: 310.10 (M^+^), 311.10 (M^+^+1). Analysis calcd. for C_17_H_18_N_4_O_2_: C, 65.79; H, 5.85; N, 18.05. Found: C, 65.55; H, 5.46; N, 18.06.

##### 1-(1-Methyl-4-oxo-2-phenyl-1,4-dihydroquinazolin-3(2*H*)-yl)-3-phenylurea (6c)

Yellow crystals, 89% yield, M. p. 180–181 °C. ^1^H NMR (400 MHz, DMSO-*d_6_*) *δ* 8.76 (s, 1H, NNHCONH, exch.), 8.34 (s, 1H, NNHCONH, exch.), 7.79 (dd, *J* = 7.7, 1.6 Hz, 1H, quinazolinone-C_5_-H), 7.49–7.22 (m, 10H, 2 × (phenyl-C_2,3,4,5,6_-H)), 6.97 (t, *J* = 7.4 Hz, 1H, quinazolinone-C_7_-H), 6.84 (t, *J* = 7.5 Hz, 1H, quinazolinone-C_6_-H), 6.69 (d, *J* = 8.2 Hz, 1H, quinazolinone-C_8_-H), 5.90 (s, 1H, quinazolinone-C_2_-H), 2.83 (s, 3H, NCH_3_). ^13^C NMR (101 MHz, DMSO-*d_6_*) *δ* 161.59 (C), 154.29 (C), 146.70 (C), 139.27 (C), 137.30 (C), 134.62 (C), 129.05 (2 × Ar-CH), 128.76 (d, *J* = 8.4 Hz, 4 × Ar-CH), 127.95 (Ar-CH), 126.50 (2 × Ar-CH), 118.18 (2 × Ar-CH), 117.65 (Ar-CH), 114.50 (Ar-CH), 112.25 (Ar-CH), 80.38 (quinazolinone-C_2_-H), 35.02 (NCH_3_). **MS**, m/z: 372.20 (M^+^), 374.20 (M^+^+2). Analysis calcd. for C_22_H_20_N_4_O_2_: C, 70.95; H, 5.41; N, 15.04. Found: C, 70.59; H, 5.09; N, 15.30.

##### 1-(2-(4-Fluorophenyl)-1-methyl-4-oxo-1,4-dihydroquinazolin-3(2*H*)-yl)-3-phenylurea (6d)

Off-white crystals, 86% yield, M. p. 230–232 °C. ^1^H NMR (400 MHz, DMSO-*d_6_*) *δ* 8.79 (s, 1H, NNHCONH, exch.), 8.39 (s, 1H, NNHCONH, exch.), 7.80 (d, *J* = 9.1 Hz, 1H, 1H, quinazolinone-C_5_-H), 7.40 (m, 5H, quinazolinone-C_7_-H, phenyl-C_2,6_-H and 4-fluorophenyl-C_2,6_-H), 7.24 (m, 4H, phenyl-C_3,5_-H and 4-fluorophenyl-C_3,5_-H), 6.98 (t, *J* = 7.3 Hz, 1H, phenyl-C_4_-H), 6.86 (t, *J* = 7.4 Hz, 1H, quinazolinone-C_6_-H), 6.71 (d, *J* = 8.3 Hz, 1H, quinazolinone-C_8_-H), 5.94 (s, 1H, quinazolinone-C_2_-H), 2.82 (s, 3H, NCH_3_). ^13^C NMR (101 MHz, DMSO-*d_6_*) *δ* 164.11 (C), 161.93 (C), 161.68 (C), 154.76 (C), 147.06 (C), 139.71 (C), 135.15 (C), 134.12 (C), 129.21 (2 × Ar-CH), 128.44 (2 × Ar-CH), 122.63 (Ar-CH), 118.71 (Ar-CH), 118.25 (Ar-CH), 116.10 (Ar-CH), 115.88 (2 × Ar-CH), 114.90 (2 × Ar-CH), 112.79 (Ar-CH), 80.11 (quinazolinone-C_2_-H quinazolinone-C_2_-H), 35.37 (NCH_3_). **MS**, m/z: 390.20 (M^+^). Analysis calcd. for C_22_H_19_FN_4_O_2_: C, 67.68; H, 4.91; N, 14.35. Found: C, 67.90; H, 4.66; N, 14.28.

##### 1-(1-Methyl-2-(4-nitrophenyl)-4-oxo-1,4-dihydroquinazolin-3(2*H*)-yl)-3-phenylurea (6e)

Yellow fluffy crystals, 79% yield, M. p. 241–243 °C. ^1^H NMR (400 MHz, DMSO-*d_6_*) *δ* 8.83 (s, 1H, NNHCONH, exch.), 8.52 (s, 1H, NNHCONH, exch.), 8.23 (d, *J* = 8.8 Hz, 2H, 4-nitrophenyl-C_3,5_-H), 7.80 (d, *J* = 7.7, 1H, quinazolinone-C_5_-H), 7.59 (d, *J* = 8.8 Hz, 2H, -nitrophenyl-C_2,6_-H), 7.52–7.38 (m, 3H, phenyl-C_3,4,5_-H), 7.33–7.21 (m, 2H, phenyl-C_2,6_-H), 6.97 (t, *J* = 7.4 Hz, 1H, quinazolinone-C_7_-H), 6.88 (t, *J* = 7.5 Hz, 1H, quinazolinone-C_6_-H), 6.73 (d, *J* = 8.2 Hz, 1H, quinazolinone-C_8_-H), 6.12 (s, 1H, quinazolinone-C_2_-H), 2.86 (s, 3H, NCH_3_). ^13^C NMR (101 MHz, DMSO-*d_6_*) *δ* 161.26 (C), 154.33 (C), 147.90 (C), 146.39 (C), 144.40 (C), 139.24 (C), 134.77 (C), 128.75 (2 × Ar-CH), 128.09 (2 × Ar-CH), 128.05 (Ar-CH),123.87 (2 × Ar-CH), 122.20 (Ar-CH), 118.36 (Ar-CH), 118.17 (2 × Ar-CH), 114.58 (Ar-CH), 112.60 (Ar-CH), 79.31 (quinazolinone-C_2_-H), 35.12 (NCH_3_). **MS**, m/z: 418.40 (M^+^+1). Analysis calcd. for C_22_H_19_N_5_O_4_: C, 63.30; H, 4.59; N, 16.78. Found: C, 63.40; H, 4.34; N, 17.05.

##### 1-(2-(4-Methoxyphenyl)-1-methyl-4-oxo-1,4-dihydroquinazolin-3(2*H*)-yl)-3-phenylurea (6f)

White fluffy crystals, 79% yield, M. p. 198–200 °C. ^1^H NMR (400 MHz, DMSO-*d_6_*) *δ* 8.75 (s, 1H, NNHCONH, exch.), 8.29 (s, 1H, NNHCONH, exch.), 7.78 (dd, *J* = 7.7, 1.6 Hz, 1H, quinazolinone-C_5_-H), 7.50–7.37 (m, 3H, phenyl-C_3,4,5_-H), 7.33–7.18 (m, 4H, 4-methoxyphenyl-C_2,6_-H and phenyl-C_2,6_-H), 7.02–6.79 (m, 4H, quinazolinone-C_7,6_-H and 4-methoxyphenyl-C_3,5_-H), 6.68 (d, *J* = 8.3 Hz, 1H, quinazolinone-C_8_-H), 5.83 (s, 1H, quinazolinone-C_2_-H), 3.72 (s, 3H, OCH_3_), 2.79 (s, 3H, NCH_3_). ^13^C NMR (101 MHz, DMSO-*d_6_*) *δ* 161.62 (C), 159.80 (d, *J* = 3.8 Hz, C), 154.26 (C), 146.75 (C), 139.29 (C), 134.57 (C), 129.35 (2 × Ar-CH), 128.80 (2 × Ar-CH), 127.92 (Ar-CH), 127.87 (Ar-CH), 122.11 (Ar-CH), 118.15 (2 × Ar-CH), 117.85 (d, *J* = 60.9 Hz, Ar-CH), 114.23 (d, *J* = 41.9 Hz, Ar-CH), 114.02 (2 × Ar-CH), 113.11 (d, *J* = 183.1 Hz, Ar-CH), 80.05 (quinazolinone-C_2_-H), 55.14 (OCH_3_), 34.86 (NCH_3_). **MS**, m/z: 402.20 (M^+^). Analysis calcd. for C_23_H_22_N_4_O_3_: C, 68.64; H, 5.51; N, 13.92. Found: C, 68.36; H, 5.19; N, 14.02.

##### 1-(4-Methoxyphenyl)-3-(1-methyl-4-oxo-1,4-dihydroquinazolin-3(2*H*)-yl)urea (6g)

White fluffy crystals, 87% yield, M. p. 219–221 °C. ^1^H NMR (400 MHz, DMSO-*d_6_*) *δ* 8.80 (s, 1H, NNHCONH, exch.), 8.50 (s, 1H, NNHCONH, exch.), 7.75 (d, *J* = 7.7 Hz, 1H, quinazolinone-C_5_-H), 7.49–7.40 (m, 1H, quinazolinone-C_7_-H), 7.35 (d, *J* = 9.1 Hz, 2H, phenyl-C_2,6_-H), 6.89–6.83 (m, 4H, phenyl-C_3,5_-H and quinazolinone-C_6,8_-H), 4.69 (s, 2H, CH_2_), 3.70 (s, 3H, OCH_3_), 2.88 (s, 3H, NCH_3_). ^13^C NMR (101 MHz, DMSO-*d_6_*) *δ* 163.34 (C), 154.95 (C), 154.62 (C), 149.59 (C), 134.01 (C), 132.42 (C), 128.26 (Ar-CH), 120.28 (2 × Ar-CH), 118.23 (Ar-CH), 115.98 (Ar-CH), 113.90 (2 × Ar-CH), 112.64 (Ar-CH), 69.43 (CH_2_), 55.16 (OCH_3_), 35.29 (NCH_3_). **MS**, m/z: 326.10 (M^+^). Analysis calcd. for C_17_H_18_N_4_O_3_: C, 62.57; H, 5.56; N, 17.17. Found: C, 62.62; H, 5.31; N, 16.81.

##### 1-(4-Methoxyphenyl)-3-(1-methyl-4-oxo-2-phenyl-1,4-dihydroquinazolin-3(2*H*)-yl)urea (6h)

Grey powder, 77% yield, M. p. 196–198 °C. ^1^H NMR (400 MHz, DMSO-*d_6_*) *δ* 8.60 (s, 1H, NNHCONH, exch), 8.26 (s, 1H, NNHCONH, exch), 7.79 (d, *J* = 7.6 Hz, 1H, quinazolinone-C_5_-H), 7.42–7.17 (m, 8H, quinazolinone-C_7_-H, 4-methoxyphenyl-C_2,6_-H and phenyl-C_2,3,4,5,6_-H), 6.86–6.81 (m, 3H, quinazolinone-C_6_-H and 4-methoxyphenyl-C_3,5_-H), 6.68 (d, *J* = 8.3 Hz, 1H, quinazolinone-C_8_-H), 5.89 (s, 1H, quinazolinone-C_2_-H), 3.70 (s, 3H, OCH_3_), 2.82 (s, 3H, NCH_3_). ^13^C NMR (101 MHz, DMSO-*d_6_*) *δ* 161.62 (C), 154.60 (C), 152.96 (C), 146.69 (C), 137.34 (C), 134.58 (C), 132.95 (C), 129.02 (Ar-CH), 128.70 (2 × Ar-CH), 127.93 (Ar-CH), 126.50 (Ar-CH), 119.92 (2 × Ar-CH), 117.62 (s), 114.55 (Ar-CH), 113.97 (2 × Ar-CH), 112.22 (Ar-CH), 80.42 (quinazolinone-C_2_-H), 55.15 (OCH_3_), 35.02 (NCH_3_). **MS**, m/z: 402.20 (M^+^), 403.20 (M^+^+1). Analysis calcd. for C_23_H_22_N_4_O_3_: C, 68.64; H, 5.51; N, 13.92. Found: C, 68.85; H, 5.67; N, 14.20.

##### 1-(4-Methoxyphenyl)-3-(1-methyl-2-(4-nitrophenyl)-4-oxo-1,4-dihydroquinazolin-3(2*H*)-yl)urea (6i)

Yellow powder, 83% yield, M. p. 217–219 °C. ^1^H NMR (400 MHz, DMSO-*d_6_*) *δ* 8.66 (s, 1H, NNHCONH, exch.), 8.44 (s, 1H, NNHCONH, exch.), 8.22 (d, *J* = 8.8 Hz, 2H, 4-nitrophenyl-C_3,5_-H), 7.80 (dd, *J* = 7.7, 1.6 Hz, 1H, quinazolinone-C_5_-H), 7.59 (d, *J* = 8.7 Hz, 2H, 4-nitrophenyl-C_2,6_-H), 7.45 (t, *J* = 7.8 Hz, 1H, quinazolinone-C_7_-H), 7.32 (d, *J* = 9.0 Hz, 2H, 4-methoxyphenyl-C_2,6_-H), 6.89–6.83 (m, 3H, quinazolinone-C_6_-H and 4-methoxyphenyl-C_3,5_-H), 6.72 (d, *J* = 8.3 Hz, 1H, quinazolinone-C_8_-H), 6.11 (s, 1H, quinazolinone-C_2_-H), 3.70 (s, 3H, OCH_3_), 2.86 (s, 3H, NCH_3_). ^13^C NMR (101 MHz, DMSO-*d_6_*) *δ* 161.32 (2 × C), 154.66 (C), 154.55 (C), 147.89 (C), 146.39 (C), 144.45 (C), 134.75 (C), 132.25 (Ar-CH), 128.08 (Ar-CH), 128.05 (2 × Ar-CH), 123.87 (2 × Ar-CH), 120.18 (2 × Ar-CH), 118.15 (Ar-CH), 114.63 (Ar-CH), 113.93 (2 × Ar-CH), 112.58 (Ar-CH), 79.36 (quinazolinone-C_2_-H), 55.16 (OCH_3_), 35.13 (NCH_3_). **MS**, m/z: 448.10 (M^+^+1). Analysis calcd. for C_23_H_21_N_5_O_5_: C, 61.74; H, 4.73; N, 15.65. Found: C, 62.04; H, 4.68; N, 15.71.

##### 1-(4-Methoxyphenyl)-3-(2-(4-methoxyphenyl)-1-methyl-4-oxo-1,4-dihydroquinazolin-3(2*H*)-yl)urea (6j)

White microcrystals, 86% yield, M. p. 205–207 °C. ^1^H NMR (400 MHz, DMSO-*d_6_*) *δ* 8.60 (s, 1H, NNHCONH, exch.), 8.21 (s, 1H, NNHCONH, exch.), 7.79 (d, *J* = 7.7 Hz, 1H, quinazolinone-C_5_-H), 7.43 (t, *J* = 8.5 Hz, 1H, quinazolinone-C_7_-H), 7.33 (d, *J* = 9.0 Hz, 2H, 4-methoxphenyl-C_2,6_-H), 7.22 (d, *J* = 8.7 Hz, 2H, 4-methoxyphenyl-C_3,5_-H), 6.94–6.79 (m, 5H, 4-methoxyphenyl-C_2,3,5,6_-H and quinazolinone-C_6_-H), 6.68 (d, *J* = 8.3 Hz, 1H, quinazolinone-C_8_-H), 5.84 (s, 1H, quinazolinone-C_2_-H), 3.71 (s, 6H, 2 × OCH_3_), 2.80 (s, 3H, NCH_3_). ^13^C NMR (101 MHz, DMSO-*d_6_*) *δ* 162.11 (2 × C), 160.22 (C), 155.05 (C), 154.93 (C), 147.20 (C), 135.00 (C), 132.78 (Ar-CH), 129.85 (2 × Ar-CH), 128.36 (Ar-CH), 128.33 (2 × Ar-CH), 120.38 (2 × Ar-CH), 117.97 (Ar-CH), 114.95 (Ar-CH), 114.46 (2 × Ar-CH), 114.43(Ar-CH), 112.63 (Ar-CH), 80.55 (quinazolinone-C_2_-H), 55.60 (2 × OCH_3_), 35.33 (NCH_3_). **MS**, m/z: 432.30 (M^+^). Analysis calcd. for C_24_H_24_N_4_O_4_: C, 66.65; H, 5.59; N, 12.96. Found: C, 66.66; H, 5.26; N, 12.99.

##### 1-(4-Fluorophenyl)-3-(1-methyl-4-oxo-1,4-dihydroquinazolin-3(2*H*)-yl)urea (6k)

White fluffy crystals, 88% yield, M.p. 246–248 °C. ^1^H NMR (400 MHz, DMSO-*d_6_*) *δ* 9.04 (s, 1H, NNHCONH, exch.), 8.61 (s, 1H, NNHCONH, exch.), 7.75 (dd, *J* = 7.7, 1.6 Hz, 1H, quinazolinone-C_5_-H), 7.49–7.43 (m, 3H, quinazolinone-C_7_-H and phenyl-C_2,6_-H), 7.10 (t, *J* = 8.9 Hz, 2H, phenyl-C_3,5_-H), 6.96–6.81 (m, 2H, quinazolinone-C_6,8_-H), 4.69 (s, 2H, CH_2_), 2.88 (s, 3H, NCH3). ^13^C NMR (101 MHz, DMSO-*d_6_*) *δ* 163.36 (C), 158.67 (C), 156.31 (C), 154.85 (C), 149.61 (C), 135.79 (C), 134.06 (C), 128.28 (Ar-CH), 120.34 (2 × Ar-CH), 118.26 (Ar-CH), 115.93 (Ar-CH), 115.32 (Ar-CH), 115.10 (Ar-CH), 112.67 (Ar-CH), 69.37 (CH_2_), 35.30 (NCH_3_). **MS**, m/z: 314.30 (M^+^), 315.30 (M^+^+1). Analysis calcd. for C_16_H_15_FN_4_O_2_: C, 61.14; H, 4.81; N, 17.83. Found: C, 60.86; H, 4.78; N, 17.99.

##### 1–(1,2-Dimethyl-4-oxo-1,4-dihydroquinazolin-3(2*H*)-yl)-3-(4-fluorophenyl)urea (6l)

White crystals, 86% yield, M. p. 187–190 °C. ^1^H NMR (400 MHz, DMSO-*d_6_*) *δ* 8.95 (s, 1H, NNHCONH, exch.), 8.64 (s, 1H, NNHCONH, exch.), 7.72 (dd, *J* = 7.7, 1.5 Hz, 1H, quinazolinone-C_5_-H), 7.58–7.38 (m, 3H, quinazolinone-C_7_-H and phenyl-C_2,6_-H), 7.10 (t, *J* = 8.9 Hz, 2H, phenyl-C_3,5_-H), 6.87–6.71 (m, 2H, quinazolinone-C_6,8_-H), 4.91 (q, *J* = 5.8 Hz, 1H, CHCH_3_), 2.88 (s, 3H, NCH_3_), 1.24 (d, *J* = 5.9 Hz, 3H, CHCH_3_). ^13^C NMR (101 MHz, DMSO-*d_6_*) *δ* 161.63 (C), 158.74 (C), 156.37 (C), 154.83 (C), 146.88 (C), 135.81 (C), 134.29 (C), 127.95 (Ar-CH), 120.50 (2 × Ar-CH), 117.52 (Ar-CH), 115.33 (2 × Ar-CH), 115.11 (Ar-CH), 112.79 (Ar-CH), 75.77 (CHCH_3_), 34.78 (NCH_3_), 14.10 (CHCH_3_). **MS**, m/z: 328.20 (M^+^), 329.20 (M^+^+1). Analysis calcd. for C_17_H_17_FN_4_O_2_: C, 62.19; H, 5.22; N, 17.06. Found: C, 62.33; H, 5.49; N, 17.32.

##### 1-(4-Fluorophenyl)-3-(1-methyl-4-oxo-2-phenyl-1,4-dihydroquinazolin-3(2*H*)-yl)urea (6m)

Pale-yellow crystals, 79% yield, M. p. 220–222 °C. ^1^H NMR (400 MHz, DMSO-*d_6_*) *δ* 8.81 (s, 1H NNHCONH, exch.), 8.38 (s, 1H NNHCONH, exch.), 7.78 (d, *J* = 7.7 Hz, 1H, quinazolinone-C_5_-H), 7.50–7.29 (m, 8H, quinazolinone-C_7_-H, 4-fluorophenyl-C_2,6_-H and pheyl-C_2,3,4,5,6_-H), 7.10 (t, *J* = 8.8 Hz, 2H, 4-fluorophenyl-C_3,5_-H), 6.84 (t, *J* = 7.5 Hz, 1H, quinazolinone-C_6_-H), 6.69 (d, *J* = 8.4 Hz, 1H, quinazolinone-C_8_-H), 5.89 (s, 1H, quinazolinone-C_2_-H), 2.82 (s, 3H, NCH_3_). ^13^C NMR (101 MHz, DMSO-*d_6_*) *δ* 161.58 (C), 158.62 (C), 156.26 (C), 154.39 (C), 146.70 (C), 137.26 (C), 135.64 (C), 134.59 (C), 129.02 (Ar-CH), 128.67 (2 × Ar-CH), 127.93 (Ar-CH), 126.52 (2 × Ar-CH), 119.94 (2 × Ar-CH), 117.63 (Ar-CH), 115.38 (2 × Ar-CH), 115.16 (Ar-CH), 112.24 (Ar-CH), 80.37 (quinazolinone-C_2_-H), 35.00 (NCH_3_). **MS**, m/z: 390.20 (M^+^). Analysis calcd. for C_22_H_19_FN_4_O_2_: C, 67.68; H, 4.91; N, 14.35. Found: C, 67.72; H, 4.71; N, 14.06.

##### 1-(4-Fluorophenyl)-3-(2–(4-fluorophenyl)-1-methyl-4-oxo-1,4-dihydroquinazolin-3(2*H*)-yl)urea (6n)

Off-white crystals, 83% yield, M. p. 214–216 °C. ^1^H NMR (400 MHz, DMSO-*d_6_*) *δ* 8.84 (s, 1H, NNHCONH, exch.), 8.44 (s, 1H, NNHCONH, exch.), 7.80 (d, *J* = 9.1 Hz, 1H, quinazolinone-C_5_-H), 7.50–7.39 (m, 3H, quinazolinone-C_7_-H and 4-fluorophenyl-C_2,6_-H), 7.39–7.27 (m, 2H, 4-fluorophenyl-C_2,6_-H), 7.15 (m, 4H, 2 × 4-fluorophenyl-C_3,5_-H), 6.86 (t, *J* = 7.4 Hz, 1H, quinazolinone-C_6_-H), 6.71 (d, *J* = 8.3 Hz, 1H, quinazolinone-C_8_-H), 5.93 (s, 1H, quinazolinone-C_2_-H), 2.81 (s, 3H, NCH_3_). ^13^C NMR (101 MHz, DMSO-*d_6_*) *δ* 164.11 (C), 161.99 (C), 161.67 (C), 159.14 (C), 156.77 (C), 154.90 (C), 147.08 (C), 136.02 (C), 135.18 (C), 134.04 (C), 129.27 (Ar-CH), 129.19 (Ar-CH), 128.45 (Ar-CH), 120.64 (Ar-CH), 120.56 (Ar-CH), 118.27 (Ar-CH), 116.06 (Ar-CH), 115.84 (2 × Ar-CH), 115.62 (Ar-CH), 114.89 (Ar-CH), 112.80 (Ar-CH), 80.15 (quinazolinone-C_2_-H), 35.38 (NCH_3_). **MS**, m/z: 408.20 (M^+^), 409.20 (M^+^+1). Analysis calcd. for C_22_H_18_F_2_N_4_O_2_: C, 64.70; H, 4.44; N, 13.72. Found: C, 65.01; H, 4.22; N, 14.01.

##### 1-(4-Fluorophenyl)-3–(1-methyl-2-(4-nitrophenyl)-4-oxo-1,4-dihydroquinazolin-3(2*H*)-yl)urea (6o)

Yellow powder, 78% yield, M. p. 250–252 °C. ^1^H NMR (400 MHz, DMSO-*d_6_*) *δ* 8.89 (s, 1H, NNHCONH, exch.), 8.58 (s, 1H, NNHCONH, exch.), 8.22 (d, *J* = 8.8 Hz, 2H, 4-nitrophenyl-C_3,5_-H), 7.80 (dd, *J* = 7.7, 1.6 Hz, 1H, quinazolinone-C_5_-H), 7.59 (d, *J* = 8.8 Hz, 2H, nitrophenyl-C_2,6_-H), 7.54–7.40 (m, 3H, quinazolinone-C_7_-H and 4-fluorophenyl-C_2,6_-H), 7.10 (t, *J* = 8.9 Hz, 2H, 4-fluorophenyl-C_3,5_-H), 6.88 (t, *J* = 7.5 Hz, 1H, quinazolinone-C_6_-H), 6.73 (d, *J* = 8.3 Hz, 1H, quinazolinone-C_8_-H), 6.11 (s, 1H, quinazolinone-C_2_-H), 2.86 (s, 3H, NCH_3_). ^13^C NMR (101 MHz, DMSO-*d_6_*) *δ* 161.28 (C), 158.68 (C), 156.31 (C), 154.46 (C), 147.89 (C), 146.40 (C), 144.39 (C), 135.62 (C), 135.60 (C), 134.76 (C), 128.08 (2 × Ar-CH), 123.84 (2 × Ar-CH), 120.24 (Ar-CH), 120.16 (Ar-CH), 118.16 (Ar-CH), 115.34 (2 × Ar-CH), 115.12 (Ar-CH), 114.87 (Ar-CH), 112.60 (Ar-CH), 79.32 (quinazolinone-C_2_-H), 35.11 (NCH_3_). **MS**, m/z: 436.30 (M^+^+1); 437.30 (M^+^+2). Analysis calcd. for C_22_H_18_FN_5_O_4_: C, 60.69; H, 4.17; N, 16.08. Found: C, 60.56; H, 3.97; N, 16.40.

##### 1-(4-Fluorophenyl)-3-(2–(4-methoxyphenyl)-1-methyl-4-oxo-1,4-dihydroquinazolin-3(2*H*)-yl)urea (6p)

Pale-yellow crystals, 85% yield, M. p. 224–226 °C. ^1^H NMR (400 MHz, DMSO-*d_6_*) *δ* 8.80 (s, 1H, NNHCONH, exch.), 8.32 (s, 1H, NNHCONH, exch.), 7.77 (dd, *J* = 7.6, 1.1 Hz, 1H, quinazolinone-C_5_-H), 7.54–7.35 (m, 3H, quinazolinone-C_7_-H and 4-fluorophenyl-C_2,6_-H), 7.21 (d, *J* = 8.6 Hz, 2H, 4-methoxyphenyl-C_2,6_-H), 7.10 (t, *J* = 8.9 Hz, 2H, 4-fluorophenyl-C_3,5_-H), 6.95–6.78 (m, 3H, quinazolinone-C_6_-H and 4-methoxyphenyl-C_3,5_-H), 6.68 (d, *J* = 8.3 Hz, 1H, quinazolinone-C_8_-H), 5.81 (s, 1H, quinazolinone-C_2_-H), 3.71 (s, 3H, OCH_3_), 2.78 (s, 3H, NCH_3_). ^13^C NMR (101 MHz, DMSO-*d_6_*) *δ* 161.58 (C), 159.75 (C), 158.61 (C), 156.24 (C), 154.36 (C), 146.75 (C), 135.66 (C), 134.54 (C), 129.32 (Ar-CH), 127.89 (2 × Ar-CH), 119.98 (Ar-CH), 119.91 (Ar-CH), 117.52 (Ar-CH), 115.37 (Ar-CH), 115.15 (Ar-CH), 114.46 (Ar-CH), 113.98 (2 × Ar-CH), 112.19 (Ar-CH), 80.05 (quinazolinone-C_2_-H), 55.12 (OCH_3_), 34.85 (NCH_3_). **MS**, m/z: 420.30 (M^+^). Analysis calcd. for C_23_H_21_FN_4_O_3_: C, 65.71; H, 5.03; N, 13.33. Found: C, 66.00; H, 4.85; N, 13.20.

### Biological activity

#### In vitro *COX-1 and COX-2 inhibitory assay*

All final synthesised compounds (**6a–p**) were tested with an *in vitro* COX-1/COX-2 inhibition assay kit (Cayman, Ann Arbour, MI, USA) using ovine COX-1 or human recombinant COX-2 and compared to celecoxib, indomethacin, and diclofenac sodium as reference drugs. The half-maximal inhibitory concentration (IC_50_, μM) was determined, and the COX-2 selectivity index (SI) was calculated as IC_50_ (COX-1)/IC_50_ (COX-2).

#### In vitro *15-LOX inhibition assay*

Evaluation of 15-LOX inhibitory activities of all the synthesised compounds **6a–p** was carried out using soybean 15-LOX inhibitor screening assay (Cayman, Ann Arbour, MI) and nordihydroguaiaretic acid (NDGA) as reference according to the manufacturer’s instructions.

#### In vitro *EGFR inhibition activity*

We performed *in vitro* EGFR inhibition assay for all our newly synthesised compounds (**6a–p**) against both wild-type EGFR and the double mutant EGFR^L858R/T790M^ purchased from AssayQuant Technologies, Inc. (Marlboro, MA, USA) as per the manufacturer’s instructions. Osimertinib and afatinib were purchased from MedChemExpress LLC (Monmouth Junction, NJ, USA) and used as references.

#### Effects on NO, ROS, and cytokines production in LPS-activated RAW 264.7 macrophage cells

TNF-α and IL-6 production were detected in RAW 264.7 macrophage cell culture supernatants following the induction of inflammation by LPS. Moreover, the probes of oxidative species 2,7-dichlorofluorescein diacetate (DCFH-DA) (Molecular Probes) and NO, 4-amino-5-methylamino-2,7-difluorofluorescein diacetate (DAF-FM diacetate) (Molecular Probes) were used to investigate the antioxidant and NO production inhibitory potential of the test compounds. Briefly, RAW 264.7 cells were cultured in black 96 well plates (200 000 cells/mL, 100 µl/well) for 24 h followed by the incubation with the individual test compounds or the reference drugs at different concentrations (12.5, 25, 50, and 100 µM) for 2 h at 37 °C. LPS was then added at a final concentration of 1 µg/mL for an additional 18 h. The cell culture supernatant was collected from cells treated with the different compounds at 25 µM to measure TNF-α and IL-6 levels (1:10 dilution) using DuoSet ELISA kits (R&D Systems, Minneapolis, MN) following the manufacturer’s instructions. Then, the plates were washed to perform ROS and NO detection assays as detailed in our previous studies^16^^,^^27^.

#### Measurement of anticancer activity against a panel of 60 cell lines

Screening of the anticancer activity of the newly synthesised compounds (**6a**, **6c**, **6d**, **6e**, **6j**, **6l**, **6m**, **6n**, **6o**, and **6p**) was performed by the US National Cancer Institute (NCI) using 60 different human tumour cell lines in accordance with standard procedures formerly reported^30^.

#### Cell viability assay against normal cell line

To evaluate the possible non-selective cytotoxic effect of the investigated compounds (**6a**, **6c**, **6d**, **6e**, **6j**, **6l**, **6m**, **6n**, **6o**, and **6p**) on normal cells, their effect on RAW 264.7 cells viability was assessed at different concentrations (12.5, 25, 50, and 100 µM). Cell viability was measured using 3-(4,5-dimethylthiazol-2-yl)-5-(3-carboxymethoxyphenyl)-2-(4 sulfophenyl)-2H-tetrazolium (MTS) assay (Promega, Madison, Wisconsin) according to the manufacturer’s instructions.

### Molecular modelling

The X-ray crystal structures of COX-1 (PDB code: 1EQG/2.61 Å), COX-2 (PDB code: 1CX2/3.00 Å), 15-LOX (PDB code: 4NRE/2.63 Å), EGFR^WT^ (PDB code: 1M17/2.60 Å), and EGFR^T790M/L858R^ (PDB code: 5EDQ/2.80 Å) were retrieved from the Protein Data Bank (http://www.rcsb.org)^31^. The molecular docking studies were implemented using the Molecular Operating Environment; MOE 2019.0102 (Chemical Computing Group, Montreal, CA)^32^. The crystal structures were individually prepared using the quick preparation protocol through the Amber10: EHT forcefield. The compounds were drawn using the Chemdraw^®^ program, then transferred to the MOE as smiles format. The compound energy was minimised with a root mean square (RMS) gradient of 0.0001 kcal/mol. The co-crystallised ligands were re-docked into the active site for validation of the docking results by measuring the root mean square deviations (RMSD). The docking protocol is a triangle matcher, using London dG as the initial scoring approach and GBVI/WSA dG as the final scoring approach. The docking energy scores (S; Kcal/mol) and visual inspection of 2D and 3D planes of the ligand–enzyme interactions were used for the analysis of the docking results. A flexible alignment study was performed on compound **6e** and the co-crystallised substrate mimic (C8E) of 15-LOX, using MOE 2019.0102. The obtained conformations were evaluated according to the score of the configuration alignment (S; Kcal/mol).

## Results and discussion

### Chemistry

**Scheme 1.**
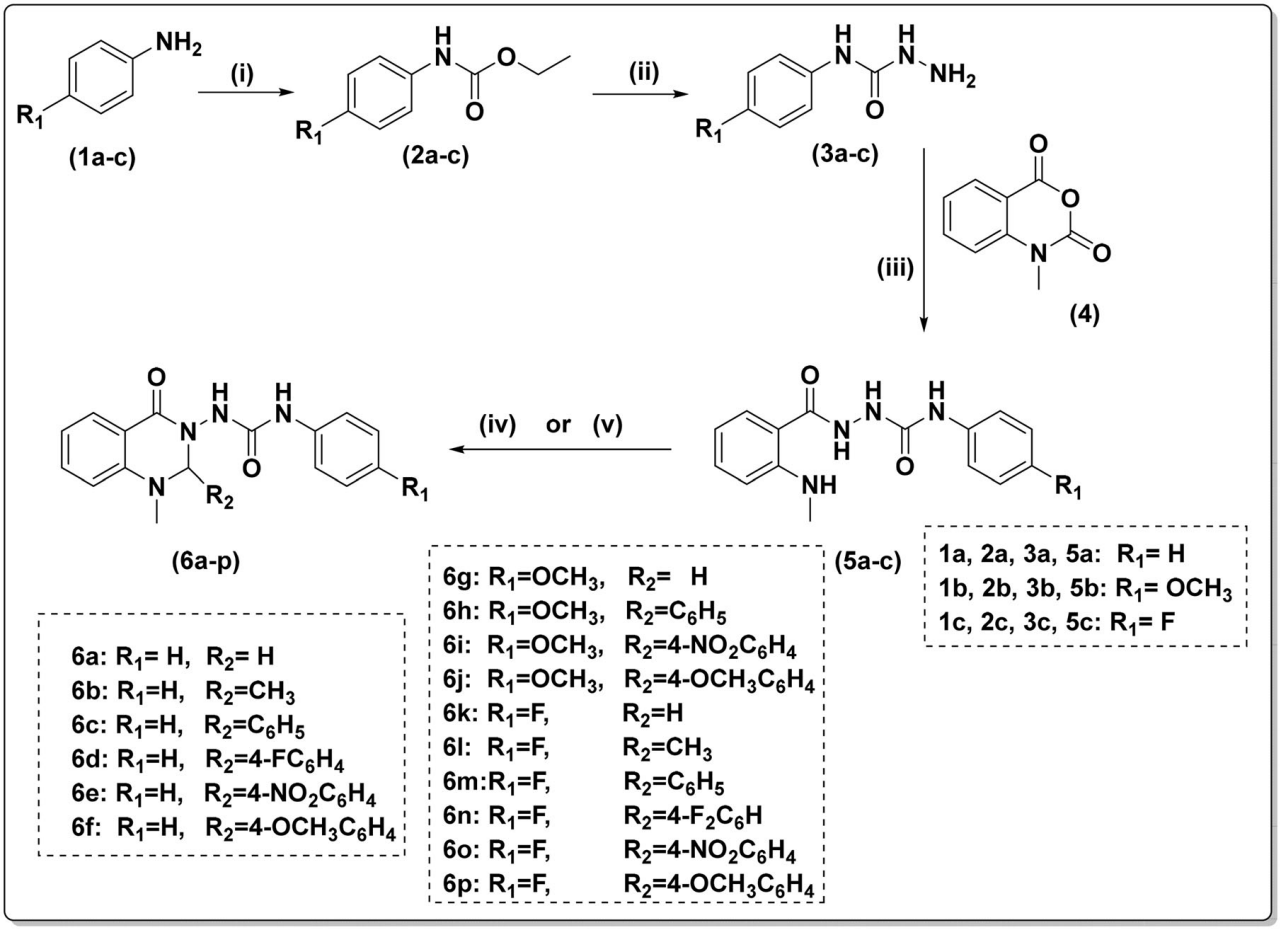
Reagents and conditions: (i) ethyl chloroformate/triethylamine/dichloromethane/0 °C—rt., 2h (ii) hydrazine hydrate 98%, ethanol, reflux, 5h (iii) ethanol/glacial acetic acid, reflux, 4 h; (iv) 37% formaline or acetaldehyde, ethanol/glacial acetic acid, reflux, 2 h; and (v) appropriate aromatic aldehyde, glacial acetic acid, reflux, 4 h.

The synthesis of compounds (**6a–p**) was carried out in four steps as illustrated in Scheme 1. First, appropriate anilines (**1a–c**) were reacted with ethyl chloroformate in dichloromethane and trimethylamine to yield the corresponding carbamates (**2a–c**). Carboxamides (**3a–c**) were then synthesised by hydrazinolysis of the ethyl ester of the carbamates (**2a–c**).

To obtain the urea linker, *N*-methyl isatoic anhydride (**4**) was reacted with the appropriate carboxamides (**3a–c**) in refluxing ethanol with a catalytic amount of glacial acetic acid, yielding key intermediates **5a–c**. The compounds **5a–c** were identified as having four NH groups in ^1^H-NMR revealed at *δ* 10.01–7.54 ppm.

Finally, cyclization of the key intermediate compounds (**5a–c**) to the target quinazolinones (**6a–p**) was achieved using different aldehydes. ^1^H-NMR and ^13^C-NMR spectroscopy, mass spectrometry, and elemental analysis were used to confirm the final compounds’ structures. In ^1^H-NMR, singlet signals of both NHCONHNH at *δ* 10.01–9.98 ppm and NHCH_3_ at 7.57–5.54 ppm disappeared. The NCHN signal of quinazolinone-C2-H appeared at 6.11–4.69 ppm. Moreover, ^13^C-NMR spectra revealed the appearance of NCN signal of quinazolinone-C2-H at 80.42–69.35 ppm.

### Biological activity

#### COX-1 and COX-2 inhibitory activities

The new quinazolines **6i** and **6n** had more potent COX-2 inhibitory profiles (IC_50_ = 0.47 and 0.65 µM) than the two reference drugs diclofenac sodium and indomethacin (IC_50_ = 0.76 and 1.57 µM, respectively); compounds **6e** and **6l** had greater COX-2 inhibitory activity than indomethacin, with IC_50_ values 0.83 and 1.49 µM, respectively, but were less potent than the reference drug celecoxib (IC_50_ = 0.03 µM). On the other hand, compounds **6a**, **6e**, **6d**, **6f**, **6g**, **6i**, **6m**, and **6n** showed moderate COX-1 inhibitory activity (IC_50_ = 3.05–12.46 µM) in comparison to the references celecoxib and diclofenac sodium (IC_50_ = 7.03 and 4.11 µM, respectively). In terms of COX-2 selectivity, compounds **6e**, **6h**, **6i**, **6k**, **6o**, and **6n** were 2–5 times more selective than diclofenac sodium (the reference drug) towards COX-2. The selectivity of the new compounds was significantly lower than that of celecoxib, which could be considered an advantage in avoiding the cardiovascular side effects of highly selective COX-2 inhibitors (Table 1).

**Table 1.** *In vitro* COX-1, COX-2, COX-2 selectivity index, and 15-LOX inhibitory activities of the final synthesised compounds **(6a–p)** and references.

Compound	^a^COX-1 IC_50_(µM)	^a^COX-2 IC_50_(µM)	^b^COX-2 SI	^a^15-LOX IC_50_(µM)
**6a**	12.46 ± 1.36^#@^	68.32 ± 1.89^*#@^	0.18	1.48 ± 0.27
**6b**	33.63 ± 3.17^*#@^	>100	–	2.32 ± 0.31
**6c**	1.00 ± 1.78	3.41 ± 1.83^*#^	0.29	1.36 ± 0.19
**6d**	4.56 ± 1.33	2.07 ± 1.39	2.20	1.96 ± 0.23
**6e**	9.53 ± 1.21^@^	0.83 ± 1.37	11.48	1.76 ± 0.27
**6f**	3.60 ± 1.57	2.13 ± 1.37	1.69	2.13 ± 0.26
**6g**	3.05 ± 3.89	3.92 ± 1.44^*#@^	0.78	1.61 ± 0.23
**6h**	47.54 ± 4.99^*#@^	2.81 ± 1.20*	16.92	2.03 ± 0.37
**6i**	7.63 ± 2.07^#^	0.47 ± 1.12	16.23	2.07 ± 0.27
**6j**	26.93 ± 3.03^*#@^	5.04 ± 1.56^*#@^	5.34	1.95 ± 0.35
**6k**	57.66 ± 2.41^*#@^	5.13 ± 1.37^*#@^	11.24	2.08 ± 0.33
**6l**	0.07 ± 2.01	1.49 ± 1.11	0.05	1.58 ± 0.28
**6m**	12.43 ± 2.74^#@^	3.16 ± 1.22^*#^	3.93	2.23 ± 0.36
**6n**	10.48 ± 1.48^@^	0.65 ± 1.30	16.12	2.00 ± 0.26
**6o**	>100	10.09 ± 1.01^*#@^	28.85	1.42 ± 0.23
**6p**	88.5 ± 3.01^*#@^	9.28 ± 1.04^*#@^	9.54	3.87 ± 2.10
Celecoxib	7.03 ± 1.40	0.03 ± 1.26	234.33	—
Indomethacin	0.64 ± 1.17	1.57 ± 1.21	0.41	—
Diclofenac	4.11 ± 1.47	0.76 ± 1.29	5.41	—
NDGA	—	—	—	1.33 ± 0.24

NDGA: nordihydroguaiaretic acid, Positive control inhibitor for LOX.

*p < 0.05 *vs.* celecoxib.

^#^*p* < 0.05 *vs.* diclofenac.

^@^*p* < 0.05 *vs.* indomethacin.

^a^IC_50_ concentration in (µM) for the different compounds.

^b^Selectivity index = (COX-1 IC_50_/COX-2 IC_50_). Values are expressed as mean ± SEM (*n* = 3 replicates).

In general, the preference of substitution of phenyl urea ring arranged as F > OCH_3_ > unsubstituted considering COX-2 selectivity. It is also notable that quinazolines with *p*-nitro phenyl substitution at 2-position (**6e**, **6i**, **6o**) showed superior COX-2 selectivity than their counterparts carrying the same phenyl urea derivative (Table 1).

#### In vitro *15-LOX inhibition activity*

Nearly all the new quinazolines **6a–o** revealed potent inhibitory activities against 15-LOX (IC_50_ = 1.36–2.32 µM) that showed comparable potency to that of the reference NDGA (IC_50_ = 1.33 µM) except for compound **6p** that showed lower potency (IC_50_ = 3.87 µM) (Table 1).

Among the unsubstituted phenyl urea derivatives (**6a–f**), compounds **6a**, **6c**, and **6e** carrying no substitution, phenyl and *p-*nitrophenyl at quinazoline C2, respectively, showed the most potent 15-LOX inhibitory activities (IC_50_ = 1.48, 1.36, and 1.76 µM). For the *p*-methoxyphenyl urea derivatives (**6g–j**), compound **6g** with 2-unsubstituted quinazoline was the most potent one (IC_50_ = 1.61 µM). Among the *p*-fluorophenyl urea derivatives (**6k–p**), compounds **6l** and **6o** carrying methyl and *p-*nitrophenyl at quinazoline C2 showed superior 15-LOX inhibitory potencies (IC_50_ = 1.58 and 1.42 µM).

#### In vitro *EGFR inhibition activity*

We performed *in vitro* EGFR inhibition assay for all our newly synthesised compounds (**6a–p**) against both wild-type EGFR and the double mutant EGFR^L858R/T790M^ using osimertinib and afatinib as references. Compounds **6a**, **6c**, **6d**, **6e**, **6g**, **6j**, and **6p** showed micromolar anti-EGFR activity that was less potent than the used references but was more selective against the double mutant EGFR^L858R/T790M^ (SI = >217–8.9) than the two reference drugs osimertinib and afatinib (SI = 8.4 and 0.11, respectively).

Moreover, compounds **6h**, **6i**, **6l**, **6m**, **6n**, and **6o** were more selective against the double mutant EGFR^L858R/T790M^ (SI = >7.4–1.5) than afatinib (SI = 0.11). Compounds **6b**, **6f,** and **6k** did not show any inhibitory activity against both EGFR types.

Except for compounds **6e** and **6o**, which had IC_50_ values of 48.69 and 52.44 µM, respectively, none of our compounds showed significant activity against EGFR^WT^. Compound 6a exhibited the greatest EGFR^L858R/T790M^ inhibitory activity, with IC_50_ values 0.46 µM and no significant activity against EGFR^WT^.

Among the unsubstituted phenyl urea derivatives (**6a–f**), compounds **6a**, **6d**, and **6e** carrying unsubstituted, *p*-fluorophenyl, and *p-*nitrophenyl at quinazoline C2, respectively, showed the best EGFR^L858R/T790M^ inhibitory activities (IC_50_ = 0.46, 5.57, and 5.48 µM). Compounds **6j** with *p*-methoxy at quinazoline C2 were the most potent of the *p*-methoxyphenyl urea derivatives (**6g–j**) (IC_50_ = 4.03 µM). Among the *p*-fluorophenyl urea derivatives (**6k–p**), compounds **6m** and **6p** carrying *p*-fluorophenyl and *p-*methoxyophenyl at quinazoline C2 showed superior EGFR^L858R/T790M^ inhibitory potencies (IC_50_ = 4.07 and 5.41 µM) (Table 2).

**Table 2.** *In vitro* inhibitory activities of the final synthesised compounds (**6a–p**) and references against wild-type EGFR and the double mutant EGFR^L858R/T790M^.

Compound	EGFR^WT^ IC_50_ (µM)	EGFR^L858R/T790M^ IC_50_ (µM)	Selectivity^a^ (SI)
**6a**	>100	0.46 ± 1.11	>217
**6b**	>100	>100	–
**6c**	>100	7.93 ± 1.11	>12.6
**6d**	>100	5.57 ± 1.78	>17.9
**6e**	48.69 ± 3.47	5.48 ± 1.33	8.9
**6f**	>100	>100	–
**6g**	>100	8.14 ± 1.07	>12.3
**6h**	>100	23.69 ± 3.54	>4.2
**6i**	>100	51.45 ± 4.58	>1.9
**6j**	>100	4.03 ± 1.43	>24.8
**6k**	>100	>100	–
**6l**	>100	17.04 ± 1.40	>5.8
**6m**	>100	4.07 ± 3.70	>7.4
**6n**	>100	13.52 ± 2.22	>7.4
**6o**	52.44 ± 1.25	36.05 ± 2.58	1.5
**6p**	>100	5.41 ± 3.21	>18.5
Osimertinib (nM)	66.04 ± 2.33	7.83 ± 1.35	8.4
Afatinib (nM)	3.70 ± 1.47	33.40 ± 1.76	0.11

^a^Selectivity ratios were calculated using the ratio of the IC_50_ values (EGFR^WT^/EGFR^L858R/T790M^).

Despite being less potent than the references, our compounds demonstrated greater selectivity against EGFR^L858R/T790M^ than the EGFR^WT^. Moreover, compound **6o** has a selectivity profile comparable to osimertinib and nearly 80-fold greater selectivity than afatinib. Interestingly, **6o** had the strongest anti-inflammatory effect against cytokine production, as well as a strong antioxidant and NO release inhibitory effects.

#### Effects on NO and ROS production in LPS-activated RAW 264.7 macrophage cells

LPS-activated RAW 264.7 macrophage cells are a popular *in vitro* model for studying various inflammatory responses and screening the mechanism of action of new anti-inflammatory candidates. The bacterial toxin LPS causes a strong inflammatory response in RAW 264.7 cells, resulting in increased production of several inflammatory mediators, including COX-2^33^, and nitric oxide (NO) via the induction of the inducible isoform of nitric oxide synthase^34^. Oxidative stress is the imbalance between oxidants and antioxidants, and occurs as a result of excessive generation of reactive oxygen species (ROS) leading to cellular injury^35^. ROS plays an important role in the inflammatory response, including LPS-mediated inflammation, and can stimulate the production of a wide range of inflammatory cytokines^36^. Moreover, increased ROS production is involved in the pathophysiology of multiple pathological conditions, including cancer^37^. As a result, targeting ROS with candidates with anti-oxidant potential is a successful strategy for treating both inflammation and cancer, as evidenced by numerous *in vivo* and *in vitro* studies^36^^,^^38^^,^^39^. Compounds **6a–p** were evaluated for their ability to inhibit NO and ROS production in LPS-activated RAW 264.7 macrophage cells.

All the tested compounds **6a–p** inhibited the production of the inflammatory mediator NO (IC_50_ = 0.97–13.54 µM) significantly more potent than the three reference drugs celecoxib, diclofenac, and indomethacin (IC_50_ = 14.39, 24.08, and 46.45 µM, respectively) with the exception of compound **6n** that showed a comparable potency to celecoxib (IC50 = 19.85 µM) and compound **6e** that did not show any significant NO inhibitory activity. The best compounds were **6a**, **6b**, **6c**, **6g**, and **6o** (IC_50_ = 0.97–1.97 µM), which showed ∼7–14-fold better IC_50_ values than that of celecoxib (IC_50_ = 14.39 µM) and 12–24-fold better IC_50_ values than that of diclofenac (IC_50_ = 24.08 µM) (Table 3).

**Table 3.** *In vitro* inhibitory activities of all final synthesised compounds (**6a–p**) and references on LPS-induced ROS, NO, TNF-α, and IL-6 production in RAW 264.7 cells.

Compound	ROS IC_50_ (µM)	NO IC_50_ (µM)	IL-6 concentration (pg/mL)	TNF-α concentration (pg/mL)
**6a**	35.71 ± 1.08	0.97 ± 1.67	173.7 ± 4.77^#^	1157.00 ± 78.71*
**6b**	55.53 ± 1.00	1.57 ± 1.29	512.1 ± 46.13*	779.60 ± 7.41^#^
**6c**	48.10 ± 1.07	1.54 ± 1.02	468.4 ± 87.46*	955.00 ± 4.66^*#^
**6d**	36.96 ± 1.00	4.12 ± 0.65	358.7 ± 12.85*	1222.00 ± 12.70*
**6e**	>100	>100	198.8 ± 6.08^#^	1929.00 ± 53.88*
**6f**	63.64 ± 1.30	4.05 ± 0.35	207.3 ± 4.51^#^	1239.00 ± 20.50*
**6g**	>100	1.24 ± 0.56	363.2 ± 12.62*	1292.00 ± 16.72*
**6h**	41.18 ± 1.00	2.73 ± 0.32	328.9 ± 1.31*	720.60 ± 4.90^*#^
**6i**	5.90 ± 1.84	7.89 ± 1.02	84.60 ± 2.67^#^	1821.00 ± 10.40*
**6j**	24.09 ± 1.08	13.54 ± 0.29	164.6 ± 5.09^#^	757.30 ± 2.50^*#^
**6k**	5.04 ± 1.43	3.10 ± 0.86	88.53 ± 0.87^#^	2024.00 ± 26.30*
**6l**	37.94 ± 1.00	5.24 ± 0.50	536.8 ± 3.55*	1250.00 ± 5.82*
**6m**	57.20 ± 1.00	10.30 ± 0.27	290.7 ± 5.5*	935.60 ± 3.85^*#^
**6n**	39.43 ± 2.25	19.85 ± 0.71	535.3 ± 15.25*	1981.00 ± 209.9*
**6o**	23.40 ± 1.13	1.97 ± 1.45	37.23 ± 1.19^#^	39.37 ± 1.21
**6p**	38.64 ± 1.33	10.84 ± 0.05	237.2 ± 3.19^*#^	1549.00 ± 20.25*
Celecoxib	7.57 ± 1.32	14.39 ± 0.08	176.4 ± 70.48^#^	383.3 ± 27.8^#^
Diclofenac	43.78 ± 1.04	24.08 ± 0.15	342.90 ± 40.81*	666.60 ± 11.71^#^
Indomethacin	79.36 ± 1.14	46.45 ± 1.07	ND	ND

NO: nitric oxide; ROS: reactive oxygen species; TNF-α: tumour necrosis factor-α; IL-6: interleukin-6; ND: not done.

IC_50 in_ (µM) concentration as expressed as mean ± SEM, for three replicates.

IL-6 and TNF-α levels were detected in cells treated with 25 µM of the tested compounds.

*p < 0.05 *vs.* celecoxib.

^#^*p* < 0.05 *vs.* diclofenac.

Except for nitro-containing compounds, unsubstituted phenyl urea derivatives (**6a–f**) outperform their corresponding counterparts with *p*-methoxyphenyl urea derivatives (**6g–j**) or *p*-fluorophenyl urea derivatives (**6k–p**).

Regarding ROS production, the majority of the tested compounds exhibited potent inhibitory activity, comparable to the two references diclofenac and indomethacin. Compounds **6i** and **6k** were the most effective at lowering ROS levels, with IC_50_ values of 5.90 and 5.04 µM, respectively (more potent than that of celecoxib, 7.57 µM, and ≈8-fold more potent than diclofenac, 43.78 µM). Unfortunately, neither compound **6e** nor compound **6g** inhibited ROS production significantly.

#### Effects on TNF-α and IL-6 production in LPS-activated RAW 264.7 macrophages cells

LPS activates macrophages to induce the production of inflammatory cytokines, including TNF-α and IL-6^40^. Excessive cytokine production following LPS injection *in vivo* can result in an uncontrolled inflammatory response that leads to severe systemic inflammatory response, tissue damage, and sepsis^41^. Our findings showed that compounds **6i**, **6k**, and **6o** had IL-6 levels (84.60, 88.53, and 37.23 pg/mL) that are ∼2–4.5-fold lower than celecoxib (176.4 pg/mL). Compounds **6a**, **6e**, **6f**, **6h**, **6j**, **6m**, and **6p** (164.6–328.9 pg/mL) were significantly more potent than diclofenac (342.90 pg/mL).

In terms of inhibiting the production of the inflammatory mediator TNF-α, compound **6o** showed TNF-α level, which is ∼10 times lower than celecoxib (39.37 *vs.* 383.3 pg/mL) and nearly 17 times lower than diclofenac (666.6 pg/mL). Compounds **6b**, **6h**, and **6j** exhibited comparable inhibitory activities (779.60, 720.60, and 757.30 pg/mL, respectively) to that of diclofenac (666.6 pg/mL). Unfortunately, the other compounds showed lower activities compared to the two references, celecoxib and diclofenac (Table 3).

Interestingly, the *p*-fluorophenyl urea derivative carrying *p-*nitrophenyl at quinazoline C2 **6o** showed the highest activity in terms of inhibiting the production of either TNF-α or IL-6.

#### In vitro *anticancer screening against 60 NCI cancer cell lines panel*

Ten of the synthesised compounds that showed the best inhibitory activities, namely (**6a**, **6c**, **6d**, **6e**, **6j**, **6l**, **6m**, **6n**, **6o**, and **6p**), were sent for testing to the National Cancer Institute (NCI) Developmental Therapeutics Program (DTP), division of cancer treatment and diagnosis, NIH, Bethesda, Maryland, USA (www.dtp.nci.nih.gov)^30^. *In vitro* anticancer screening was carried out for the selected compounds at a single dose (10^−5^ M) against 60 different human tumour cell lines of nine different cancer cell types: leukaemia, lung, colon, CNS, melanoma, ovarian, renal, prostate, and breast cancers.

The screening results revealed that compound **6n** (the *p*-fluorophenyl urea derivative carrying *p-*fluorophenyl at quinazoline C2) showed the broadest anticancer activity against 32 out of the 60 NCI cell lines with growth inhibition percentages in the 10.09–57.11% range. Also, the *p*-fluorophenyl urea derivatives **6m** and **6o** showed anticancer activities against 19 (10.52–40.1%) and 21 (10.66–41.34%) cell lines, respectively.

Compounds **6e (**the unsubstituted phenyl urea derivative carrying *p-*nitrophenyl at quinazoline C2) and **6j** (*p*-methoxyphenyl urea derivative carrying *p-*methoxyphenyl at quinazoline C2) exhibited the most potent anticancer activity against breast cancer cell line BT-459 with growth inhibition percentages of 67.14 and 70.07%, respectively. In addition, compound **6e** exhibited good anticancer activity against colon cancer cell line HCT-116 and melanoma cell line M14 with cell growth inhibition percentages of 53.14 and 67.23%, respectively (Table 4).

**Table 4.** The growth inhibition percentages for *in vitro* panel/cell lines from the single dose (10^−5^ M) test of compounds (**6a**, **6c–e**, **6j**, **6l–p**).

Panel/cell line	Compound									
	6a	6c	6d	6e	6j	6l	6m	6n	6o	6p
Leukaemia										
CCRF-CEM	–	–	–	–	–	–	–	–	–	–
HL-60(TB)	–	–	–	–	–	–	–	–	–	–
K-562	–	–	–	–	–	–	–	22.06	17.47	–
MOLT-4	–	–	11.08	–	–	–	23.47	43.09	20.40	11.25
RPMI-8226	–	–	–	–	–	–	16.14	33.09	22.40	11.25
SR	–	–	–	–	11.60	–	–	14.42	12.23	–
Non-small cell lung cancer										
A549/ATCC	–	–	–	–	–	–	–	–	–	–
EKVX	–	–	–	–	–	–	10.52	16.37	19.84	–
HOP-62	–	–	–	–	–	–	–	13.31	–	–
HOP-92	–	–	–	–	–	–	–	–	–	–
NCI-H226	–	–	–	–	–	–	–	20.96	–	–
NCI-H23	–	–	–	–	–	–	–	–	–	–
NCI-H322M	–	–	–	–	–	–	–	–	–	–
NCI-H460	–	–	–	–	–	–	–	–	–	–
NCI-H522	–	–	–	–	–	–	–	–	–	–
Colon cancer										
COLO 205	–	–	–	–	–	–	–	–	–	–
HCC-2998	–	–	–	–	–	–	–	–	–	–
HCT-116	–	16.84	–	53.14	40.57	–	–	22.85	21.65	13.98
HCT-15	–	–	–	–	–	–	14.29	17.48	10.66	–
HT29	–	–	–	–	–	–	–	–	–	–
KM12	–	–	–	–	–	–	–	15.37	–	–
SW-620	–	–	–	–	–	–	–	–	–	–
CNS Cancer										
SF-268	–	–	–	–	–	–	–	21.73	–	–
SF-295	–	–	–	–	–	–	–	–	–	–
SF-539	–	–	–	–	–	–	–	10.09	–	–
SNB-19	–	–	–	–	–	–	–	–	11.43	–
SNB-75	–	–	13.77	–	–	–	–	28.06	–	–
U251	–	–	–	–	–	–	–	–	–	–
Melanoma										
LOX IMVI	–	–	–	–	–	–	21.92	31.59	29.49	18.10
MALME-3M	–	–	–	–	–	–	10.82	17.56	14.13	–
M14	–	–	–	67.23	29.65	–	18.94	23.17	17.96	11.85
MDA-MB-435	–	–	–	–	–	–	–	25.82	19.93	10.69
SK-MEL-2	–	–	–	–	–	–	–	12.52	–	–
SK-MEL-28	–	–	–	–	–	–	–	–	–	–
SK-MEL-5	–	–	–	–	–	–	15.95	20.31	13.69	–
UACC-257	–	–	–	–	21.88	–	–	–	–	–
UACC-62	–	–	15.09	–	–	–	20.7	38.12	27.36	–
Ovarian cancer										
IGROV1	–	–	–	–	–	–	–	–	–	–
OVCAR-3	–	11.72	12.52	–	10.24	16.89	20.5	41.94	22.72	17.09
OVCAR-4	–	–	–	–	–	–	10.82	29.72	16.41	–
OVCAR-5	–	–	–	–	–	–	–	–	–	–
OVCAR-8	–	–	–	–	–	–	–	–	–	–
NCI/ADR-RES	–	–	–	–	–	–	16.78	23.17	–	–
SK-OV-3	–	–	–	–	–	–	–	22.86	–	–
Renal cancer										
786-0	–	35.28	11.86	39.88	34.20	–	12.93	–	–	–
A498	–	–	–	–	–	–	–	–	–	–
ACHN	–	–	–	–	–	–	–	12.57	–	–
CAKI-1	–	–	13.02	–	–	–	–	25.05	18.37	–
RXF 393	–	–	–	–	–	–	–	–T	–	–
SN12C	–	–	–	–	–	–	–	–	–	–
TK-10	–	–	–	–	–	15.42	–	–	–	–
UO-31	–	19.47	36.16	14.36	24.65	–	40.1	57.11	41.34	42.56
Prostate cancer										
PC-3	–	–	18.61	–	10.80	–	22.08	38.06	31.01	15.25
DU-145	–	–	–	–	–	–	–	–	–	–
Breast cancer										
MCF7	–	–	–	–	–	10.73	21.17	29.94	17.95	14.73
MDA-MB-231/ATCC	–	–	–	–	–	–	22.99	29.83	22.50	12.57
HS 578T	–	56.75	–	–	–	–	–	19.70	–	–
BT-549	–	–	–	67.14	70.07	–	–	–	–	–
T-47D	–	–	–	–	–	–	13.92	11.66	–	–
MDA-MB-468	–	–	–	–	–	–	–	–	–	–

Only growth inhibition (GI) percent higher than 10% are shown.

It is worth mentioning, that 8 out of the 10 tested compounds showed inhibitory activity against the ovarian cancer cell line OVCAR-3 and the renal cancer cell line UO-31 with cell growth inhibition ranging between 10.24–41.94 and 19.47–57.11%, respectively.

Unfortunately, compound **6a (**the unsubstituted phenyl urea derivative with no substituent at quinazoline C2) did not show any anticancer activities.

#### Cell viability assay against normal cell line

Cell viability testing for the newly synthesised compounds (**6a**, **6c**, **6d**, **6e**, **6j**, **6l**, **6m**, **6n**, **6o**, and **6p**) on RAW 264.7 cells showed that all the compounds were non-toxic and had a cell viability percentage that exceeded 90% of the control values when tested at concentrations up to 50 μM. Moreover, most of the compounds showed a cell viability percentage in the 83–100% range at a concentration of 100 μM, indicating their safety and selectivity (Supplementary Material Figure 1).

### Molecular modelling

#### Docking study into COX-1/COX-2

The docking studies into COX-1 (PDB: 1EQG)^42^ and COX-2 (PDB: 1CX2)^43^ were initiated by re-docking the co-crystallised ligands; ibuprofen and SC-558 [1-phenylsulfonamide-3-trifluoromethyl-5-parabromophenylpyrazole] into the active sites, respectively. The results revealed that the re-docked ibuprofen and SC-558 showed 0.7825 and 0.5734 Å for RMSD values, respectively, validating the docking procedure.

The targeted compounds **6a–p** were docked into both COX-1 and COX-2. In order to understand the biological results; the binding modes, docking energy, and interactions of the compounds were assessed against the co-crystallised ligands, ibuprofen, and SC-588. The docking energy for the compounds **6a–p** ranged from −8.4697 to −5.4624 Kcal/mol for COX-2 and −6.5881 to −5.3863 Kcal/mol for COX-1 (Supplementary Material Table 1).

Within the **6k–p** series bearing *p*-fluorophenyl urea, we found that compounds **6k**, **6n–p** have better COX-2 selectivity indices, revealed by the docking studies; as the *p*-fluorophenyl moiety (at urea linker) occupied a pocket surrounded by hydrophobic residues; Val116, Val349, Leu359, Trp387, Ile517, Phe518, and Ala527. Compounds **6k**, **6o**, and **6p** showed arene-H bonds with Tyr355, Leu531, and Ser353, respectively. Compound **2n** exhibited numerous H-bonds with Ser353, Val349, and Ala527 in addition to arene-H interaction with Tyr355, Trp387, and Leu531.

On the contrary, binding modes for the compounds **6k**, **6n–p** showed no interaction within the COX-1 active site except **6p,** which formed arene-H bond with Tyr355. Their *p*-fluorophenyl moiety (at urea linker) was surrounded with hydrophilic amino acid residues (Glu524 and Ser353), which might result in repulsion by the fluoro atom.

Within **6g–j** series bearing *p*-methoxyphenyl urea, methoxy group of the compounds **6h–i** showed hydrophobic contact with His90, Ser353, and Ala 516 in the COX-2 active site. In addition to arene-H and arene-cation formation with the quinazolinonyl moiety of the compound **6i**. The compounds were deeply oriented within the COX-2 pocket with S scores of −7.5751 and −7.1059 Kcal/mol, respectively. However, in COX-1, the quinazolinonyl moiety of the compounds **6h–i** protruded out from the pocket with S scores −6.1801 and −5.9169 Kcal/mol, respectively.

**Figure 3.**
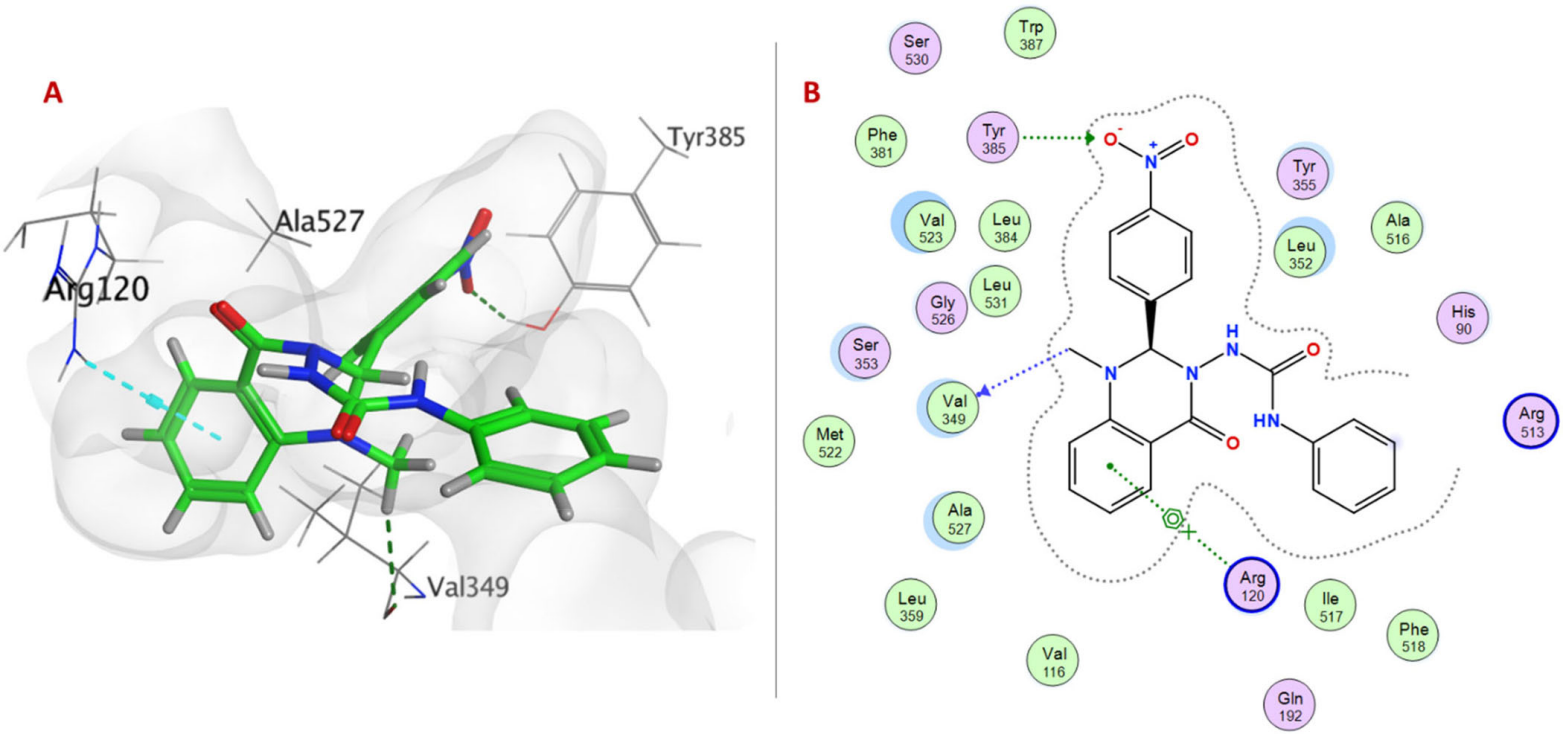
(A) 3D Interaction diagram of compound **6e** (thick green sticks) in the molecular surface of COX-2 (PDB: 1CX2) binding site. (B) 2D Interaction diagram of compound **6e** with amino acid residues of COX-2.

**Figure 4.**
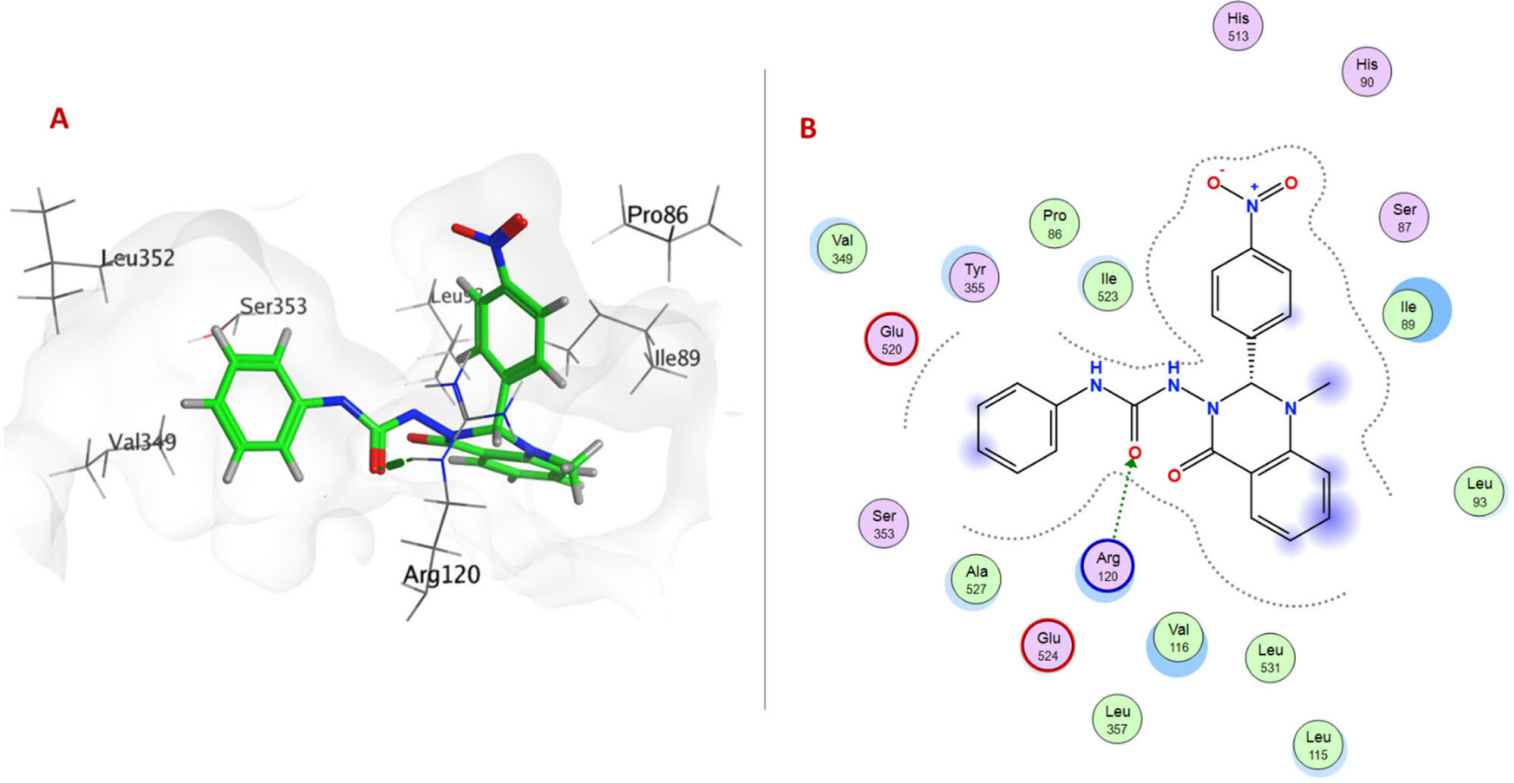
(A) 3D Interaction diagram of compound **6e** (thick green sticks) in the molecular surface of COX-1 (PDB: 1EQG) binding site. (B) 2D Interaction diagram of compound **6e** with amino acid residues of COX-1.

Within **6a–f** series (unsubstituted phenyl urea derivatives), compound **6e** showed better COX-2 selectivity (S score −8.4697 Kcal/mol) as *p*-nitrophenyl ring allowed the compound to occupy the side pocket and exhibited H-bond with Tyr385 (Figure 3). Moreover, the quinazolinonyl moiety of the compound **6e** was stabilised by H-bond and arene-cation formation with Val349 and Arg120, respectively. On the other hand, compound **6e** exhibited a different binding orientation with COX-1, as the urea linker formed a conventional H-bond with Arg120 (S score −6.1528 Kcal/mol) (Figure 4).

These results showed that the aforementioned compounds would have higher affinities to COX-2 over COX-1, in concordance with their selectivity towards COX-2.

#### Docking study into 15-LOX

The docking study into 15-LOX (PDB: 4NRE)^44^ was initiated by re-docking the co-crystallised ligand; C8E [hydroxyethyloxy)tri(ethyloxy)octane] into the active site. The results revealed that the re-docked ligand showed 1.3448 Å for RMSD value.

Docking simulations of the targeted compounds **6a–p** into 15-LOX active site afforded good binding energy ranging from −7.0847 to −5.8295 Kcal/mol (Supplementary Material Table 2).

Compound **6c** exhibited the best docking binding energy (−7.0847 Kcal/mol); matching the *in vitro* 15-LOX binding assay (1.36 µM). As the compound **6c** showed several hydrophobic interactions with Phe184, Tyr185, Ala188, Phe192, Leu419, Gln425, Arg429 and Leu609 residues. Moreover, compound **6c** was stabilised through H-bond formation with Leu419.

**Figure 5.**
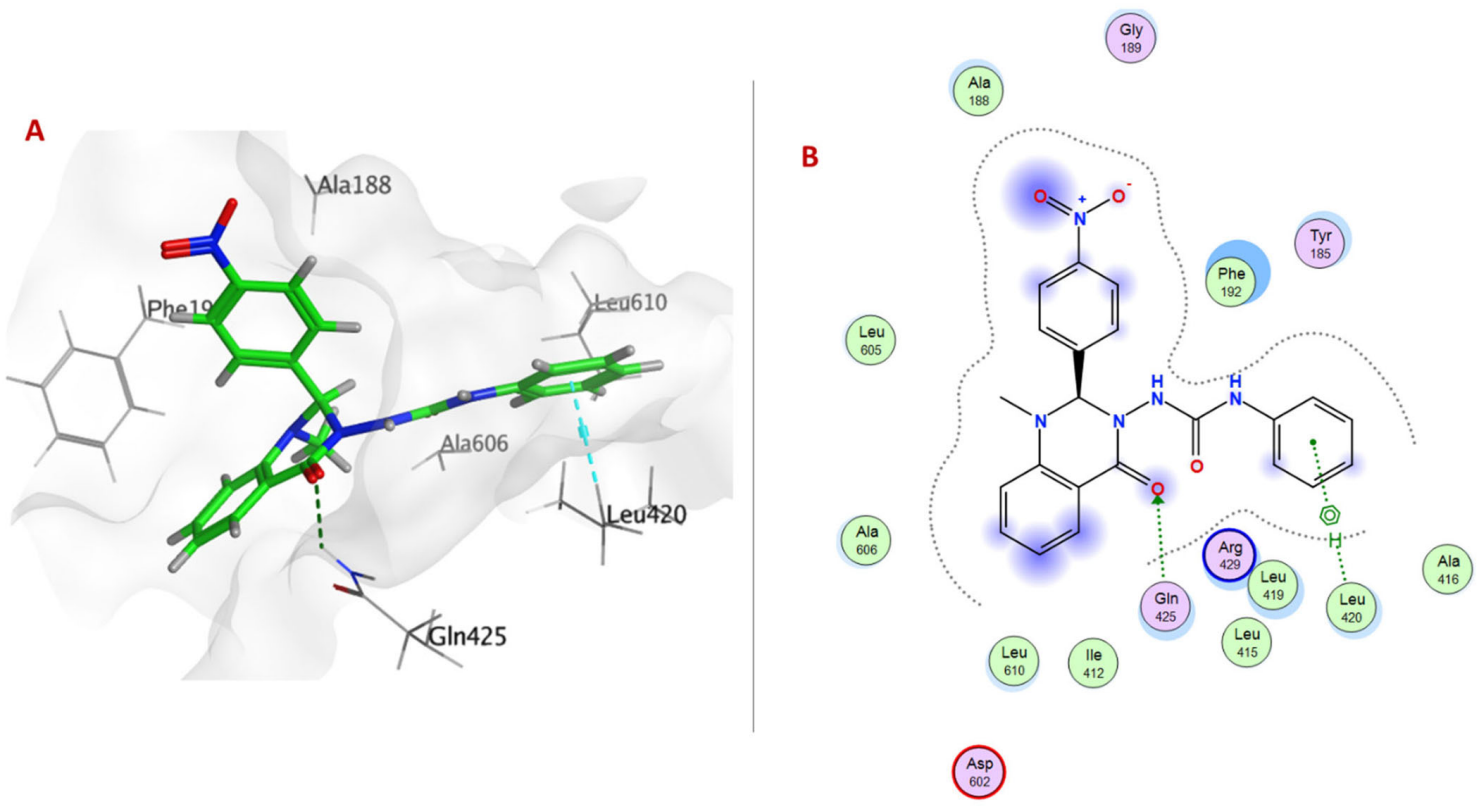
(A) 3D Interaction diagram of compound **6e** (thick green sticks) in the molecular surface of 15-LOX (PDB: 4NRE) binding site. (B) 2D Interaction diagram of compound **6e** with amino acid residues of 15-LOX.

**Figure 6.**
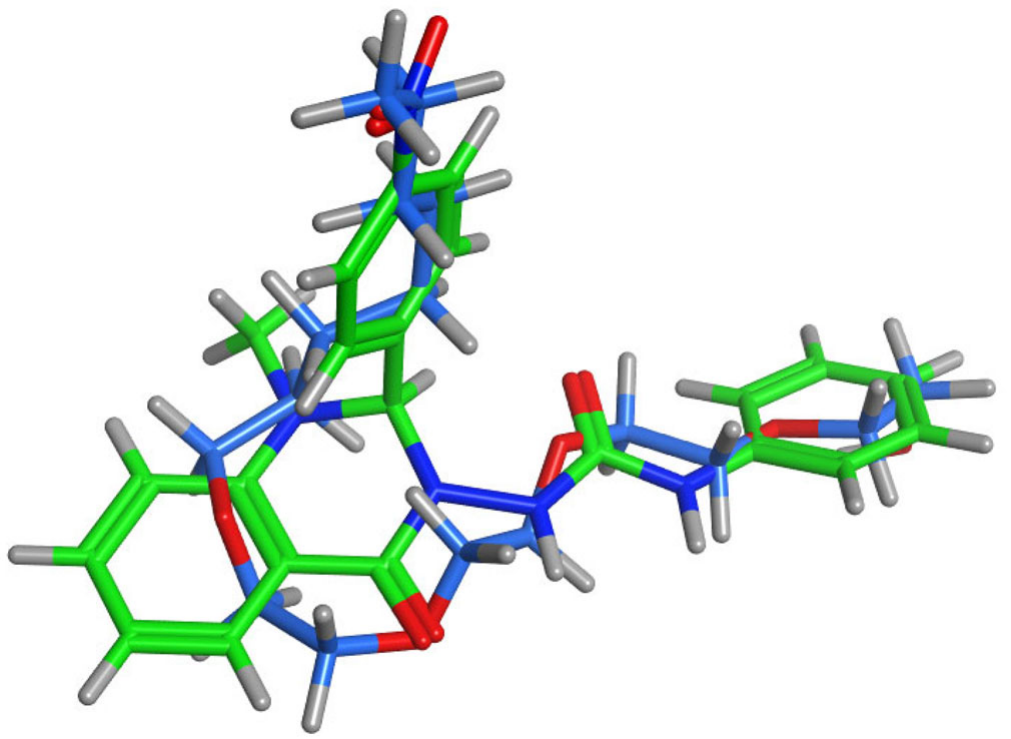
Flexible alignment of compound **6e** (thick green sticks) with the co-crystallised ligand; C8E [hydroxyethyloxy)tri(ethyloxy)octane] (thick blue sticks) of 15-LOX (PDB: 4NRE).

Quinazolinone ring in compounds **6a**, **6e** (Figure 5), **6f**, **6h–j**, **6m**, **6o**, **6p** formed either arene-arene, arene-H, or H-bond interactions with one or two of the following amino acid residues: Phe184, Phe192, Leu420, Arg429, Gln425, or Ala606.

Urea linker in compounds **6b–d**, **6h**, **6i**, **6k–n**, or **6p** formed H-bond with one of the following amino acid residues: Leu419, Val426, Arg429, Asp602, or Ala606.

All compounds revealed stabilising non-covalent interactions could be responsible for the inhibitory effect against 15-LOX by blocking its binding pocket.

The co-crystallised ligand (C8E) has a U-shaped conformation in the active site^44^. Compound **6e** was examined for its conformational similarity with C8E through the flexible alignment approach in MOE 2019.0102.

As indicated from the 3D flexible alignment result, compound **6e** showed similar conformations to C8E and this was verified and approved by the docking study. The score of the configuration alignment (S) is −70.4956 Kcal/mol; indicating excellent better alignment (Figure 6).

#### Docking study into EGFR^WT^ and EGFR^L858R/T790M^

The docking studies into EGFR wild-type (EGFR^WT^, PDB: 1M17)^45^ and EGFR double mutant type (EGFR^L858R/T790M^, PDB: 5EDQ)^46^ were initiated by re-docking the co-crystallised ligands; erlotinib and 5N3 [*N*-(7-chloro-1*H*-indazol-3-yl)-7,7-dimethyl-2-(1*H*-pyrazol-4-yl)-5*H*-furo[3,4-*d*]pyrimidin-4-amine] into the active sites, respectively. The results revealed that the re-docked erlotinib and 5N3 showed 0.8681 and 1.3783 Å for RMSD values, respectively. The binding modes of the compounds were evaluated by docking into the ATP binding sites of both EGFR^WT^ and EGFR^L858R/T790M^. The docking energy scores for the compounds **6a–p** ranged from −7.4186 to −6.6860 Kcal/mol for EGFR^WT^ and −8.1780 to −6.5123 Kcal/mol for EGFR^L858R/T790M^ (Supplementary Material Table 3).

Within EGFR^WT^, the quinazolinone ring in compound **6e** formed H-bond with Met769 and arene-H interactions with both Leu694 and Gly772. One NH group of the urea linker of compound **6o** showed H-bond with the acidic Asp831 in addition to an H-bond between the nitro group and Met769.

Within EGFR^L858R/T790M^; urea linker allowed the compounds **6a–p** to be oriented into the ATP binding site and exposed to the key amino acid residues. Urea linker in compounds **6a**, **6c–e**, **6g**, **6h**, **6j**, and **6m–o** formed H-bond with one or two amino acid residues: Lys745, Glu762, Met790, and Thr854. The formation of a non-covalent bond with Met790 showed EGFR^L858R/T790M^ selectivity over EGFR^WT^. As Met790 is considered a gatekeeper residue of EGFR^L858R/T790M^^47^^,^^48^.

**Figure 7.**
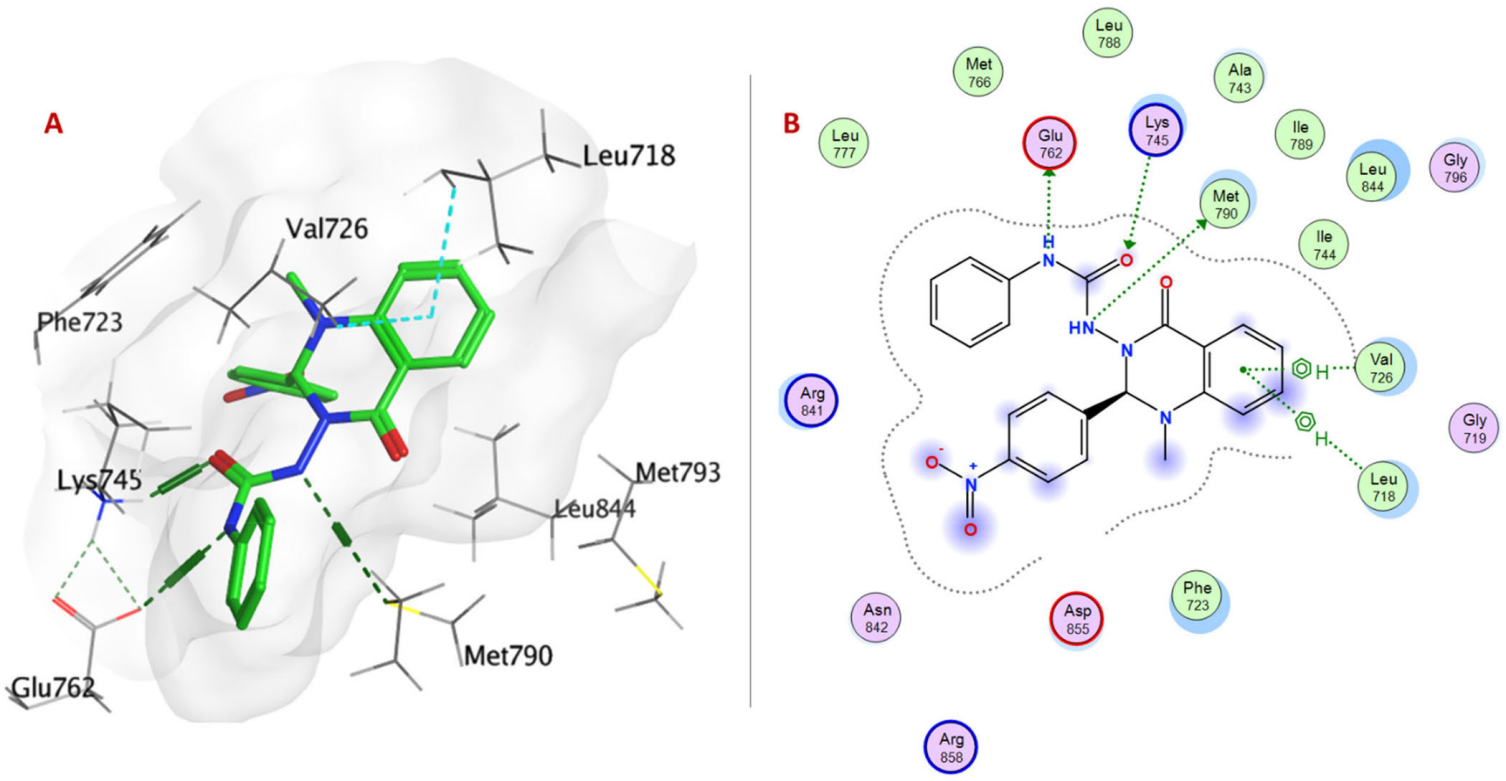
(A) 3D Interaction diagram of compound **6e** (thick green sticks) in the molecular surface of EGFR^L858R/T790M^ (PDB: 5EDQ) binding site. (B) 2D Interaction diagram of compound **6e** with amino acid residues EGFR^L858R/T790M^.

Urea linker in compound **6e** is considered an anchor for positioning the rest of the compound in the ATP-binding pocket within EGFR^T790M/L858R^. Two NH groups exhibited H-bonds with Glu762 and Met790, and carbonyl group formed H-bonds with Lys745. Moreover, quinazolinone ring showed two arene-H interactions with Leu718 and Val726 (Figure 7).

**Figure 8.**
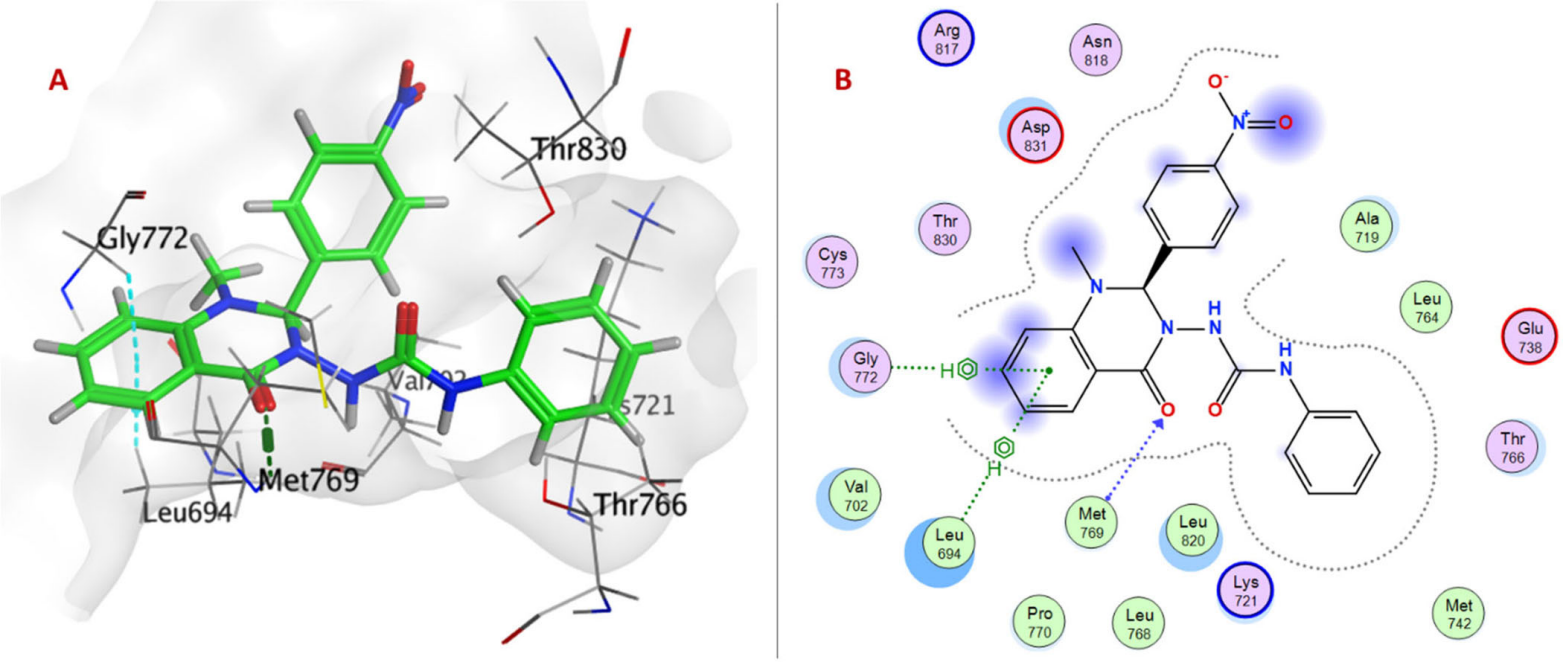
(A) 3D Interaction diagram of compound **6e** (thick green sticks) in the molecular surface of EGFR^WT^ (PDB: 1M17) binding site. (B) 2D Interaction diagram of compound **6e** with amino acid residues of EGFR^WT^.

On the other hand, only the quinazolinone ring of compound **6e** showed H-bond with Met769 and arene-H interactions with Leu694 and Gly772 within the ATP-binding pocket of EGFR^WT^ (Figure 8).

A summary of the crucial amino acid residues involved in hydrogen bonds, arene-arene, arene-cation, arene-H interactions, and remarkable docking energy scores of compounds **6a–p** with COX-1, COX-2, 15-LOX, EGFR^WT^, and EGFR^T790M/L858R^ are presented in Supplementary Material Tables 1–3. These results supported the *in vitro* biological results.

## Conclusion

Sixteen novel quinazolinone tethered phenyl urea derivatives (**6a–p**) were designed to triple target EGFR, COX-2, and 15-LOX. The Novel compounds (**6a–p**) were synthesised and their inhibitory activity evaluated against COX-1, COX-2, 15-LOX, wild-type EGFR, and the double mutant EGFR^L858R/T790M^. Their anti-inflammatory activities were also evaluated by testing their inhibitory effect of LPS-stimulated NO, ROS, TNF-α, and IL-6 production in RAW 264.7 macrophages. Moreover, the ten most active compounds were submitted to NCI for an anticancer activity assessment. Five compounds (**6e**, **6d**, **6j**, **6m**, and **6n**) showed moderate inhibitory activities against the three targets with low micromolar IC_50_ values (0.83–5.04, 1.79–2.23, and 4.03–13.52 µM for COX-2, 15-LOX, and the double mutant EGFR^L858R/T790M^, respectively). They also showed good selectivity for COX-2 over COX-1 and for EGFR ^L858R/T790M^ over wild-type EGFR. All the tested compounds **6a–p** inhibited the production of the inflammatory mediator NO (IC50 = 0.97–13.54 µM) significantly more potent compared to the three reference drugs celecoxib, diclofenac, and indomethacin (IC_50_ = 14.39, 24.08, and 46.45 µM, respectively) with the exception of compounds **6n** and **6e.** The most potent compounds in reducing ROS levels were compounds **6i** and **6k**, which showed IC_50_ values of 5.90 and 5.04 µM (≈1.5-fold more potent than that of celecoxib, 7.57 µM, ≈8-fold more potent than that of diclofenac, 43.78 µM). Interestingly, in terms of inhibiting the production of the inflammatory mediator TNF-α, compound **6o-**treated raw 264.7 cells showed TNF-α level, which is ∼10 times lower than that of celecoxib (39.37 *vs.* 383.3 pg/mL). Compound **6n** showed the broadest anticancer activity against 32 out of the 60 NCI cell lines with growth inhibition percentages in the 10.09–57.11% range. Compounds **6e** and **6j** exhibited the most potent anticancer activity against breast cancer cell line BT-459 with growth inhibition percentages of 67.14 and 70.07%, respectively.

These results were complemented by molecular docking studies, which identify the ability of the target compounds to make essential key interactions, known to be pivotal for EGFR, COX-2, and 15-LOX inhibitors. Our compounds could offer new structural insights into the understanding and development of triple-target EGFR^L858R/T790M^, COX-2, and 15-LOX inhibitors for anticancer outcomes.

## Supplementary Material

Supplemental Material
