# Supplementary material for: Triple targeting of mutant EGFRL858R/T790M, COX-2, and 15-LOX: design and synthesis of novel quinazolinone tethered phenyl urea derivatives for anti-inflammatory and anticancer evaluation
Source: J Enzyme Inhib Med Chem. 2023 Apr 11;38(1):2199166. doi: 10.1080/14756366.2023.2199166 (PMC10114980; doi:10.1080/14756366.2023.2199166)
Supplement: Supplemental Material [file IENZ_A_2199166_SM3187.pdf]

Supplementary material (1)

Triple Targeting of Mutant EGFR$^{L858R/T790M}$, COX-2 and 15-LOX: Design and Synthesis of Novel Quinazolinone Tethered Phenyl Urea Derivatives for Anti-Inflammatory and Anticancer Evaluation

Hend Kothayer[a *], Samar Rezq[b,c,d,e,f], Ahmed S. Abdelkhalek[a], Damian G. Romero[c,d,e,f], and Samar S. Elbaramawi[a]

[a]*Department of Medicinal Chemistry, Faculty of Pharmacy, Zagazig University Zagazig, Egypt*

[b]*Department of Pharmacology and Toxicology, Faculty of Pharmacy, Zagazig University, Egypt.*

[c]*Department of Cell and Molecular Biology,* [d]*Mississippi Center of Excellence in Perinatal Research,* [e]*Women's Health Research Center,* [f]*Cardiovascular-Renal Research Center, University of Mississippi Medical Center, Jackson, MS, USA*

[*]Corresponding author. Tel. 00201224578259; E-mail: hendo1311@hotmail.com or hkelhamalawy@pharmacy.zu.edu.eg

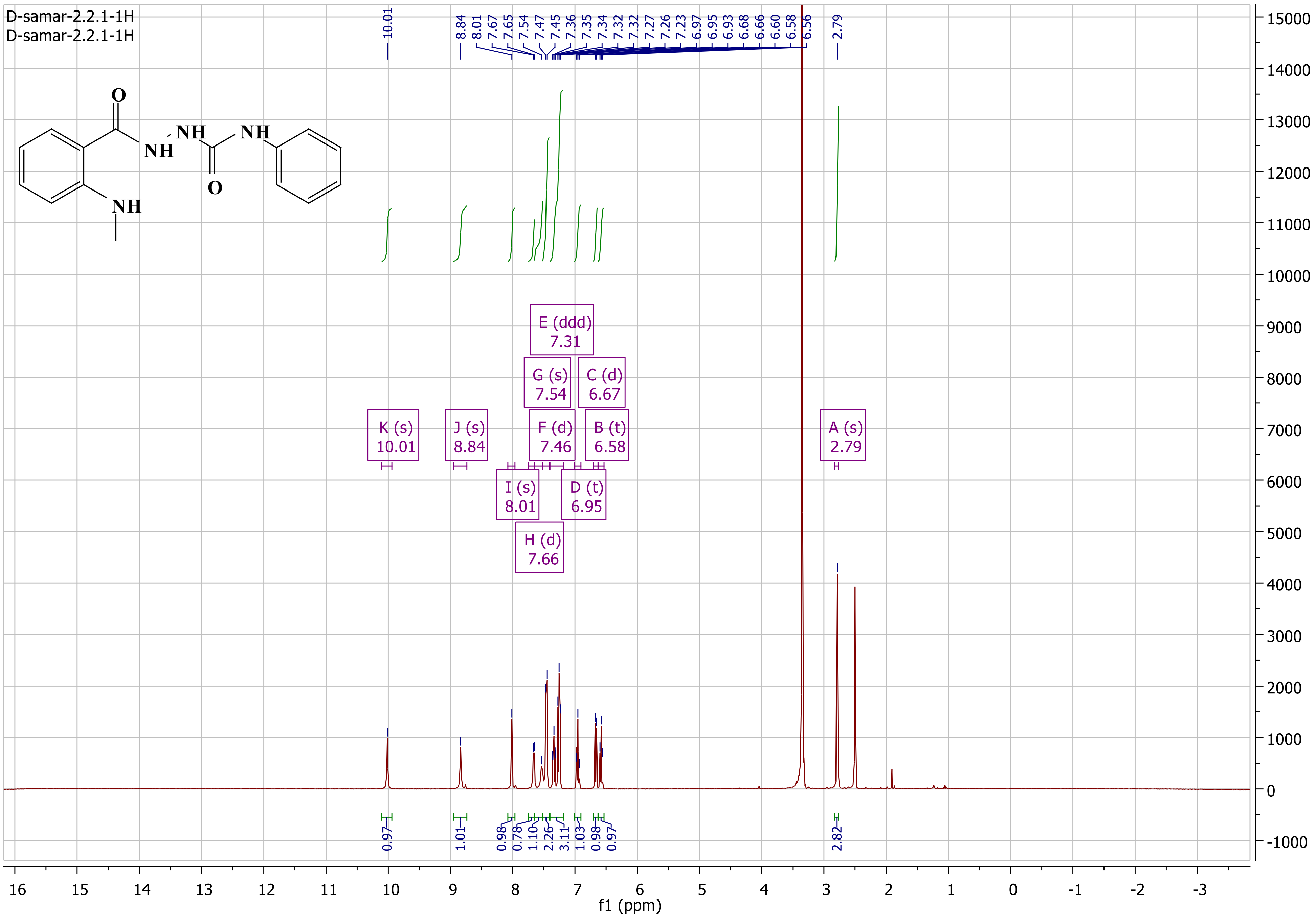
D-samar-2.2.1-1H
D-samar-2.2.1-1H
10.01
8.84
8.01
7.67
7.65
7.54
7.47
7.45
7.36
7.35
7.34
7.32
7.32
7.27
7.26
7.23
6.97
6.95
6.93
6.68
6.66
6.60
6.58
6.56
2.79
E (ddd) 7.31
G (s) 7.54
C (d) 6.67
K (s) 10.01
J (s) 8.84
F (d) 7.46
B (t) 6.58
A (s) 2.79
I (s) 8.01
D (t) 6.95
H (d) 7.66
0.97
1.01
0.98
0.78
1.10
2.26
3.11
1.03
0.98
0.97
2.82
f1 (ppm)

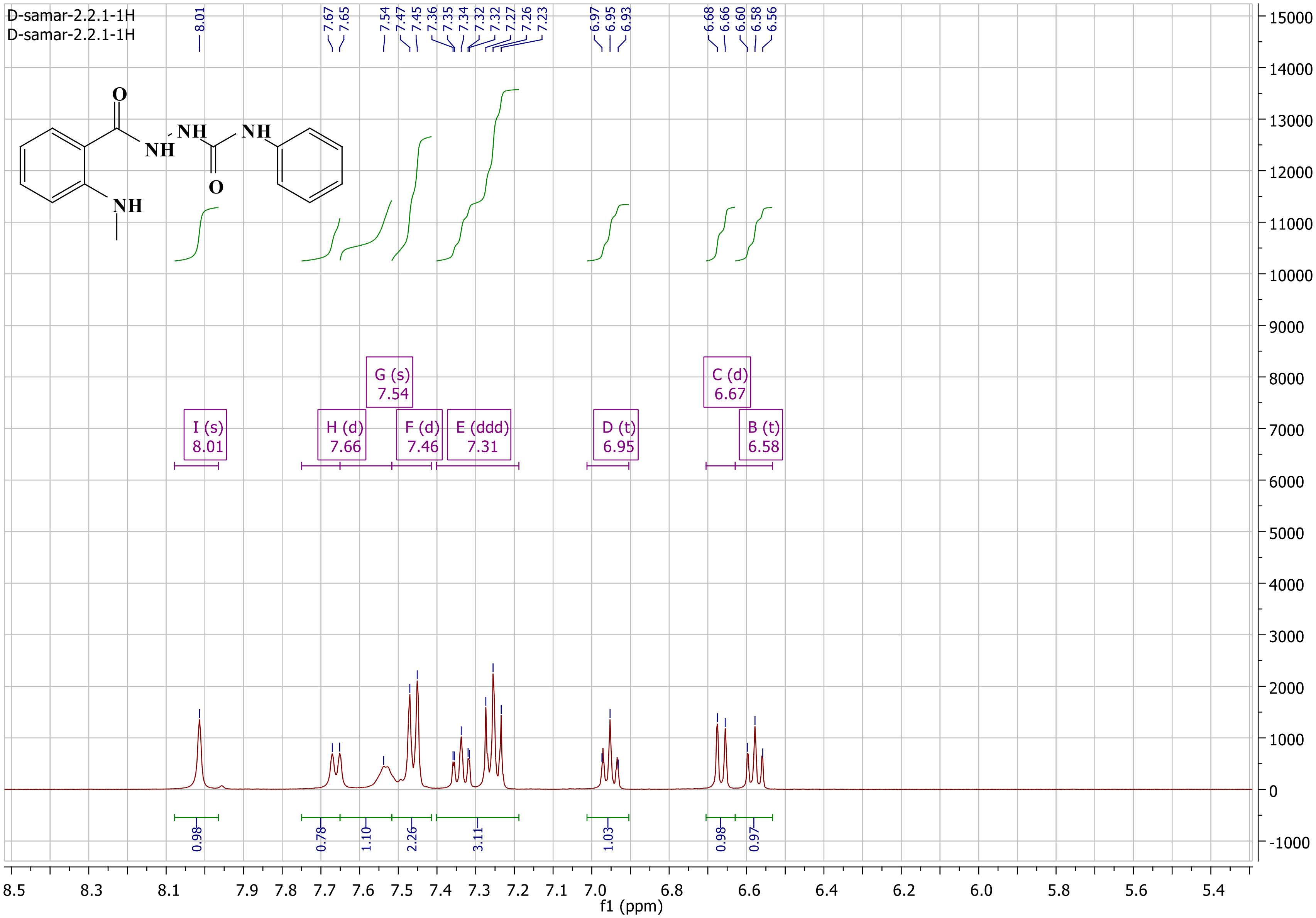

D-samar-2.2.1-1H
D-samar-2.2.1-1H
8.01
7.67
7.65
7.54
7.47
7.45
7.36
7.35
7.34
7.32
7.32
7.27
7.26
7.23
6.97
6.95
6.93
6.68
6.66
6.60
6.58
6.56
I (s) 8.01
H (d) 7.66
G (s) 7.54
F (d) 7.46
E (ddd) 7.31
D (t) 6.95
C (d) 6.67
B (t) 6.58
0.98
0.78
1.10
2.26
3.11
1.03
0.98
0.97
f1 (ppm)

D-samar-2-1H

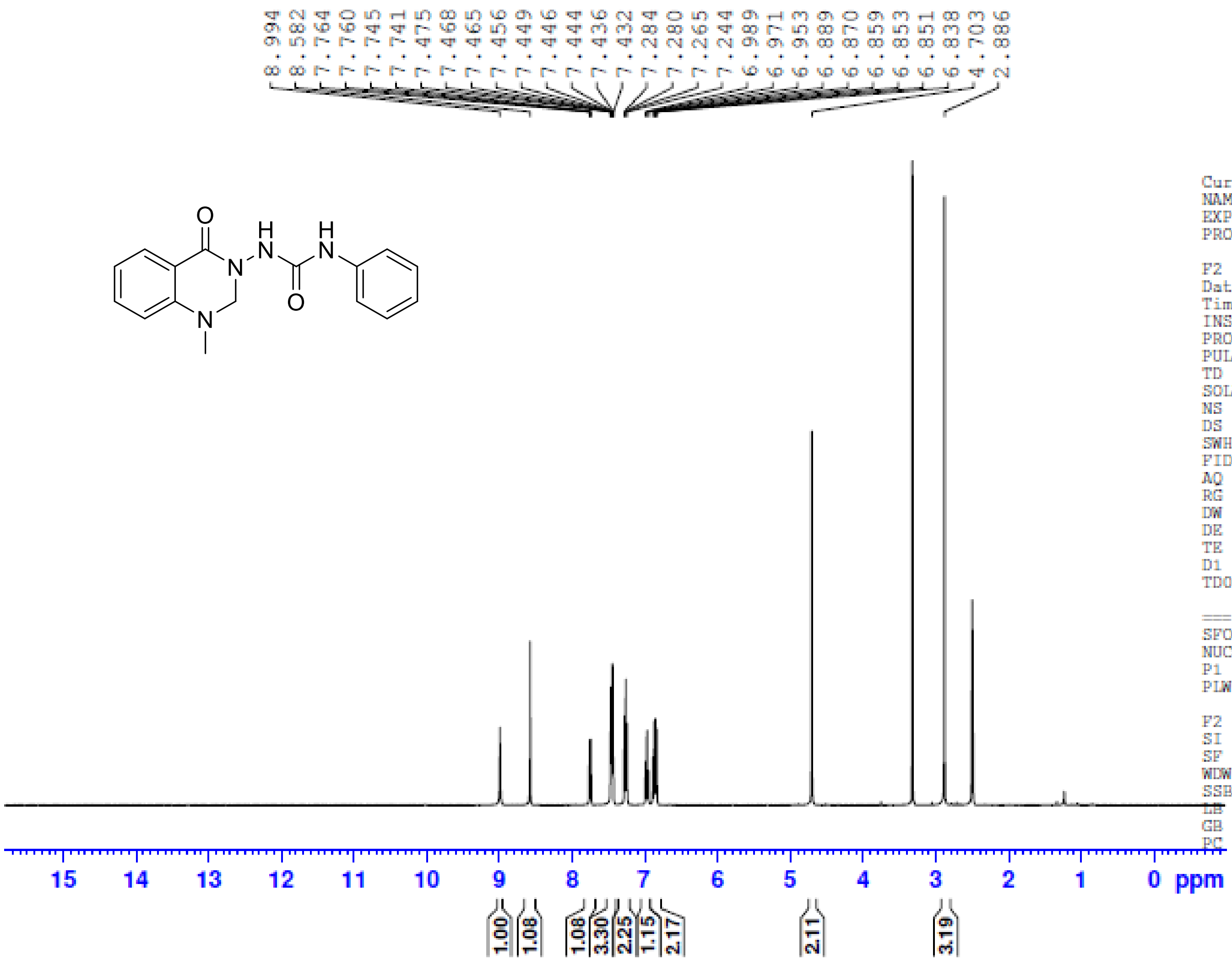

Current Data Parameters
NAME D-samar-2-1H
EXPNO 2
PROCNO 1

F2 - Acquisition Parameters
Date_ 20201201
Time 11.18
INSTRUM spect
PROBHD 5 mm PABBO BB/
PULPROG zg30
TD 65536
SOLVENT DMSO
NS 16
DS 2
SWH 8012.820 Hz
FIDRES 0.122266 Hz
AQ 4.0894465 sec
RG 201.61
DW 62.400 usec
DE 6.50 usec
TE 298.2 K
D1 1.00000000 sec
TD0 1

======= CHANNEL f1 =======
SFO1 400.1324710 MHz
NUC1 1H
P1 15.00 usec
PLW1 10.39999962 W

F2 - Processing parameters
SI 65536
SF 400.1300036 MHz
WDW EM
SSB 0
LB 0.30 Hz
GB 0
PC 1.00

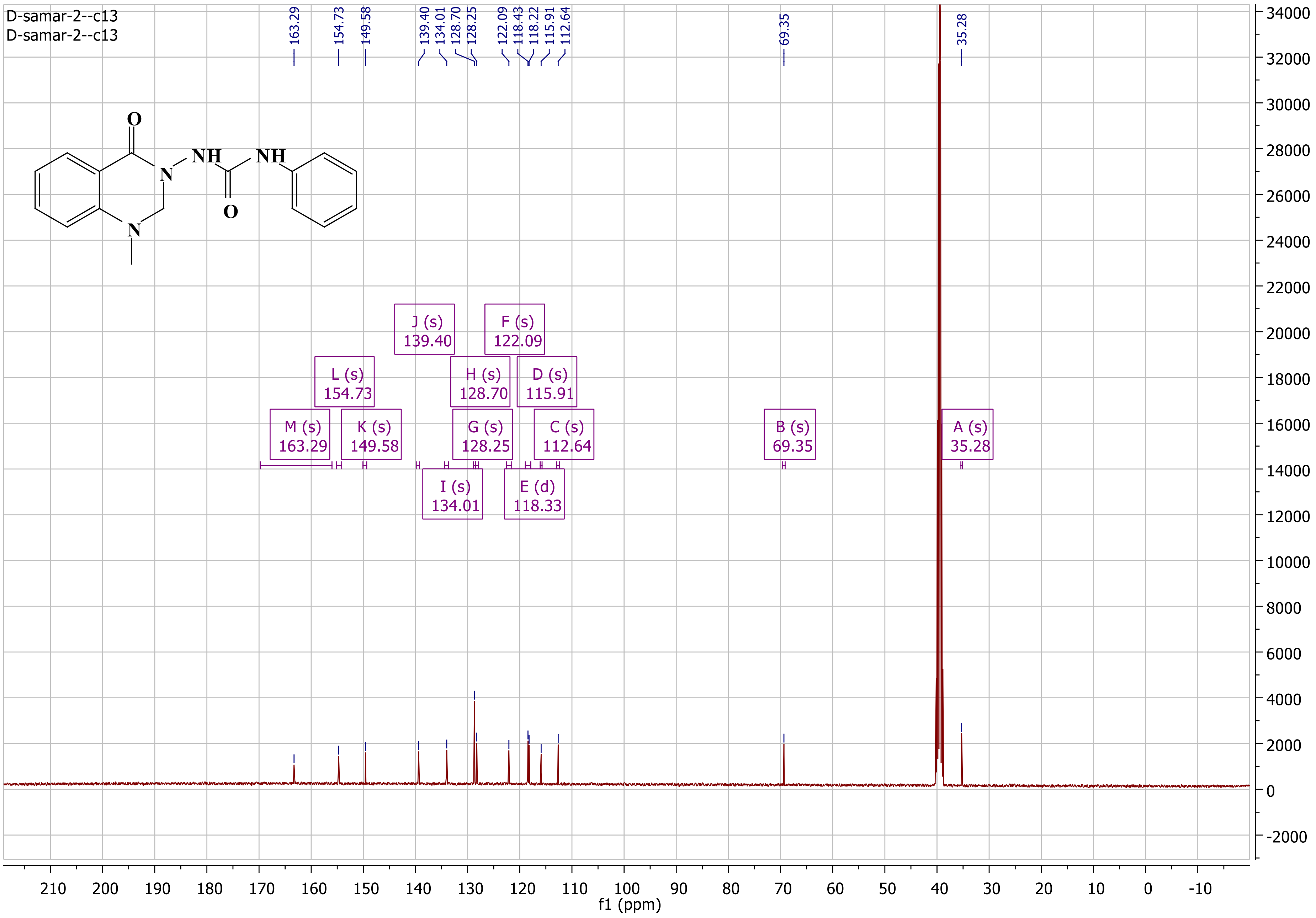

D-samar-2--c13
D-samar-2--c13
163.29
154.73
149.58
139.40
134.01
128.70
128.25
122.09
118.43
118.22
115.91
112.64
69.35
35.28
O
NH
NH
N
N
O
J (s) 139.40
F (s) 122.09
L (s) 154.73
H (s) 128.70
D (s) 115.91
M (s) 163.29
K (s) 149.58
G (s) 128.25
C (s) 112.64
B (s) 69.35
A (s) 35.28
I (s) 134.01
E (d) 118.33
f1 (ppm)

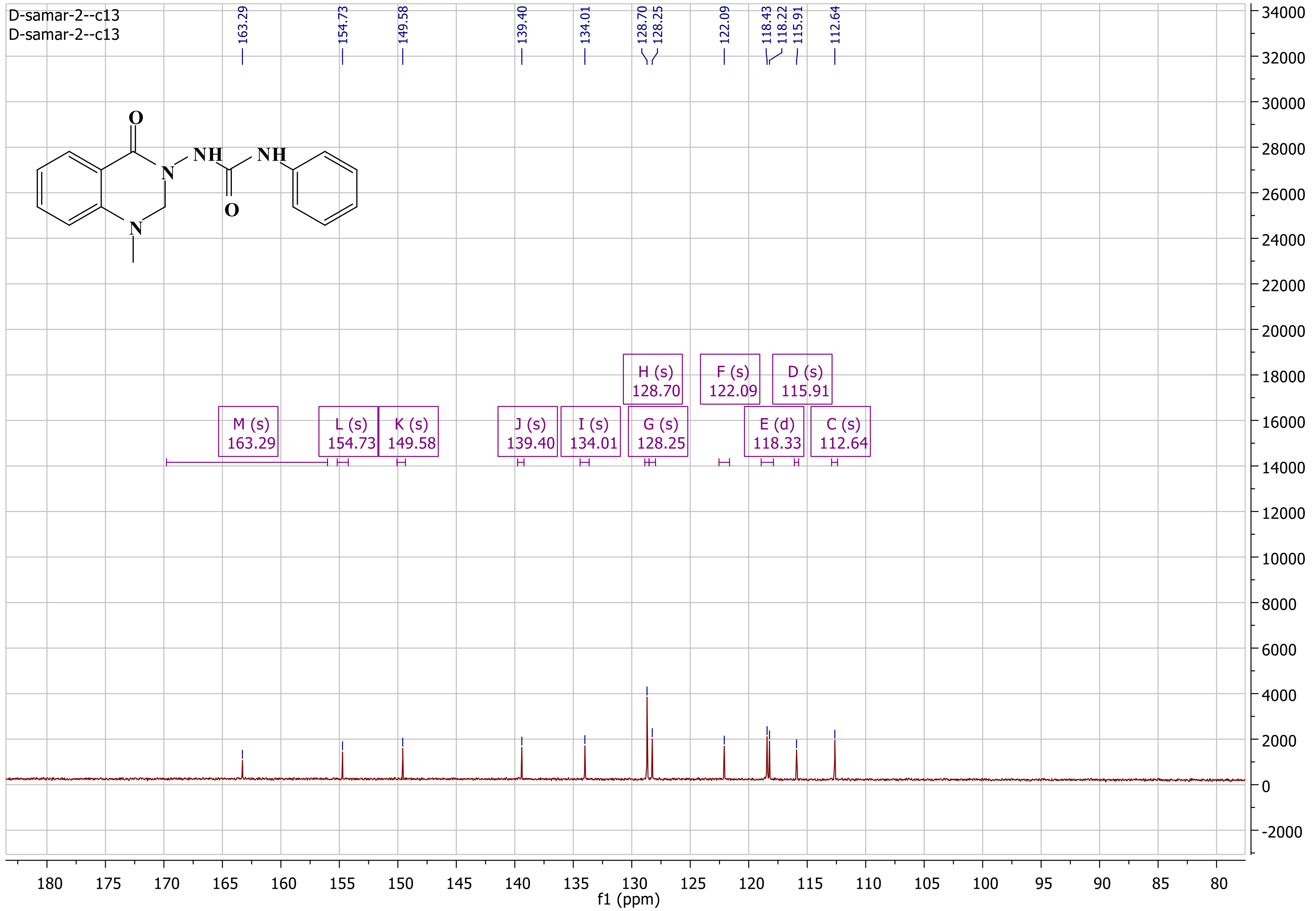
D-samar-2--c13
D-samar-2--c13
163.29
154.73
149.58
139.40
134.01
128.70
128.25
122.09
118.43
118.22
115.91
112.64
O
NH
NH
N
O
N
H (s)
128.70
F (s)
122.09
D (s)
115.91
M (s)
163.29
L (s)
154.73
K (s)
149.58
J (s)
139.40
I (s)
134.01
G (s)
128.25
E (d)
118.33
C (s)
112.64
f1 (ppm)

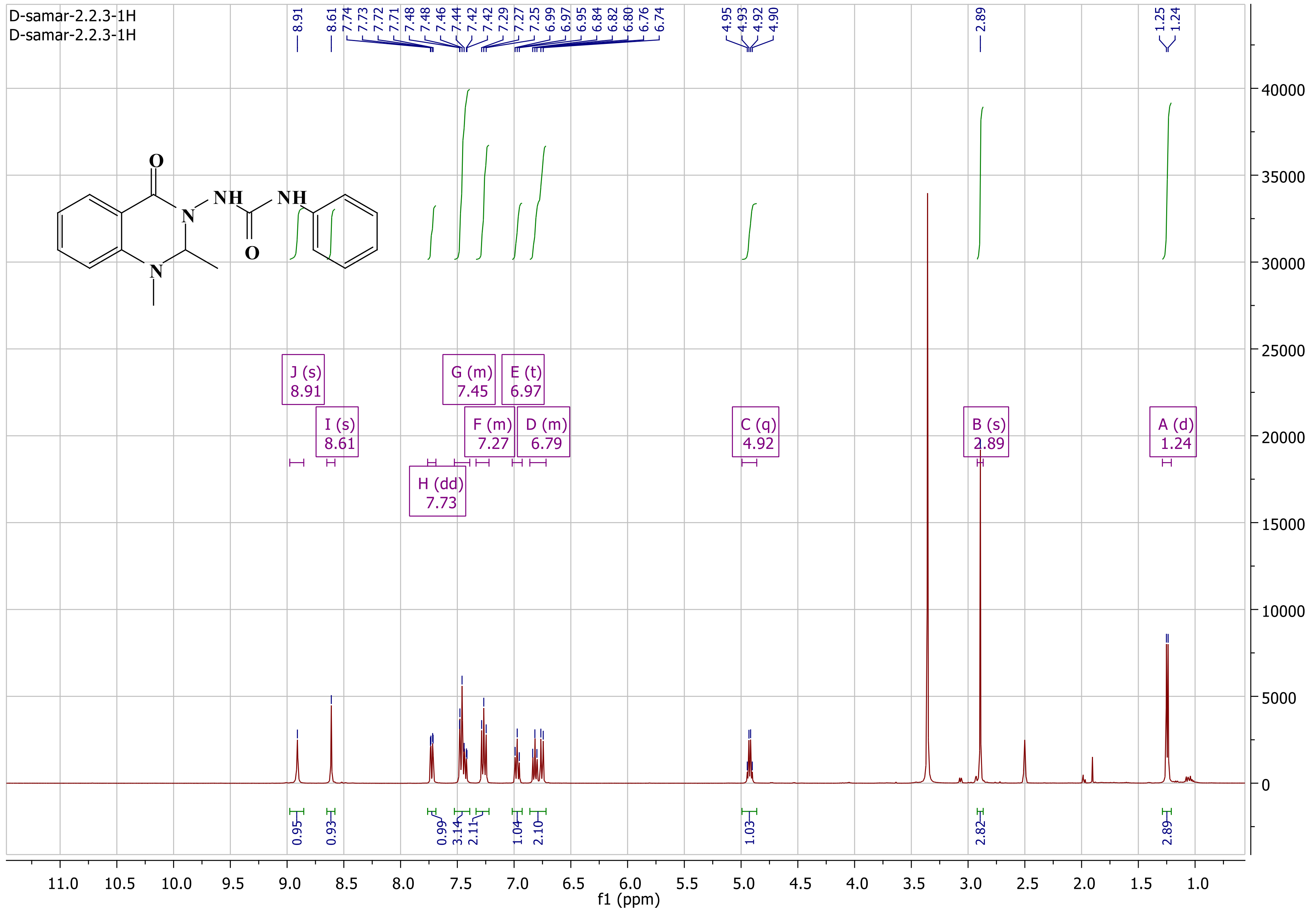

D-samar-2.2.3-1H
D-samar-2.2.3-1H
J (s) 8.91
I (s) 8.61
H (dd) 7.73
G (m) 7.45
F (m) 7.27
E (t) 6.97
D (m) 6.79
C (q) 4.92
B (s) 2.89
A (d) 1.24
f1 (ppm)

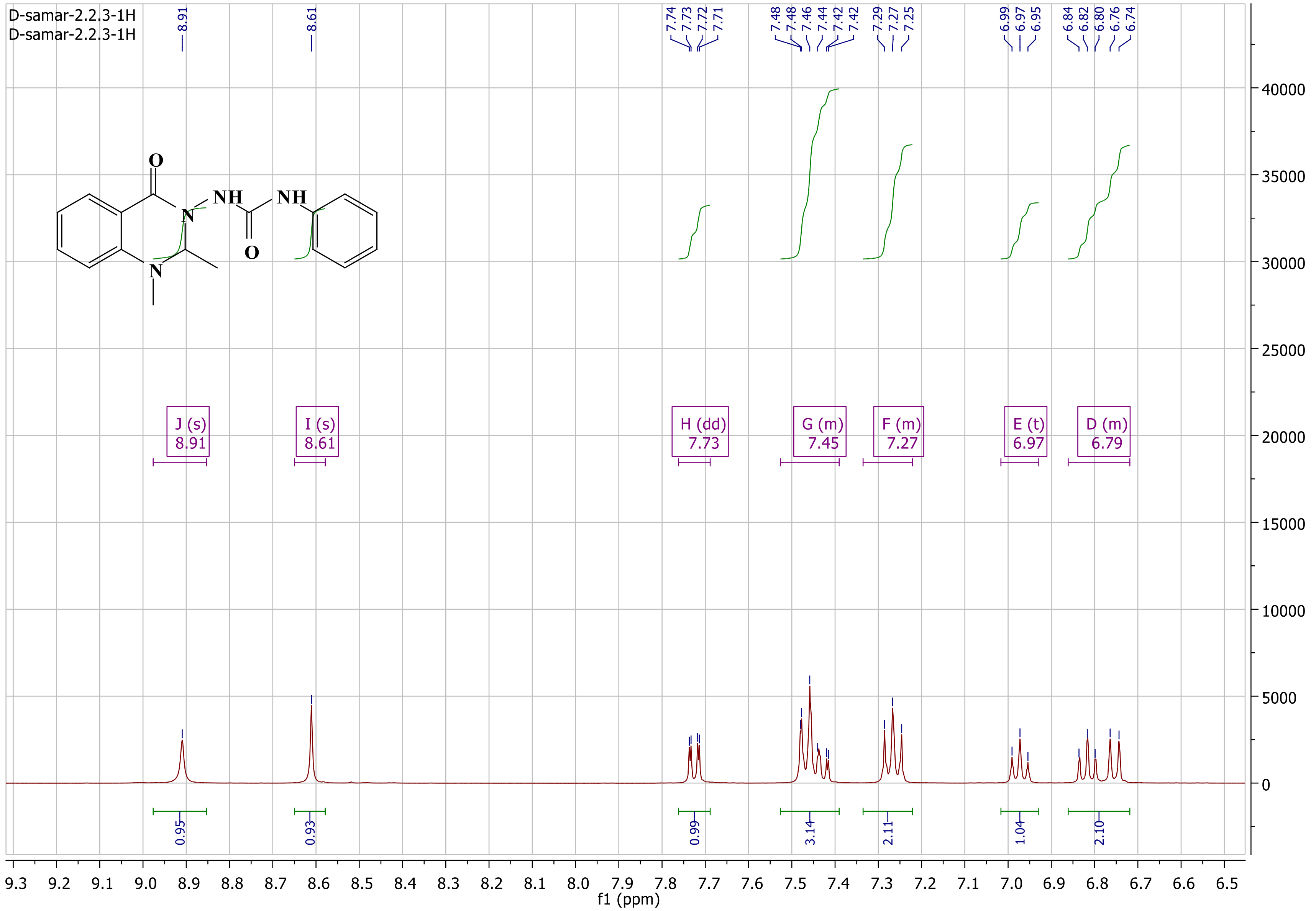

D-samar-2.2.3-1H
D-samar-2.2.3-1H
8.91
8.61
7.74 7.73 7.72 7.71
7.48 7.48 7.46 7.44 7.42 7.42
7.29 7.27 7.25
6.99 6.97 6.95
6.84 6.82 6.80 6.76 6.74
O
NH
NH
N
O
N
J (s) 8.91
I (s) 8.61
H (dd) 7.73
G (m) 7.45
F (m) 7.27
E (t) 6.97
D (m) 6.79
0.95
0.93
0.99
3.14
2.11
1.04
2.10
40000
35000
30000
25000
20000
15000
10000
5000
0
9.3 9.2 9.1 9.0 8.9 8.8 8.7 8.6 8.5 8.4 8.3 8.2 8.1 8.0 7.9 7.8 7.7 7.6 7.5 7.4 7.3 7.2 7.1 7.0 6.9 6.8 6.7 6.6 6.5
f1 (ppm)

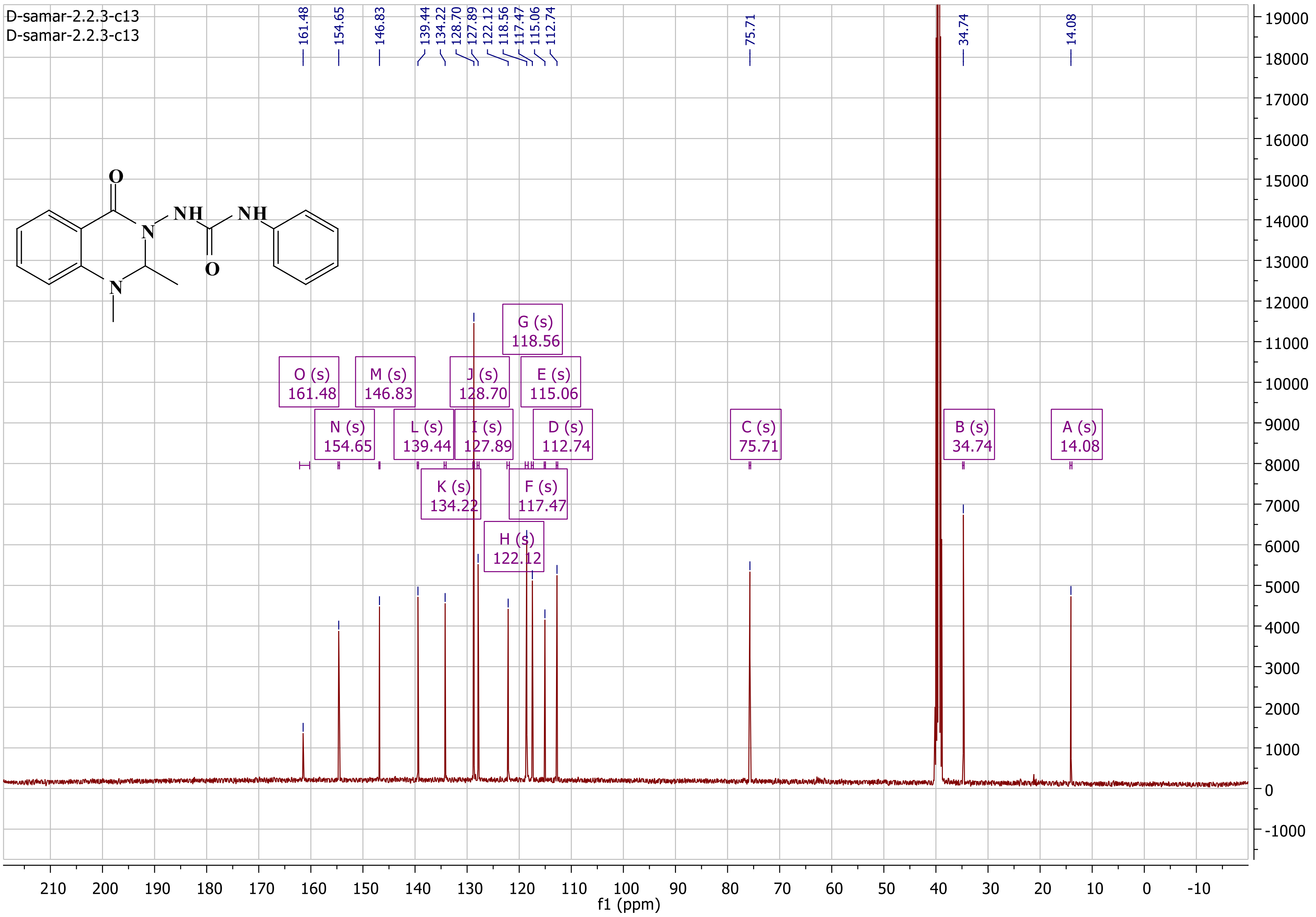
D-samar-2.2.3-c13
D-samar-2.2.3-c13
161.48
154.65
146.83
139.44
134.22
128.70
127.89
122.12
118.56
117.47
115.06
112.74
75.71
34.74
14.08
O
NH
NH
N
N
O
O (s) 161.48
N (s) 154.65
M (s) 146.83
L (s) 139.44
K (s) 134.22
J (s) 128.70
I (s) 127.89
H (s) 122.12
G (s) 118.56
F (s) 117.47
E (s) 115.06
D (s) 112.74
C (s) 75.71
B (s) 34.74
A (s) 14.08
f1 (ppm)

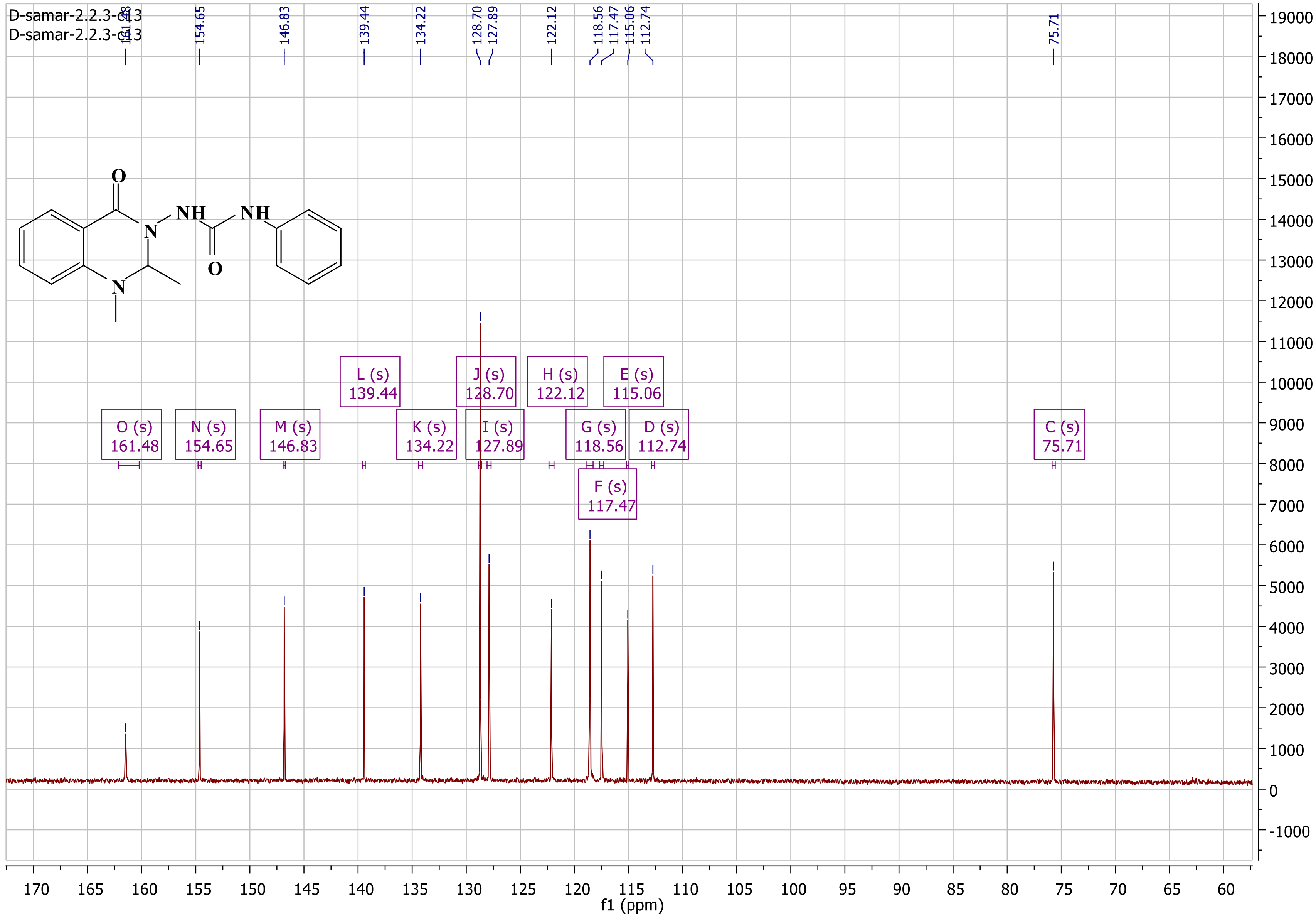
D-samar-2.2.3-c13
D-samar-2.2.3-c13
161.48
154.65
146.83
139.44
134.22
128.70
127.89
122.12
118.56
117.47
115.06
112.74
75.71
O
NH
NH
N
N
O
O (s) 161.48
N (s) 154.65
M (s) 146.83
L (s) 139.44
K (s) 134.22
J (s) 128.70
I (s) 127.89
H (s) 122.12
G (s) 118.56
F (s) 117.47
E (s) 115.06
D (s) 112.74
C (s) 75.71
f1 (ppm)

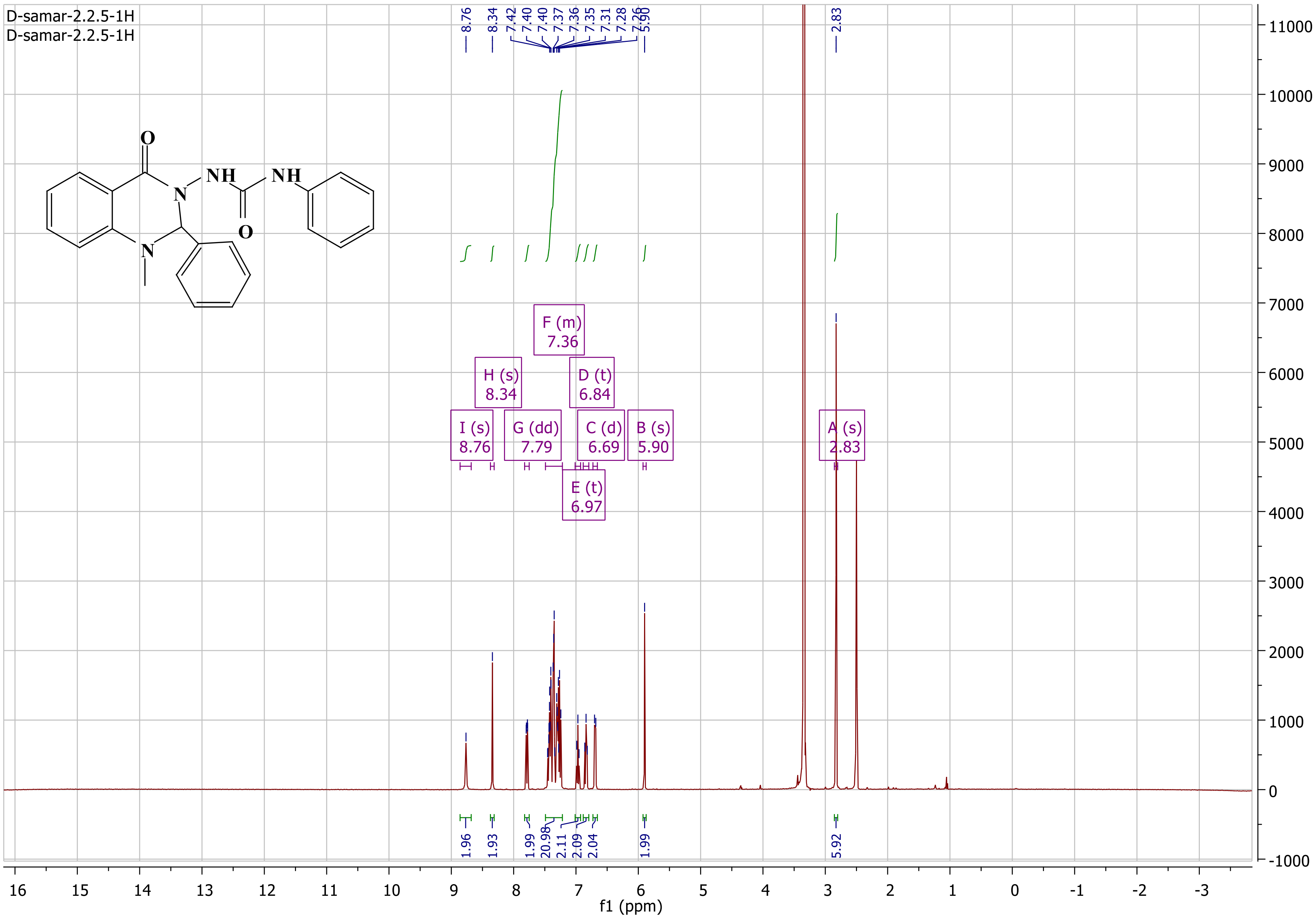
D-samar-2.2.5-1H
D-samar-2.2.5-1H
8.76
8.34
7.42
7.40
7.40
7.37
7.36
7.35
7.31
7.28
7.26
5.90
2.83
F (m) 7.36
H (s) 8.34
D (t) 6.84
I (s) 8.76
G (dd) 7.79
C (d) 6.69
B (s) 5.90
A (s) 2.83
E (t) 6.97
1.96
1.93
1.99
20.98
2.11
2.09
2.04
1.99
5.92
f1 (ppm)

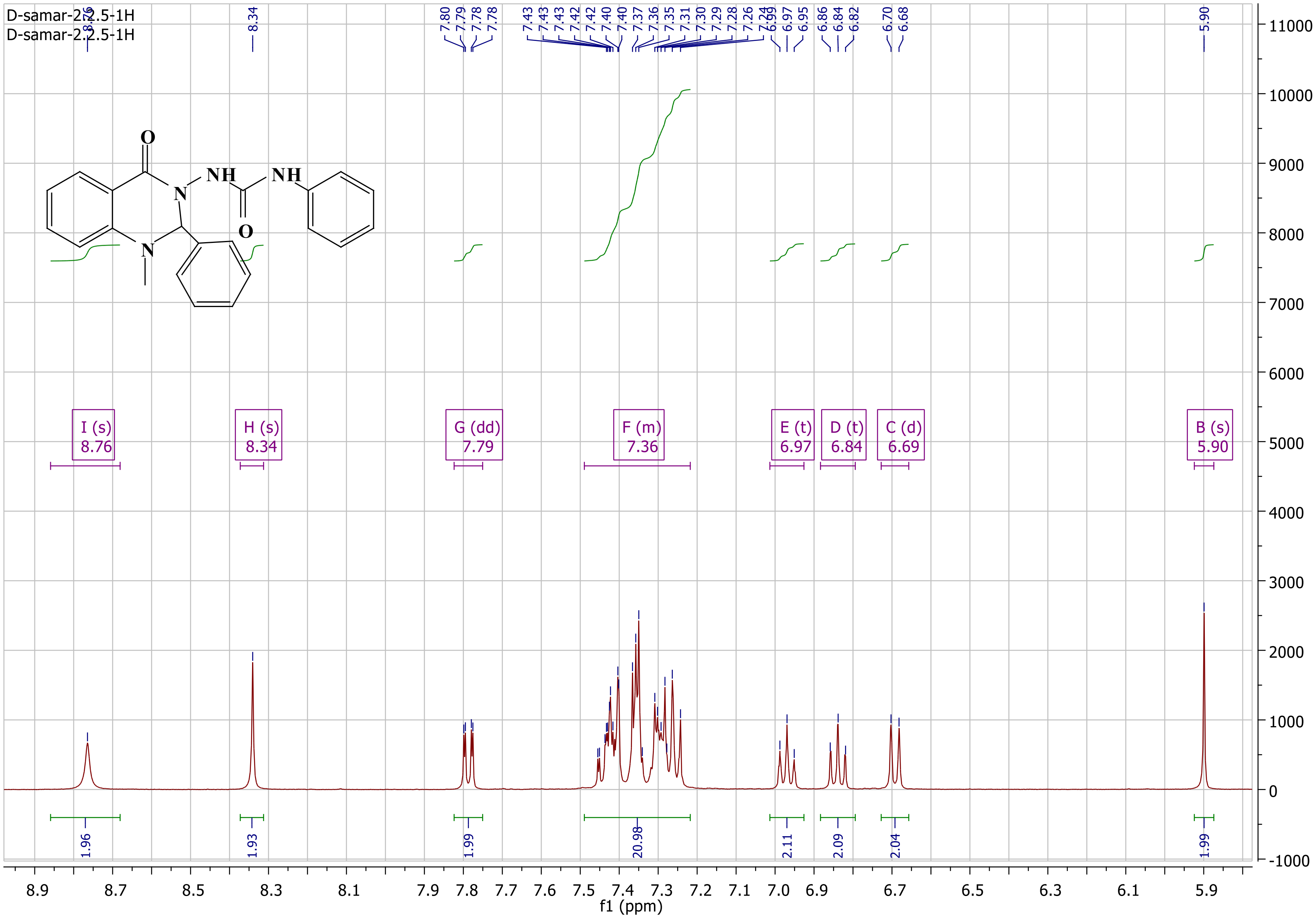
D-samar-2.2.5-1H
D-samar-2.2.5-1H
8.76
8.34
7.80 7.79 7.78 7.78
7.43 7.43 7.43 7.42 7.40 7.40 7.37 7.36 7.35 7.31 7.30 7.29 7.28 7.26 7.24
6.99 6.97 6.95
6.86 6.84 6.82
6.70 6.68
5.90
O
NH
NH
N
N
O
I (s) 8.76
H (s) 8.34
G (dd) 7.79
F (m) 7.36
E (t) 6.97
D (t) 6.84
C (d) 6.69
B (s) 5.90
1.96
1.93
1.99
20.98
2.11
2.09
2.04
1.99
11000
10000
9000
8000
7000
6000
5000
4000
3000
2000
1000
0
-1000
8.9 8.7 8.5 8.3 8.1 7.9 7.8 7.7 7.6 7.5 7.4 7.3 7.2 7.1 7.0 6.9 6.7 6.5 6.3 6.1 5.9
f1 (ppm)

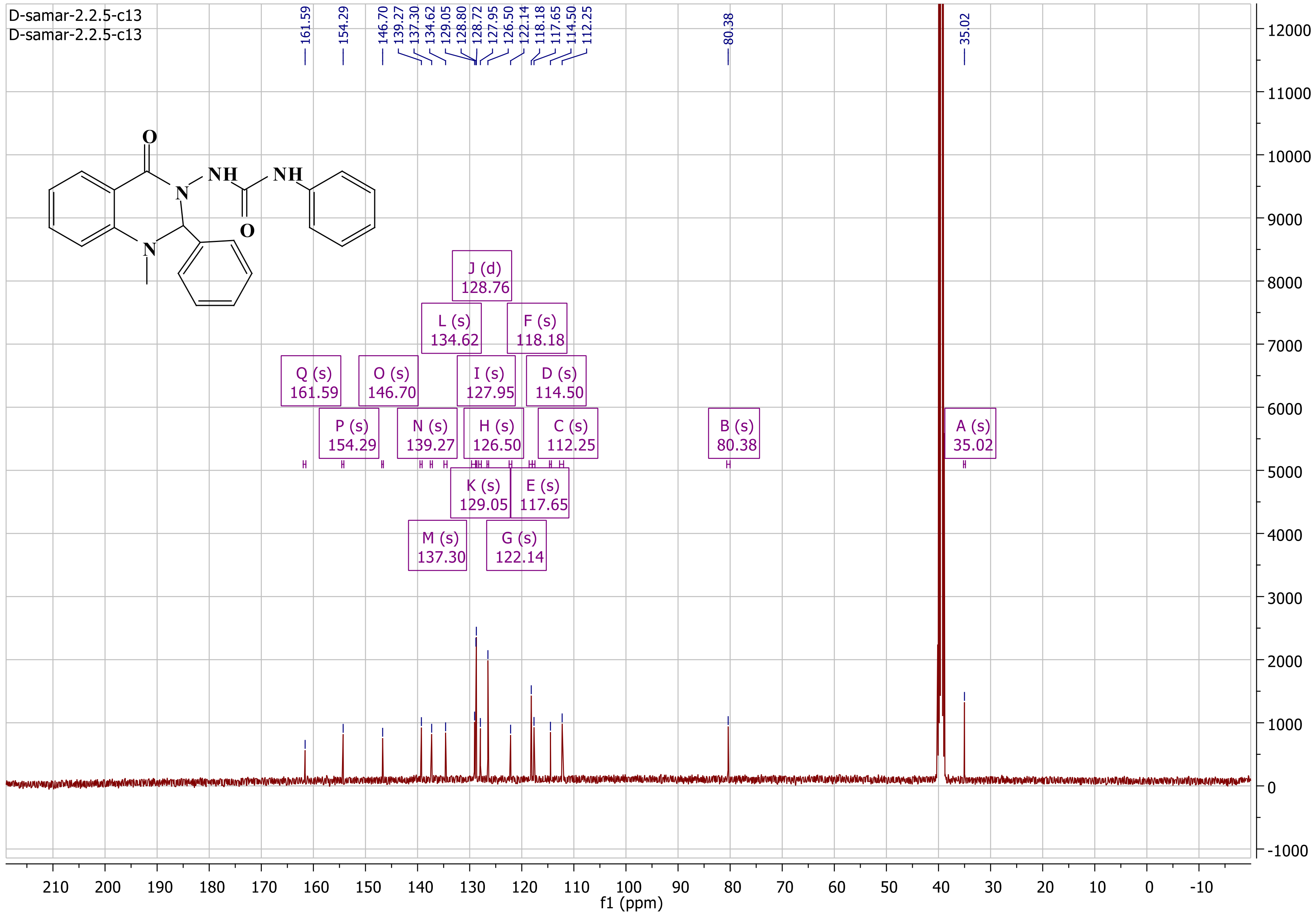
D-samar-2.2.5-c13
D-samar-2.2.5-c13
161.59
154.29
146.70
139.27
137.30
134.62
129.05
128.80
128.72
127.95
126.50
122.14
118.18
117.65
114.50
112.25
80.38
35.02
O
NH
NH
N
O
N
J (d) 128.76
L (s) 134.62
F (s) 118.18
Q (s) 161.59
O (s) 146.70
I (s) 127.95
D (s) 114.50
P (s) 154.29
N (s) 139.27
H (s) 126.50
C (s) 112.25
B (s) 80.38
A (s) 35.02
K (s) 129.05
E (s) 117.65
M (s) 137.30
G (s) 122.14
12000
11000
10000
9000
8000
7000
6000
5000
4000
3000
2000
1000
0
-1000
210 200 190 180 170 160 150 140 130 120 110 100 90 80 70 60 50 40 30 20 10 0 -10
f1 (ppm)

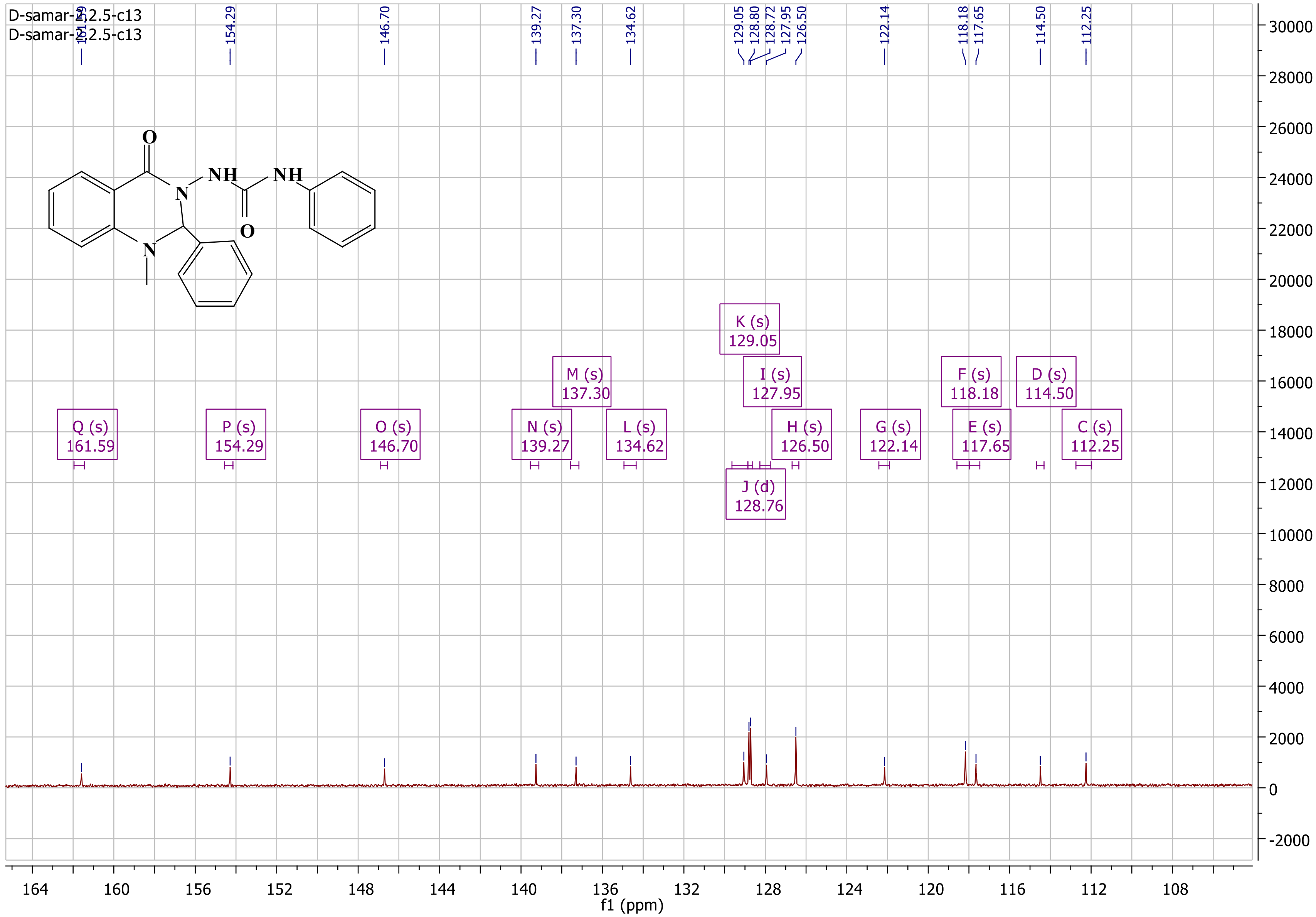

D-samar-
161.59
154.29
146.70
139.27
137.30
134.62
129.05
128.80
128.72
127.95
126.50
122.14
118.18
117.65
114.50
112.25
O
NH
NH
N
N
O
K (s) 129.05
M (s) 137.30
I (s) 127.95
F (s) 118.18
D (s) 114.50
Q (s) 161.59
P (s) 154.29
O (s) 146.70
N (s) 139.27
L (s) 134.62
H (s) 126.50
G (s) 122.14
E (s) 117.65
C (s) 112.25
J (d) 128.76
f1 (ppm)

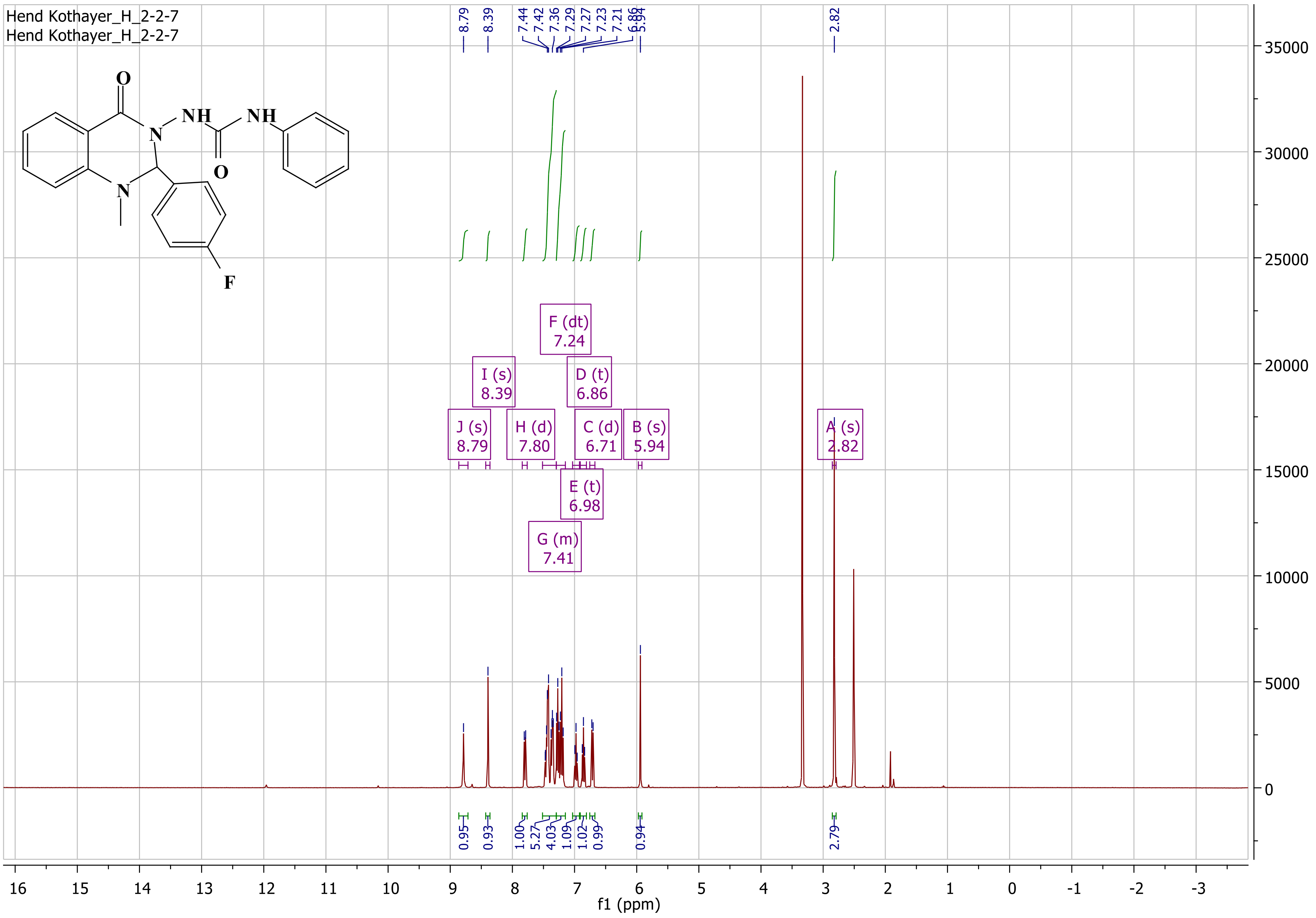

Hend Kothayer_H_2-2-7
Hend Kothayer_H_2-2-7
F (dt) 7.24
I (s) 8.39
D (t) 6.86
J (s) 8.79
H (d) 7.80
C (d) 6.71
B (s) 5.94
A (s) 2.82
E (t) 6.98
G (m) 7.41
f1 (ppm)

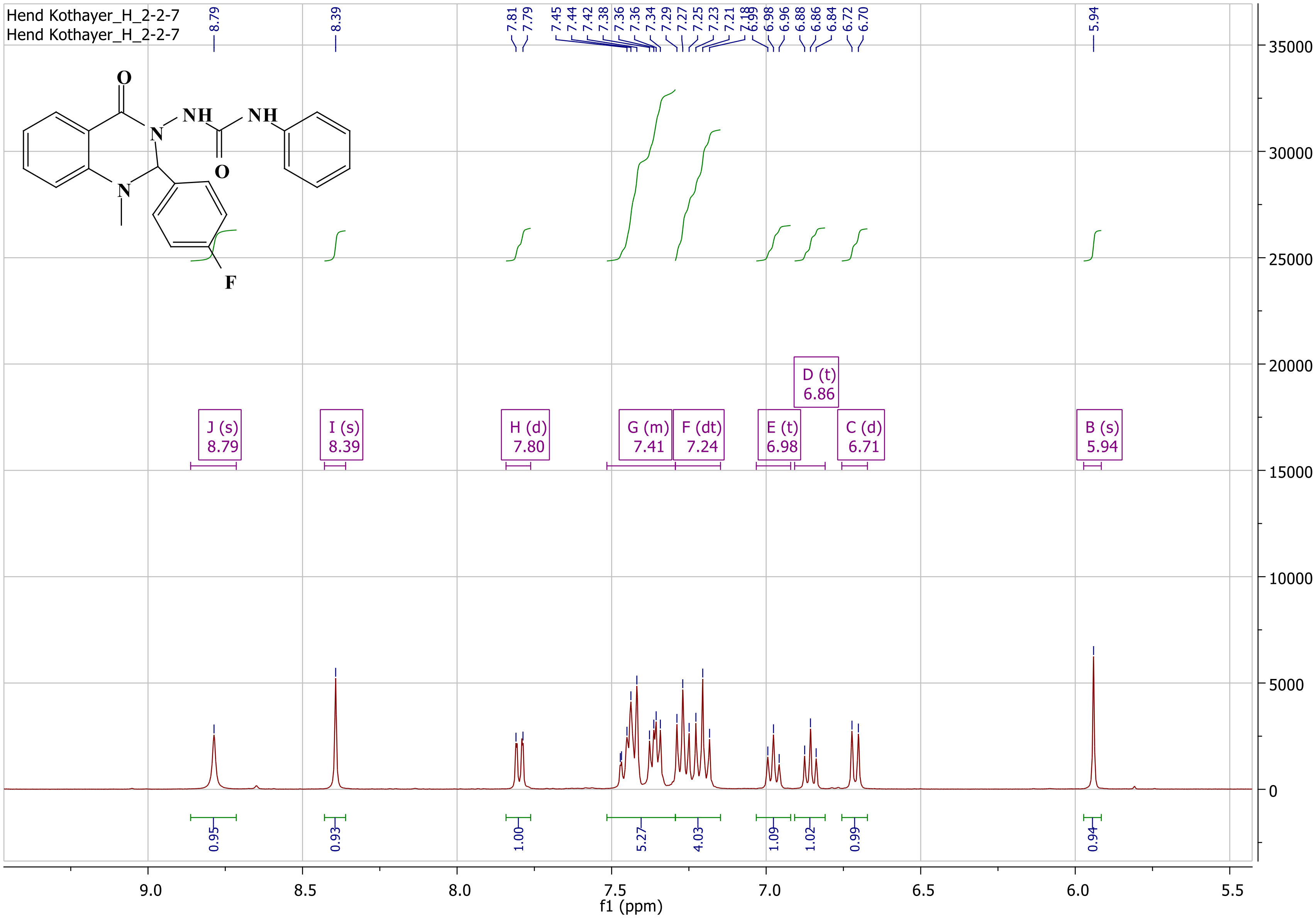

Hend Kothayer_H_2-2-7
Hend Kothayer_H_2-2-7
8.79
8.39
7.81
7.79
7.45
7.44
7.42
7.38
7.36
7.36
7.34
7.29
7.27
7.25
7.23
7.21
7.18
6.99
6.98
6.96
6.88
6.86
6.84
6.72
6.70
5.94
O
NH
NH
N
O
N
F
J (s) 8.79
I (s) 8.39
H (d) 7.80
G (m) 7.41
F (dt) 7.24
E (t) 6.98
D (t) 6.86
C (d) 6.71
B (s) 5.94
0.95
0.93
1.00
5.27
4.03
1.09
1.02
0.99
0.94
35000
30000
25000
20000
15000
10000
5000
0
9.0
8.5
8.0
7.5
7.0
6.5
6.0
5.5
f1 (ppm)

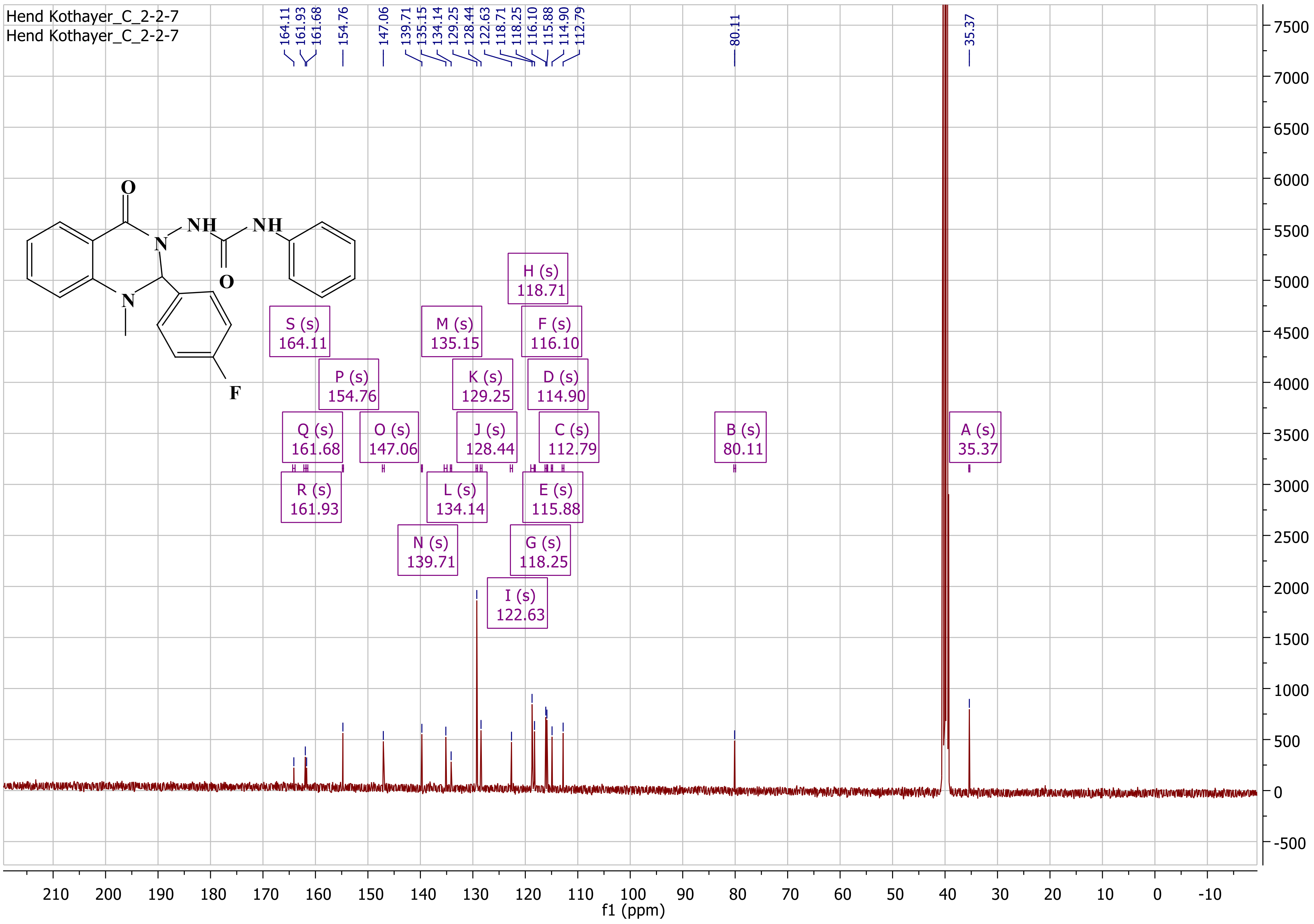
Hend Kothayer_C_2-2-7
Hend Kothayer_C_2-2-7
164.11
161.93
161.68
154.76
147.06
139.71
135.15
134.14
129.25
128.44
122.63
118.71
118.25
116.10
115.88
114.90
112.79
80.11
35.37
H (s) 118.71
S (s) 164.11
M (s) 135.15
F (s) 116.10
P (s) 154.76
K (s) 129.25
D (s) 114.90
Q (s) 161.68
O (s) 147.06
J (s) 128.44
C (s) 112.79
B (s) 80.11
A (s) 35.37
R (s) 161.93
L (s) 134.14
E (s) 115.88
N (s) 139.71
G (s) 118.25
I (s) 122.63
O
NH
NH
N
O
N
F
f1 (ppm)

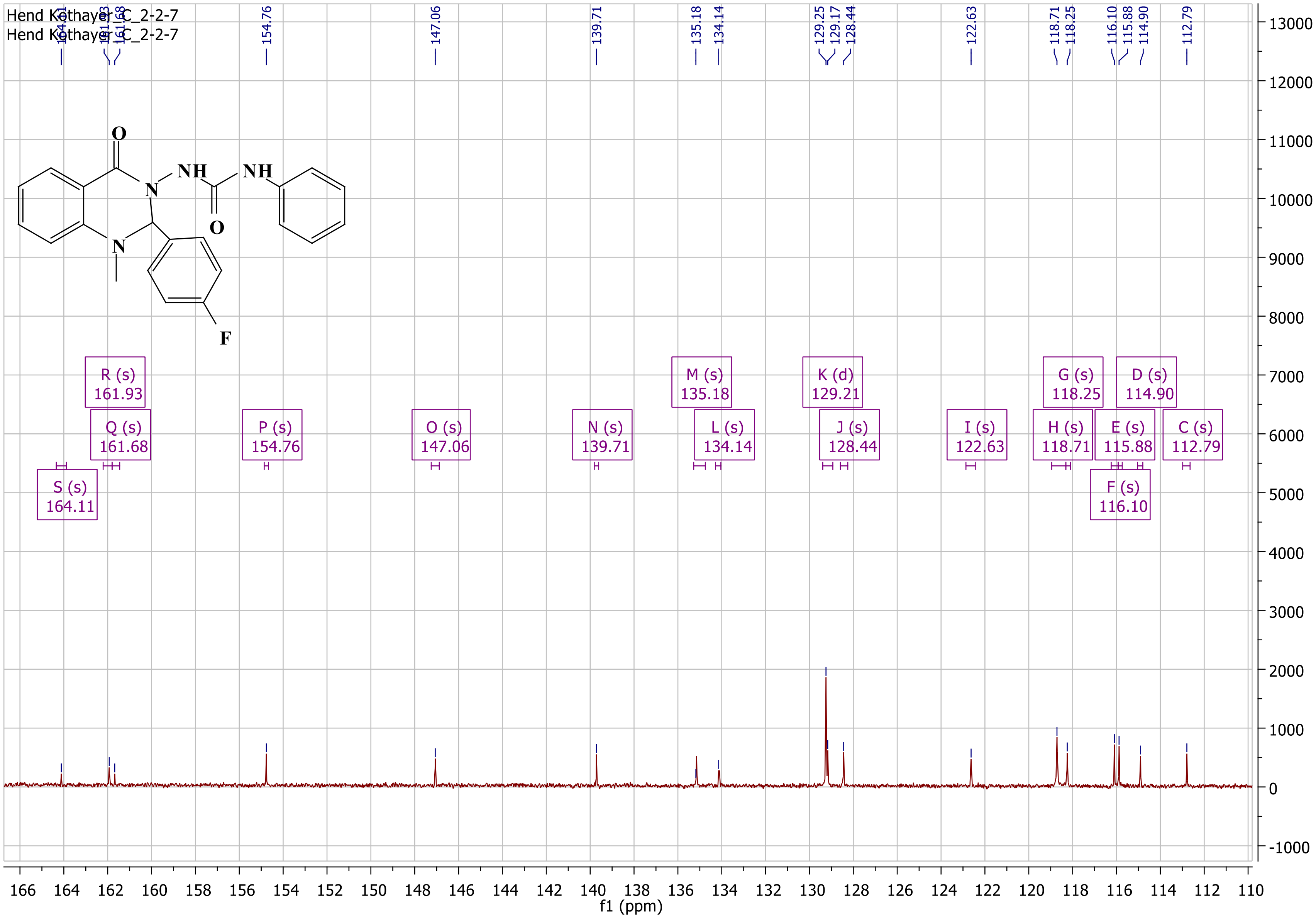
Hend Kothayer_C_2-2-7
164.11
161.93
161.68
154.76
147.06
139.71
135.18
134.14
129.25
129.17
128.44
122.63
118.71
118.25
116.10
115.88
114.90
112.79
O
NH
NH
N
N
O
F
R (s) 161.93
Q (s) 161.68
S (s) 164.11
P (s) 154.76
O (s) 147.06
N (s) 139.71
M (s) 135.18
L (s) 134.14
K (d) 129.21
J (s) 128.44
I (s) 122.63
G (s) 118.25
H (s) 118.71
D (s) 114.90
E (s) 115.88
F (s) 116.10
C (s) 112.79
f1 (ppm)

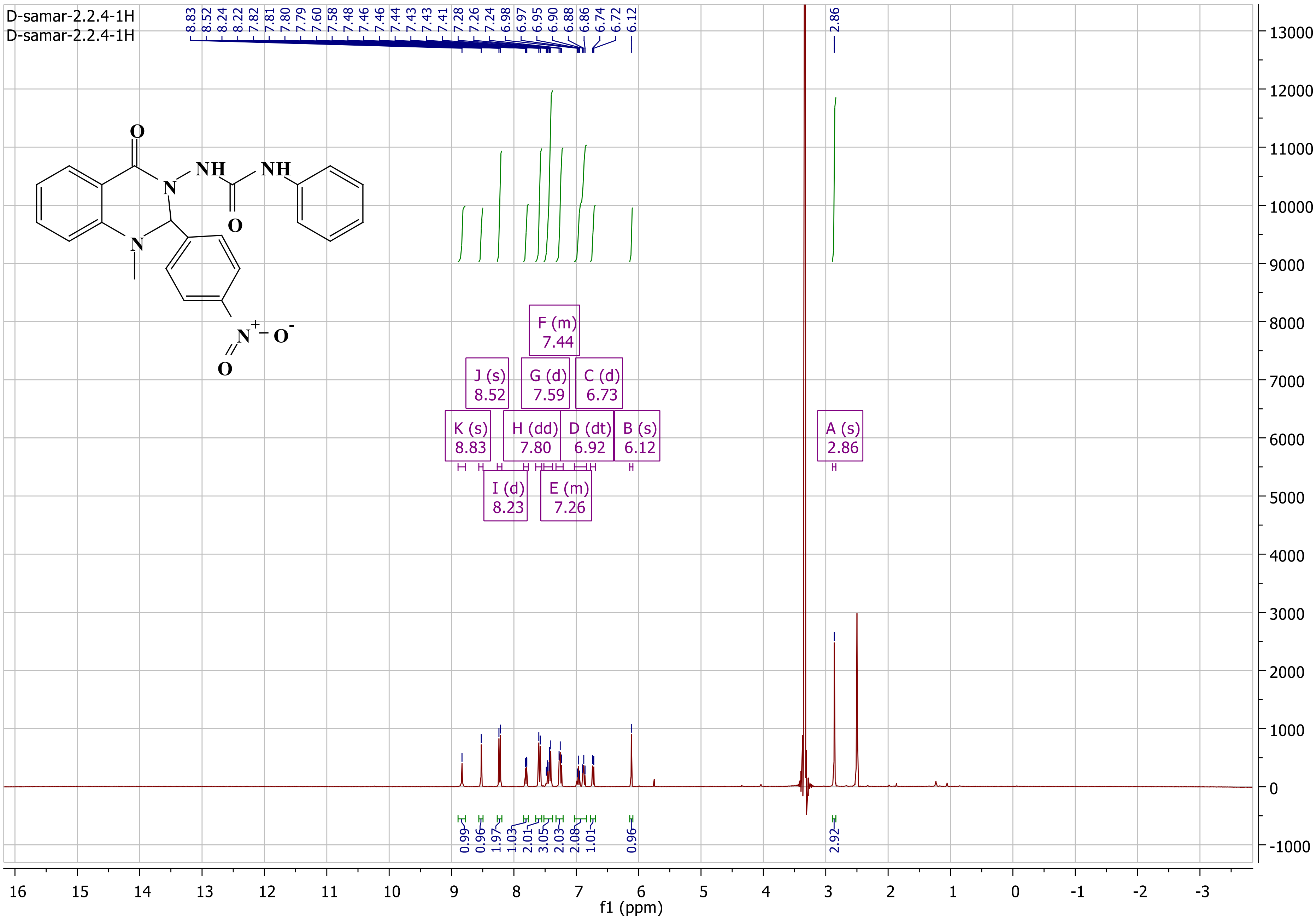
D-samar-2.2.4-1H
D-samar-2.2.4-1H
8.83 8.52 8.24 8.22 7.82 7.81 7.80 7.79 7.60 7.58 7.48 7.46 7.46 7.44 7.43 7.43 7.41 7.28 7.26 7.24 6.98 6.97 6.95 6.90 6.88 6.86 6.74 6.72 6.12
2.86
F (m) 7.44
J (s) 8.52
G (d) 7.59
C (d) 6.73
K (s) 8.83
H (dd) 7.80
D (dt) 6.92
B (s) 6.12
A (s) 2.86
I (d) 8.23
E (m) 7.26
0.99 0.96 1.97 1.03 2.01 3.05 2.03 2.08 1.01 0.96 2.92
f1 (ppm)

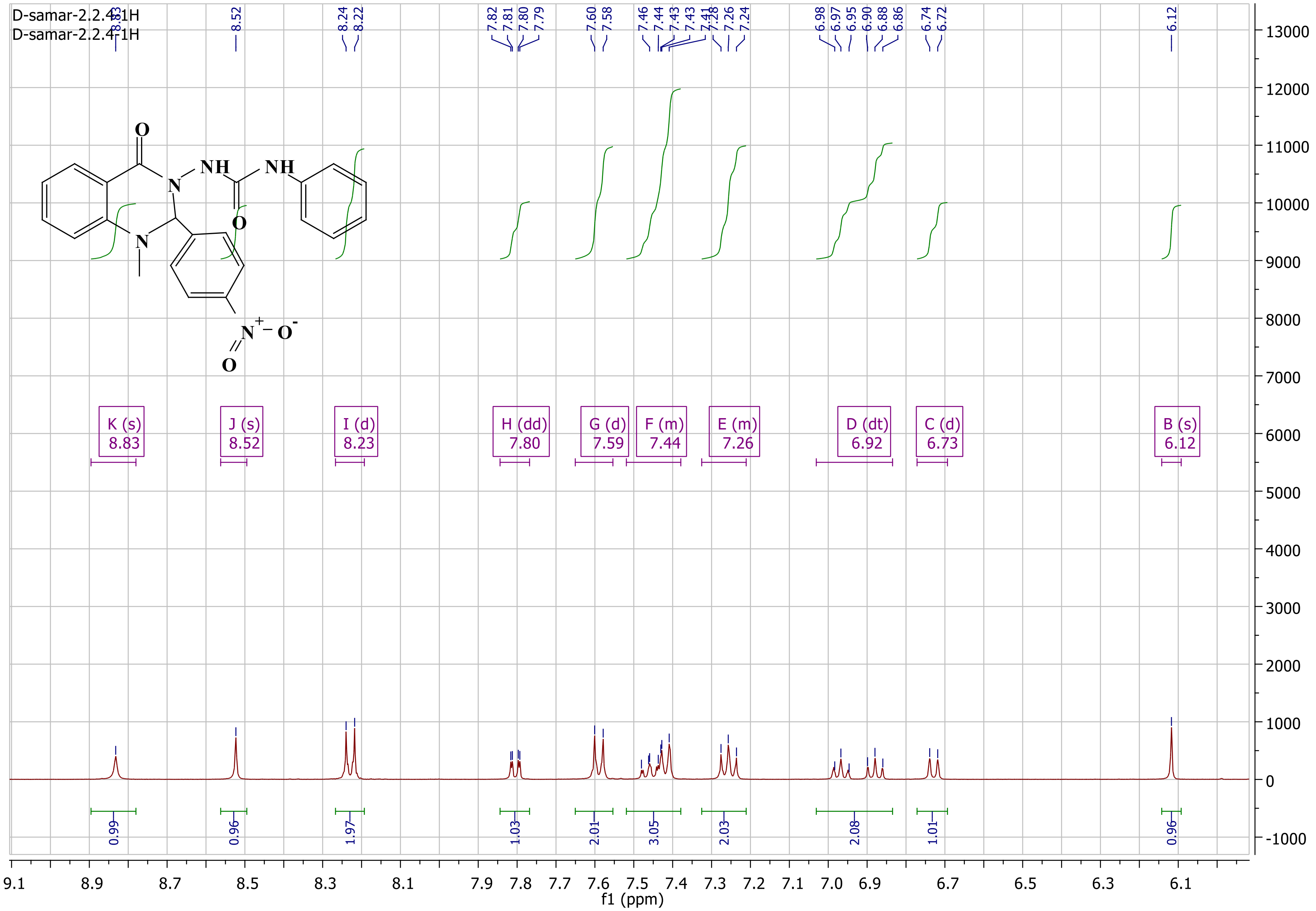

D-samar-2.2.4-1H
D-samar-2.2.4-1H
8.83
8.52
8.24
8.22
7.82
7.81
7.80
7.79
7.60
7.58
7.46
7.44
7.43
7.43
7.41
7.28
7.26
7.24
6.98
6.97
6.95
6.90
6.88
6.86
6.74
6.72
6.12
O
NH
NH
N
O
N
N
O
O
K (s) 8.83
J (s) 8.52
I (d) 8.23
H (dd) 7.80
G (d) 7.59
F (m) 7.44
E (m) 7.26
D (dt) 6.92
C (d) 6.73
B (s) 6.12
0.99
0.96
1.97
1.03
2.01
3.05
2.03
2.08
1.01
0.96
13000
12000
11000
10000
9000
8000
7000
6000
5000
4000
3000
2000
1000
0
-1000
9.1
8.9
8.7
8.5
8.3
8.1
7.9
7.8
7.7
7.6
7.5
7.4
7.3
7.2
7.1
7.0
6.9
6.7
6.5
6.3
6.1
f1 (ppm)

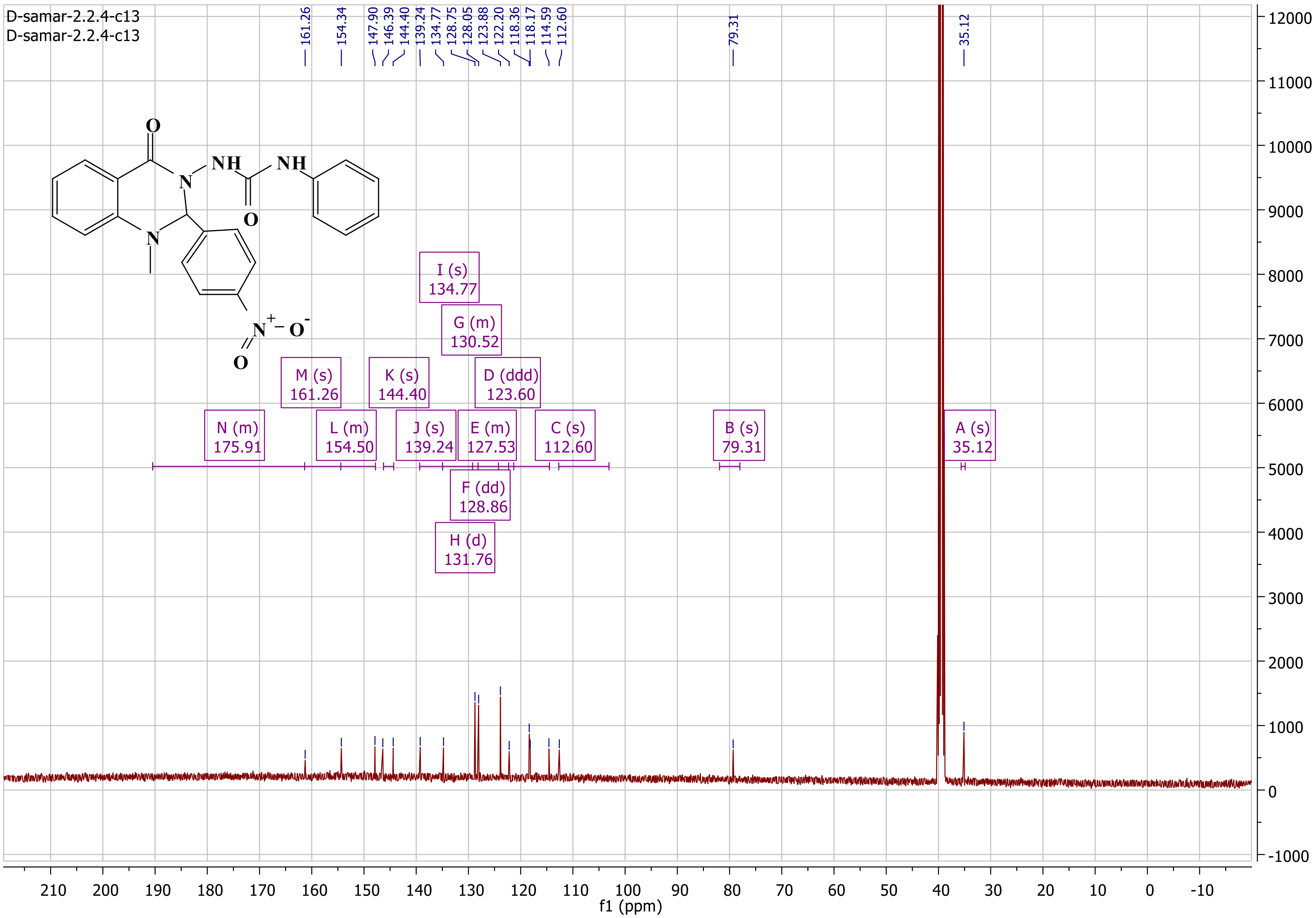
D-samar-2.2.4-c13
D-samar-2.2.4-c13
161.26
154.34
147.90
146.39
144.40
139.24
134.77
128.75
128.05
123.88
122.20
118.36
118.17
114.59
112.60
79.31
35.12
N (m) 175.91
M (s) 161.26
L (m) 154.50
K (s) 144.40
J (s) 139.24
I (s) 134.77
H (d) 131.76
G (m) 130.52
F (dd) 128.86
E (m) 127.53
D (ddd) 123.60
C (s) 112.60
B (s) 79.31
A (s) 35.12
O
NH
NH
N
O
N
N
O
O
f1 (ppm)

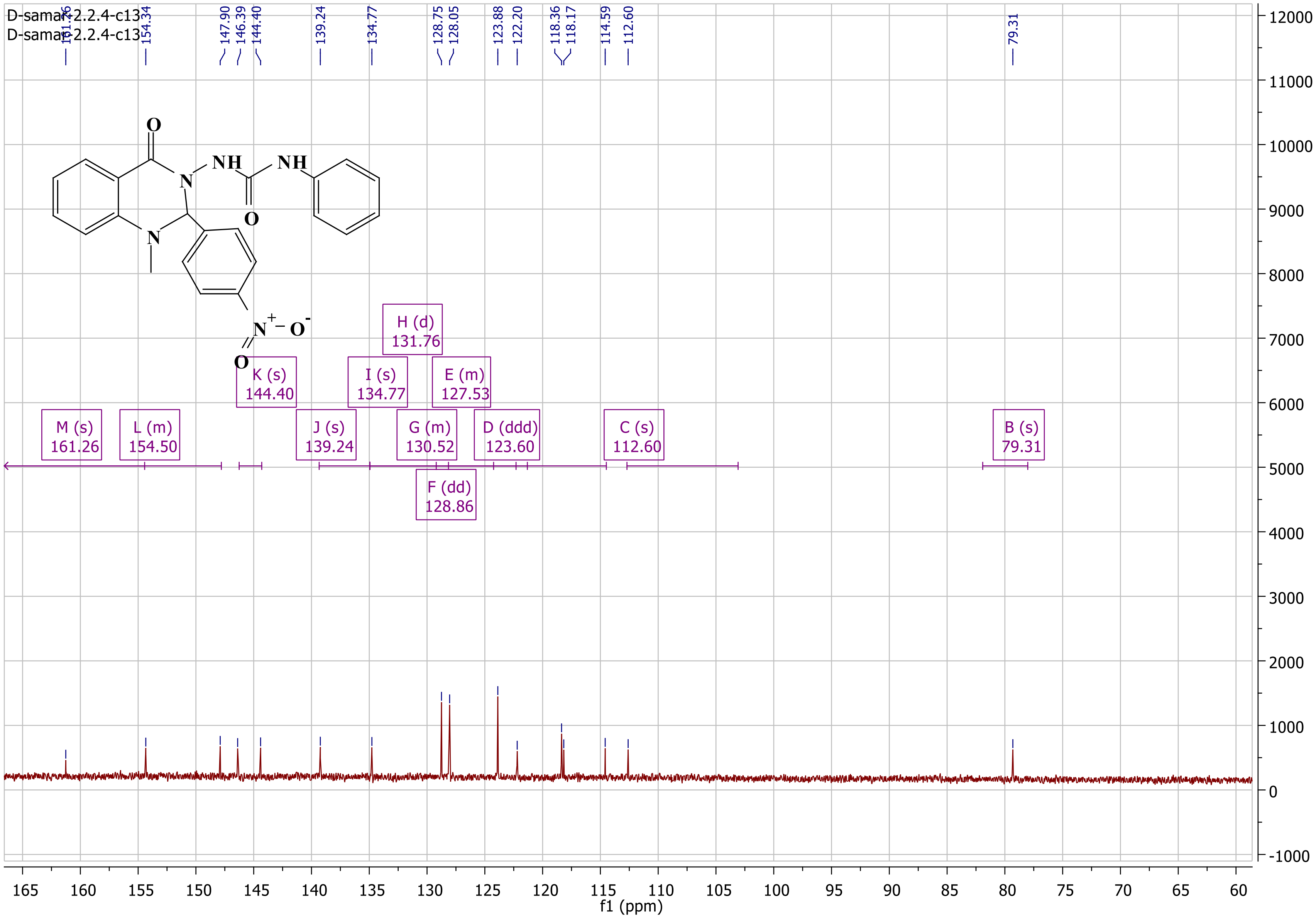
D-samar-2.2.4-c13
161.26
154.34
147.90
146.39
144.40
139.24
134.77
128.75
128.05
123.88
122.20
118.36
118.17
114.59
112.60
79.31
H (d) 131.76
K (s) 144.40
I (s) 134.77
E (m) 127.53
M (s) 161.26
L (m) 154.50
J (s) 139.24
G (m) 130.52
D (ddd) 123.60
C (s) 112.60
B (s) 79.31
F (dd) 128.86
f1 (ppm)

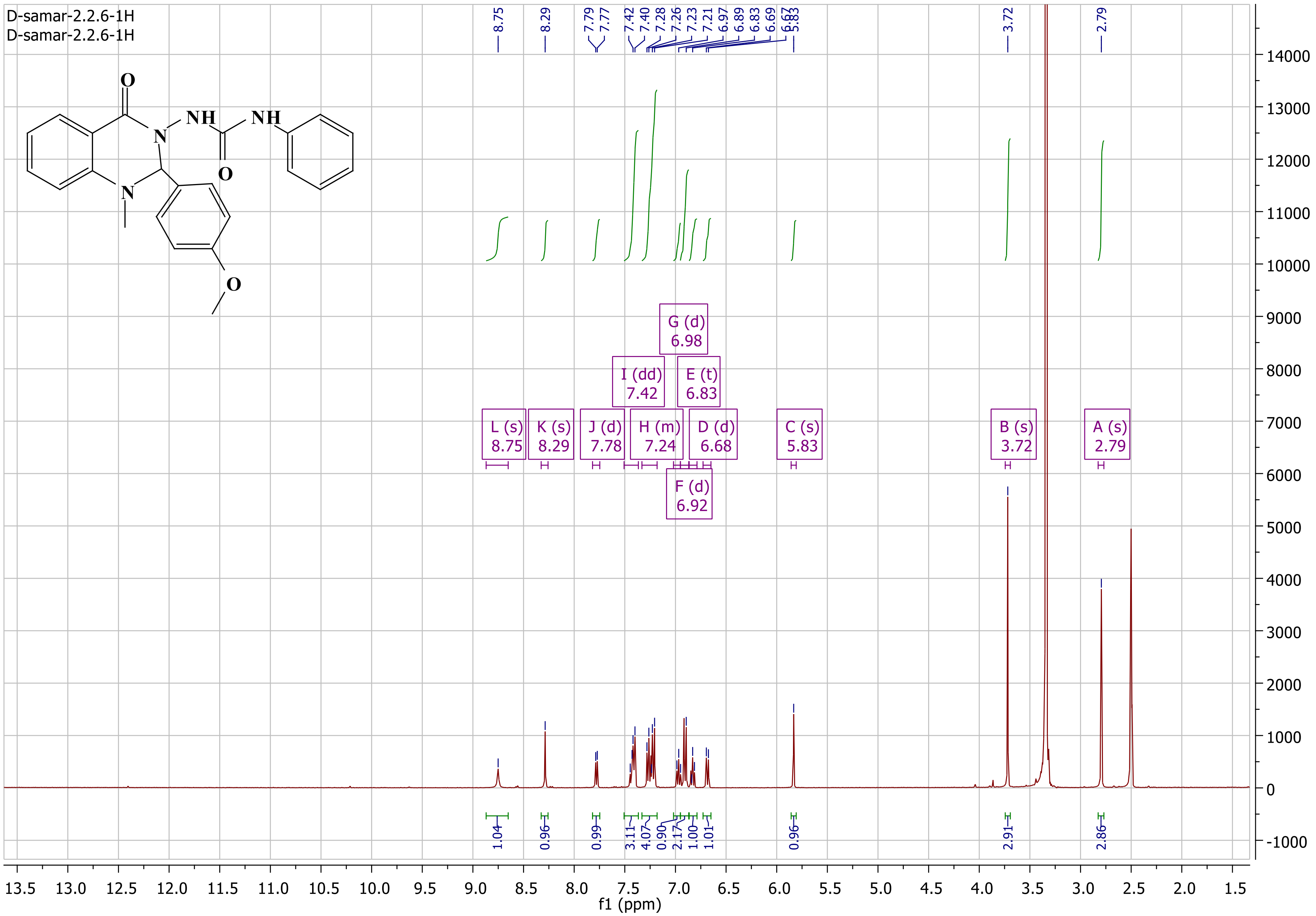

D-samar-2.2.6-1H
D-samar-2.2.6-1H
O
NH
NH
N
O
N
O
8.75
8.29
7.79
7.77
7.42
7.40
7.28
7.26
7.23
7.21
6.97
6.89
6.83
6.69
6.67
5.83
3.72
2.79
G (d) 6.98
I (dd) 7.42
E (t) 6.83
L (s) 8.75
K (s) 8.29
J (d) 7.78
H (m) 7.24
D (d) 6.68
C (s) 5.83
B (s) 3.72
A (s) 2.79
F (d) 6.92
1.04
0.96
0.99
3.11
4.07
0.90
2.17
1.00
1.01
0.96
2.91
2.86
f1 (ppm)

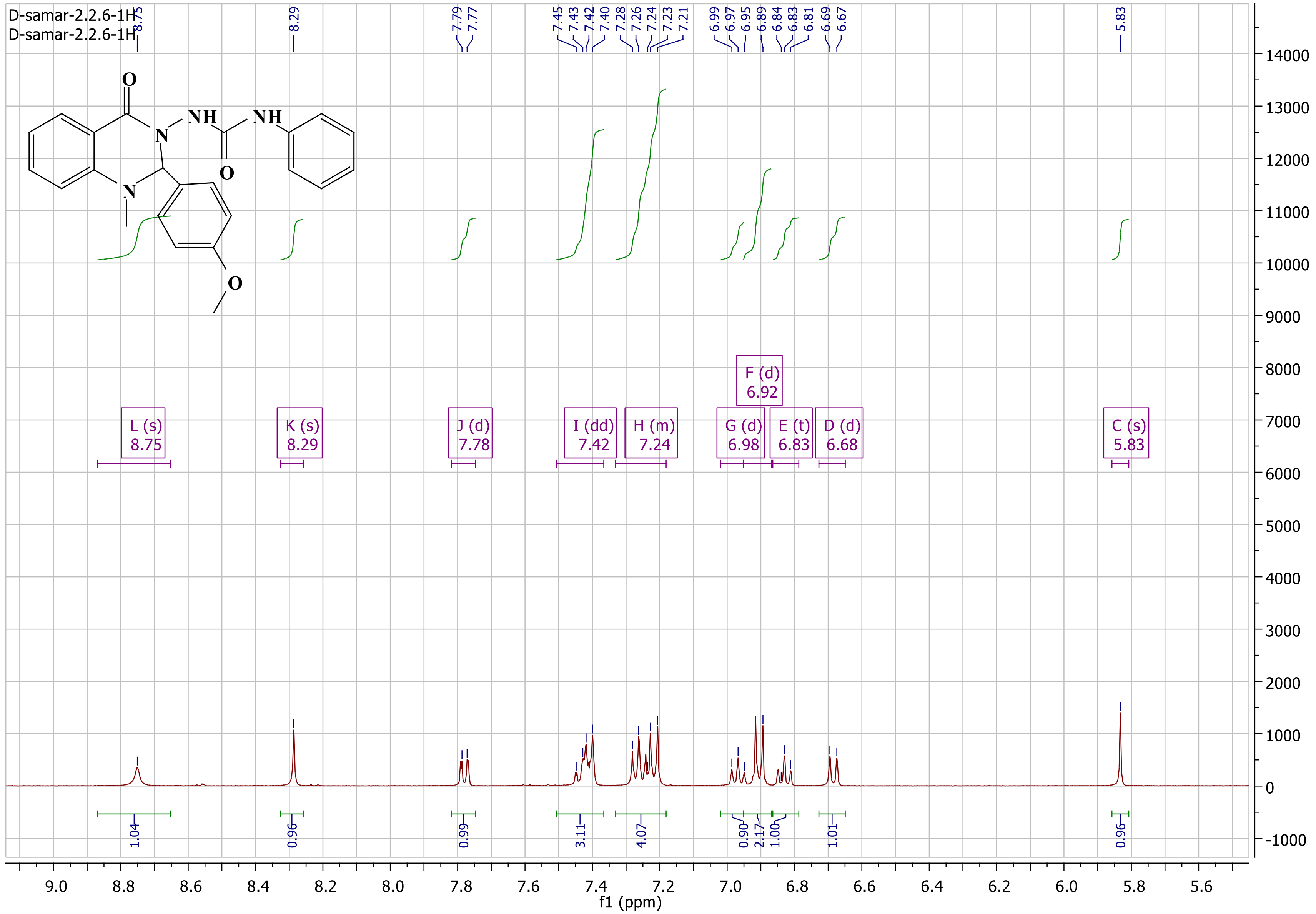

D-samar-2.2.6-1H
D-samar-2.2.6-1H
8.75
8.29
7.79
7.77
7.45
7.43
7.42
7.40
7.28
7.26
7.24
7.23
7.21
6.99
6.97
6.95
6.89
6.84
6.83
6.81
6.69
6.67
5.83
O
NH
NH
N
O
N
O
F (d) 6.92
L (s) 8.75
K (s) 8.29
J (d) 7.78
I (dd) 7.42
H (m) 7.24
G (d) 6.98
E (t) 6.83
D (d) 6.68
C (s) 5.83
1.04
0.96
0.99
3.11
4.07
0.90
2.17
1.00
1.01
0.96
f1 (ppm)

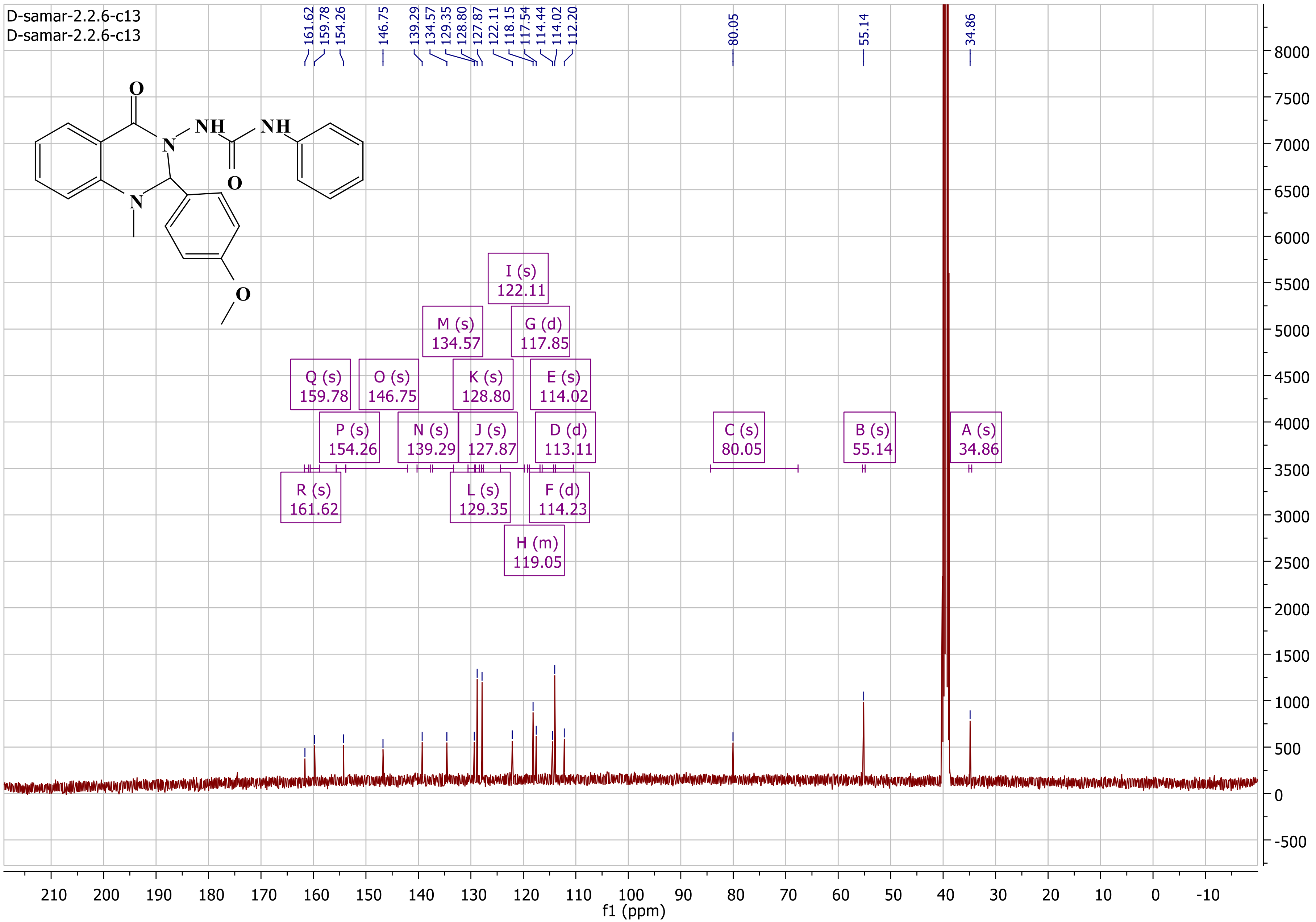
D-samar-2.2.6-c13
D-samar-2.2.6-c13
161.62
159.78
154.26
146.75
139.29
134.57
129.35
128.80
127.87
122.11
118.15
117.54
114.44
114.02
112.20
80.05
55.14
34.86
O
NH
NH
N
O
N
O
I (s) 122.11
M (s) 134.57
G (d) 117.85
Q (s) 159.78
O (s) 146.75
K (s) 128.80
E (s) 114.02
P (s) 154.26
N (s) 139.29
J (s) 127.87
D (d) 113.11
C (s) 80.05
B (s) 55.14
A (s) 34.86
R (s) 161.62
L (s) 129.35
F (d) 114.23
H (m) 119.05
f1 (ppm)

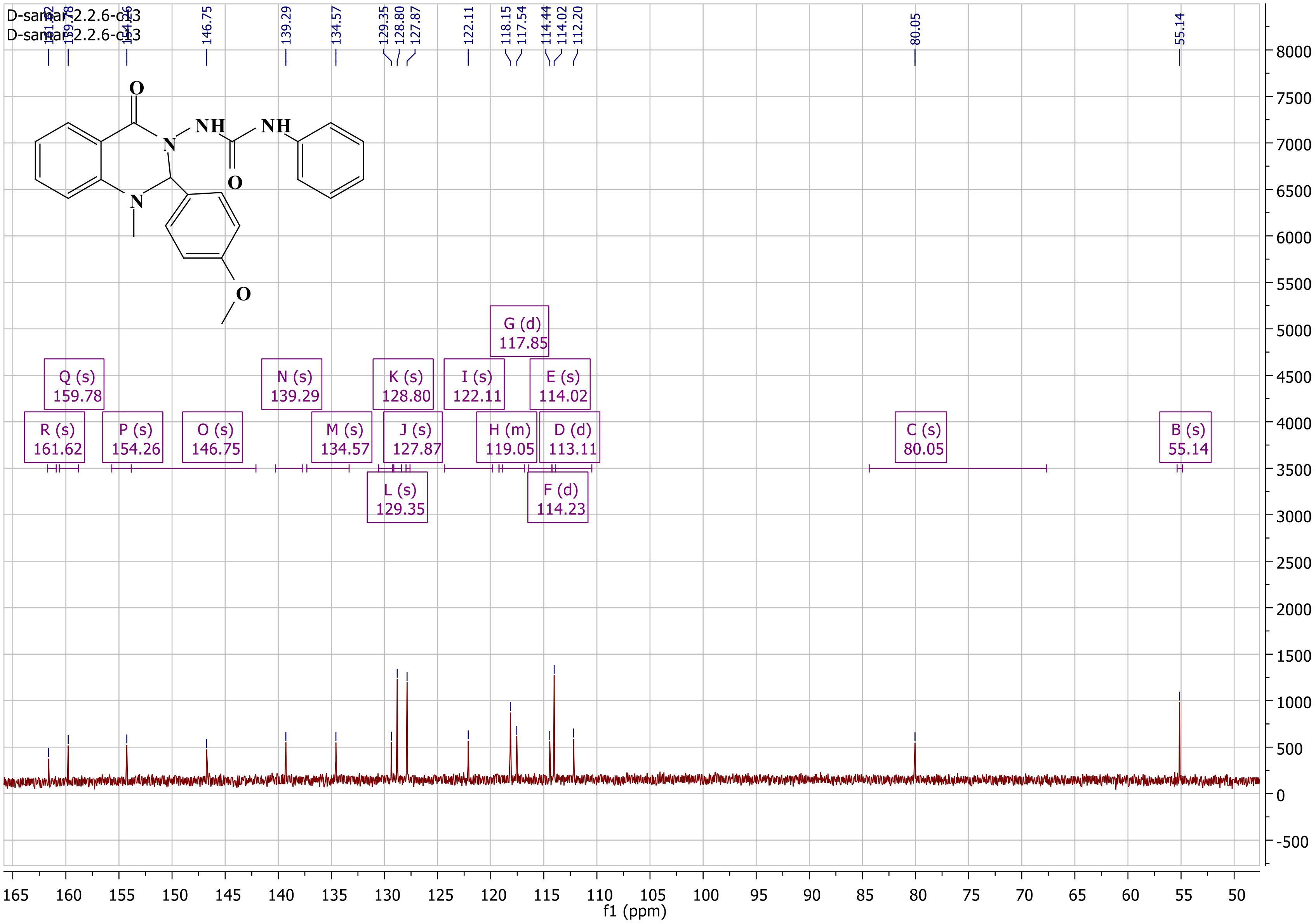
D-samar-2.2.6-c13
D-samar-2.2.6-c13
161.62
159.78
154.26
146.75
139.29
134.57
129.35
128.80
127.87
122.11
118.15
117.54
114.44
114.02
112.20
80.05
55.14
G (d) 117.85
Q (s) 159.78
N (s) 139.29
K (s) 128.80
I (s) 122.11
E (s) 114.02
R (s) 161.62
P (s) 154.26
O (s) 146.75
M (s) 134.57
J (s) 127.87
H (m) 119.05
D (d) 113.11
C (s) 80.05
B (s) 55.14
L (s) 129.35
F (d) 114.23
f1 (ppm)

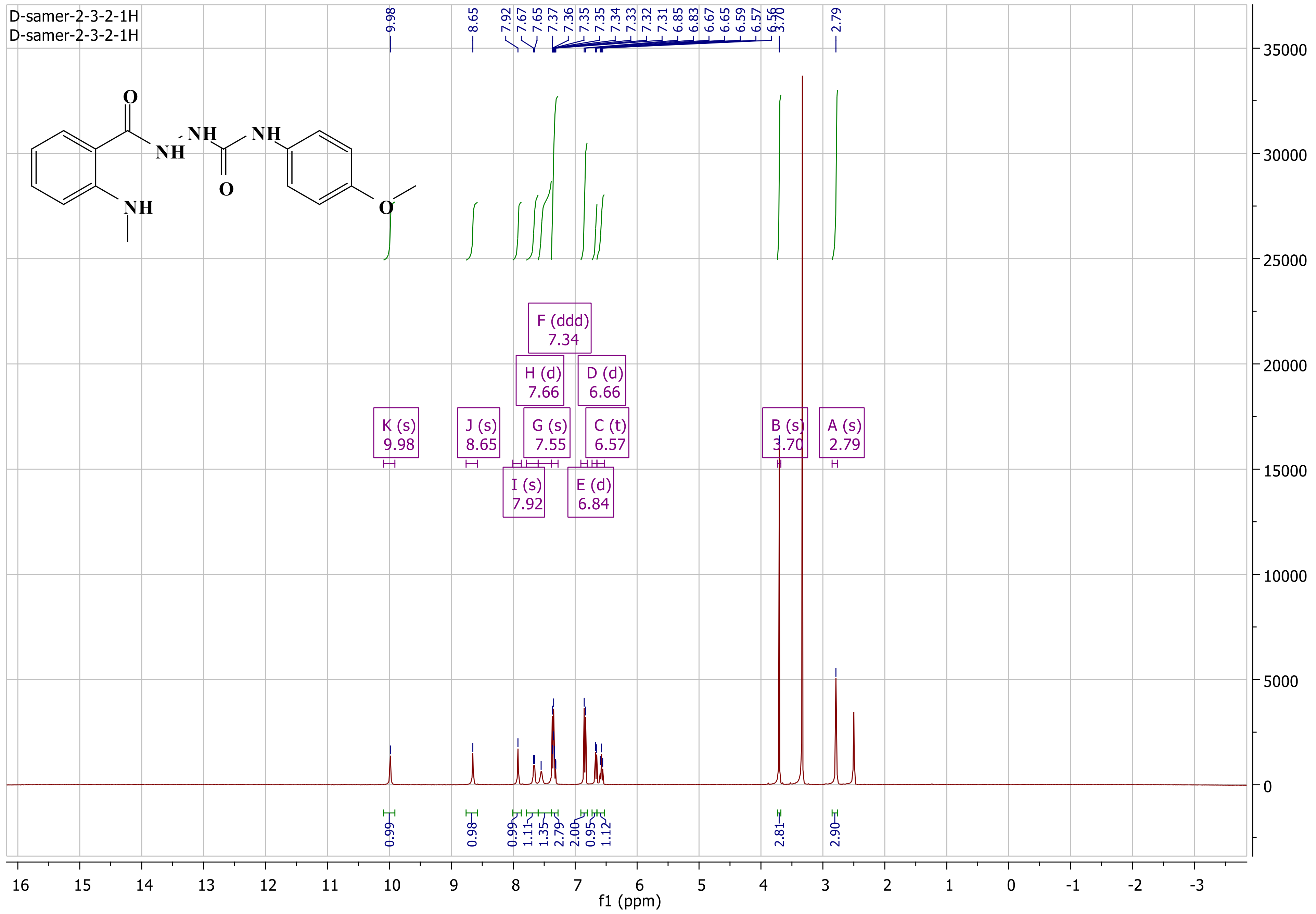
D-samer-2-3-2-1H
D-samer-2-3-2-1H
9.98
8.65
7.92
7.67
7.65
7.37
7.36
7.35
7.35
7.34
7.33
7.32
7.31
6.85
6.83
6.67
6.65
6.59
6.57
6.56
3.70
2.79
O
NH
NH
NH
O
NH
O
F (ddd) 7.34
H (d) 7.66
D (d) 6.66
K (s) 9.98
J (s) 8.65
G (s) 7.55
C (t) 6.57
B (s) 3.70
A (s) 2.79
I (s) 7.92
E (d) 6.84
0.99
0.98
0.99
1.11
1.35
2.79
2.00
0.95
1.12
2.81
2.90
f1 (ppm)

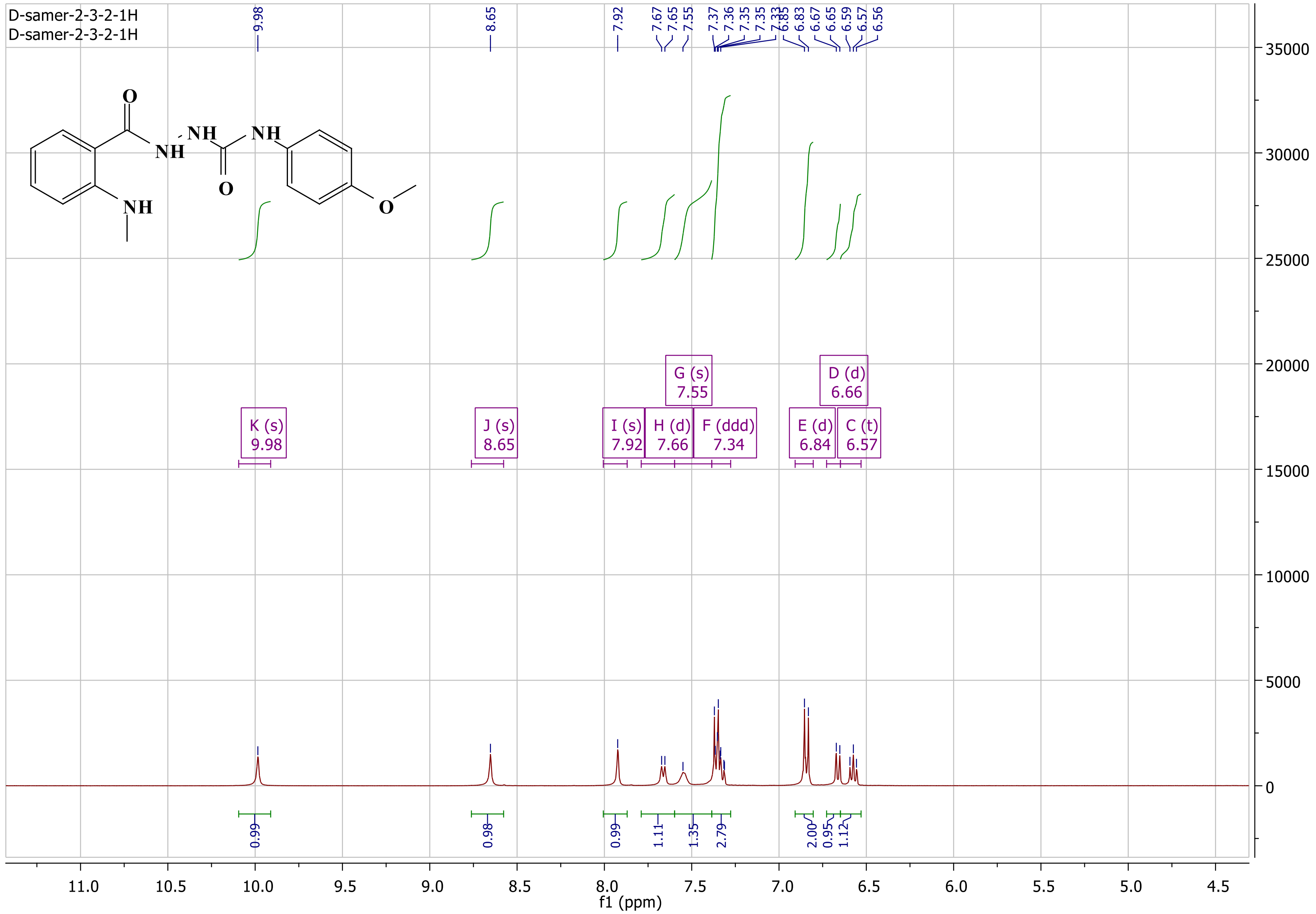

D-samer-2-3-2-1H
D-samer-2-3-2-1H
9.98
8.65
7.92
7.67
7.65
7.55
7.37
7.36
7.35
7.35
7.33
6.85
6.83
6.67
6.65
6.59
6.57
6.56
O
NH
NH
NH
O
NH
O
K (s) 9.98
J (s) 8.65
I (s) 7.92
H (d) 7.66
G (s) 7.55
F (ddd) 7.34
E (d) 6.84
D (d) 6.66
C (t) 6.57
0.99
0.98
0.99
1.11
1.35
2.79
2.00
0.95
1.12
f1 (ppm)

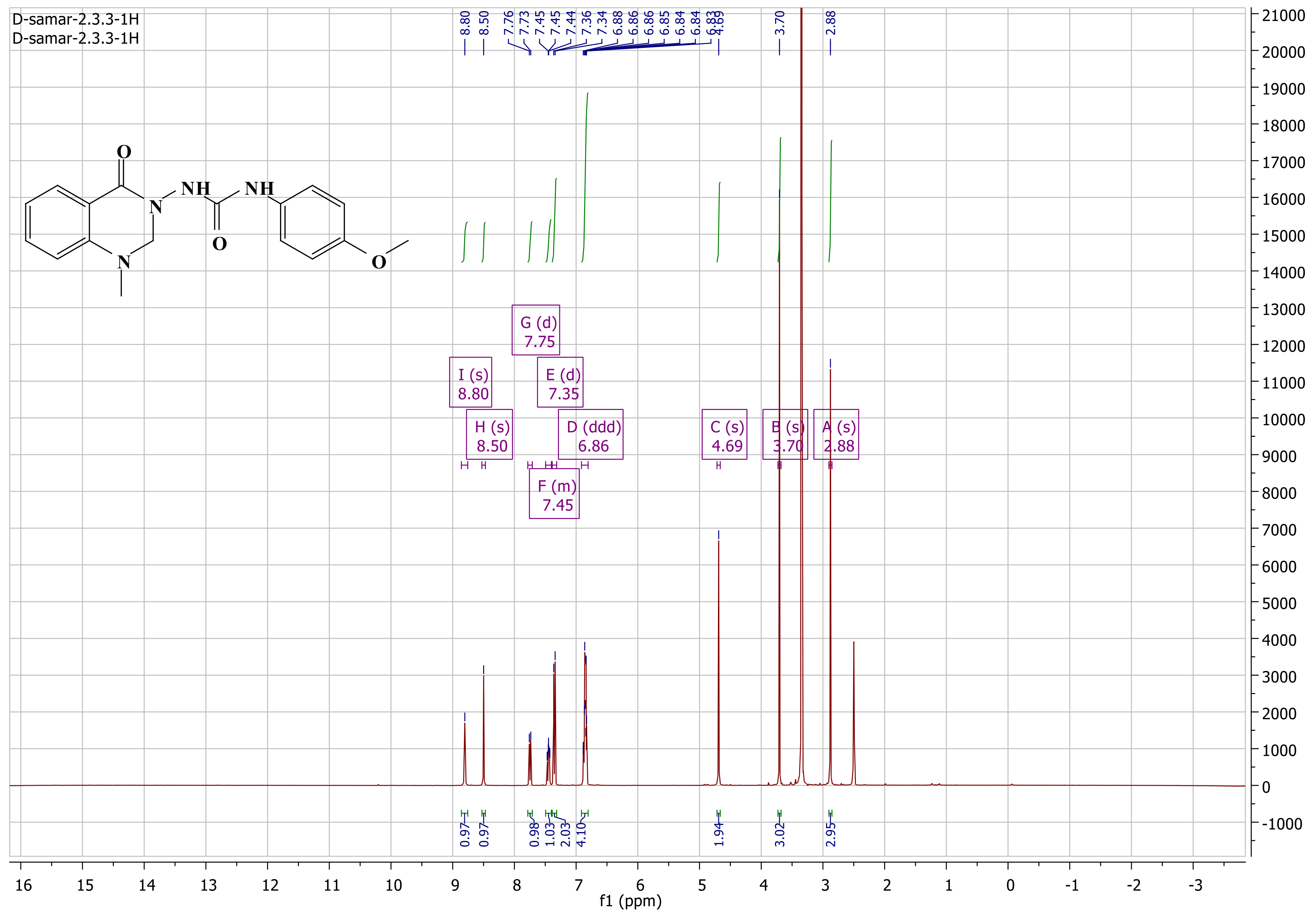
D-samar-2.3.3-1H
D-samar-2.3.3-1H
8.80
8.50
7.76
7.73
7.45
7.45
7.44
7.36
7.34
6.88
6.86
6.86
6.85
6.84
6.84
6.83
4.69
3.70
2.88
O
NH
NH
N
O
N
O
G (d)
7.75
I (s)
8.80
E (d)
7.35
H (s)
8.50
D (ddd)
6.86
C (s)
4.69
B (s)
3.70
A (s)
2.88
F (m)
7.45
0.97
0.97
0.98
1.03
2.03
4.10
1.94
3.02
2.95
f1 (ppm)

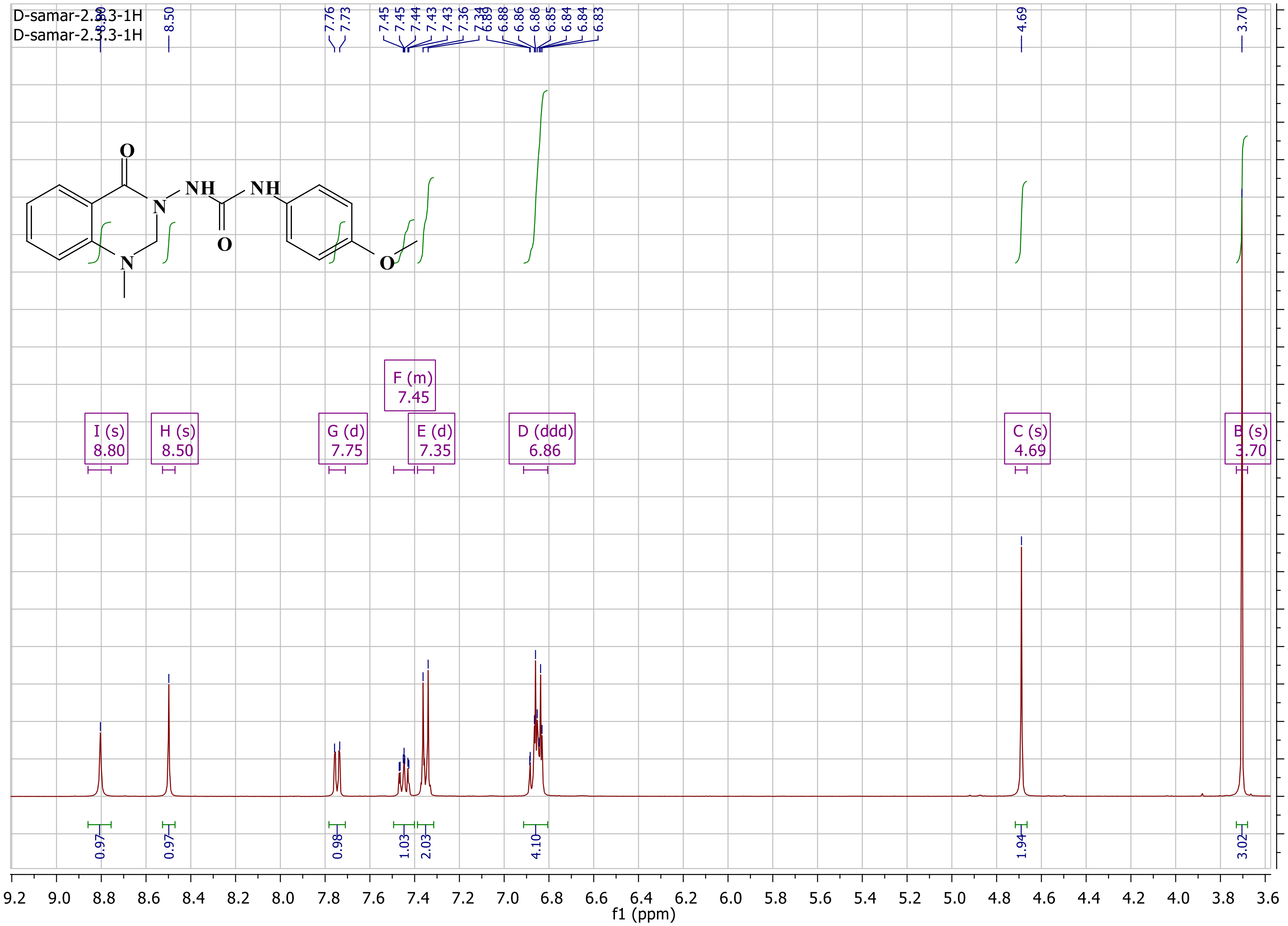
D-samar-2.3.3-1H
D-samar-2.3.3-1H
8.80
8.50
7.76
7.73
7.45
7.45
7.44
7.43
7.43
7.36
7.34
6.89
6.88
6.86
6.86
6.85
6.84
6.84
6.83
4.69
3.70
F (m)
7.45
I (s)
8.80
H (s)
8.50
G (d)
7.75
E (d)
7.35
D (ddd)
6.86
C (s)
4.69
B (s)
3.70
0.97
0.97
0.98
1.03
2.03
4.10
1.94
3.02
f1 (ppm)

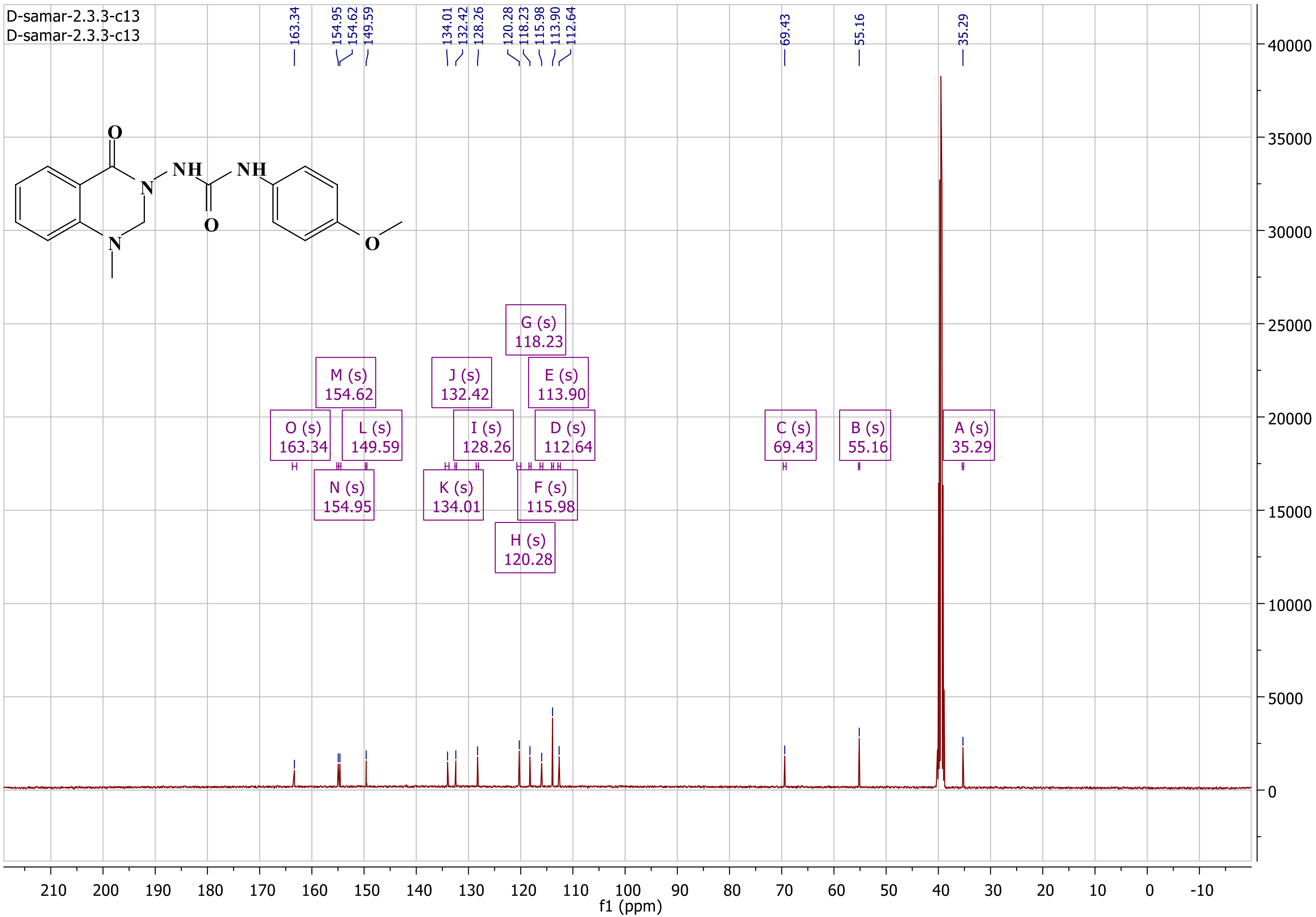
D-samar-2.3.3-c13
D-samar-2.3.3-c13
163.34
154.95
154.62
149.59
134.01
132.42
128.26
120.28
118.23
115.98
113.90
112.64
69.43
55.16
35.29
O
NH
NH
N
N
O
O
G (s) 118.23
M (s) 154.62
J (s) 132.42
E (s) 113.90
O (s) 163.34
L (s) 149.59
I (s) 128.26
D (s) 112.64
C (s) 69.43
B (s) 55.16
A (s) 35.29
N (s) 154.95
K (s) 134.01
F (s) 115.98
H (s) 120.28
f1 (ppm)

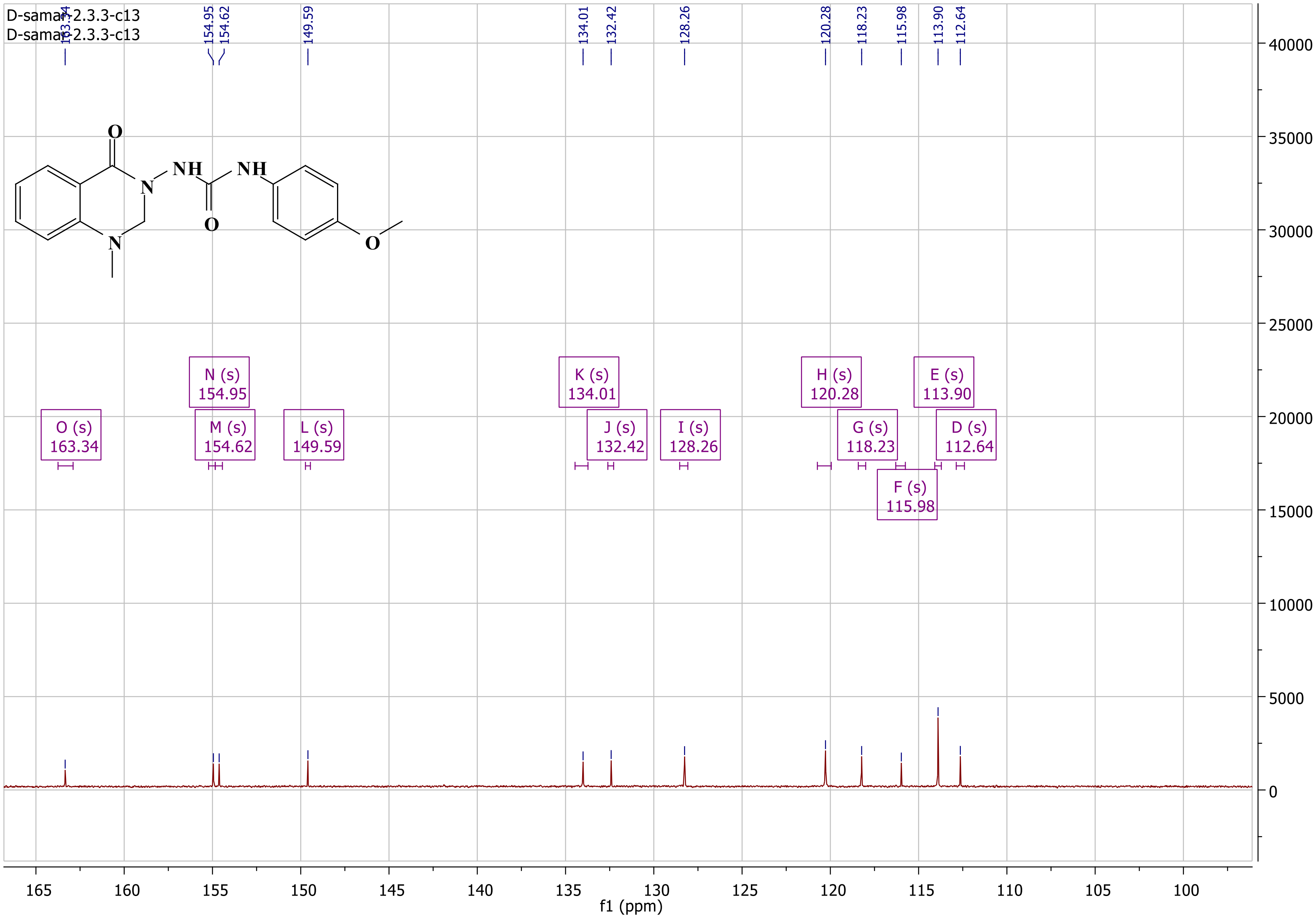
D-samar-2.3.3-c13
D-samar-2.3.3-c13
163.34
154.95
154.62
149.59
134.01
132.42
128.26
120.28
118.23
115.98
113.90
112.64
O
NH
NH
N
N
O
O
N (s) 154.95
K (s) 134.01
H (s) 120.28
E (s) 113.90
O (s) 163.34
M (s) 154.62
L (s) 149.59
J (s) 132.42
I (s) 128.26
G (s) 118.23
D (s) 112.64
F (s) 115.98
165
160
155
150
145
140
135
130
125
120
115
110
105
100
f1 (ppm)
40000
35000
30000
25000
20000
15000
10000
5000
0

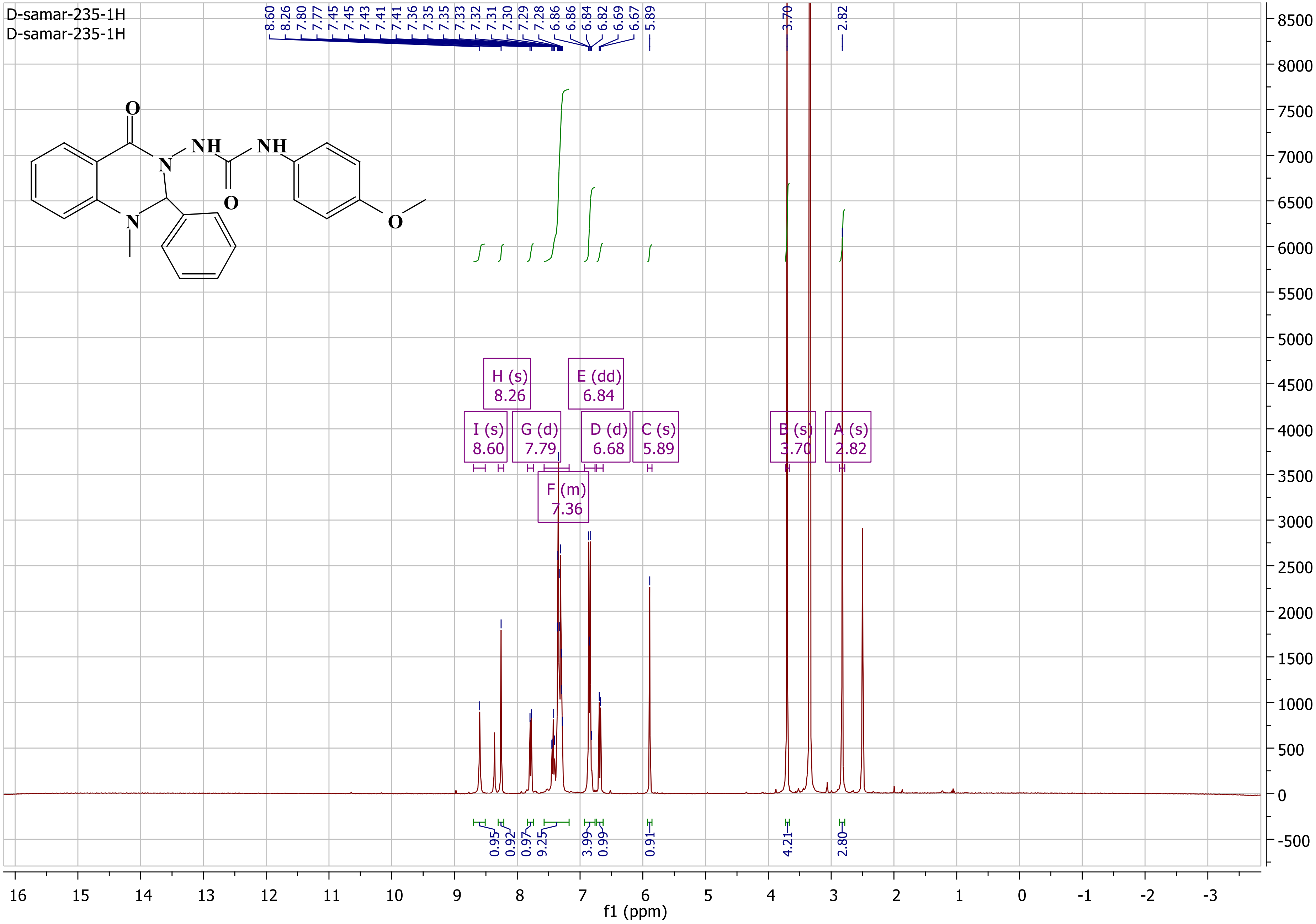
D-samar-235-1H
D-samar-235-1H
8.60
8.26
7.80
7.77
7.45
7.45
7.43
7.41
7.41
7.36
7.35
7.35
7.33
7.32
7.31
7.30
7.29
7.28
6.86
6.86
6.84
6.82
6.69
6.67
5.89
3.70
2.82
H (s) 8.26
E (dd) 6.84
I (s) 8.60
G (d) 7.79
D (d) 6.68
C (s) 5.89
B (s) 3.70
A (s) 2.82
F (m) 7.36
0.95
0.92
0.97
9.25
3.99
0.99
0.91
4.21
2.80
f1 (ppm)
O
NH
NH
N
N
O
O

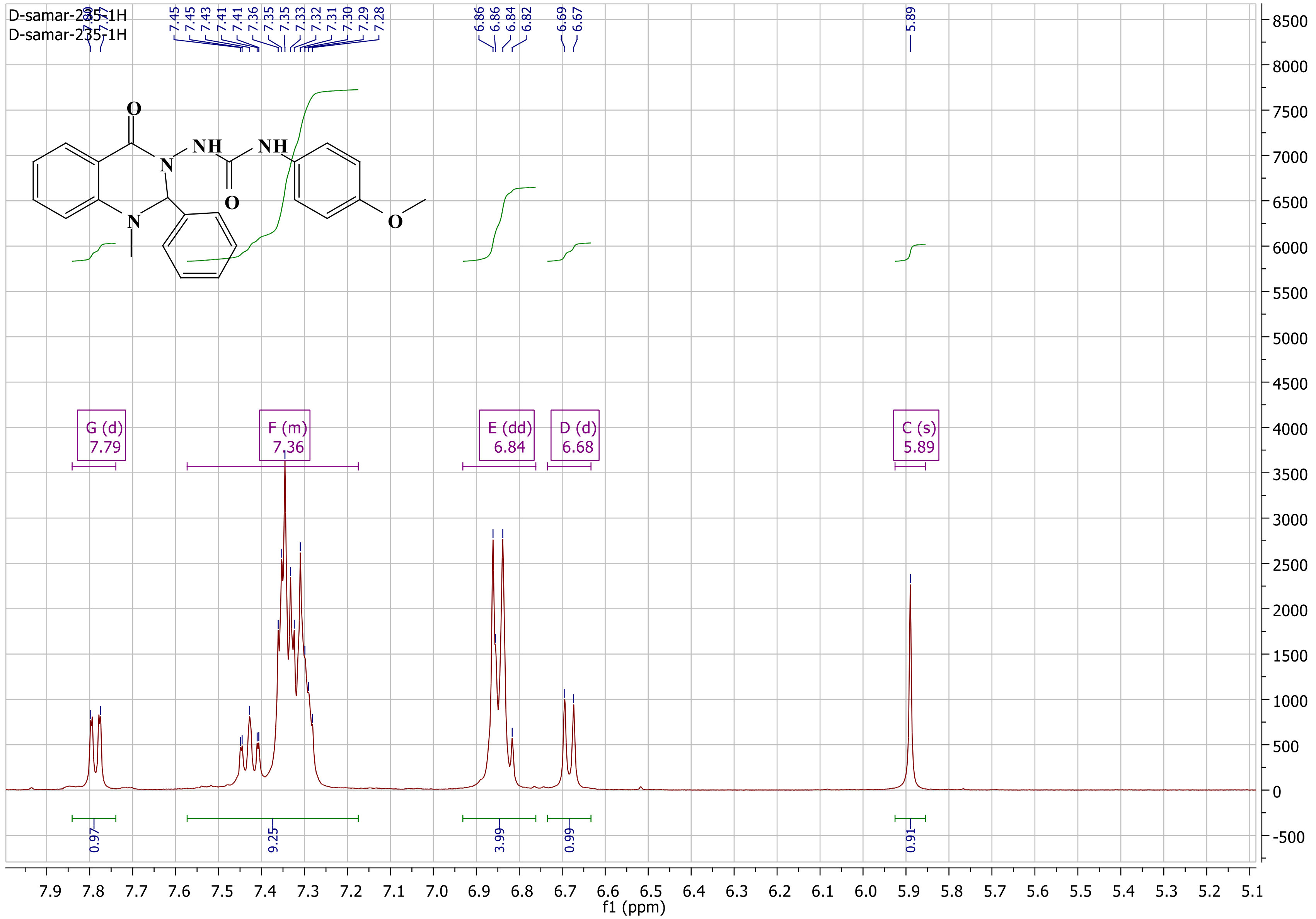

D-samar-235-1H
D-samar-235-1H
7.45
7.45
7.43
7.41
7.41
7.36
7.35
7.35
7.33
7.32
7.31
7.30
7.29
7.28
6.86
6.86
6.84
6.82
6.69
6.67
5.89
G (d) 7.79
F (m) 7.36
E (dd) 6.84
D (d) 6.68
C (s) 5.89
0.97
9.25
3.99
0.99
0.91
f1 (ppm)

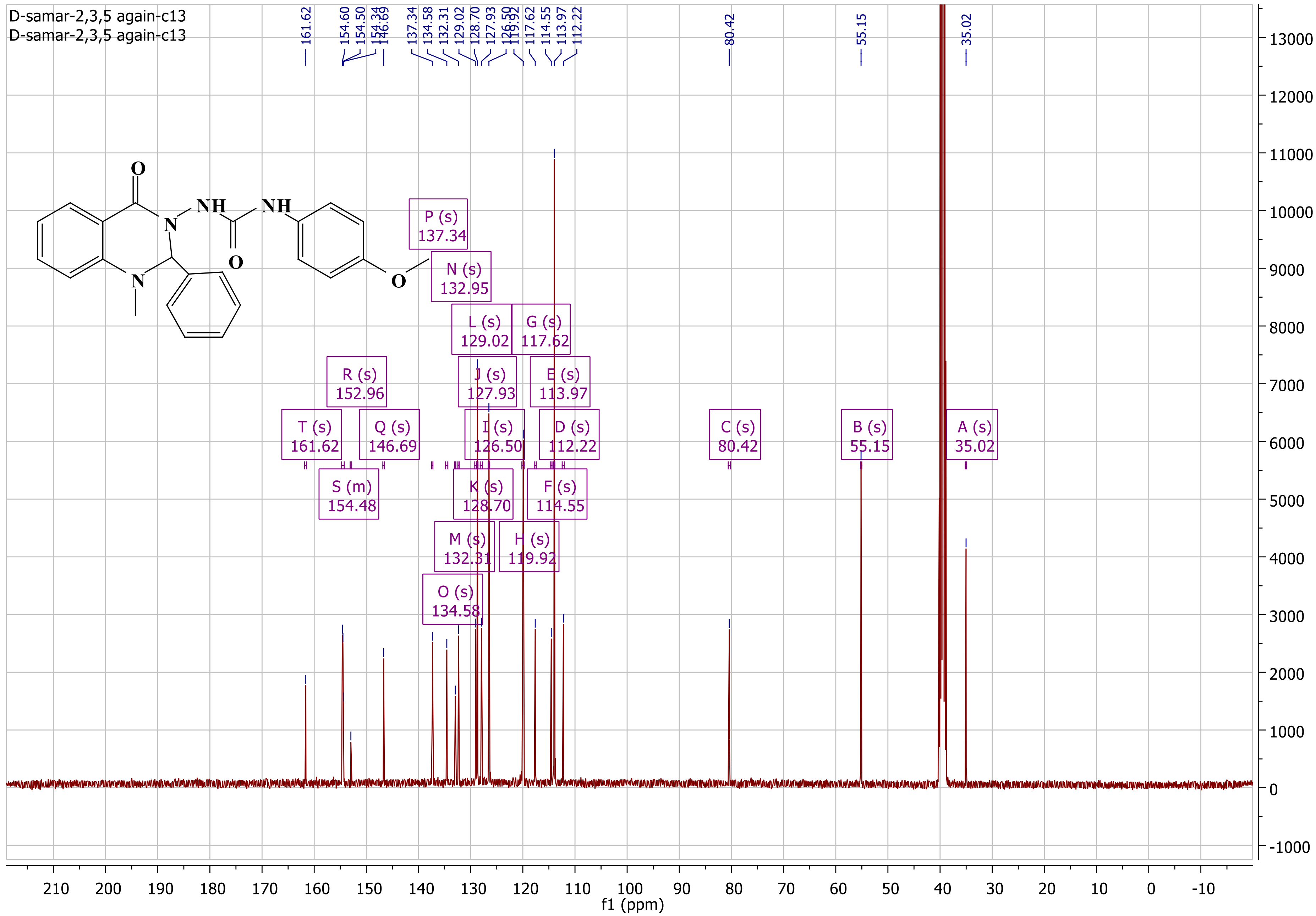

D-samar-2,3,5 again-c13
D-samar-2,3,5 again-c13
161.62
154.60
154.50
154.34
146.69
137.34
134.58
132.31
129.02
128.70
127.93
126.50
119.92
117.62
114.55
113.97
112.22
80.42
55.15
35.02
O
NH
NH
N
O
N
O
P (s) 137.34
N (s) 132.95
L (s) 129.02
G (s) 117.62
R (s) 152.96
J (s) 127.93
E (s) 113.97
T (s) 161.62
Q (s) 146.69
I (s) 126.50
D (s) 112.22
C (s) 80.42
B (s) 55.15
A (s) 35.02
S (m) 154.48
K (s) 128.70
F (s) 114.55
M (s) 132.31
H (s) 119.92
O (s) 134.58
13000
12000
11000
10000
9000
8000
7000
6000
5000
4000
3000
2000
1000
0
-1000
210
200
190
180
170
160
150
140
130
120
110
100
90
80
70
60
50
40
30
20
10
0
-10
f1 (ppm)

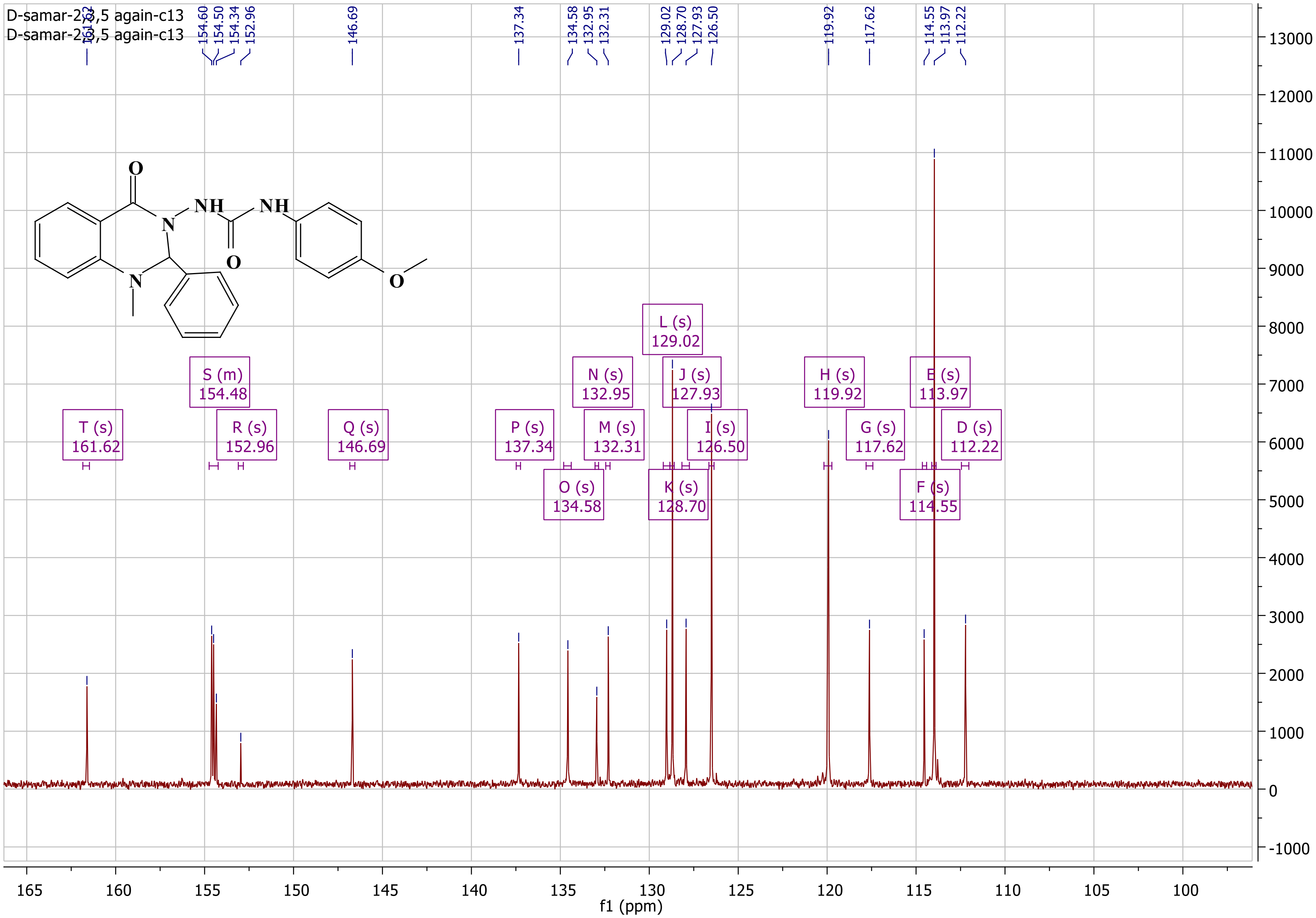
161.62
154.60
154.50
154.34
152.96
146.69
137.34
134.58
132.95
132.31
129.02
128.70
127.93
126.50
119.92
117.62
114.55
113.97
112.22
T (s) 161.62
S (m) 154.48
R (s) 152.96
Q (s) 146.69
P (s) 137.34
O (s) 134.58
N (s) 132.95
M (s) 132.31
L (s) 129.02
K (s) 128.70
J (s) 127.93
I (s) 126.50
H (s) 119.92
G (s) 117.62
F (s) 114.55
E (s) 113.97
D (s) 112.22
O
NH
NH
N
N
O
O
f1 (ppm)

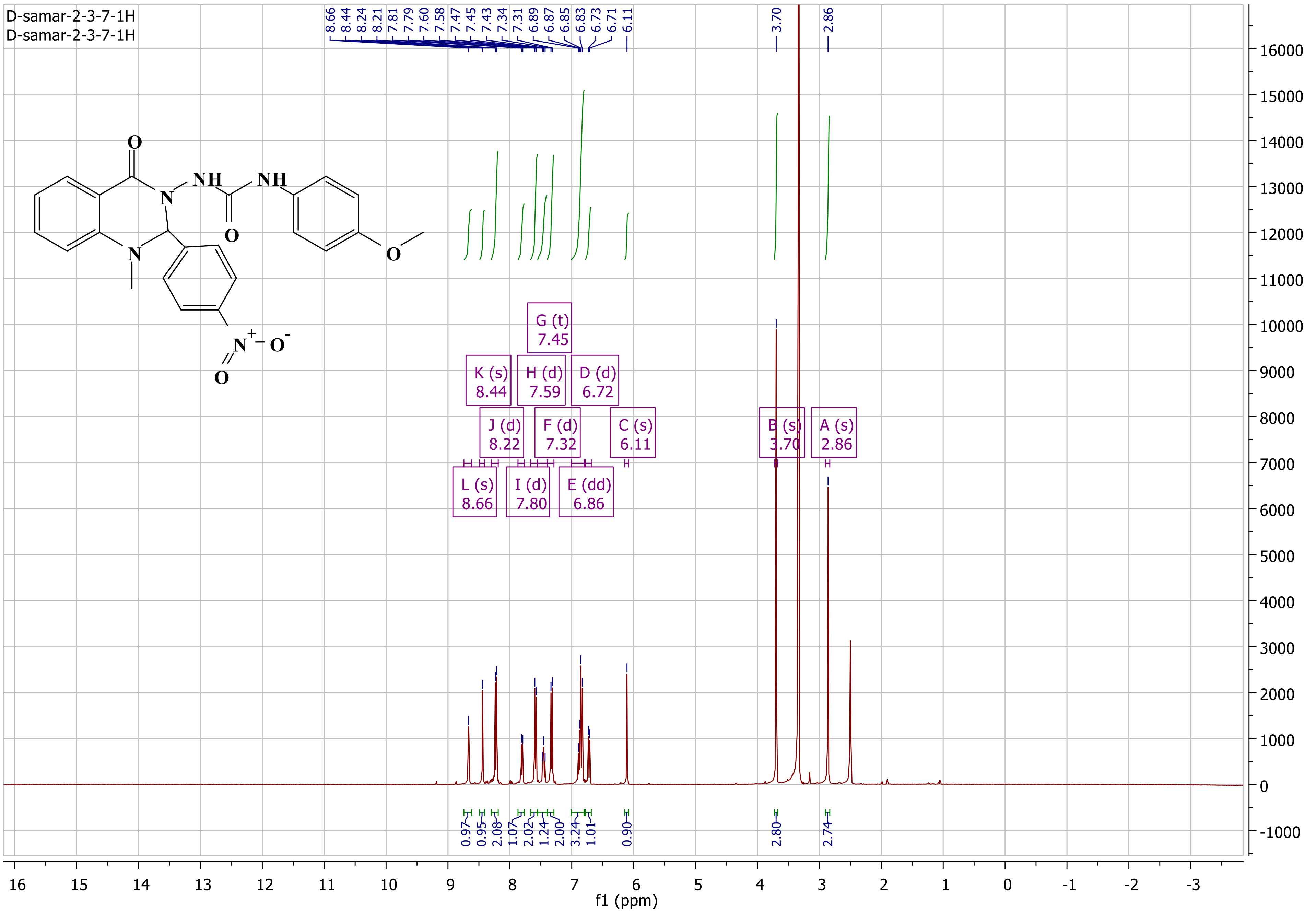
D-samar-2-3-7-1H
D-samar-2-3-7-1H
8.66 8.44 8.24 8.21 7.81 7.79 7.60 7.58 7.47 7.45 7.43 7.34 7.31 6.89 6.87 6.85 6.83 6.73 6.71 6.11
3.70
2.86
G (t) 7.45
K (s) 8.44
H (d) 7.59
D (d) 6.72
J (d) 8.22
F (d) 7.32
C (s) 6.11
B (s) 3.70
A (s) 2.86
L (s) 8.66
I (d) 7.80
E (dd) 6.86
0.97 0.95 2.08 1.07 2.02 1.24 2.00 3.24 1.01 0.90 2.80 2.74
O
NH
NH
N
O
N
O
N+
O-
O
16 15 14 13 12 11 10 9 8 7 6 5 4 3 2 1 0 -1 -2 -3
f1 (ppm)
16000 15000 14000 13000 12000 11000 10000 9000 8000 7000 6000 5000 4000 3000 2000 1000 0 -1000

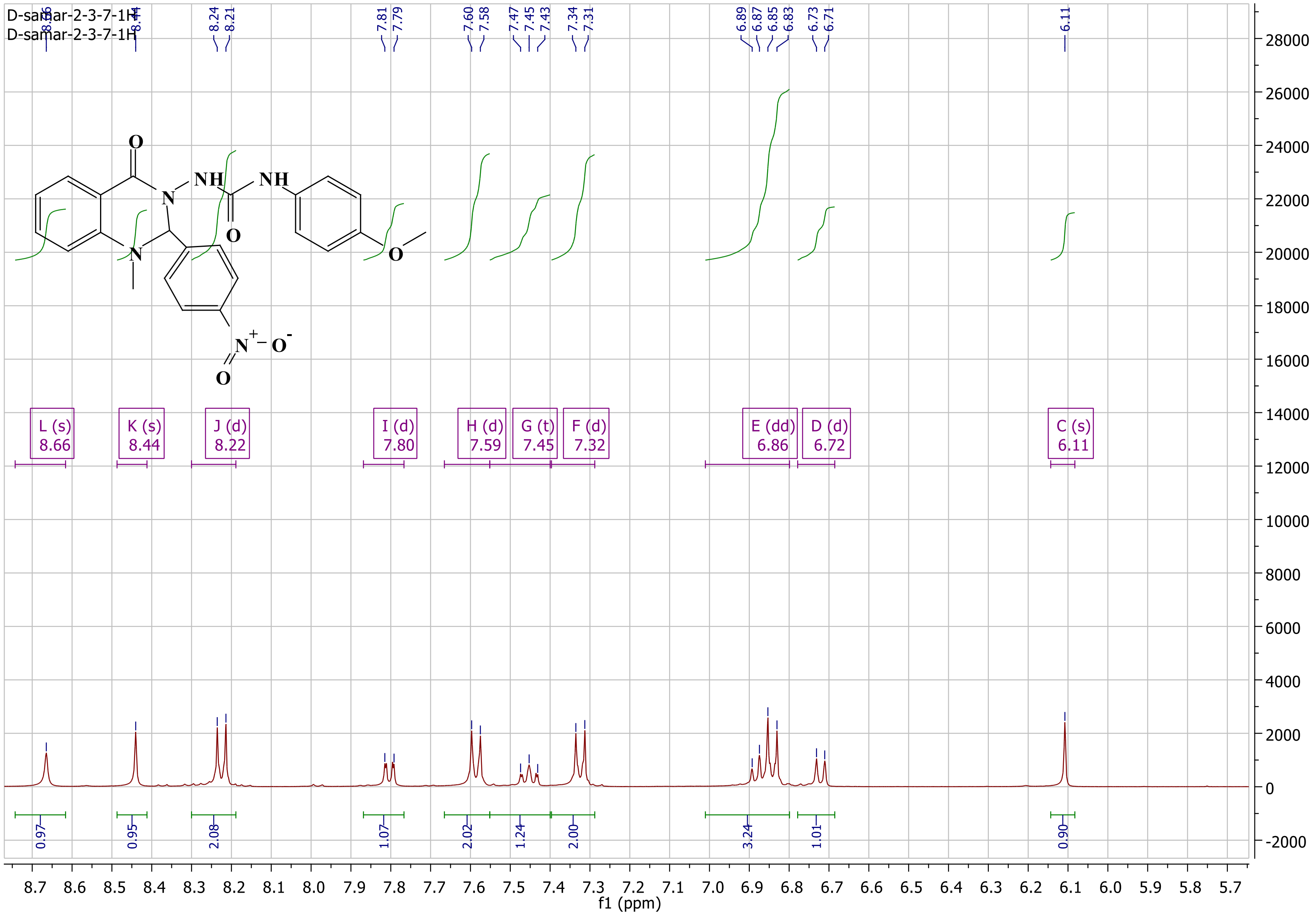
D-samar-2-3-7-1H
D-samar-2-3-7-1H
8.66
8.44
8.24
8.21
7.81
7.79
7.60
7.58
7.47
7.45
7.43
7.34
7.31
6.89
6.87
6.85
6.83
6.73
6.71
6.11
L (s) 8.66
K (s) 8.44
J (d) 8.22
I (d) 7.80
H (d) 7.59
G (t) 7.45
F (d) 7.32
E (dd) 6.86
D (d) 6.72
C (s) 6.11
0.97
0.95
2.08
1.07
2.02
1.24
2.00
3.24
1.01
0.90
f1 (ppm)

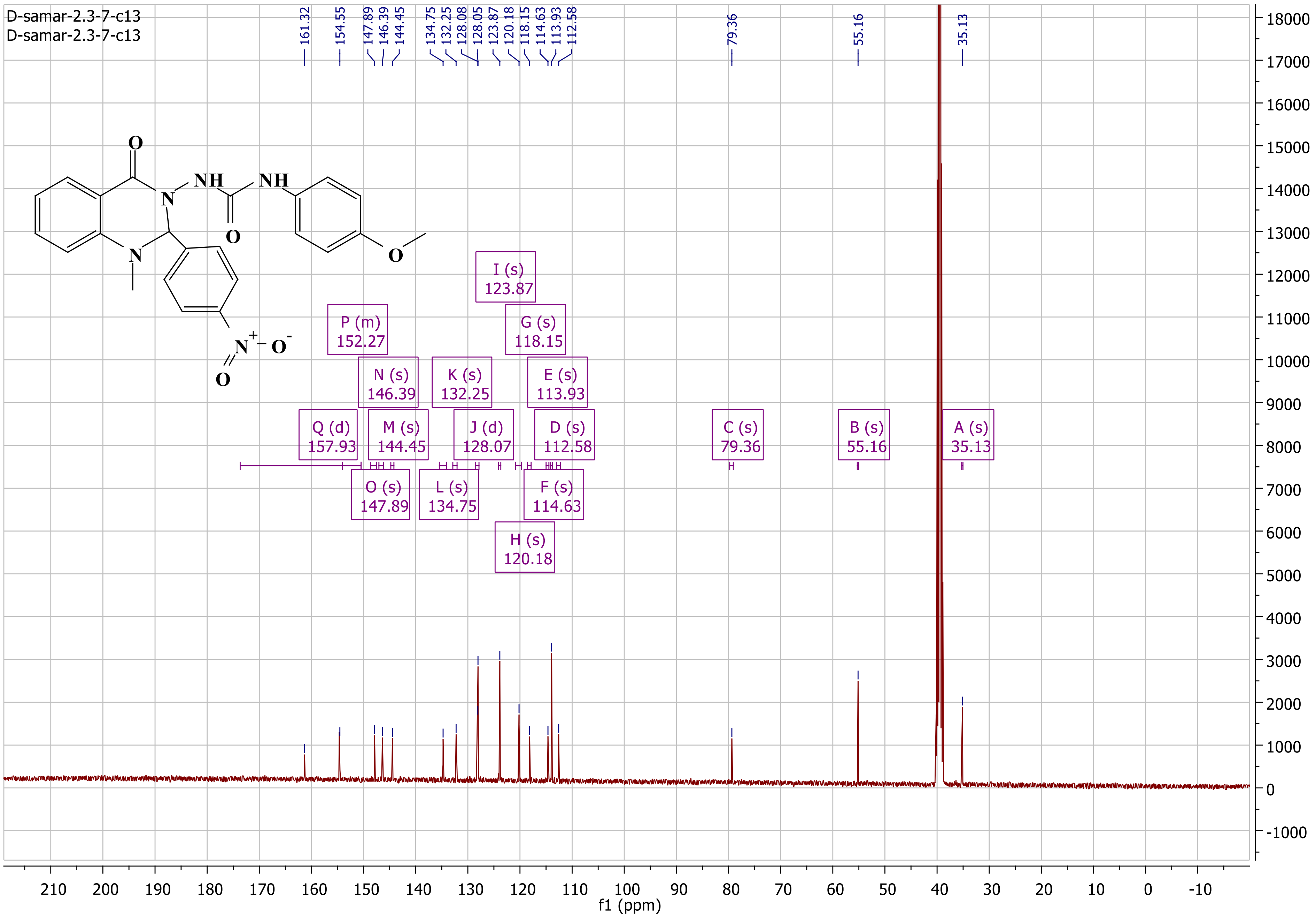
D-samar-2.3-7-c13
D-samar-2.3-7-c13
161.32
154.55
147.89
146.39
144.45
134.75
132.25
128.08
128.05
123.87
120.18
118.15
114.63
113.93
112.58
79.36
55.16
35.13
I (s) 123.87
P (m) 152.27
G (s) 118.15
N (s) 146.39
K (s) 132.25
E (s) 113.93
Q (d) 157.93
M (s) 144.45
J (d) 128.07
D (s) 112.58
C (s) 79.36
B (s) 55.16
A (s) 35.13
O (s) 147.89
L (s) 134.75
F (s) 114.63
H (s) 120.18
f1 (ppm)

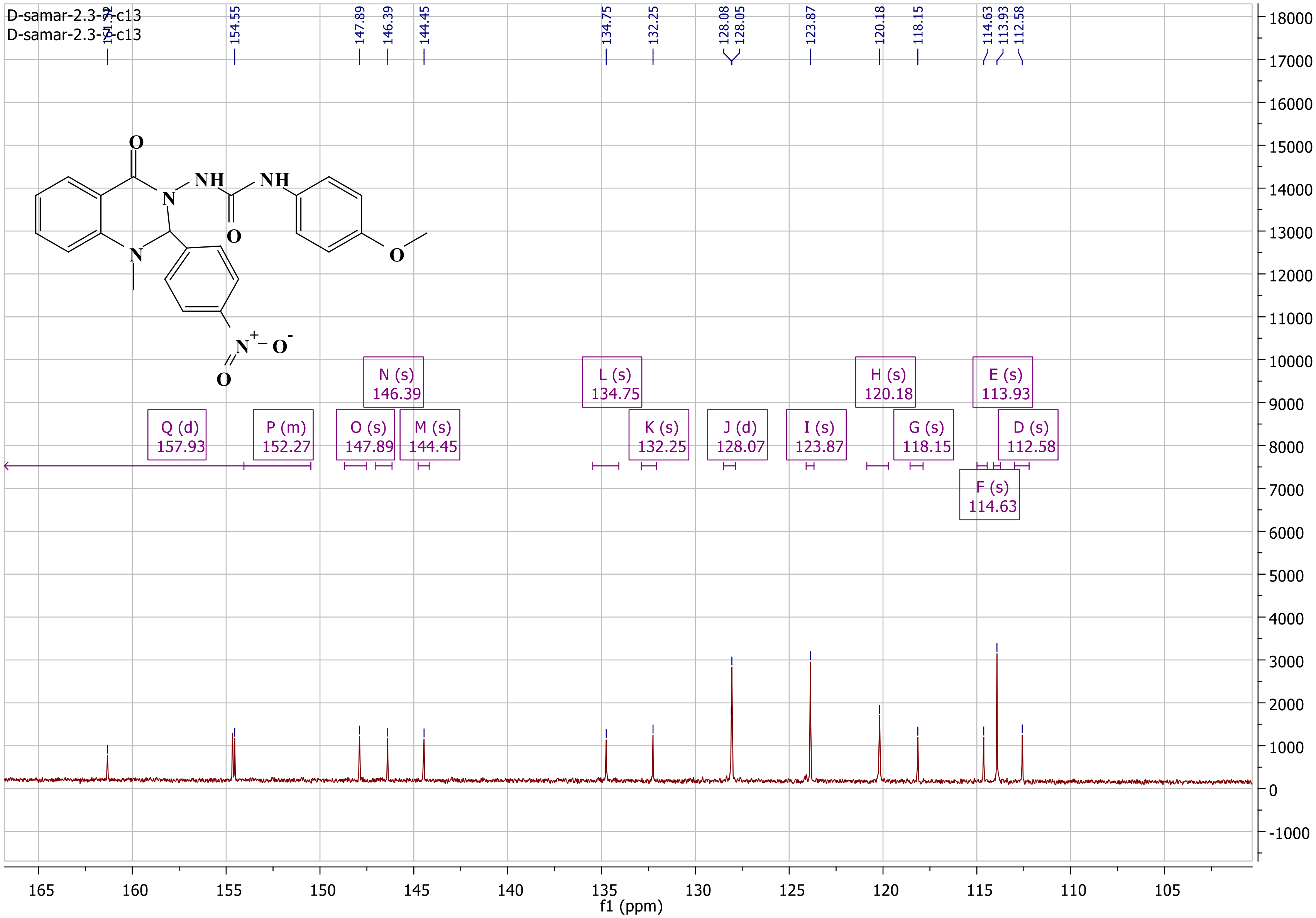
D-samar-2.3-7-c13
D-samar-2.3-7-c13
161.32
154.55
147.89
146.39
144.45
134.75
132.25
128.08
128.05
123.87
120.18
118.15
114.63
113.93
112.58
N (s) 146.39
L (s) 134.75
H (s) 120.18
E (s) 113.93
Q (d) 157.93
P (m) 152.27
O (s) 147.89
M (s) 144.45
K (s) 132.25
J (d) 128.07
I (s) 123.87
G (s) 118.15
D (s) 112.58
F (s) 114.63
f1 (ppm)

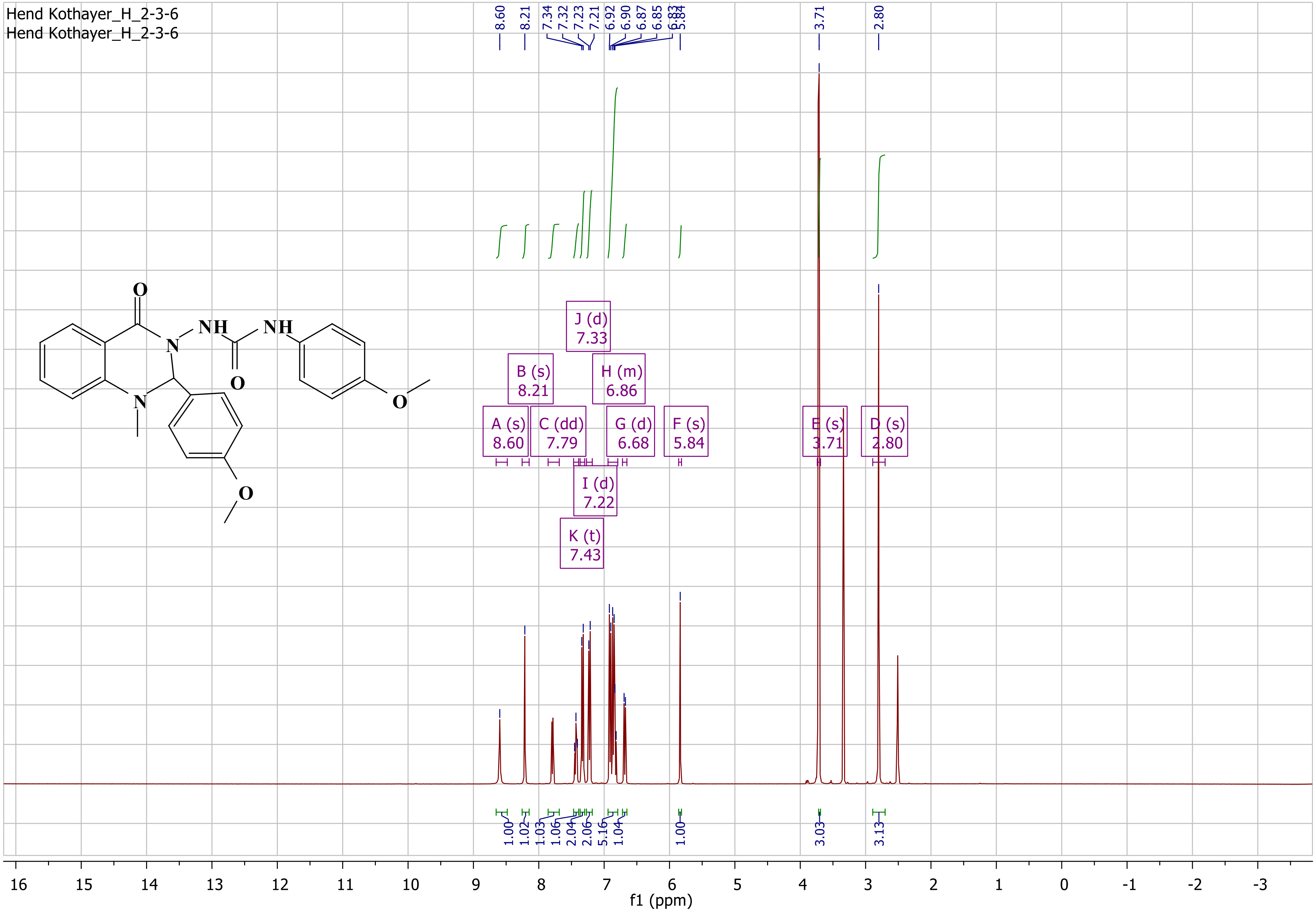

Hend Kothayer_H_2-3-6
Hend Kothayer_H_2-3-6
8.60
8.21
7.34
7.32
7.23
7.21
6.92
6.90
6.87
6.85
6.83
5.84
3.71
2.80
J (d) 7.33
B (s) 8.21
H (m) 6.86
A (s) 8.60
C (dd) 7.79
G (d) 6.68
F (s) 5.84
E (s) 3.71
D (s) 2.80
I (d) 7.22
K (t) 7.43
1.00
1.02
1.03
1.06
2.04
2.06
5.16
1.04
1.00
3.03
3.13
O
NH
NH
N
O
N
O
O
16
15
14
13
12
11
10
9
8
7
6
5
4
3
2
1
0
-1
-2
-3
f1 (ppm)

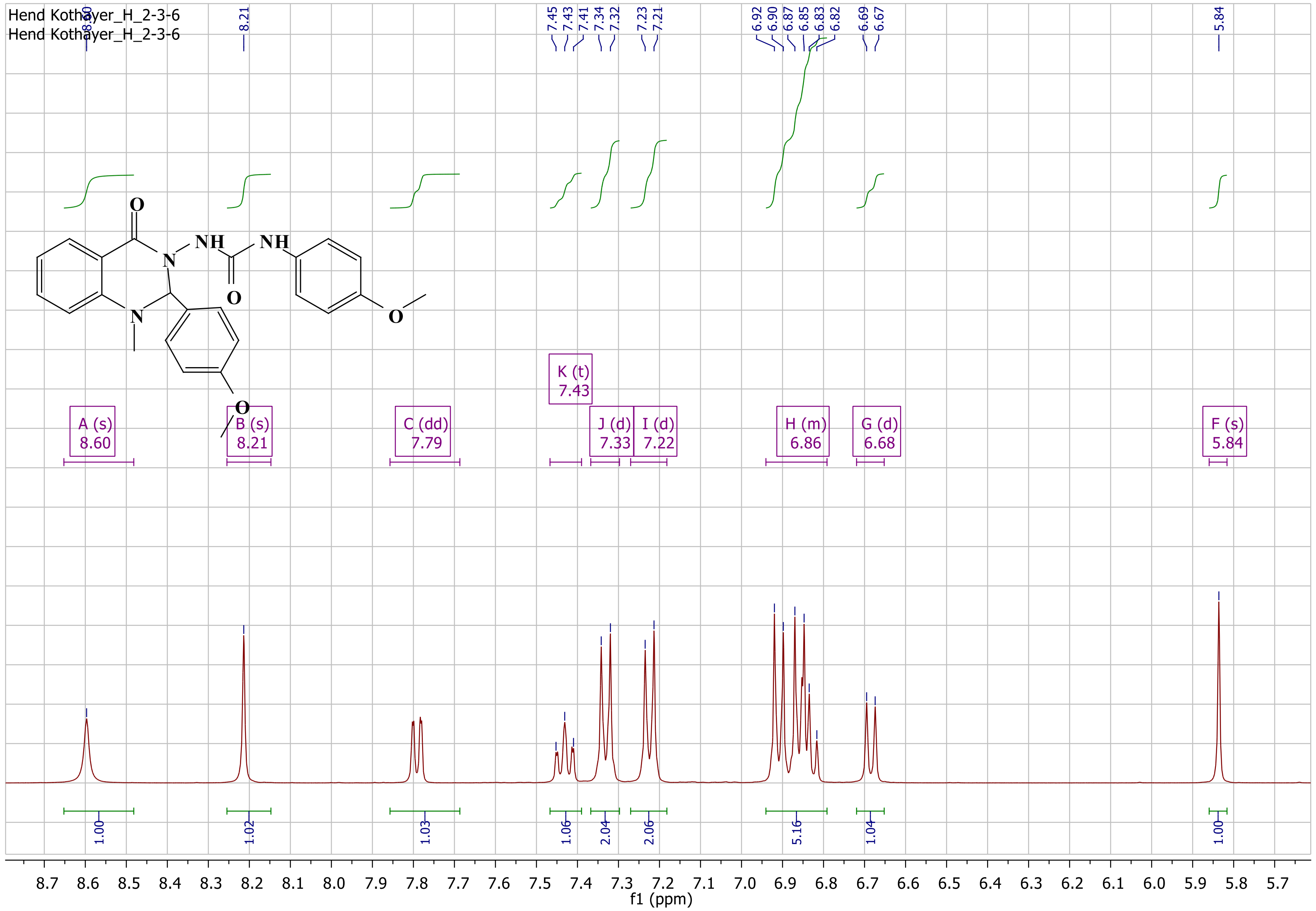
Hend Kothayer_H_2-3-6
Hend Kothayer_H_2-3-6
8.60
8.21
7.45 7.43 7.41 7.34 7.32
7.23 7.21
6.92 6.90 6.87 6.85 6.83 6.82
6.69 6.67
5.84
A (s) 8.60
B (s) 8.21
C (dd) 7.79
K (t) 7.43
J (d) 7.33
I (d) 7.22
H (m) 6.86
G (d) 6.68
F (s) 5.84
1.00
1.02
1.03
1.06
2.04
2.06
5.16
1.04
1.00
8.7 8.6 8.5 8.4 8.3 8.2 8.1 8.0 7.9 7.8 7.7 7.6 7.5 7.4 7.3 7.2 7.1 7.0 6.9 6.8 6.7 6.6 6.5 6.4 6.3 6.2 6.1 6.0 5.9 5.8 5.7
f1 (ppm)
O
N
NH
NH
O
N
O
O

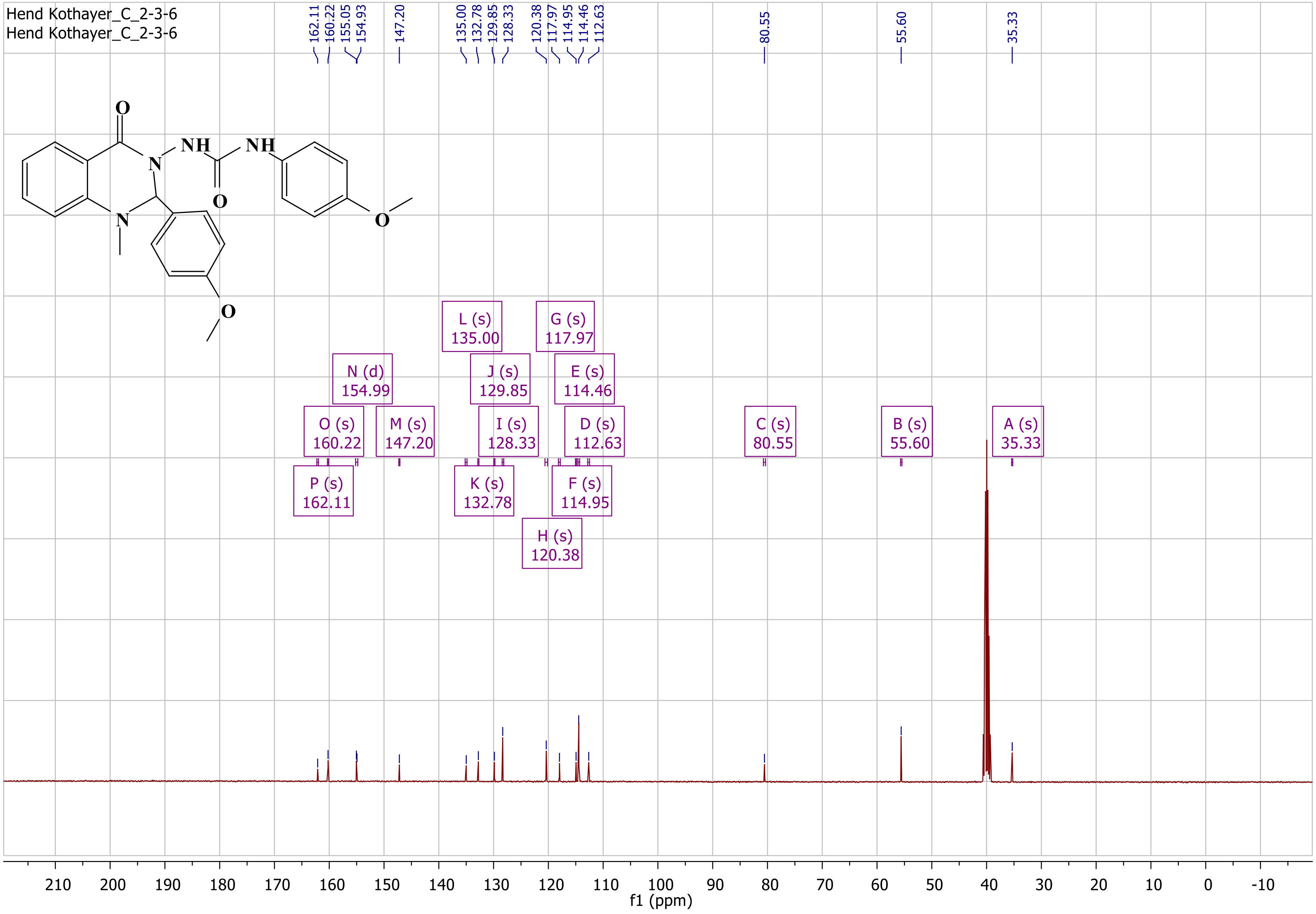
Hend Kothayer_C_2-3-6
Hend Kothayer_C_2-3-6
162.11
160.22
155.05
154.93
147.20
135.00
132.78
129.85
128.33
120.38
117.97
114.95
114.46
112.63
80.55
55.60
35.33
L (s) 135.00
G (s) 117.97
N (d) 154.99
J (s) 129.85
E (s) 114.46
O (s) 160.22
M (s) 147.20
I (s) 128.33
D (s) 112.63
C (s) 80.55
B (s) 55.60
A (s) 35.33
P (s) 162.11
K (s) 132.78
F (s) 114.95
H (s) 120.38
f1 (ppm)

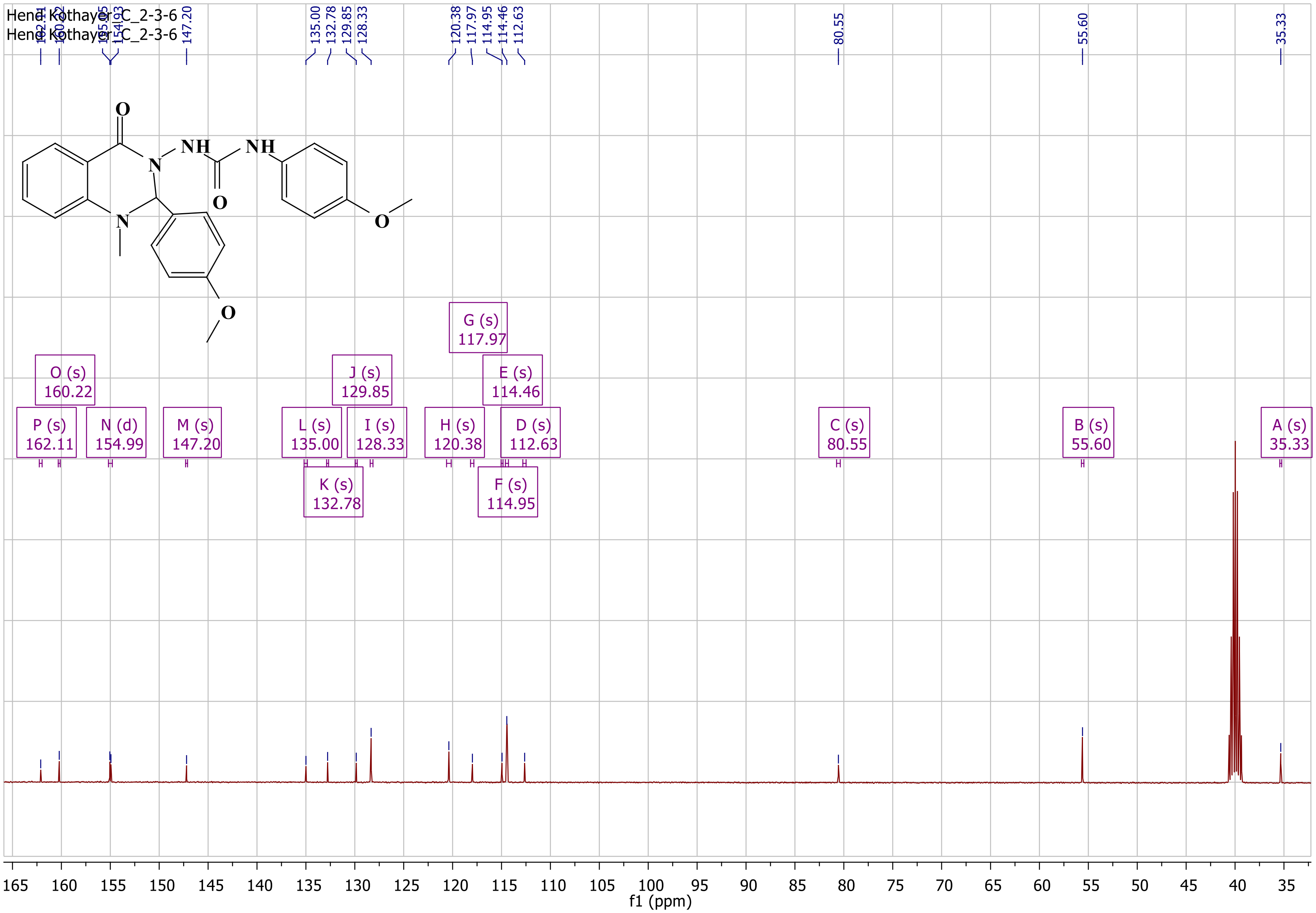

Hend Kothayer_C_2-3-6
Hend Kothayer_C_2-3-6
162.11
160.22
155.05
154.93
147.20
135.00
132.78
129.85
128.33
120.38
117.97
114.95
114.46
112.63
80.55
55.60
35.33
O
NH
NH
N
O
N
O
O
G (s) 117.97
O (s) 160.22
J (s) 129.85
E (s) 114.46
P (s) 162.11
N (d) 154.99
M (s) 147.20
L (s) 135.00
I (s) 128.33
H (s) 120.38
D (s) 112.63
C (s) 80.55
B (s) 55.60
A (s) 35.33
K (s) 132.78
F (s) 114.95
165 160 155 150 145 140 135 130 125 120 115 110 105 100 95 90 85 80 75 70 65 60 55 50 45 40 35
f1 (ppm)

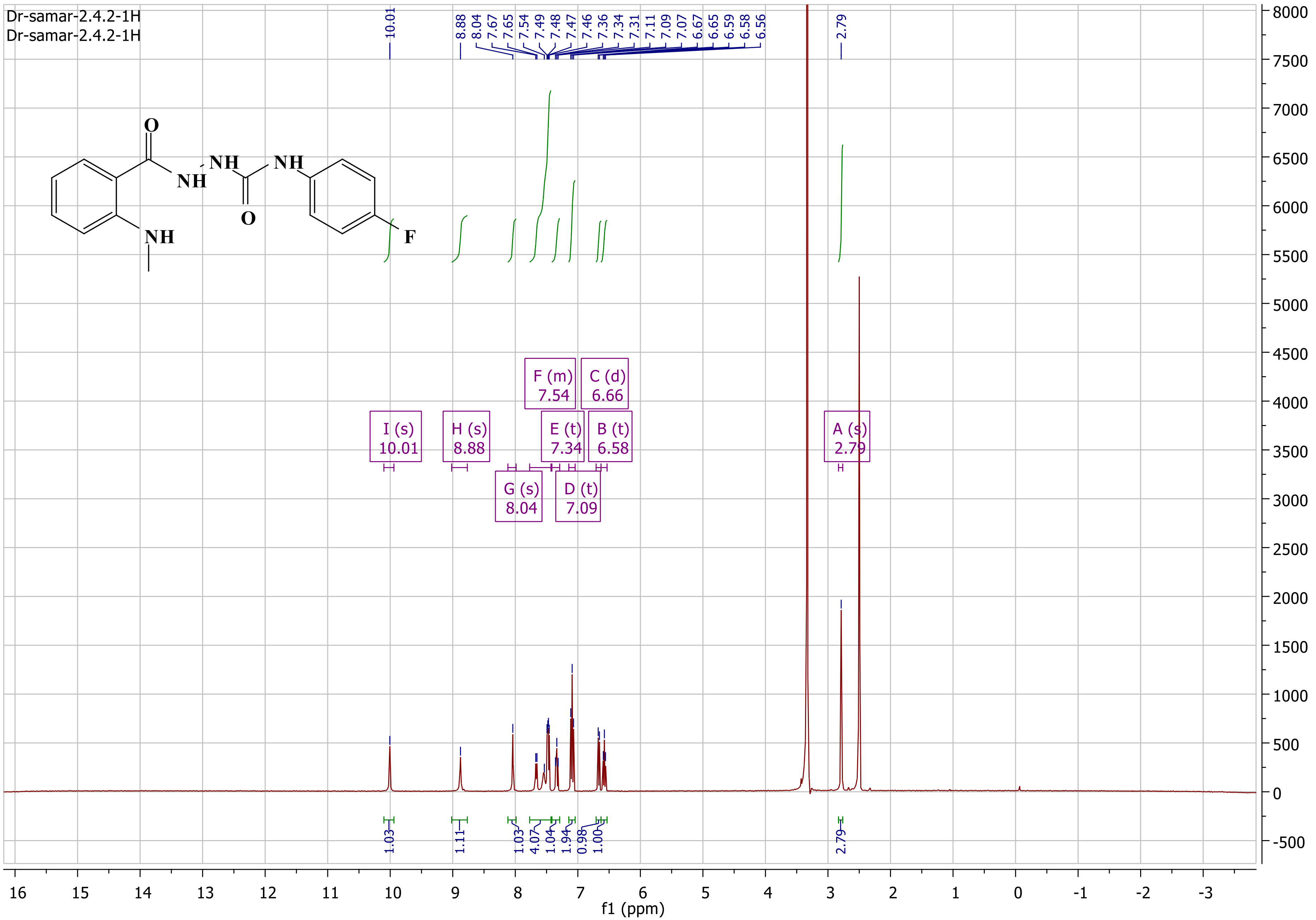

Dr-samar-2.4.2-1H
Dr-samar-2.4.2-1H
I (s) 10.01
H (s) 8.88
G (s) 8.04
F (m) 7.54
E (t) 7.34
D (t) 7.09
C (d) 6.66
B (t) 6.58
A (s) 2.79
f1 (ppm)

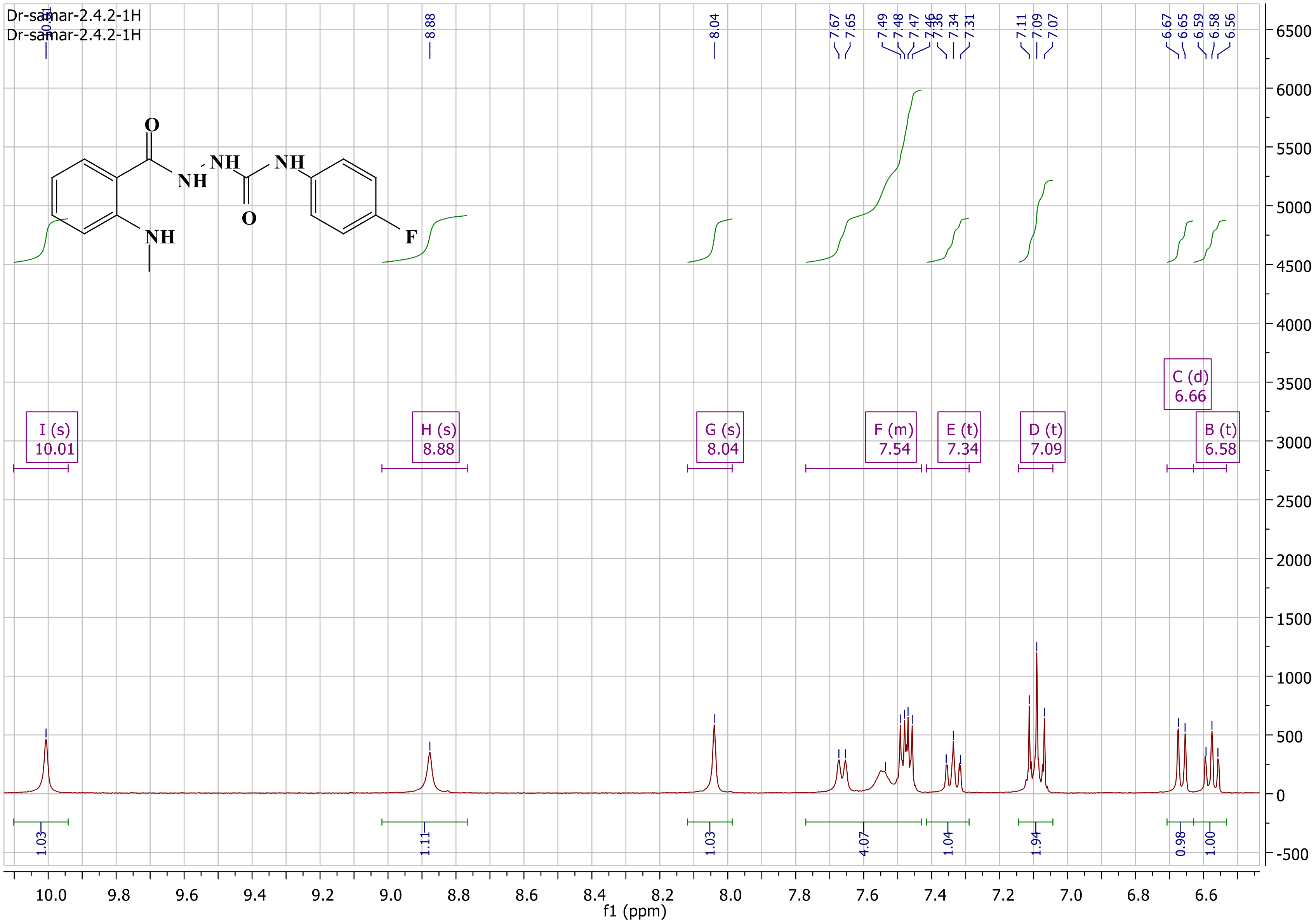

Dr-samar-2.4.2-1H
Dr-samar-2.4.2-1H
10.01
8.88
8.04
7.67
7.65
7.49
7.48
7.47
7.46
7.36
7.34
7.31
7.11
7.09
7.07
6.67
6.65
6.59
6.58
6.56
O
NH
NH
NH
O
NH
F
I (s) 10.01
H (s) 8.88
G (s) 8.04
F (m) 7.54
E (t) 7.34
D (t) 7.09
C (d) 6.66
B (t) 6.58
1.03
1.11
1.03
4.07
1.04
1.94
0.98
1.00
f1 (ppm)

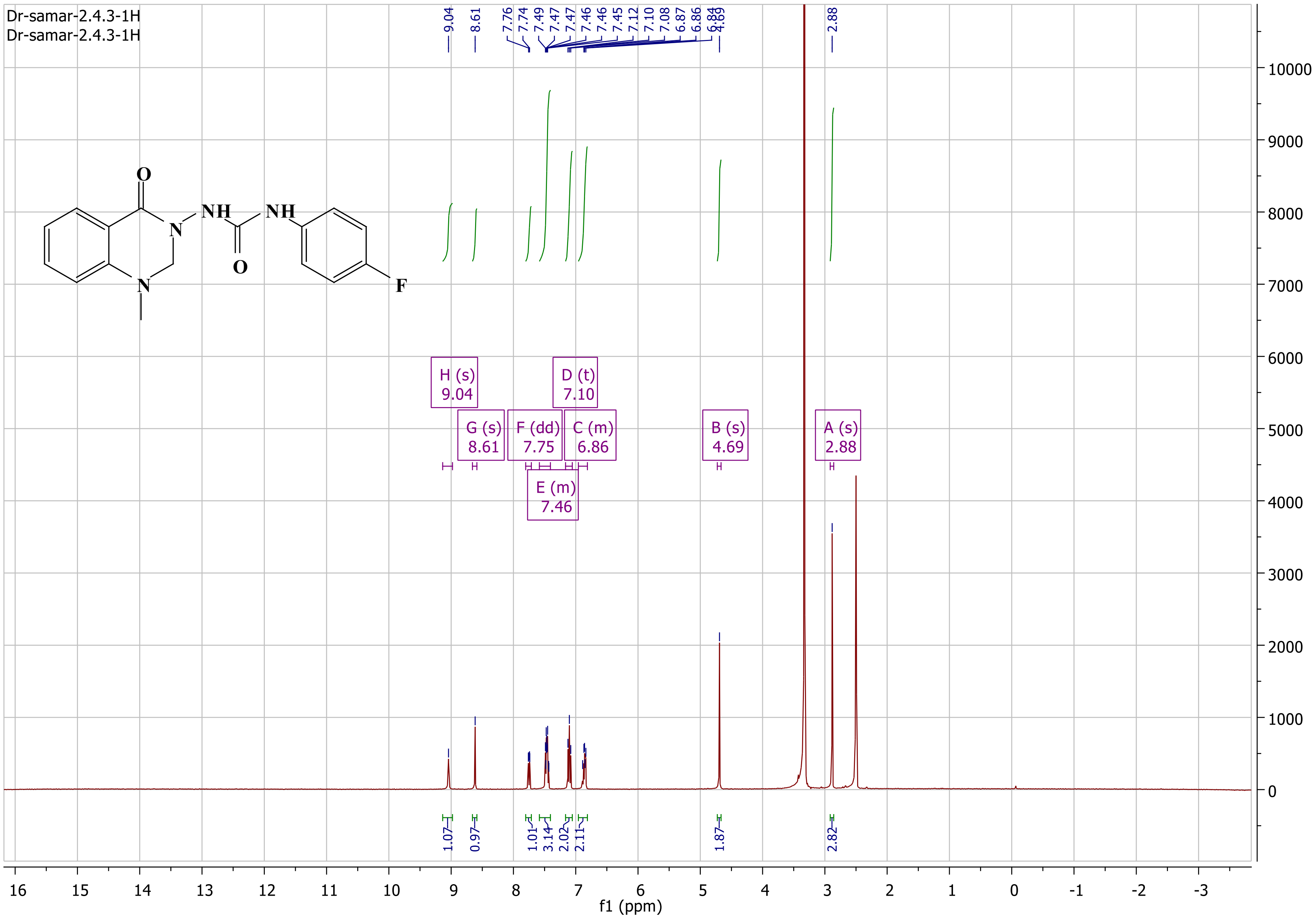
Dr-samar-2.4.3-1H
Dr-samar-2.4.3-1H
9.04
8.61
7.76
7.74
7.49
7.47
7.47
7.46
7.46
7.45
7.12
7.10
7.08
6.87
6.86
6.84
4.69
2.88
O
NH
NH
N
N
O
F
H (s) 9.04
D (t) 7.10
G (s) 8.61
F (dd) 7.75
C (m) 6.86
B (s) 4.69
A (s) 2.88
E (m) 7.46
1.07
0.97
1.01
3.14
2.02
2.11
1.87
2.82
10000
9000
8000
7000
6000
5000
4000
3000
2000
1000
0
16
15
14
13
12
11
10
9
8
7
6
5
4
3
2
1
0
-1
-2
-3
f1 (ppm)

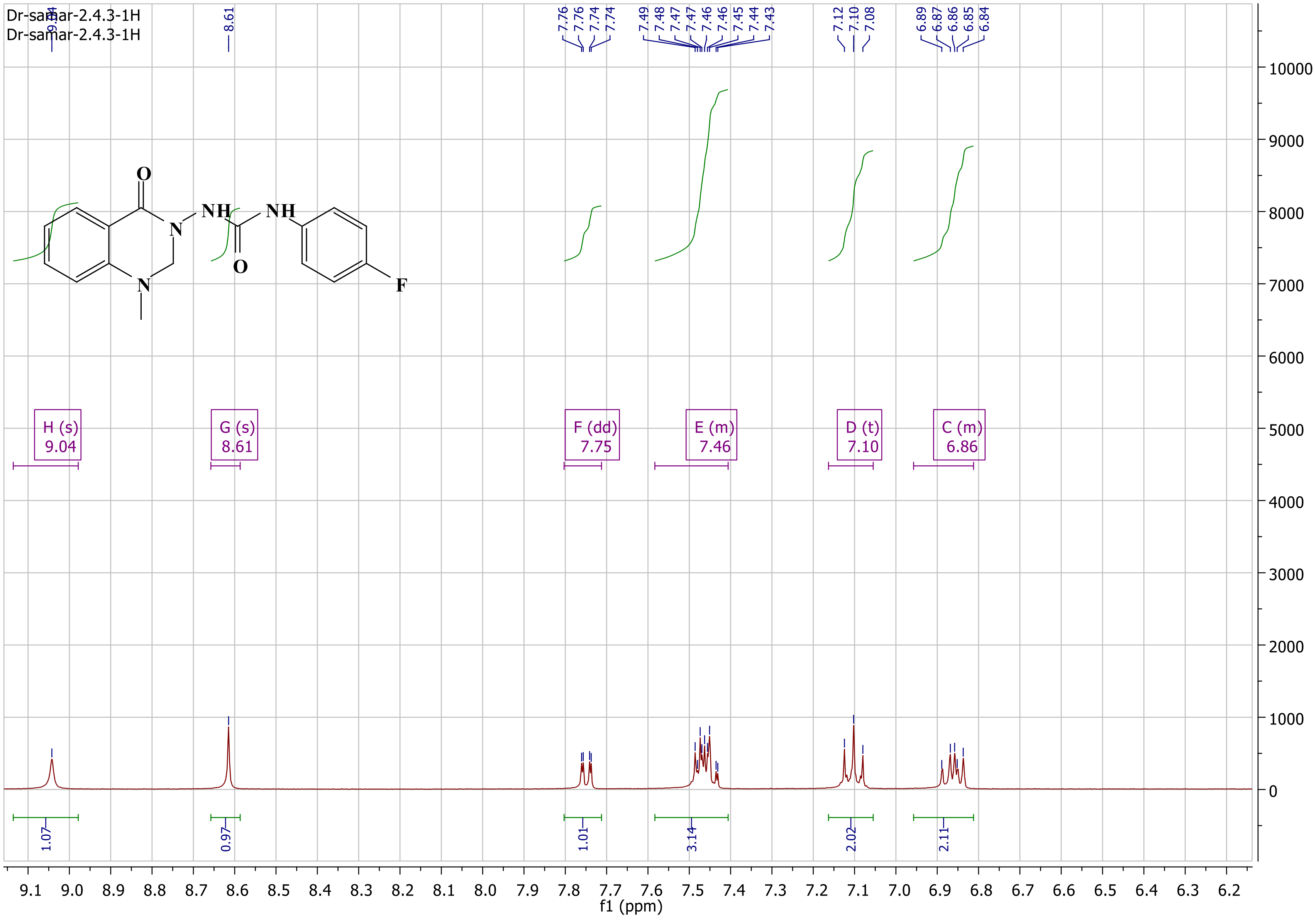

Dr-samar-2.4.3-1H
Dr-samar-2.4.3-1H
9.04
8.61
7.76 7.76 7.74 7.74
7.49 7.48 7.47 7.47 7.46 7.46 7.45 7.44 7.43
7.12 7.10 7.08
6.89 6.87 6.86 6.85 6.84
O
NH
NH
N
O
N
F
H (s) 9.04
G (s) 8.61
F (dd) 7.75
E (m) 7.46
D (t) 7.10
C (m) 6.86
1.07
0.97
1.01
3.14
2.02
2.11
f1 (ppm)

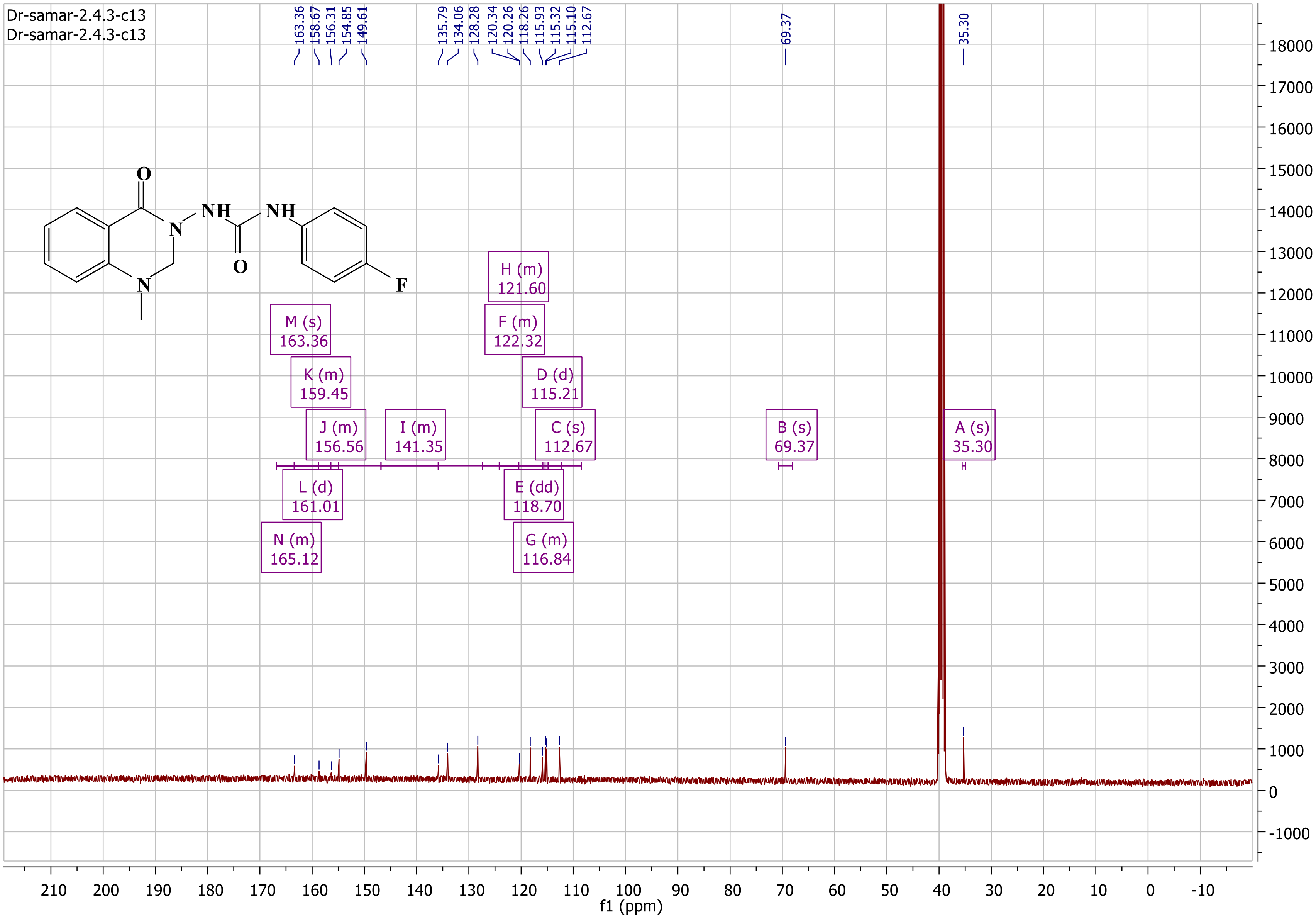
Dr-samar-2.4.3-c13
Dr-samar-2.4.3-c13
163.36
158.67
156.31
154.85
149.61
135.79
134.06
128.28
120.34
120.26
118.26
115.93
115.32
115.10
112.67
69.37
35.30
O
NH
NH
N
N
O
F
H (m) 121.60
M (s) 163.36
F (m) 122.32
K (m) 159.45
D (d) 115.21
J (m) 156.56
I (m) 141.35
C (s) 112.67
B (s) 69.37
A (s) 35.30
L (d) 161.01
E (dd) 118.70
N (m) 165.12
G (m) 116.84
f1 (ppm)

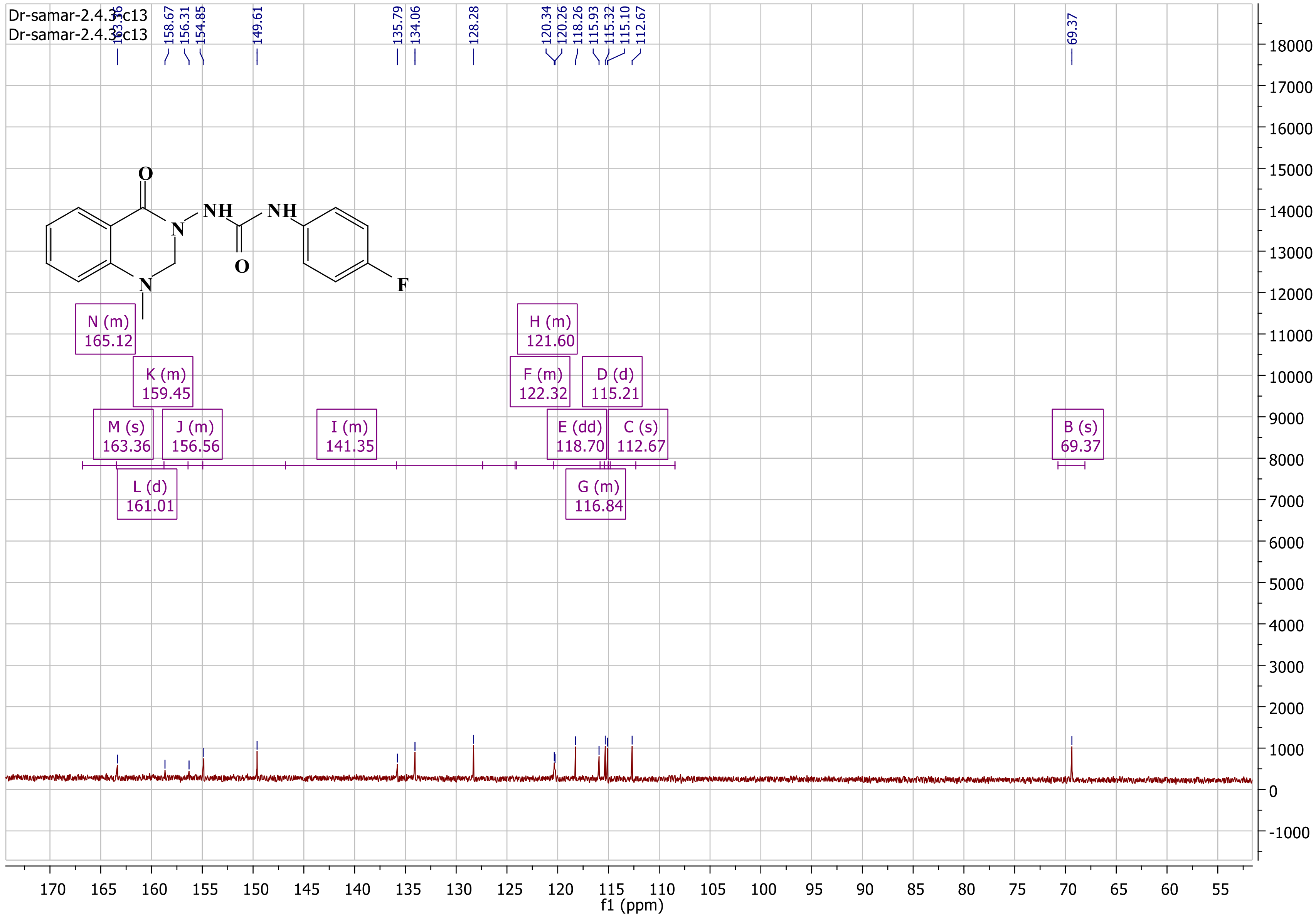
Dr-samar-2.4.3-c13
Dr-samar-2.4.3-c13
163.36
158.67
156.31
154.85
149.61
135.79
134.06
128.28
120.34
120.26
118.26
115.93
115.32
115.10
112.67
69.37
O
NH
NH
N
N
O
F
N (m) 165.12
K (m) 159.45
M (s) 163.36
J (m) 156.56
L (d) 161.01
I (m) 141.35
H (m) 121.60
F (m) 122.32
D (d) 115.21
E (dd) 118.70
C (s) 112.67
G (m) 116.84
B (s) 69.37
f1 (ppm)

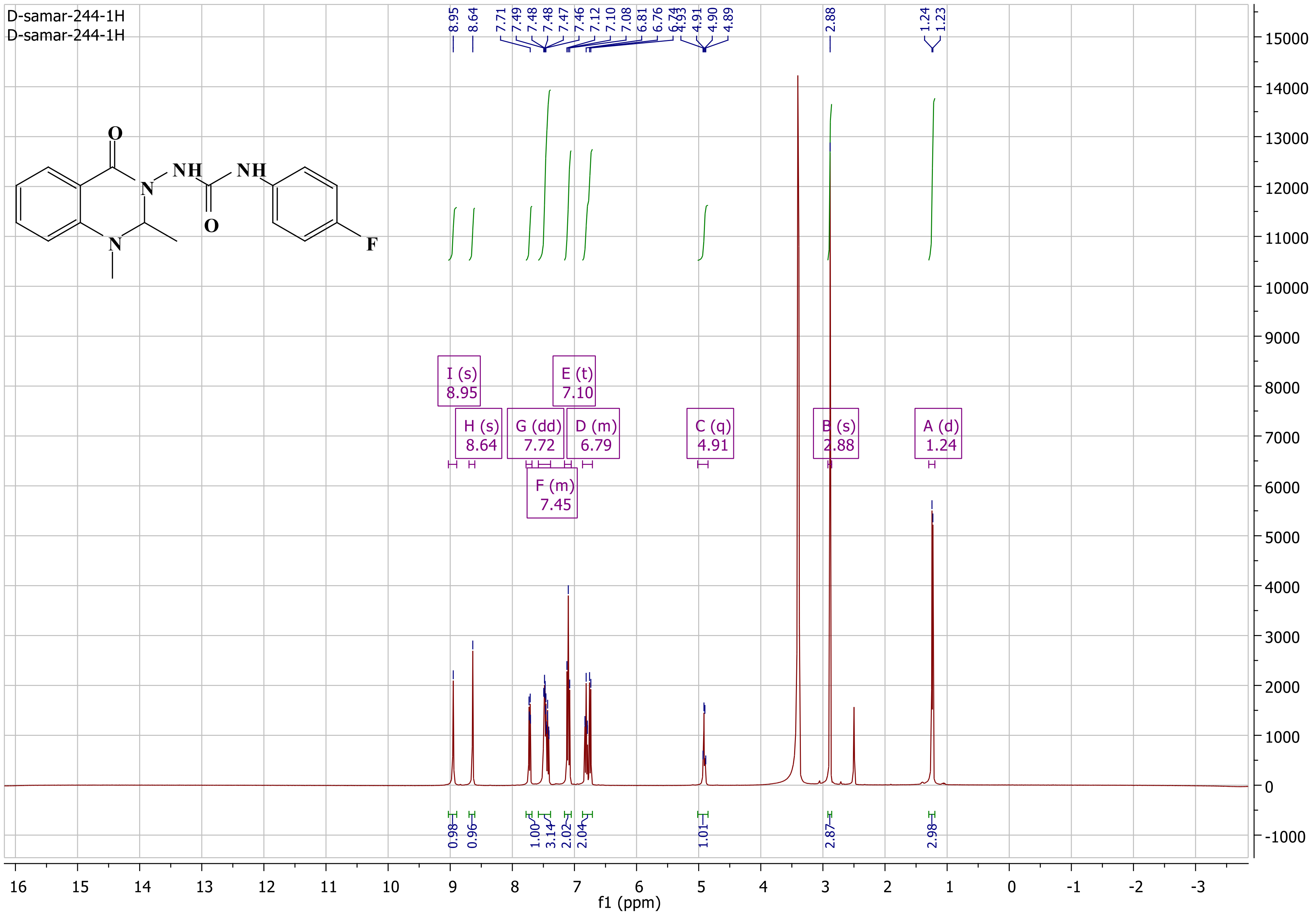
D-samar-244-1H
D-samar-244-1H
8.95
8.64
7.71
7.49
7.48
7.48
7.47
7.46
7.12
7.10
7.08
6.81
6.76
6.74
4.93
4.91
4.90
4.89
2.88
1.24
1.23
O
NH
NH
N
O
N
F
I (s)
8.95
H (s)
8.64
G (dd)
7.72
F (m)
7.45
E (t)
7.10
D (m)
6.79
C (q)
4.91
B (s)
2.88
A (d)
1.24
0.98
0.96
1.00
3.14
2.02
2.04
1.01
2.87
2.98
f1 (ppm)

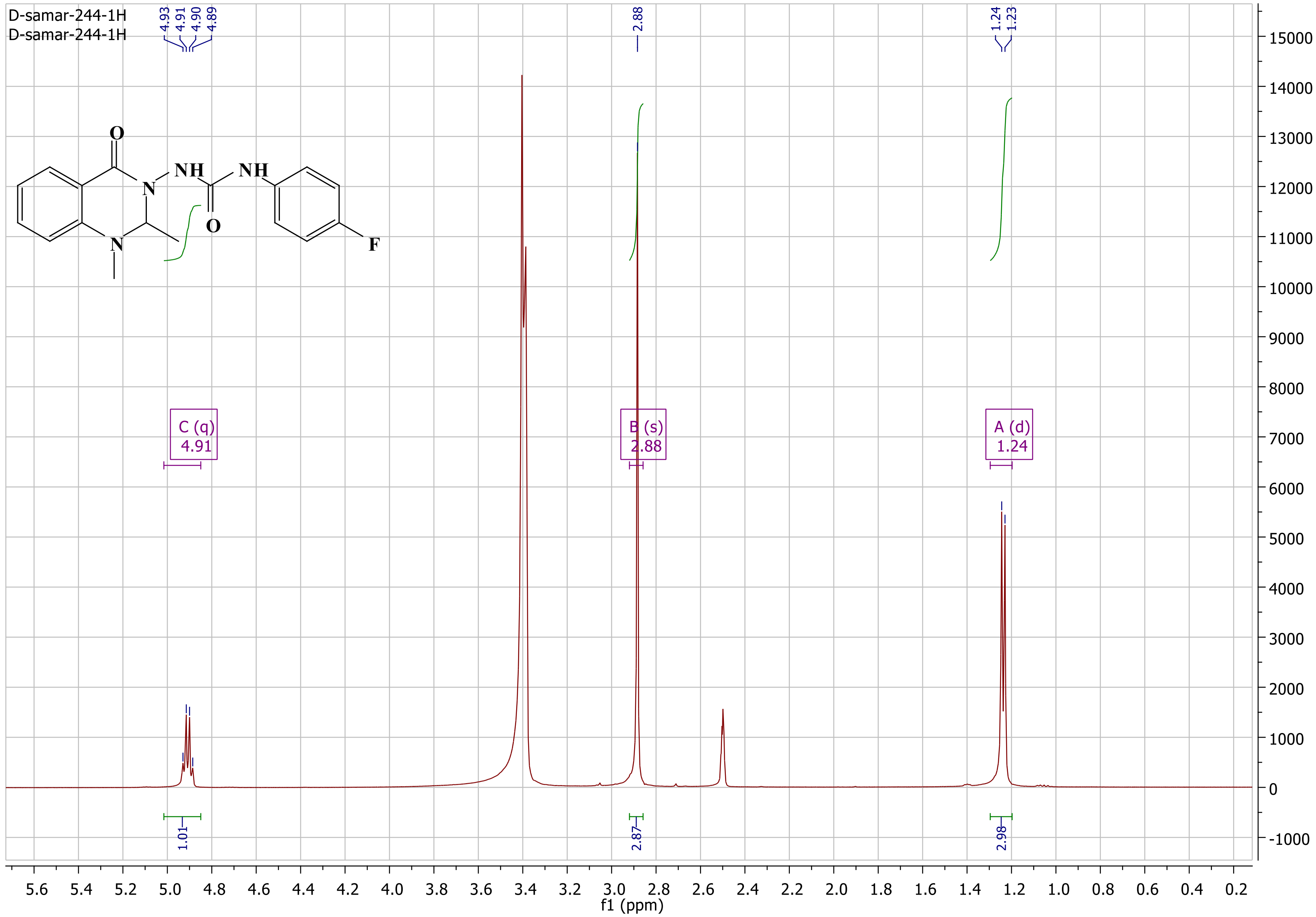
D-samar-244-1H
D-samar-244-1H
4.93
4.91
4.90
4.89
2.88
1.24
1.23
C (q)
4.91
B (s)
2.88
A (d)
1.24
1.01
2.87
2.98
f1 (ppm)

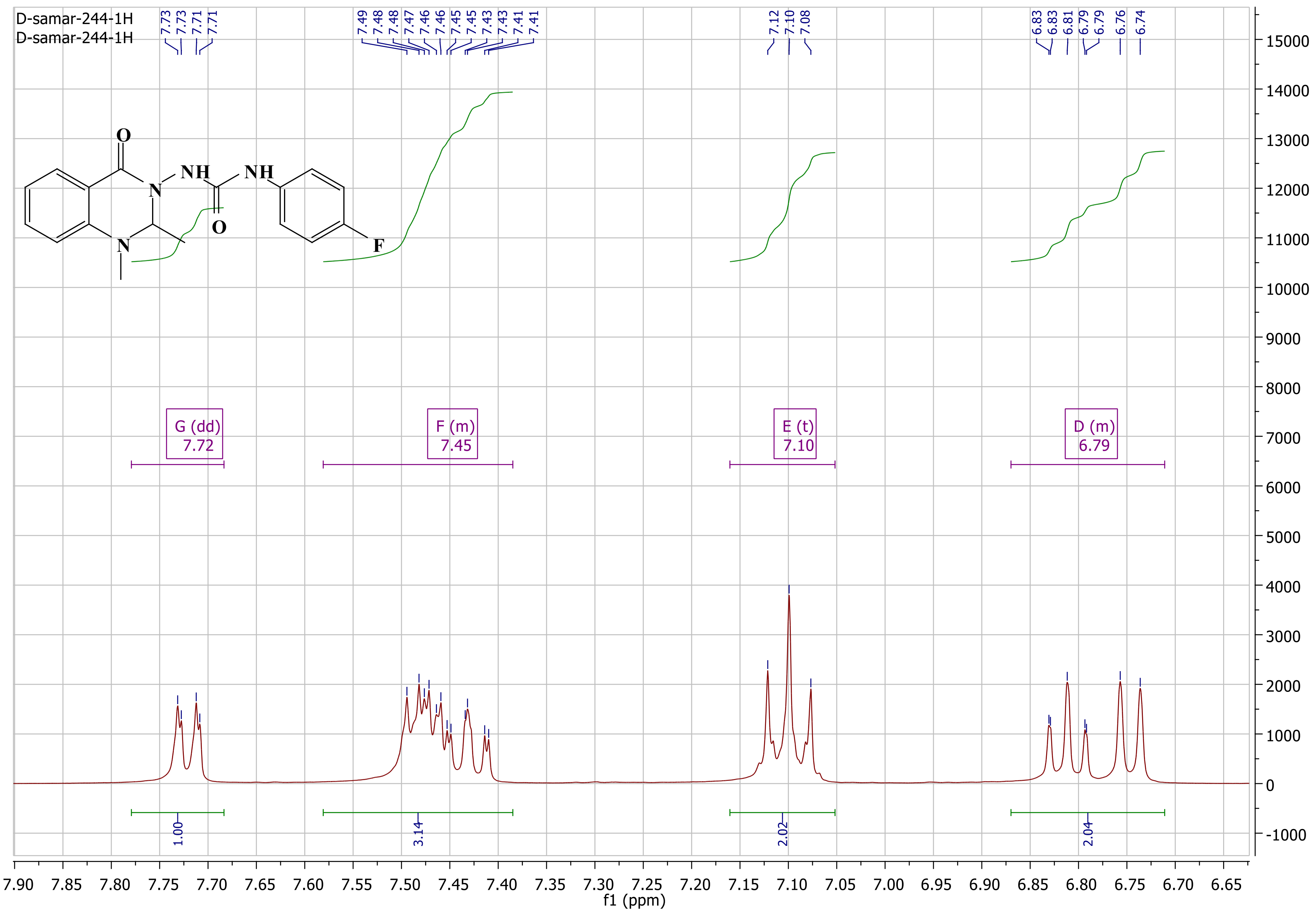
D-samar-244-1H
D-samar-244-1H
7.73
7.73
7.71
7.71
7.49
7.48
7.48
7.47
7.46
7.46
7.45
7.45
7.43
7.43
7.41
7.41
7.12
7.10
7.08
6.83
6.83
6.81
6.79
6.79
6.76
6.74
O
NH
NH
N
N
O
F
G (dd)
7.72
F (m)
7.45
E (t)
7.10
D (m)
6.79
1.00
3.14
2.02
2.04
f1 (ppm)

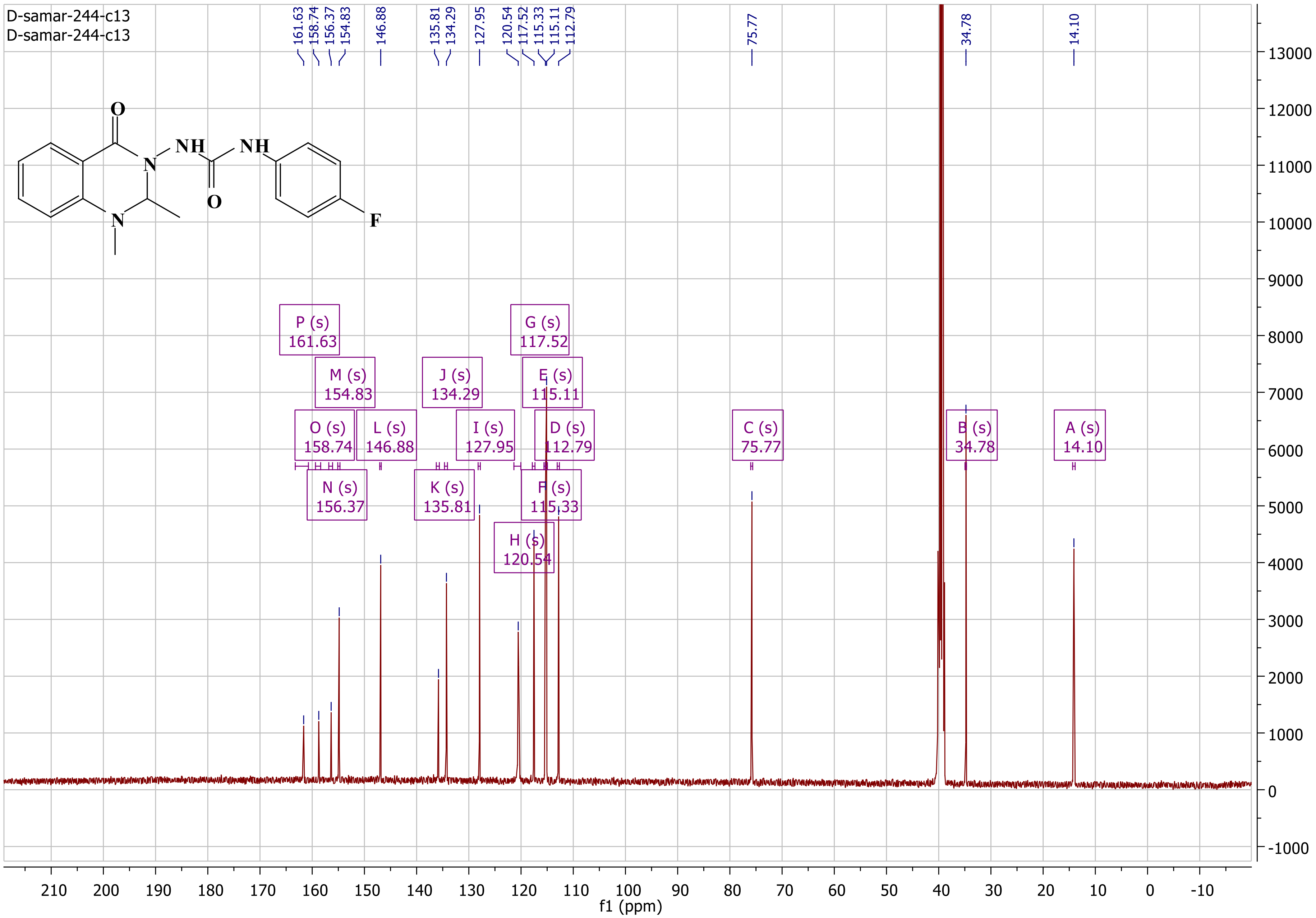
D-samar-244-c13
D-samar-244-c13
161.63
158.74
156.37
154.83
146.88
135.81
134.29
127.95
120.54
117.52
115.33
115.11
112.79
75.77
34.78
14.10
O
NH
NH
N
N
O
F
P (s) 161.63
M (s) 154.83
O (s) 158.74
L (s) 146.88
N (s) 156.37
J (s) 134.29
I (s) 127.95
K (s) 135.81
G (s) 117.52
E (s) 115.11
D (s) 112.79
F (s) 115.33
H (s) 120.54
C (s) 75.77
B (s) 34.78
A (s) 14.10
f1 (ppm)

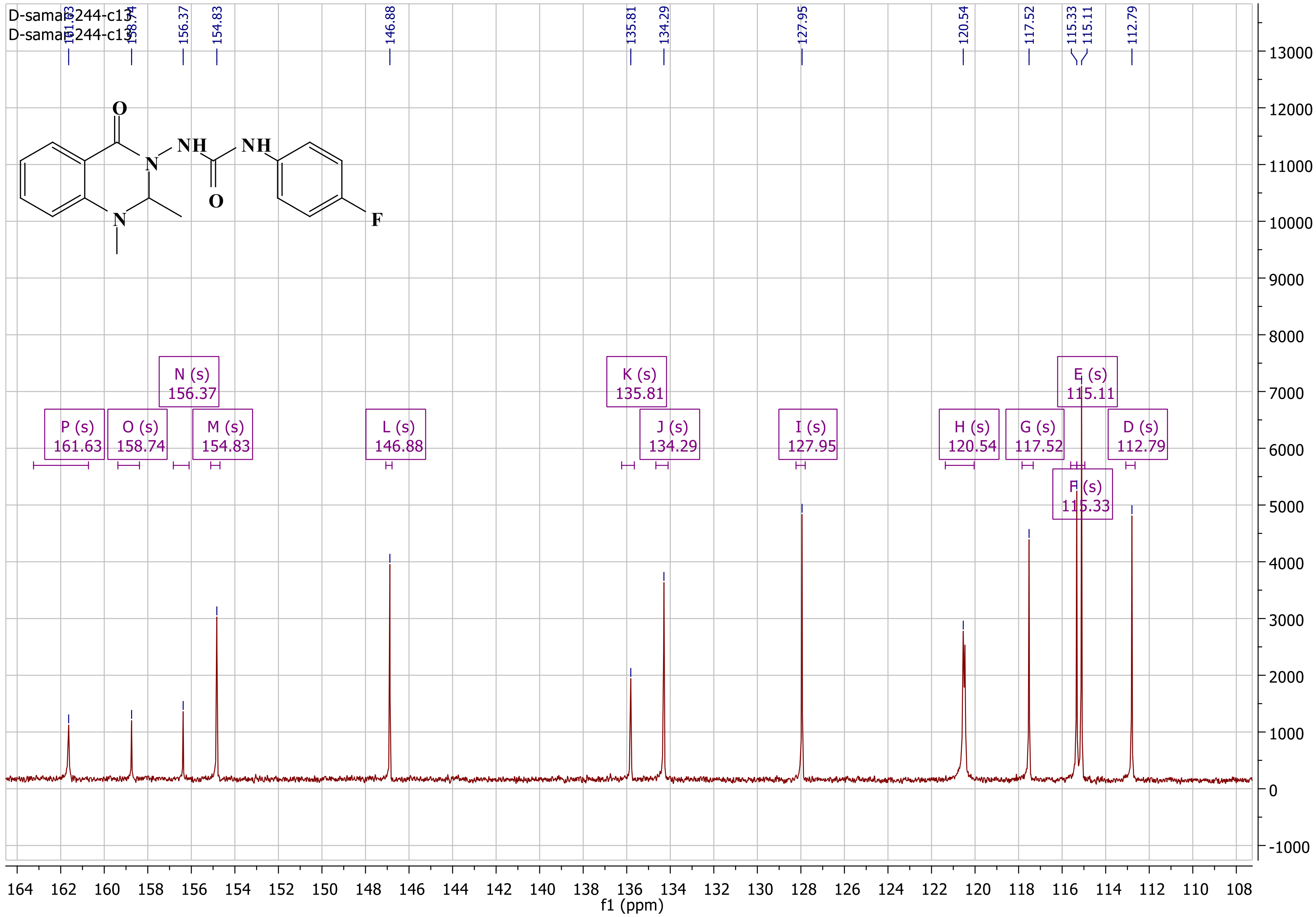
D-samar244-c13
D-samar244-c13
161.63
158.74
156.37
154.83
146.88
135.81
134.29
127.95
120.54
117.52
115.33
115.11
112.79
O
NH
NH
N
N
O
F
P (s) 161.63
O (s) 158.74
N (s) 156.37
M (s) 154.83
L (s) 146.88
K (s) 135.81
J (s) 134.29
I (s) 127.95
H (s) 120.54
G (s) 117.52
F (s) 115.33
E (s) 115.11
D (s) 112.79
13000
12000
11000
10000
9000
8000
7000
6000
5000
4000
3000
2000
1000
0
-1000
164 162 160 158 156 154 152 150 148 146 144 142 140 138 136 134 132 130 128 126 124 122 120 118 116 114 112 110 108
f1 (ppm)

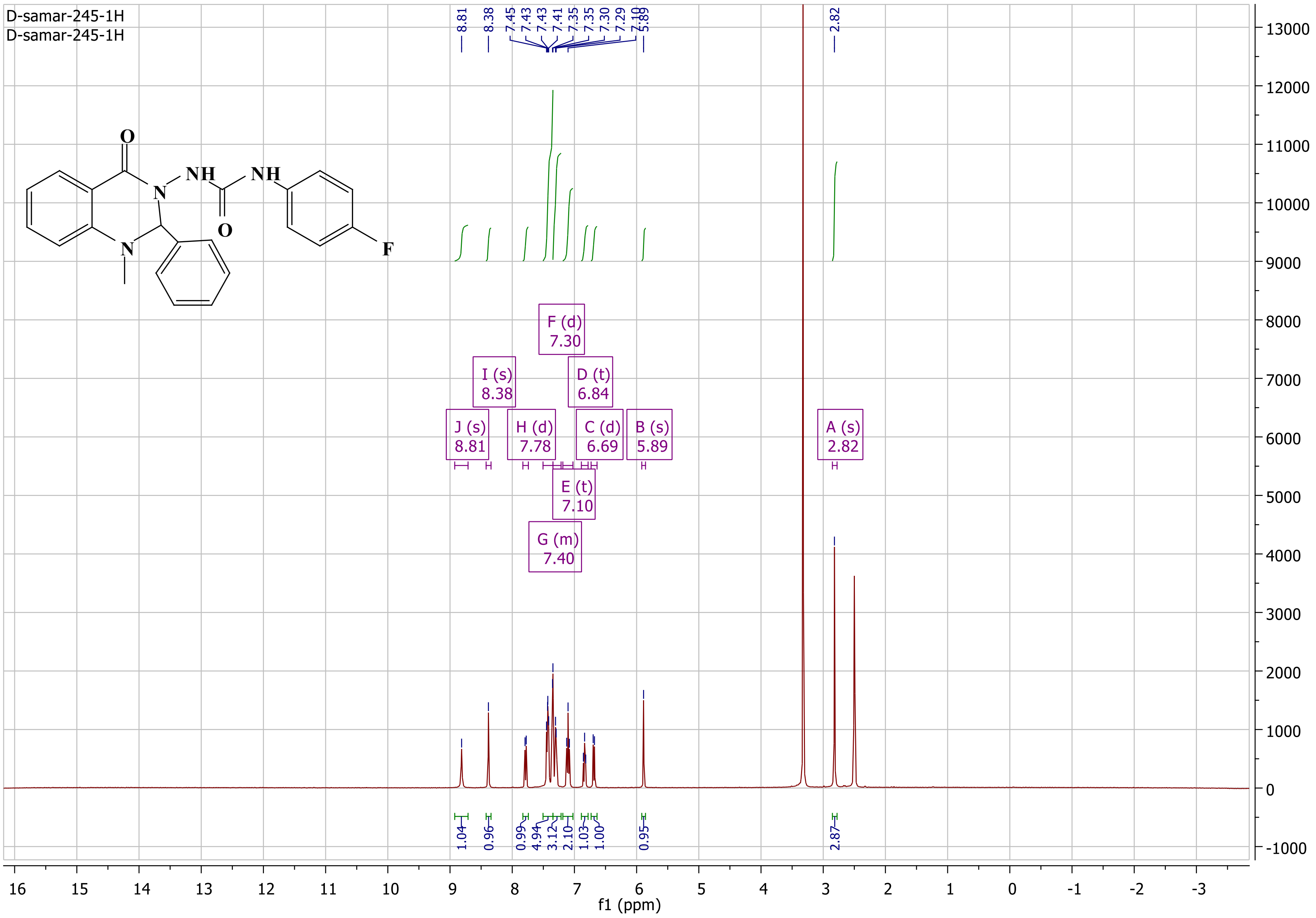
D-samar-245-1H
D-samar-245-1H
8.81
8.38
7.45
7.43
7.43
7.41
7.35
7.35
7.30
7.29
7.10
5.89
2.82
F (d) 7.30
I (s) 8.38
D (t) 6.84
J (s) 8.81
H (d) 7.78
C (d) 6.69
B (s) 5.89
A (s) 2.82
E (t) 7.10
G (m) 7.40
1.04
0.96
0.99
4.94
3.12
2.10
1.03
1.00
0.95
2.87
f1 (ppm)

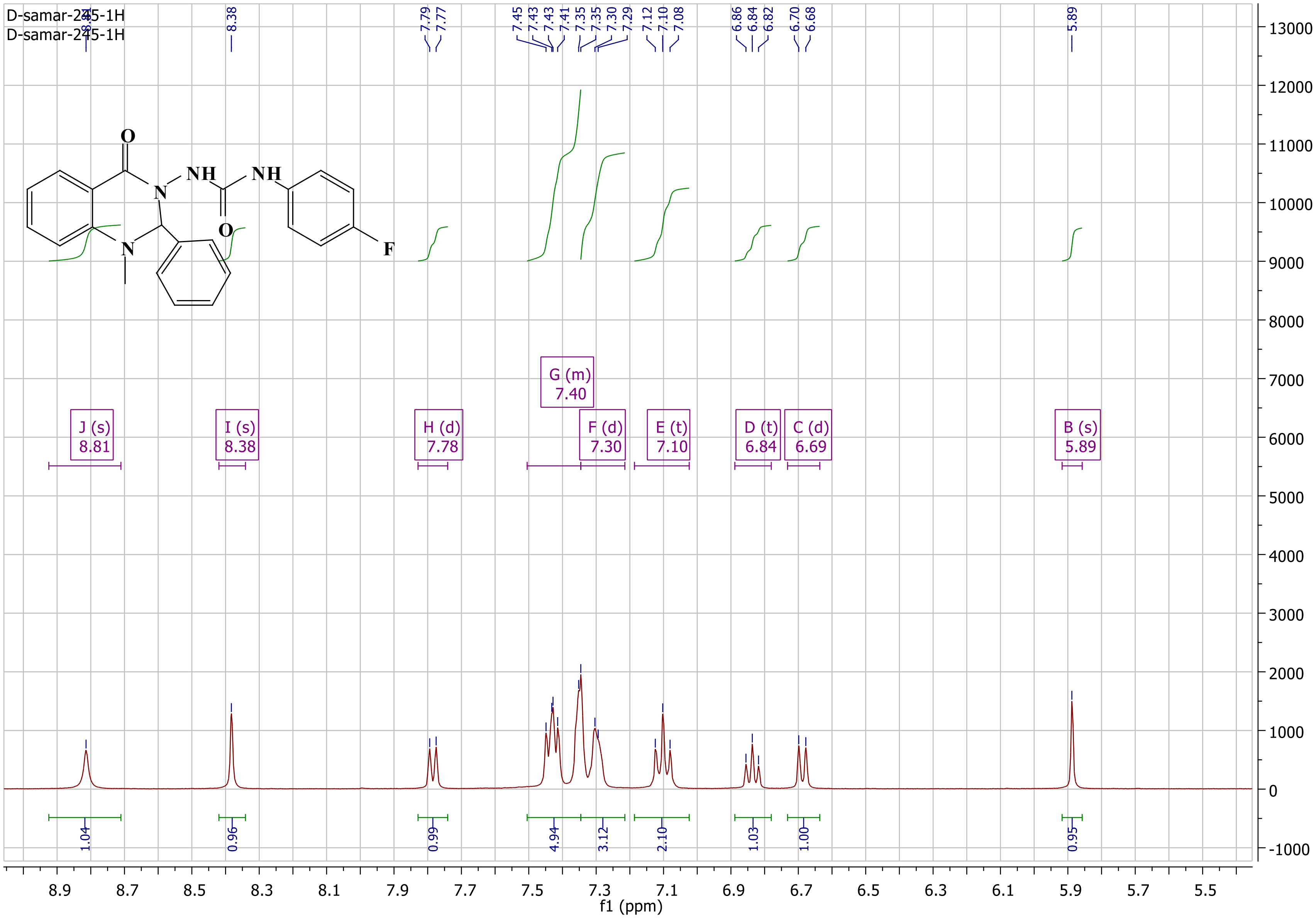

D-samar-245-1H
D-samar-245-1H
8.38
7.79
7.77
7.45
7.43
7.43
7.41
7.35
7.35
7.30
7.29
7.12
7.10
7.08
6.86
6.84
6.82
6.70
6.68
5.89
O
NH
NH
N
N
O
F
G (m) 7.40
J (s) 8.81
I (s) 8.38
H (d) 7.78
F (d) 7.30
E (t) 7.10
D (t) 6.84
C (d) 6.69
B (s) 5.89
1.04
0.96
0.99
4.94
3.12
2.10
1.03
1.00
0.95
f1 (ppm)

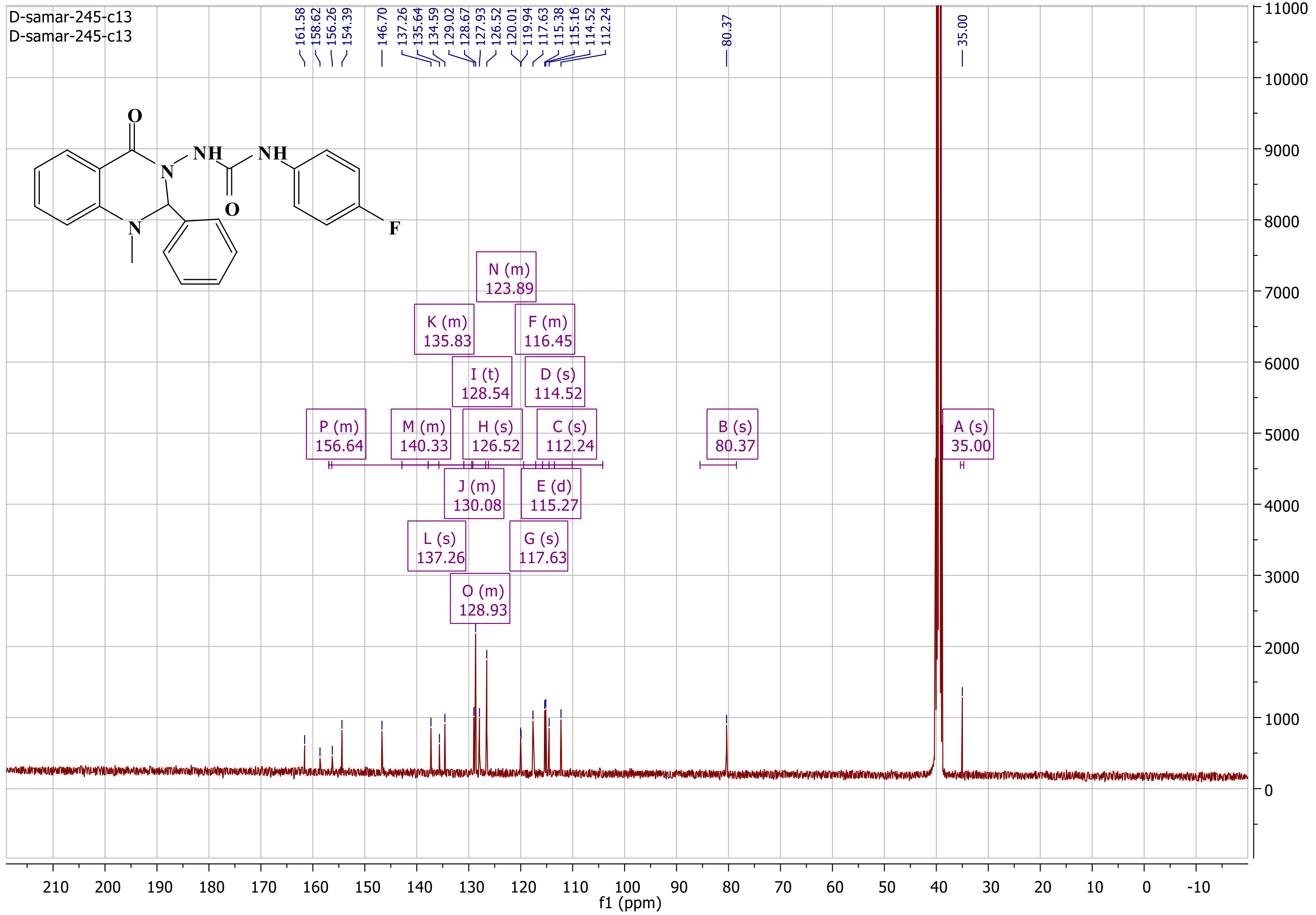

D-samar-245-c13
D-samar-245-c13
161.58
158.62
156.26
154.39
146.70
137.26
135.64
134.59
129.02
128.67
127.93
126.52
120.01
119.94
117.63
115.38
115.16
114.52
112.24
80.37
35.00
O
NH
NH
N
O
N
F
N (m) 123.89
K (m) 135.83
F (m) 116.45
I (t) 128.54
D (s) 114.52
P (m) 156.64
M (m) 140.33
H (s) 126.52
C (s) 112.24
B (s) 80.37
A (s) 35.00
J (m) 130.08
E (d) 115.27
L (s) 137.26
G (s) 117.63
O (m) 128.93
f1 (ppm)

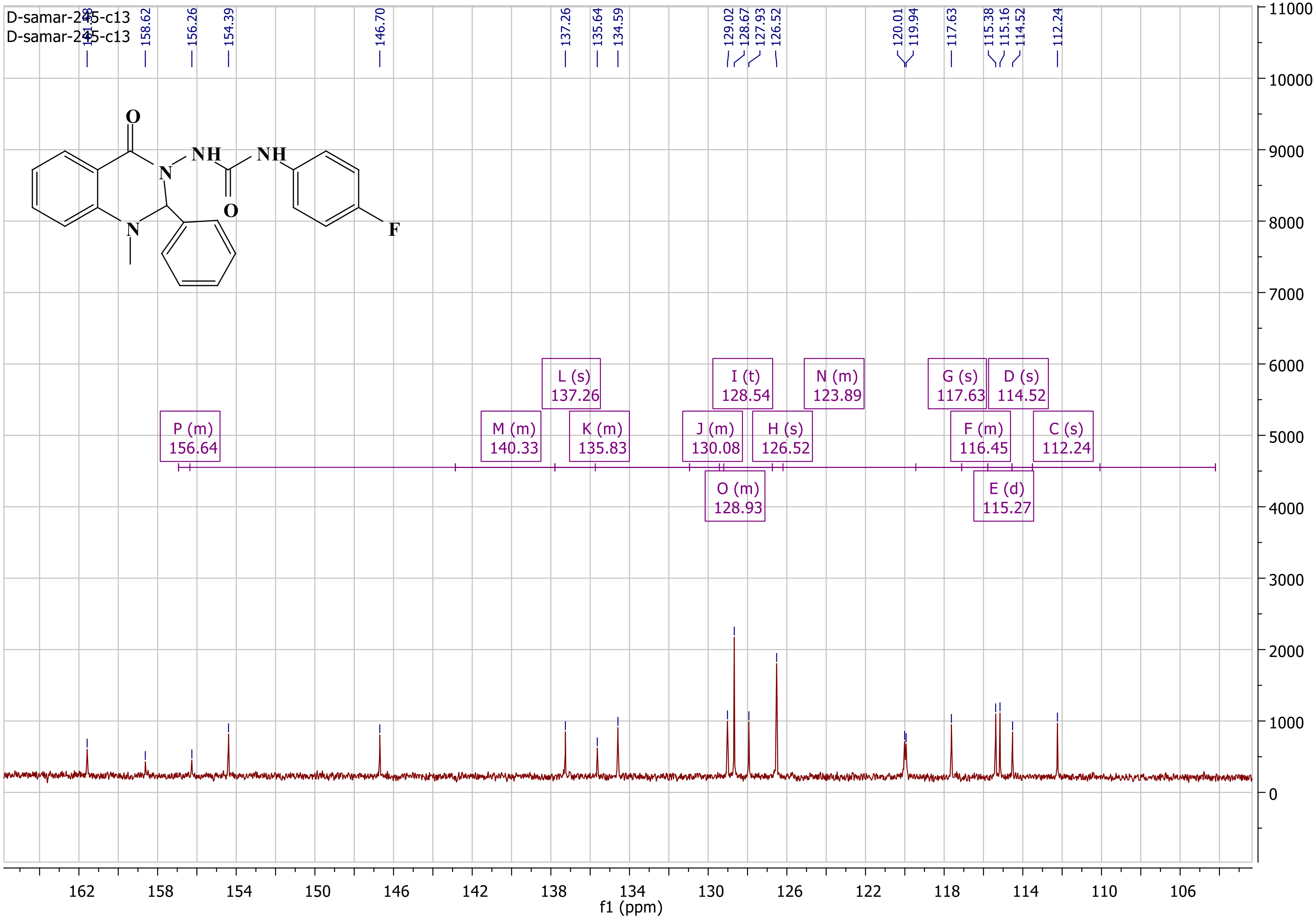

D-samar-245-c13
D-samar-245-c13
158.62
156.26
154.39
146.70
137.26
135.64
134.59
129.02
128.67
127.93
126.52
120.01
119.94
117.63
115.38
115.16
114.52
112.24
O
NH
NH
N
N
O
F
L (s) 137.26
I (t) 128.54
N (m) 123.89
G (s) 117.63
D (s) 114.52
P (m) 156.64
M (m) 140.33
K (m) 135.83
J (m) 130.08
H (s) 126.52
F (m) 116.45
C (s) 112.24
O (m) 128.93
E (d) 115.27
f1 (ppm)

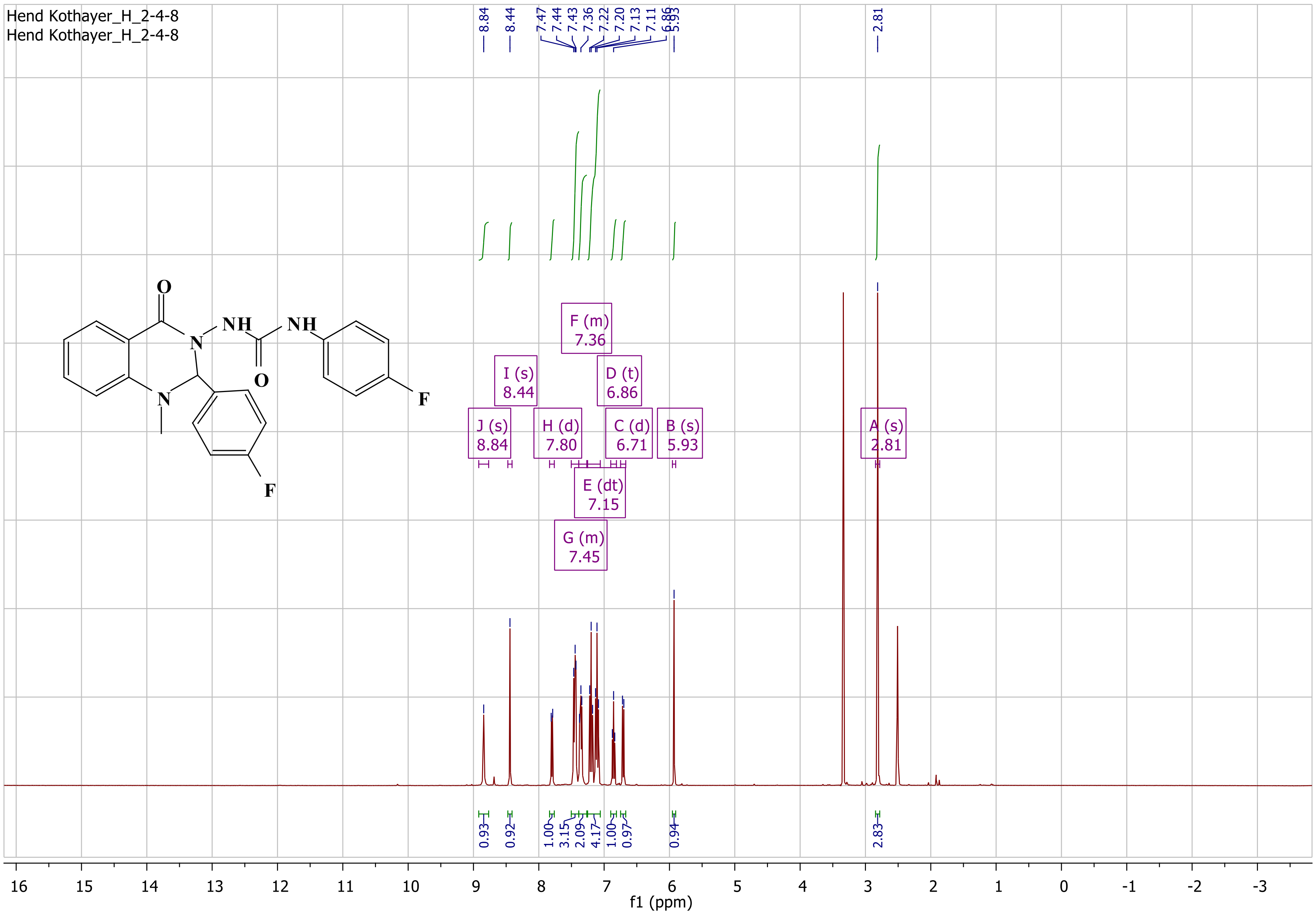

Hend Kothayer_H_2-4-8
Hend Kothayer_H_2-4-8
8.84
8.44
7.47
7.44
7.43
7.36
7.22
7.20
7.13
7.11
6.86
5.93
2.81
O
NH
NH
N
O
N
F
F
F (m)
7.36
I (s)
8.44
D (t)
6.86
J (s)
8.84
H (d)
7.80
C (d)
6.71
B (s)
5.93
A (s)
2.81
E (dt)
7.15
G (m)
7.45
0.93
0.92
1.00
3.15
2.09
4.17
1.00
0.97
0.94
2.83
16
15
14
13
12
11
10
9
8
7
6
5
4
3
2
1
0
-1
-2
-3
f1 (ppm)

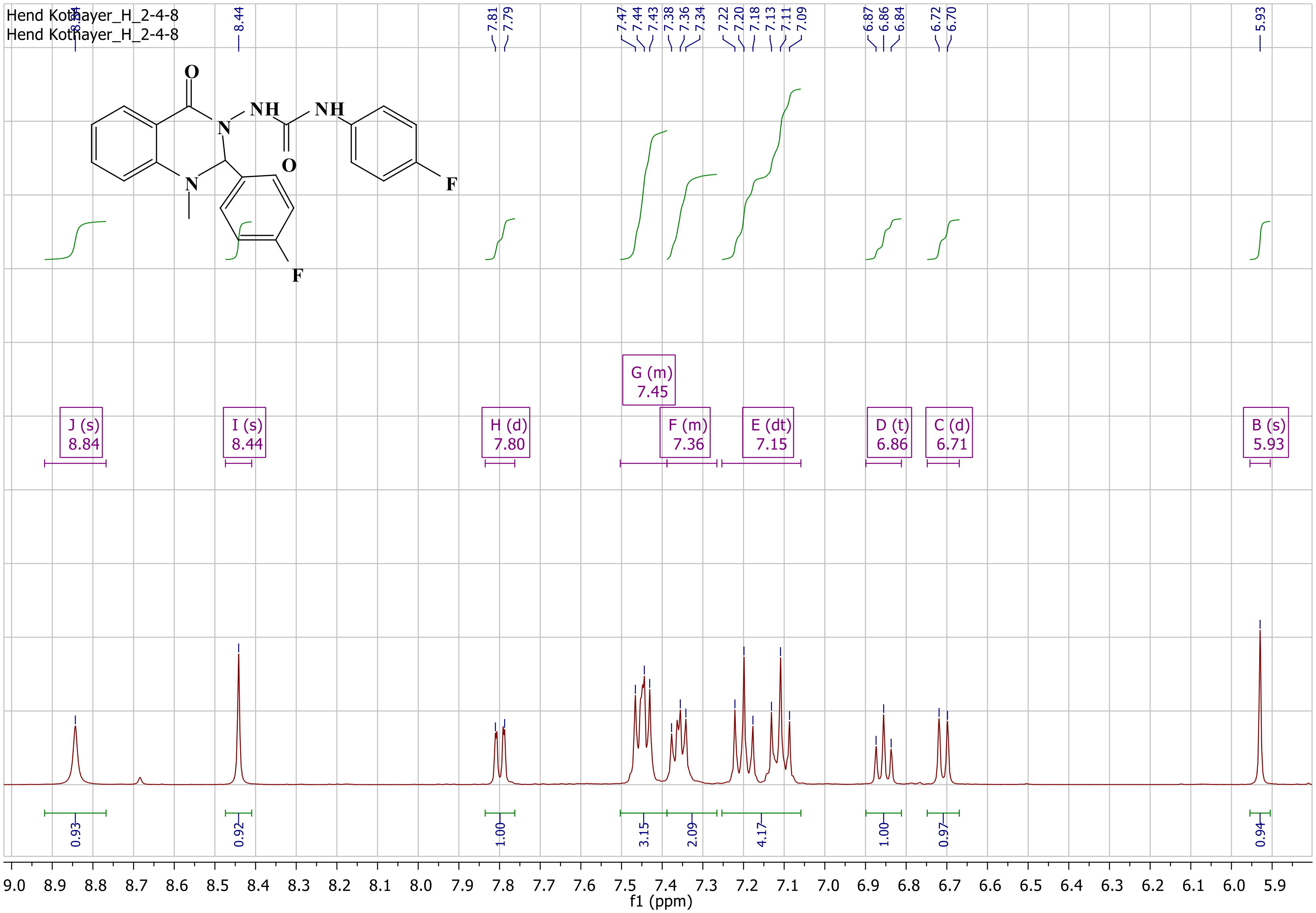
Hend Kothayer_H_2-4-8
Hend Kothayer_H_2-4-8
8.84
8.44
7.81
7.79
7.47
7.44
7.43
7.38
7.36
7.34
7.22
7.20
7.18
7.13
7.11
7.09
6.87
6.86
6.84
6.72
6.70
5.93
O
NH
NH
N
O
N
F
F
G (m) 7.45
J (s) 8.84
I (s) 8.44
H (d) 7.80
F (m) 7.36
E (dt) 7.15
D (t) 6.86
C (d) 6.71
B (s) 5.93
0.93
0.92
1.00
3.15
2.09
4.17
1.00
0.97
0.94
9.0 8.9 8.8 8.7 8.6 8.5 8.4 8.3 8.2 8.1 8.0 7.9 7.8 7.7 7.6 7.5 7.4 7.3 7.2 7.1 7.0 6.9 6.8 6.7 6.6 6.5 6.4 6.3 6.2 6.1 6.0 5.9
f1 (ppm)

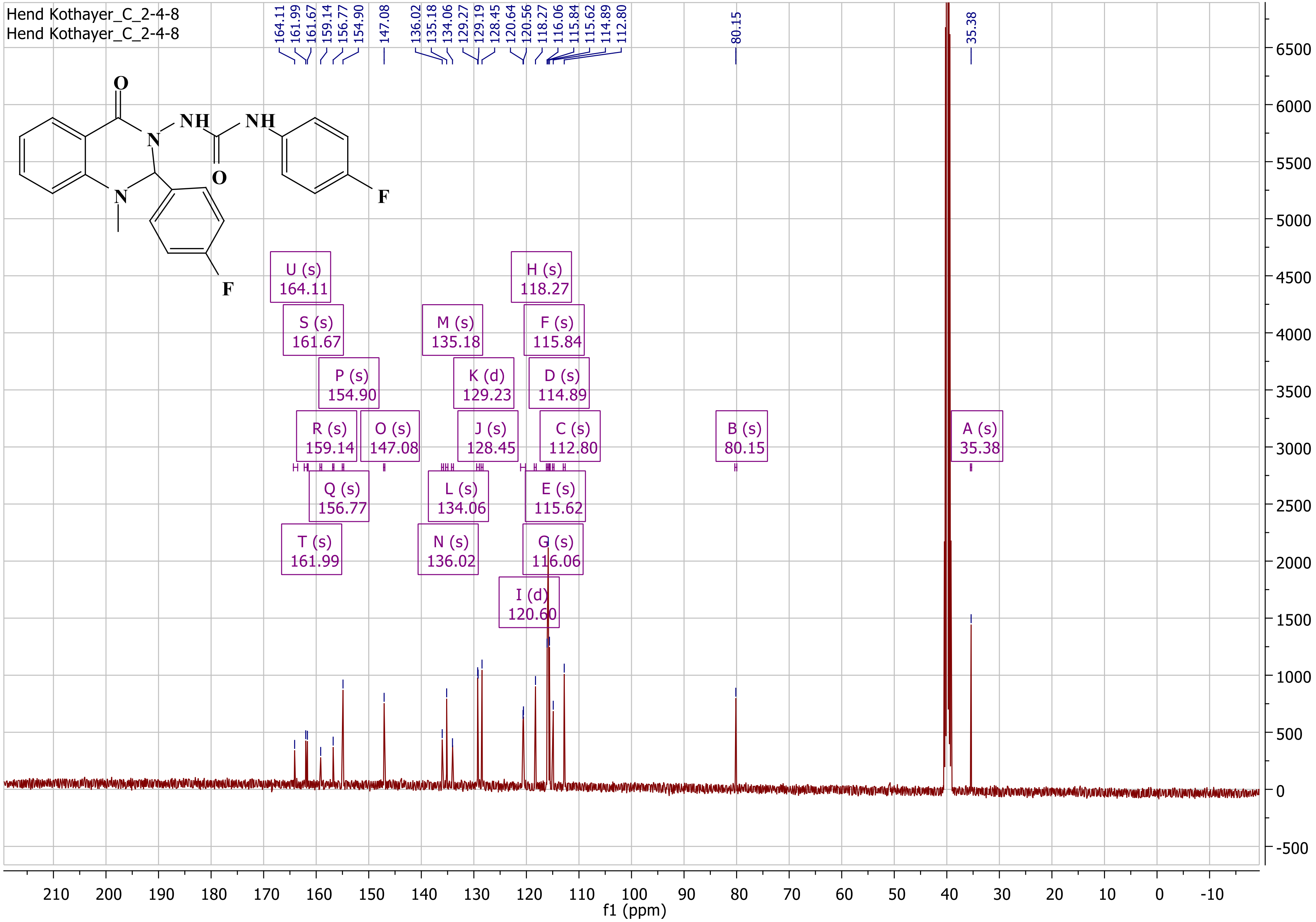

Hend Kothayer_C_2-4-8
Hend Kothayer_C_2-4-8
164.11
161.99
161.67
159.14
156.77
154.90
147.08
136.02
135.18
134.06
129.27
129.19
128.45
120.64
120.56
118.27
116.06
115.84
115.62
114.89
112.80
80.15
35.38
U (s) 164.11
S (s) 161.67
P (s) 154.90
R (s) 159.14
O (s) 147.08
Q (s) 156.77
T (s) 161.99
M (s) 135.18
K (d) 129.23
J (s) 128.45
L (s) 134.06
N (s) 136.02
H (s) 118.27
F (s) 115.84
D (s) 114.89
C (s) 112.80
E (s) 115.62
G (s) 116.06
I (d) 120.60
B (s) 80.15
A (s) 35.38
f1 (ppm)

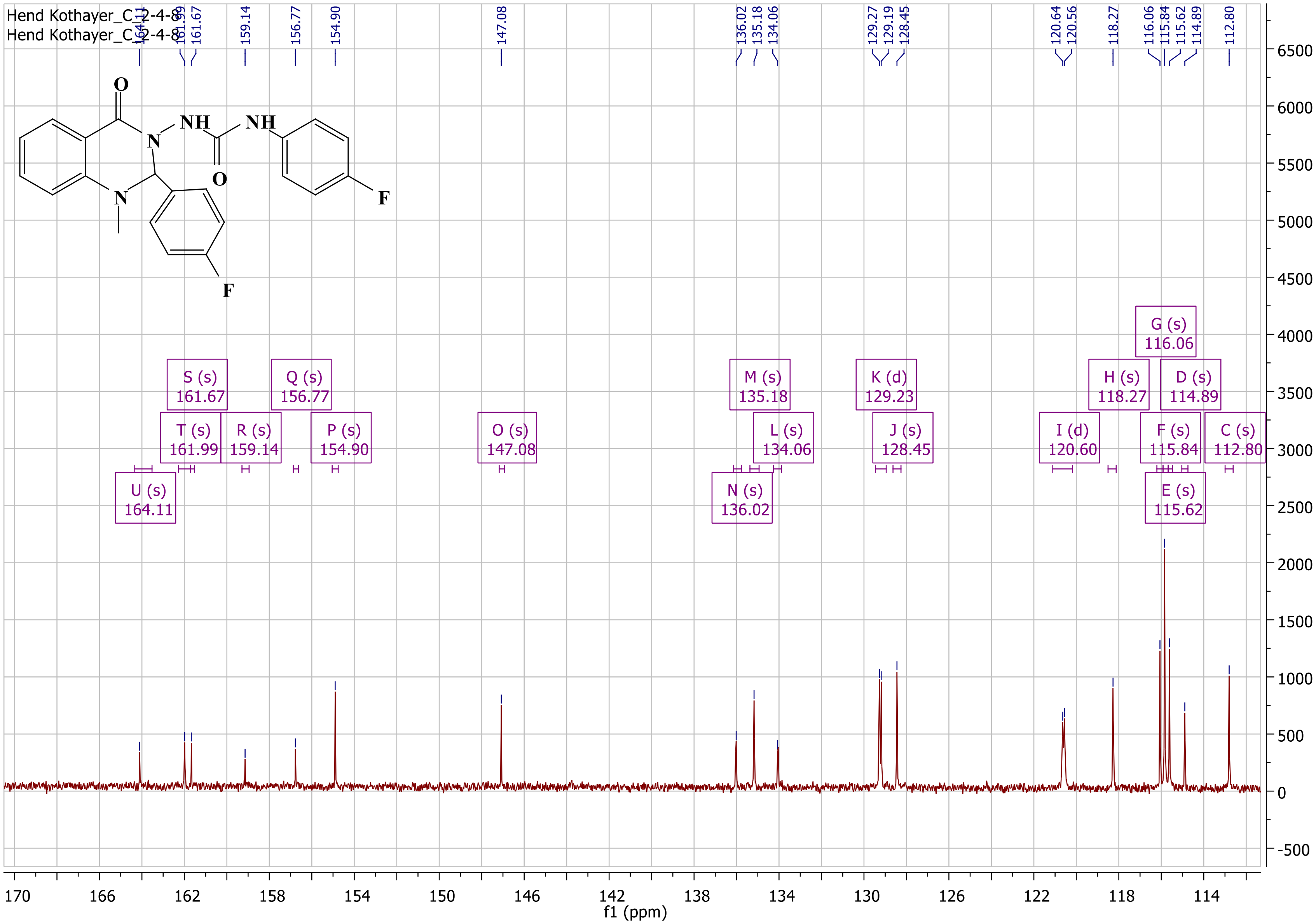
Hend Kothayer_C2-4-8
Hend Kothayer_C2-4-8
164.11
161.99
161.67
159.14
156.77
154.90
147.08
136.02
135.18
134.06
129.27
129.19
128.45
120.64
120.56
118.27
116.06
115.84
115.62
114.89
112.80
U (s) 164.11
T (s) 161.99
S (s) 161.67
R (s) 159.14
Q (s) 156.77
P (s) 154.90
O (s) 147.08
N (s) 136.02
M (s) 135.18
L (s) 134.06
K (d) 129.23
J (s) 128.45
I (d) 120.60
H (s) 118.27
G (s) 116.06
F (s) 115.84
E (s) 115.62
D (s) 114.89
C (s) 112.80
f1 (ppm)

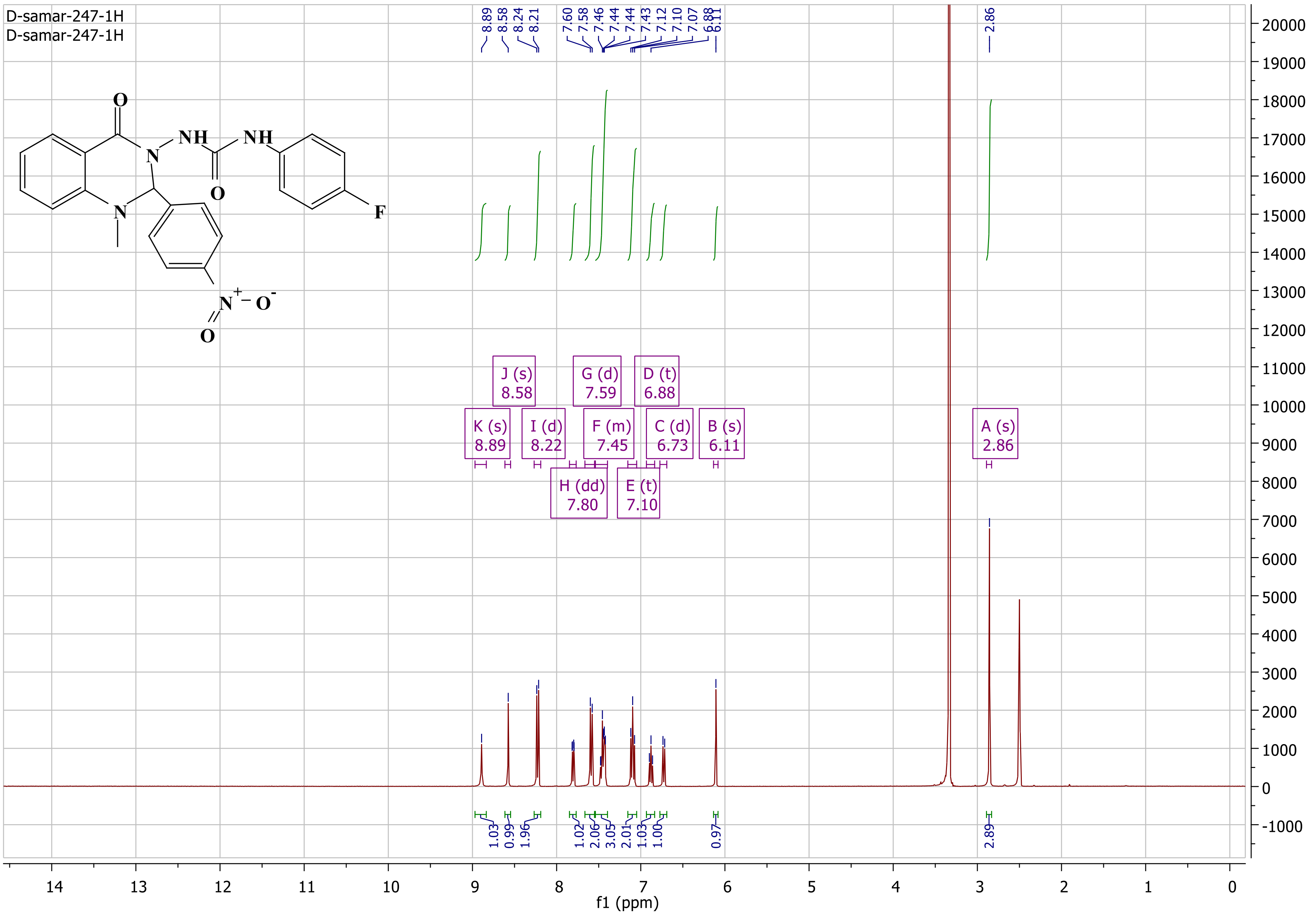
D-samar-247-1H
D-samar-247-1H
8.89
8.58
8.24
8.21
7.60
7.58
7.46
7.44
7.44
7.43
7.12
7.10
7.07
6.88
6.11
2.86
J (s) 8.58
G (d) 7.59
D (t) 6.88
K (s) 8.89
I (d) 8.22
F (m) 7.45
C (d) 6.73
B (s) 6.11
A (s) 2.86
H (dd) 7.80
E (t) 7.10
1.03
0.99
1.96
1.02
2.06
3.05
2.01
1.03
1.00
0.97
2.89
O
NH
NH
N
O
F
N
N+
O-
O
f1 (ppm)

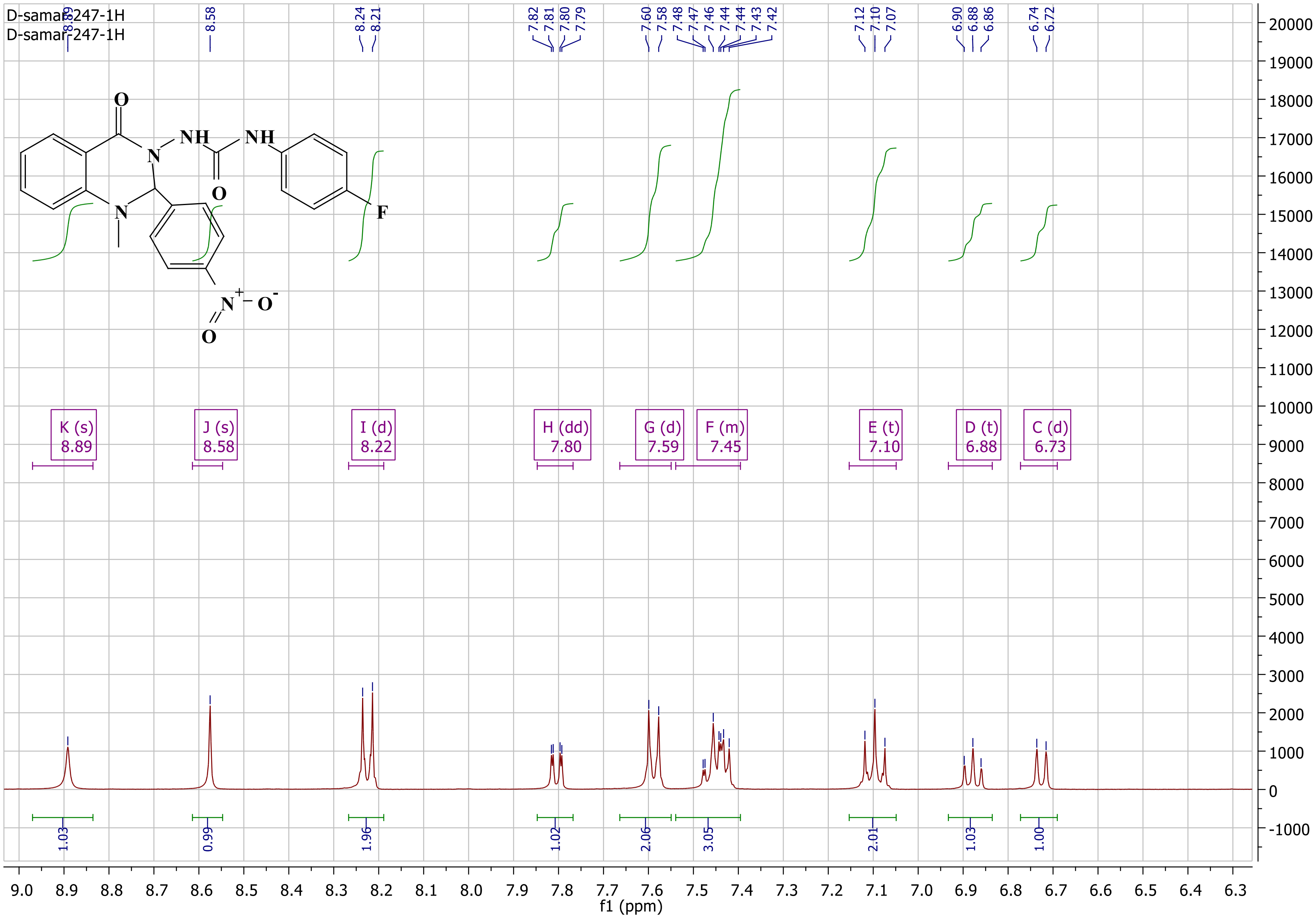
D-samar-247-1H
D-samar-247-1H
8.89
8.58
8.24
8.21
7.82
7.81
7.80
7.79
7.60
7.58
7.48
7.47
7.46
7.44
7.44
7.43
7.42
7.12
7.10
7.07
6.90
6.88
6.86
6.74
6.72
O
NH
NH
N
O
F
N
N+
O-
O
K (s) 8.89
J (s) 8.58
I (d) 8.22
H (dd) 7.80
G (d) 7.59
F (m) 7.45
E (t) 7.10
D (t) 6.88
C (d) 6.73
1.03
0.99
1.96
1.02
2.06
3.05
2.01
1.03
1.00
f1 (ppm)

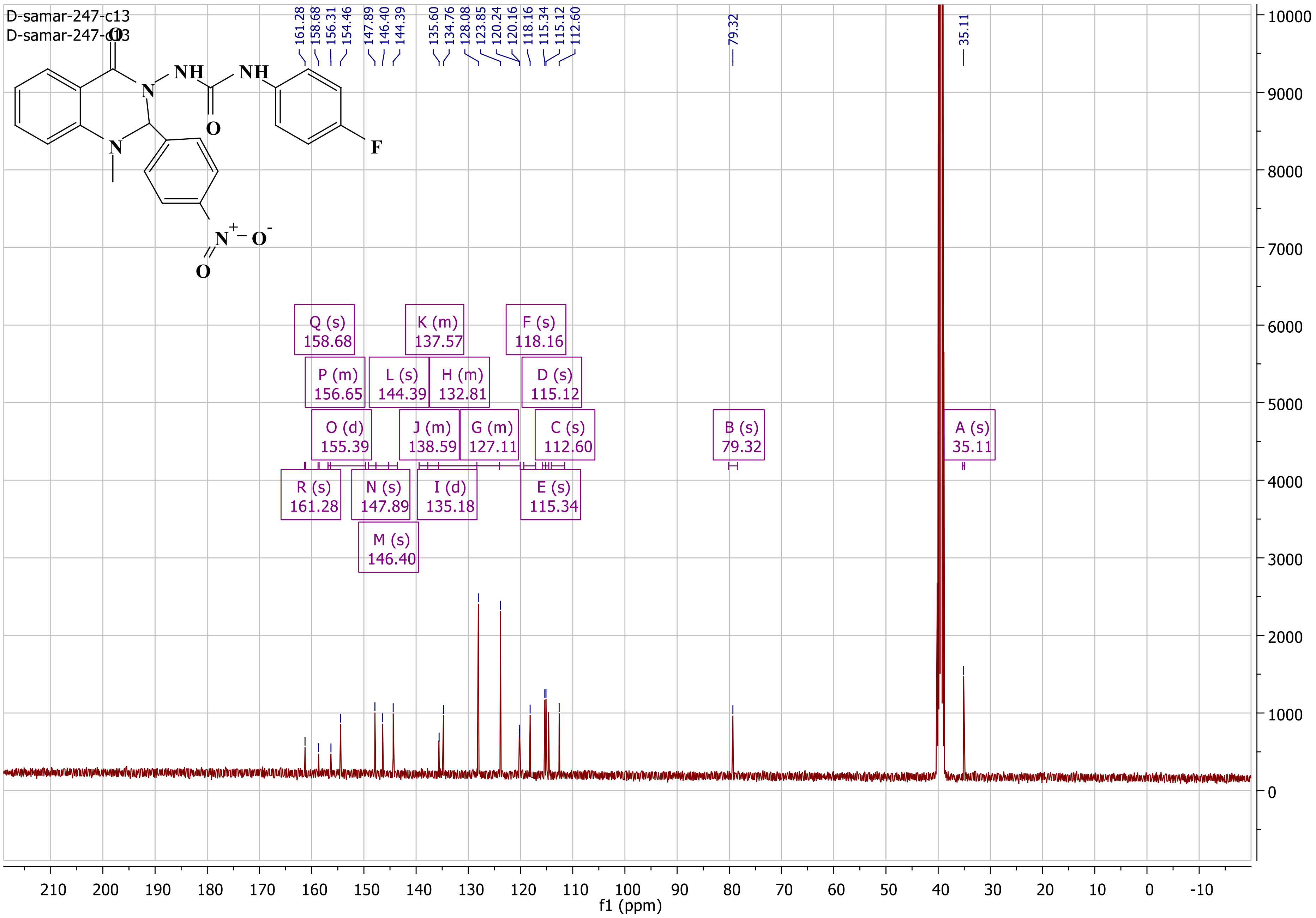

D-samar-247-c13
D-samar-247-c13
NH
NH
N
O
O
N
F
N+
O-
O
161.28
158.68
156.31
154.46
147.89
146.40
144.39
135.60
134.76
128.08
123.85
120.24
120.16
118.16
115.34
115.12
112.60
79.32
35.11
Q (s) 158.68
K (m) 137.57
F (s) 118.16
P (m) 156.65
L (s) 144.39
H (m) 132.81
D (s) 115.12
O (d) 155.39
J (m) 138.59
G (m) 127.11
C (s) 112.60
B (s) 79.32
A (s) 35.11
R (s) 161.28
N (s) 147.89
I (d) 135.18
E (s) 115.34
M (s) 146.40
f1 (ppm)

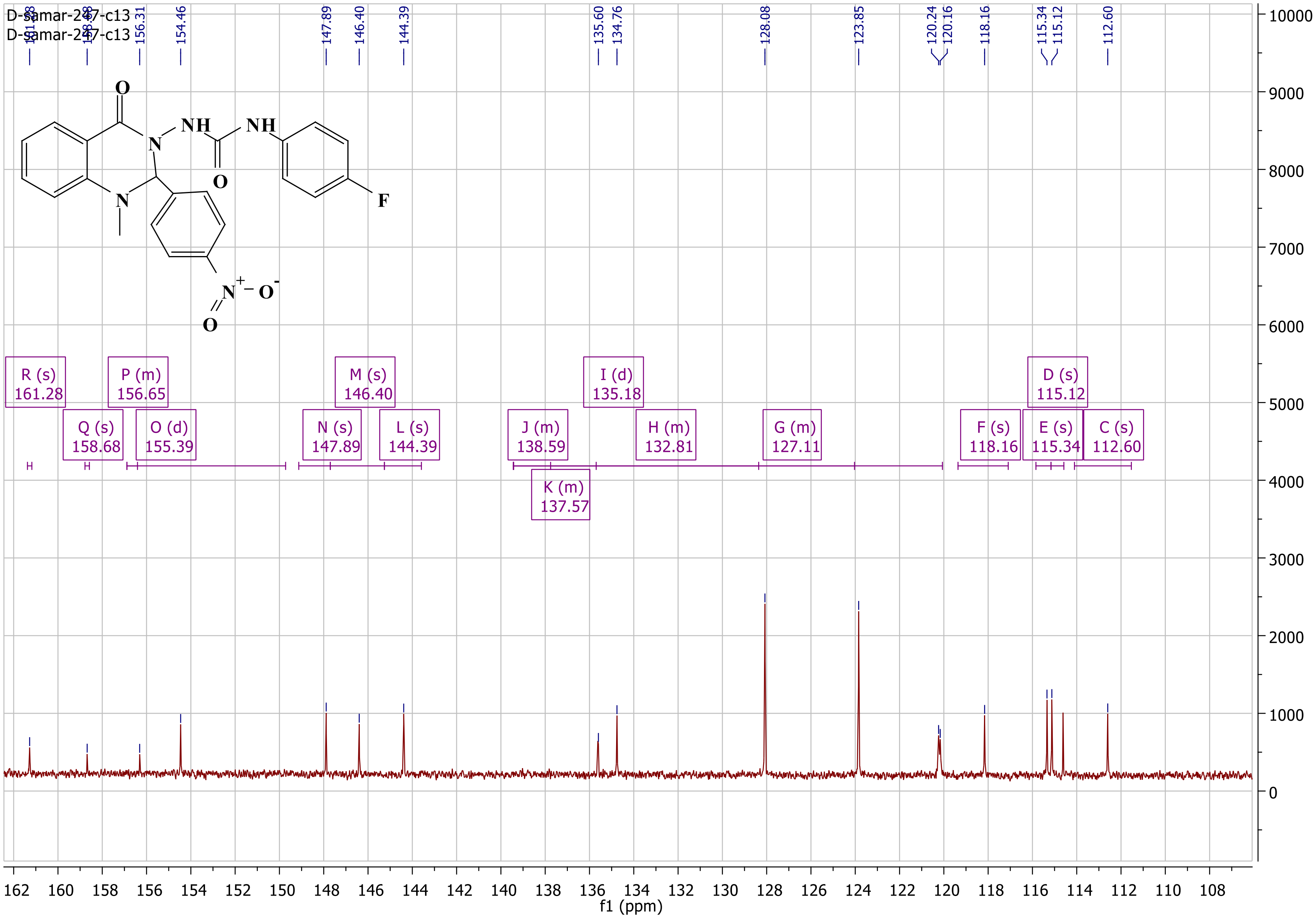

D-samar-247-c13
D-samar-247-c13
156.31
154.46
147.89
146.40
144.39
135.60
134.76
128.08
123.85
120.24
120.16
118.16
115.34
115.12
112.60
R (s) 161.28
Q (s) 158.68
P (m) 156.65
O (d) 155.39
N (s) 147.89
M (s) 146.40
L (s) 144.39
J (m) 138.59
K (m) 137.57
I (d) 135.18
H (m) 132.81
G (m) 127.11
F (s) 118.16
E (s) 115.34
D (s) 115.12
C (s) 112.60
f1 (ppm)

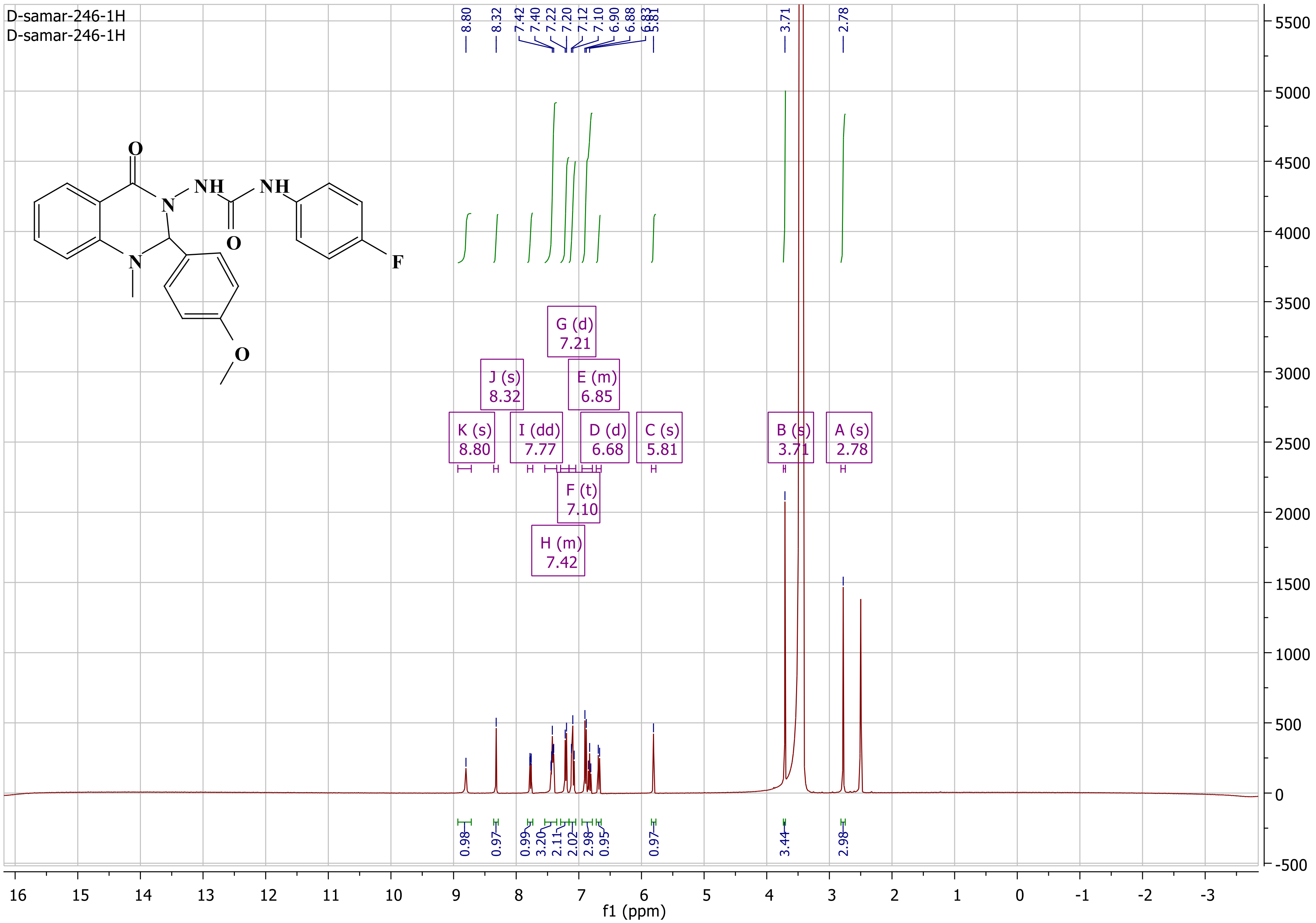
D-samar-246-1H
D-samar-246-1H
8.80
8.32
7.42
7.40
7.22
7.20
7.12
7.10
6.90
6.88
6.83
5.81
3.71
2.78
G (d) 7.21
J (s) 8.32
E (m) 6.85
K (s) 8.80
I (dd) 7.77
D (d) 6.68
C (s) 5.81
B (s) 3.71
A (s) 2.78
F (t) 7.10
H (m) 7.42
0.98
0.97
0.99
3.20
2.11
2.02
2.98
0.95
0.97
3.44
2.98
f1 (ppm)

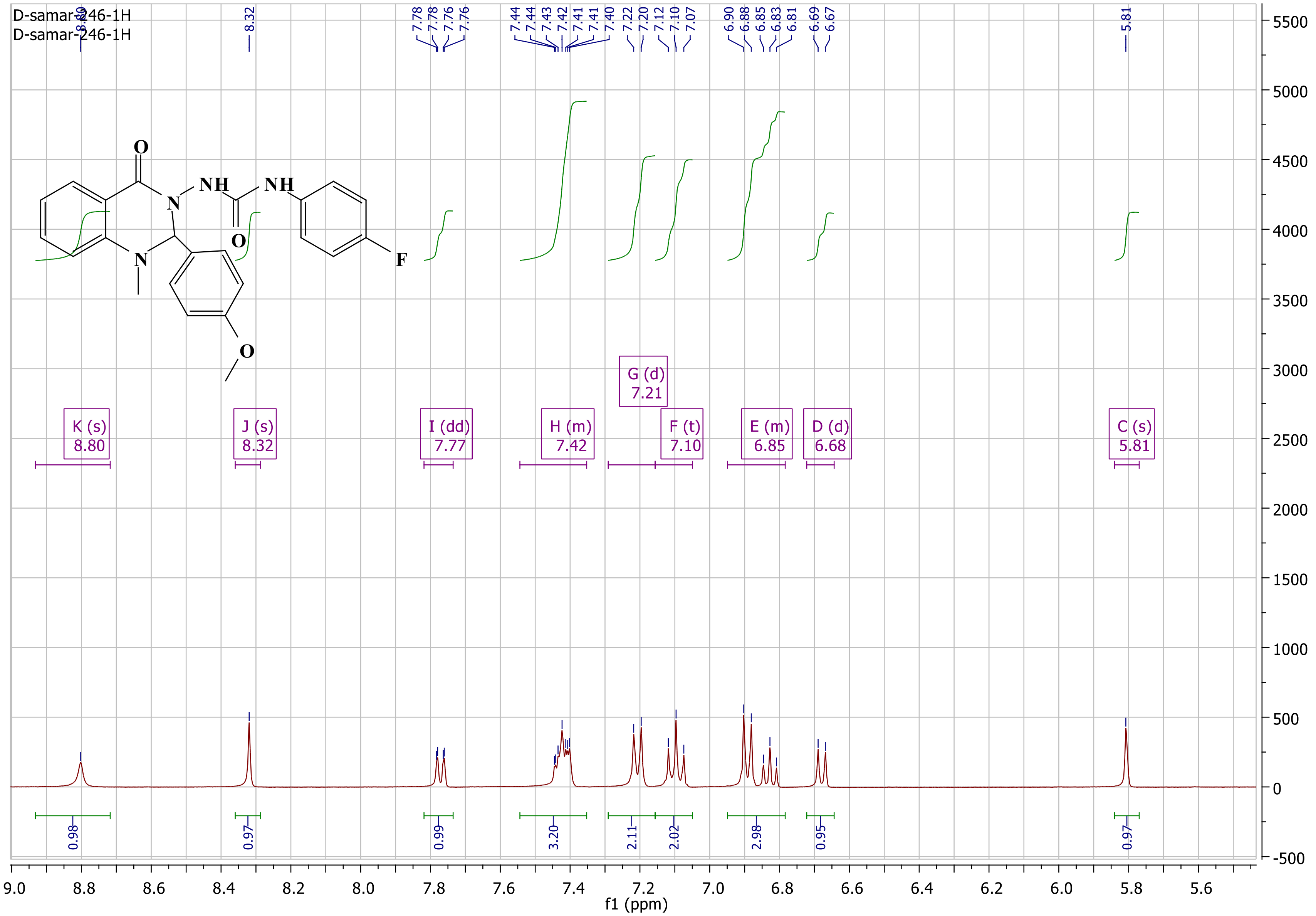
D-samar-246-1H
D-samar-246-1H
8.80
8.32
7.78
7.78
7.76
7.76
7.44
7.44
7.43
7.42
7.41
7.41
7.40
7.22
7.20
7.12
7.10
7.07
6.90
6.88
6.85
6.83
6.81
6.69
6.67
5.81
O
NH
NH
N
O
F
N
O
K (s)
8.80
J (s)
8.32
I (dd)
7.77
H (m)
7.42
G (d)
7.21
F (t)
7.10
E (m)
6.85
D (d)
6.68
C (s)
5.81
0.98
0.97
0.99
3.20
2.11
2.02
2.98
0.95
0.97
f1 (ppm)

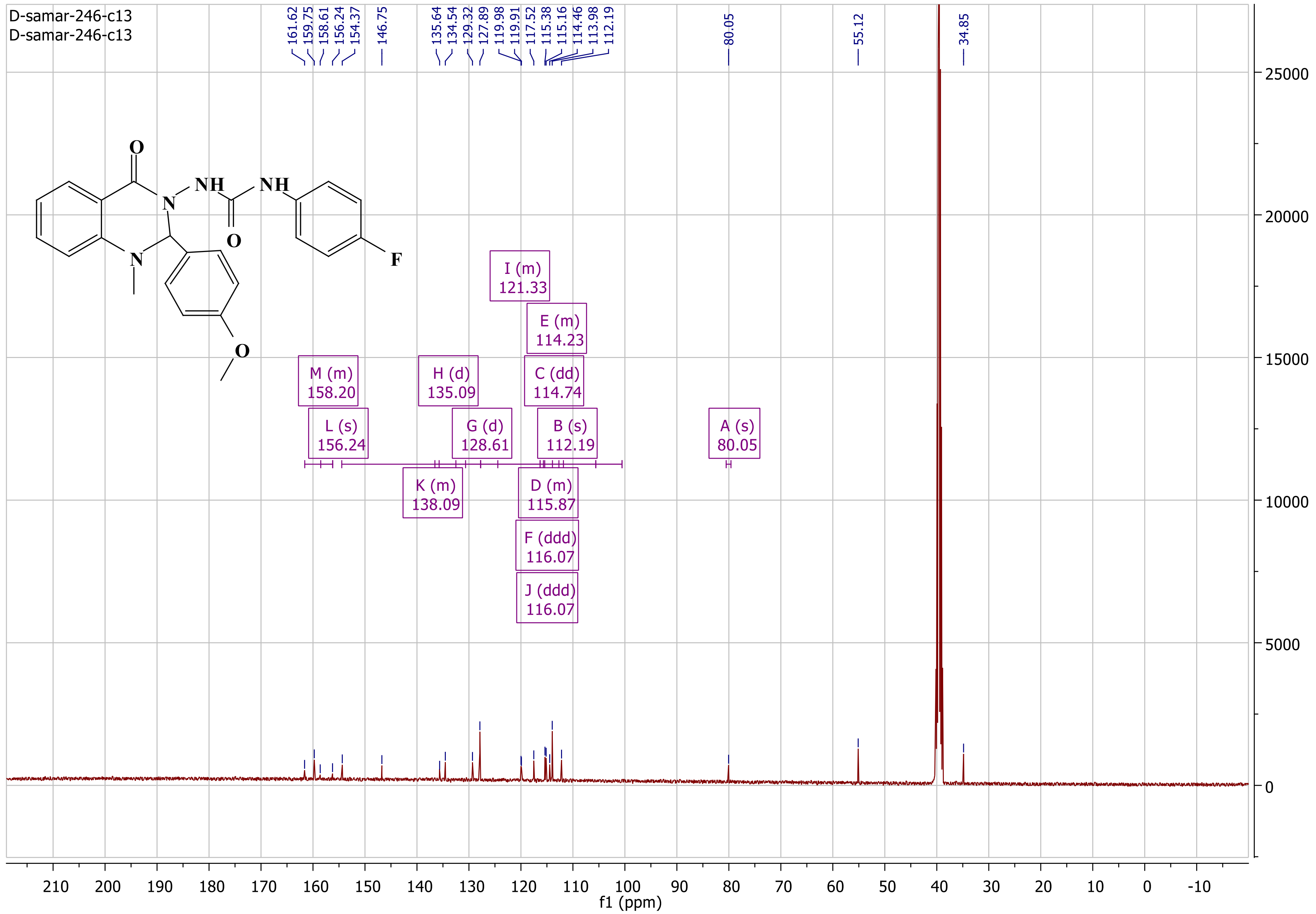
D-samar-246-c13
D-samar-246-c13
161.62
159.75
158.61
156.24
154.37
146.75
135.64
134.54
129.32
127.89
119.98
119.91
117.52
115.38
115.16
114.46
113.98
112.19
80.05
55.12
34.85
I (m) 121.33
E (m) 114.23
M (m) 158.20
H (d) 135.09
C (dd) 114.74
L (s) 156.24
G (d) 128.61
B (s) 112.19
A (s) 80.05
K (m) 138.09
D (m) 115.87
F (ddd) 116.07
J (ddd) 116.07
f1 (ppm)

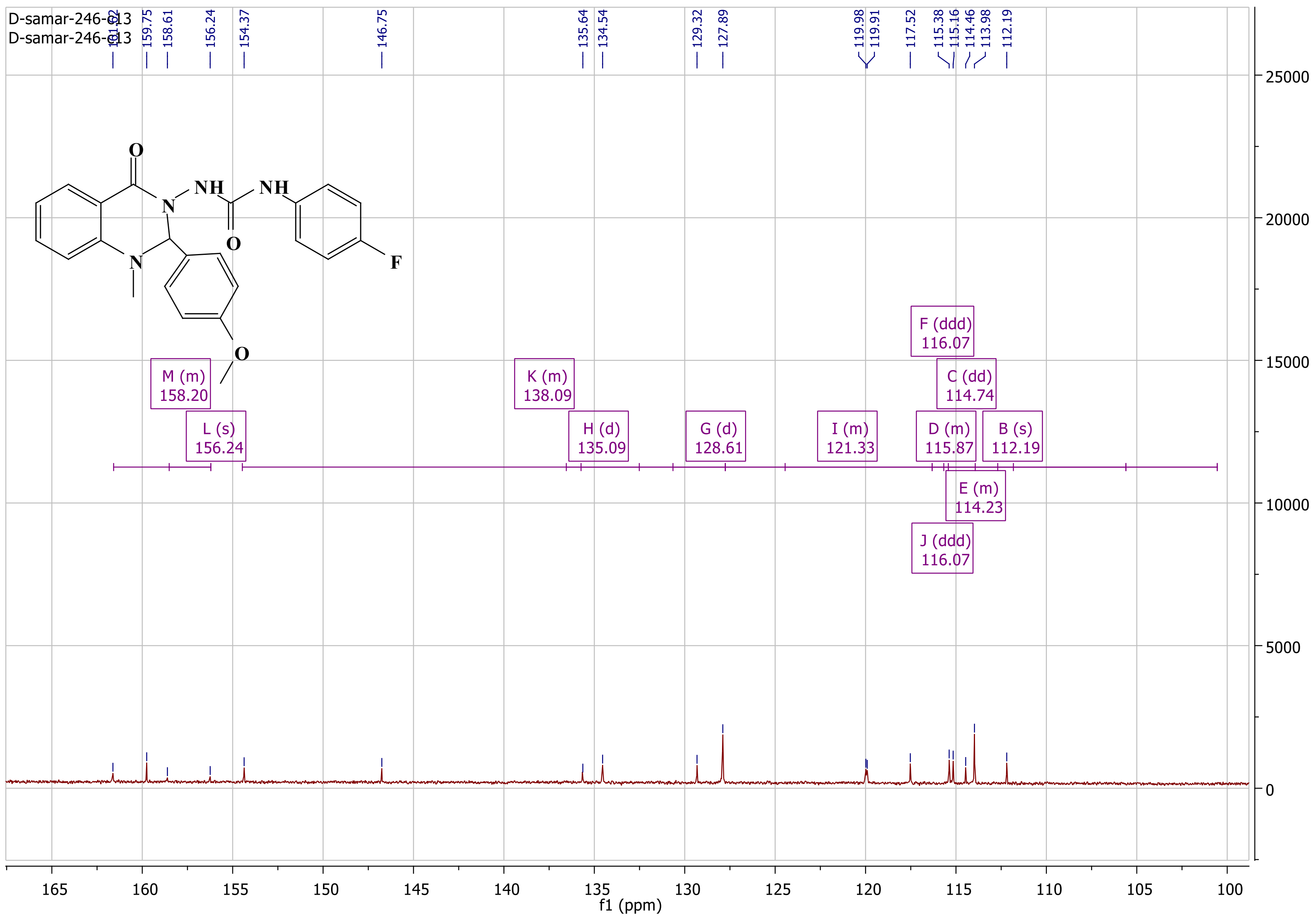
D-samar-246-c13
D-samar-246-c13
161.62
159.75
158.61
156.24
154.37
146.75
135.64
134.54
129.32
127.89
119.98
119.91
117.52
115.38
115.16
114.46
113.98
112.19
M (m) 158.20
L (s) 156.24
K (m) 138.09
H (d) 135.09
G (d) 128.61
I (m) 121.33
F (ddd) 116.07
C (dd) 114.74
D (m) 115.87
B (s) 112.19
E (m) 114.23
J (ddd) 116.07
O
NH
NH
N
O
N
F
O
f1 (ppm)

Table 1: Molecular docking details of target compounds with both (1CX2 and 1EQG)								
	Docking of target compounds and 1CX2 (COX2)				Docking of target compounds and 1EQG (COX1)			
**Code**	**Amino acid interactions**	**Docking scores (Kcal/mol)**	**Type of bond**	**Number of bond**	**Amino acid interactions**	**Docking scores (Kcal/mol)**	**Type of bond**	**Number of Bond**
**6a**	-	-6.4628	-	-	Arg120, Tyr355	-5.8739	2 HB [a]	2
**6b**	-	-6.2910	-	-	Arg120	-5.3863	HB [a]	1
**6c**	Arg120	-6.4486	1 arene-H [b]	1	Tyr355	-6.3733	1 HB [a], 1 arene-H [b]	2
**6d**	Arg120, Ser353	-6.7428	1 arene-H [b], 1 arene-cation	2	-	-6.1979	-	-
**6e**	Arg120, Val349 Tyr385	-8.4697	2 HB [a], 1 arene-cation	3	Arg120	-6.1528	HB [a]	1
**6f**	Val349, Met522, Ala527	-6.0983	3 HB [a]	3	Arg120, Tyr355	-6.1194	2 HB [a] , 1 arene-cation	3
**6g**	Val349	-6.1788	1 HB [a]	1	Arg120, Tyr355	-6.3907	2HB [a]	2
**6h**	-	-6.8253	-	-	Tyr355	-6.1801	1 HB [a], 1 arene-H [b]	2
**6i**	Arg120, Tyr355	-7.5751	1 arene-H [b], 1 arene-cation	2	Val116	-5.9169	HB [a]	1
**6j**	Ser353	-7.1059	1 HB [a], 1 arene-H [b]	2	Arg120	-6.5881	HB [a]	1
**6k**	Tyr355	-7.4480	1 HB [b]	1	-	-	-	-
**6l**	Arg120	-6.4717	1 arene-cation[b]	1	Arg120, Tyr355	-5.8911	HB [a]	2
**6m**	Met522, Val523	-7.7837	2 HB [a]	2	Arg120, Tyr355	-5.8813	2 HB [a], 1 arene-H [b]	2
**6n**	Ser353, Val349, Ala527, Tyr355, Trp387, Leu531	-6.7307	3 HB [a], 3 arene-H[b]	6	-	-6.2822	-	-
**6o**	Leu531	-5.4624	1 arene-H[b]	1	-	-5.6081	-	-
**6p**	Ser530	-7.7614	1 HB[a], 1 arene-H[b]	2	Tyr355	-5.9729	Arene-H [b]	1
**SC-558**	His90, Arg120, Leu352	-9.2971	3 HB [a]	3	-	-	**-**	**-**
**Ibuprofen**	-	-	**-**	**-**	Arg120, Tyr355	-7.4630	HB [a]	3

**Table 2: Molecular docking details of target compounds with (15-LOX)**				
	**Docking of target compounds and 4NRE**			
**Code**	**Amino acid interactions**	**Docking scores (Kcal/mol)**	**Type of bond**	**Number of bond**
**6a**	Leu420, Arg429	-5.8295	arene-H[b]	2
**6b**	Leu419, Val426	-5.9878	2 HB [a]	2
**6c**	Leu419	-7.0847	HB [a]	1
**6d**	Leu201, Asp602	-6.7188	1 HB[a], 1 arene-H[b]	2
**6e**	Leu420, Gln425	-6.7684	1 HB[a], 1 arene-H[b]	2
**6f**	Phe184, Gly189	-6.5446	2 arene-H[b]	2
**6g**	Leu420	-6.1768	1 arene-H[b]	1
**6h**	Arg429	-6.7697	1 HB [a]	1
**6i**	Phe184, Ala606	-6.9536	1 HB [a], 1 arene-H[b]	2
**6j**	His378, Leu420, Arg429	-7.0450	1 arene-H[b],1 H-arene, 1 arene-cation	3
**6k**	Leu419, Leu420	-5.9000	1 HB [a], 1 arene-H[b]	2
**6l**	Leu419	-6.4064	2 HB[a]	2
**6m**	Gln425, Val426	-6.3528	2 HB[a]	2
**6n**	Leu419, Val426	-6.6561	2 HB[a]	2
**6o**	Phe192, Arg429	-6.6249	1 HB[a], 1 arene-arene	2
**6p**	Phe192, Asp602	-6.7753	1 HB[a], 1 arene-arene	2
**C8E**	Arg429	-8.6019	1 HB[a]	1

Table 3: Molecular docking details of target compounds with both (1M17 (wild EGFR) and 5EDQ T790M/L858R EGFR

	Docking of target compounds and 1M17				Docking of target compounds and 5EDQ			
**Code**	**Amino acid interactions**	**Docking scores (Kcal/mol)**	**Type of bond**	**Number of bond**	**Amino acid interactions**	**Docking scores (Kcal/mol)**	**Type of bond**	**Number of Bond**
**6a**	Leu694, Met769	-6.9806	1 HB[a], 1 arene-H	2	Leu718, Met790, Gly798	-6.6524	1 HB[a], 2 arene-H	3
**6b**	Leu694, Thr830	-6.8483	1 HB[a], 1 arene-H	2	Leu718	-6.5123	1 arene-H	1
**6c**	Leu694, Asp831	-7.0625	1 HB[a], 1 arene-H	2	Met766, Thr854	-7.3728	2 HB[a]	2
**6d**	Leu694, Gly772, Asp831	-7.1152	1 HB[a], 2 arene-H	3	Lys745, Met790	-7.6660	2 HB[a], 1 arene-H	3
**6e**	Leu694, Met769, Gly772	-7.3867	1 HB[a], 2 arene-H	3	Leu718, Val726, Lys745, Glu762, Met790	-7.8441	3 HB[a], 2 arene-H	5
**6f**	Leu694, Met769	-6.9494	1 HB[a], 1 arene-H	2	Leu718, Leu844	-7.4767	2 arene-H	2
**6g**	Leu694, Gly772	-6.9199	1 arene-H	2	Met790	-7.0191	2 HB[a]	2
**6h**	Asp831	-6.6860	1 HB[a]	1	Met790, Thr854	-7.4117	2 HB[a]	2
**6i**	Leu694, Gly772, Asp831	-7.3335	1 HB[a], 2 arene-H	3	Thr854	-8.1780	1 HB[a]	1
**6j**	Leu694	-7.0745	1 arene-H	1	Lys745, Met790, Thr854	-7.8763	3 HB[a], 1 arene-H	4
**6k**	Gly772	-6.8238	1 arene-H	1	-	-6.8007	-	-
**6l**	Leu694, Met769	-6.7953	1 HB[a], 1 arene-H	2	Leu718	-6.7805	1 arene-H	1
**6m**	Leu694, Met769	-7.0591	1 HB[a], 1 arene-H	2	Leu718, Val726, Lys745, Glu762	-7.7122	2 HB[a], 2 arene-H	4
**6n**	Met769	-6.8364	1 HB[a]	1	Met790, Thr854	-7.7911	2 HB[a]	2
**6o**	Met769, Asp831	-7.1365	2 HB[a]	2	Lys745	-7.7064	1 HB[a]	1
**6p**	Leu694, Met769	-7.4186	1 HB[a], 1 arene-H	2	Leu718, Lys745, Met790, Gly796, Thr854	-7.8727	2 HB[a], 3 arene-H	5
**Erlotinib**	Leu694, Gln767, Met769	-8.2337	2 HB[a], 1 arene-H	3				
**5N3**					Val726, Lys745, Ala743	-7.0693	2 HB[a], 2 arene-H	4

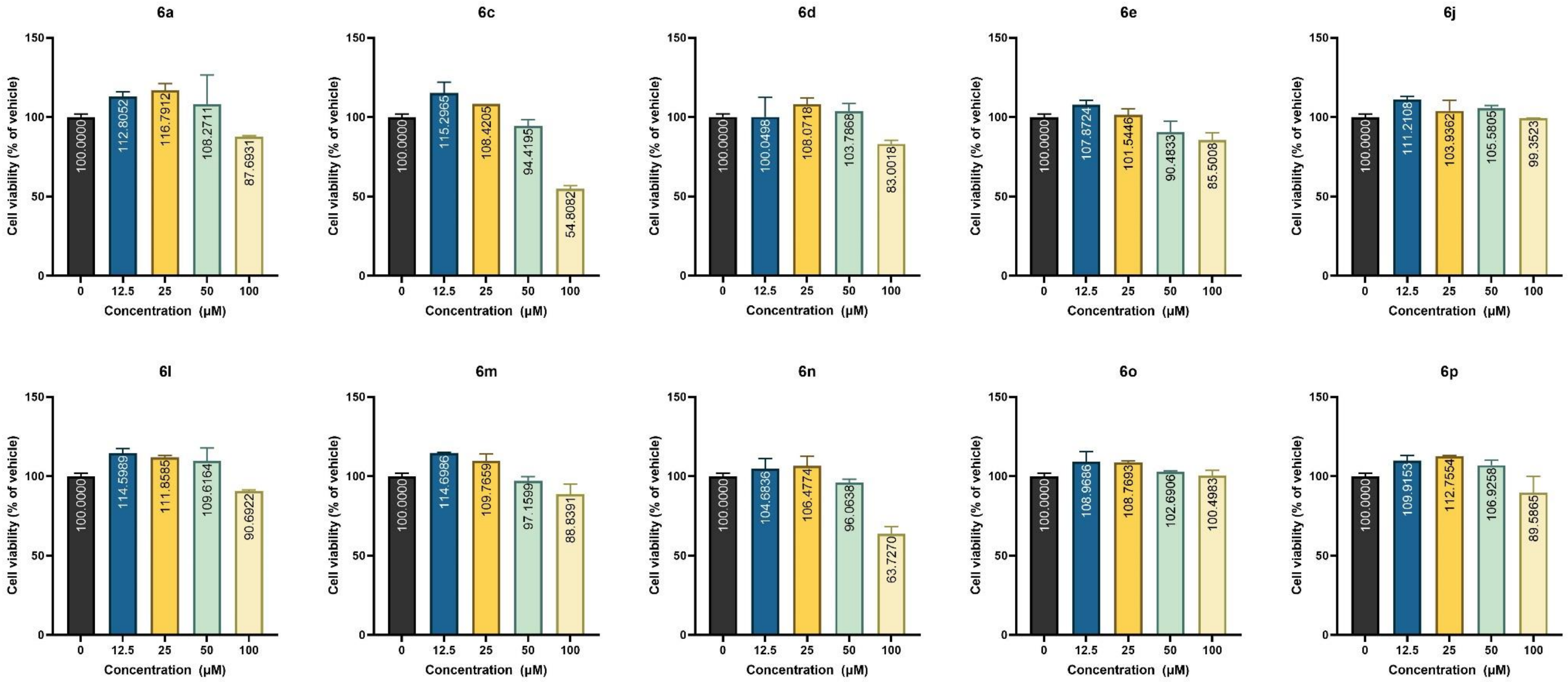

Fig.1. Cell viability assay for compounds**(6a, 6c, 6d, 6e, 6j, 6l, 6m, 6n, 6o, and 6p)** using normal RAW 264.7 cells at different concentrations (12.5, 25, 50, and 100 µM)
